# Conventional Medicinal Uses, Phytoconstituents, and Biological Activities of *Euphorbia officinarum* L.: A Systematic Review

**DOI:** 10.1155/2022/9971085

**Published:** 2022-01-06

**Authors:** Imane Chamkhi, Mohamed Hnini, Jamal Aurag

**Affiliations:** ^1^Geo-Biodiversity and Natural Patrimony Laboratory, Scientific Institute, Mohammed V University in Rabat, Rabat, Morocco; ^2^Microbiology and Molecular Biology Team, Center of Plant and Microbial Biotechnology, Biodiversity and Environment, Faculty of Sciences, Mohammed V University of Rabat, Avenue Ibn Battouta BP 1014, Rabat 10000, Morocco

## Abstract

The Moroccan endemic plant *Euphorbia officinarum* is a traditional medicinal plant, known locally as “Daghmus.” Plants in the genus *Euphorbia* are well known for the chemical diversity of their diterpenoids and isoprenoid constituents, which perform many activities such as cytotoxic, antimicrobial, and anti-inflammatory activities, as well as different biological properties, that cannot be overlooked. The effect of bioactive compounds (antiviral, antidiabetic, anticancer, and antioxidant). *Euphorbia officinarum* is an important conventional medicine for the treatment of various conditions, including skin and ophthalmological diseases. It is also used against human pathogens (intestinal parasites). *E. officinarum* latex is the major part of the plant used for conventional medicine and synthesizing new bioactive compounds. The characterization and isolation of its components are necessary to exploiting and enhancing its therapeutic potential. However, to the best of our knowledge, no review is available to date. In order to have and define a research question, we adopt a strategy by considering the items of the PRISMA checklist. Therefore, this review aims to cover *E. officinarum* taxonomy, botanical description, distribution, conventional uses, and phytochemical compounds of this plant, including the biological activities of compounds isolated and of these semisynthesized compounds. This article provides a foundation for any further studies from this plant.

## 1. Introduction

Euphorbiaceae family is the fourth largest family of flowering plants, englobing 6000 species of plants, ranging from tiny annual weeds to giant trees, distributed all over the world except for the arctic and antarctic. Some present an economic income and are essential to modern life. This vast number of species is divided up into five subfamilies and a number of tribes and subtribes and further into 300 genera [1]. The genus *Euphorbia* is the largest in the spurge family, regroups more than 2000 species [2].

Including, the family of Euphorbiaceae contains the well-known skin irritating and tumor-promoting diterpenoids, which have tigliane, ingenane, and daphnane skeletons [3]. *Euphorbia* are succulent plants that may be found all over the world, from Africa to the Canary Islands, Madagascar, India, and the Americas; even Australia (1), this genus comprising some species used as medicinal plants due to the chemical diversity of their isoprenoid constituents, they are utilized for the treatment of skin diseases, gonorrhea, migraine, and intestinal parasites and warts [4]. Moreover, some of them are characterized by the presence of latex-containing triterpenic compounds that were used against human pathogens antiherpes [5], as well as antitumor [6], antileishmanial [7, 8], anti-inflammatory [9], and antimicrobial activities [10].

*Euphorbia officinarum* represents one of the Moroccan endemic plants, locally known under several names: “Tikiout, Zaggoum, and Daghmus” growing also in Mauritania, Western Sahara, and Algeria. In Morocco, *E. officinarum* is distributed at forms, dense stands along the Atlantic Ocean, from the south of Oued Souss to Cape Barbas, and arrives, with less frequency, as far as Cape Blanc in Mauritania [11]. The most used part of *E. officinarum* is the latex, which is used in traditional medicine to treat skin and ophthalmologic conditions [12] and intestinal parasites [13]. Moreover, in these last years, the latex of. *E officinarum* was largely used to hemisynthesize several triterpene derivatives as bioactive compounds from natural triterpenes isolated from the latex. The chemical modification of triterpenes through enzymatic or chemical synthesis often resulted in enhanced phytochemicals and pharmacological and biological properties of natural triterpene [14]. The characterization and the isolation of its components are necessary to exploiting its therapeutic potential.

Despite the importance of this plant, the pharmacological interest and activities are still weak, and there is a great need for this plant to be in-depth normalized and validated enough for further therapeutic research, mechanistic and molecular-level studies. However, to the best of our knowledge, no review covering all important aspects for *E. officinarum* plant is available to date. This review covers its taxonomy, botanical description, distribution, and conventional uses and lists all of the compounds isolated from the *E. officinarum,* and the phytochemical of the bioactive semisynthetic compounds from *E. officinarum* plant over the past few decades, notably their biological activities while this systematic review was performed in accordance with the Preferred Reporting Items for Systematic Reviews and Meta-Analyses (PRISMA).

Finally, the work purposes provide a foundation for any further studies from this plant, also to motivate other searchers to carry out other scientific studies, especially on their properties.

## 2. Research Methodology

**Figure 1:**
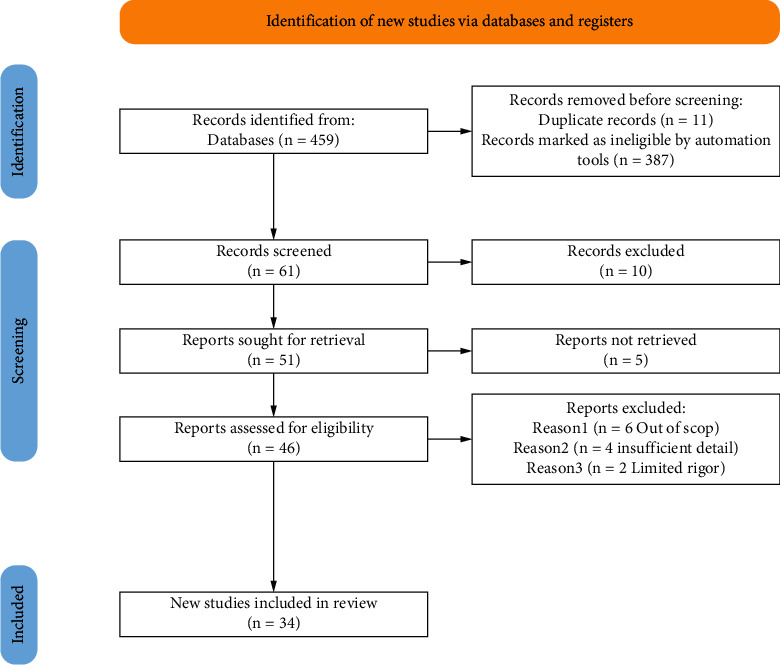
The PRISMA flow diagram showing the flow of information in the procedure of including studies in this review.

For the purpose of this study, a comprehensive literature search was undertaken to characterise and valorise the *E. officinarum* at the taxonomy level, botanical description, distribution, conventional, and phytochemical compounds of this plant; all the papers published until the end of March 2020 in literature databases such as Scopus (52), ScienceDirect (294), Google Scholar (54), and Web of Science (25) are included (the total numbers of papers was 458) (Figure 1). Several terms were to collect these data and different keywords (*Euphorbia officinarum*, biological effects of *Euphorbia officinarum,* and chemical composition of *Euphorbia officinarum)* were used to identify used publications. Data are organized in Tables 1 and 2 and highlighted.

## 3. Results and Discussion

### 3.1. Synonymy

*Euphorbia officinarum* L. is current name (ID: 1130835) (https://www.ncbi.nlm.nih.gov). The synonyms were mentioned in the encyclopedia of succulents (http://www.llifle.com) and CJBG (Conservatoire et Jardin Botanique Genève) (http://www.ville-ge.ch): Homotypic synonym(s): *Euphorbia officinarum* L. subsp. *Officinarum*, *Euphorbia officinarum* var. *Beaumieriana* (Hook. f. and Coss.) Maire (1932), and *Euphorbia beaumieriana* Hook. f. and Coss. (1874). Heterotypic synonym(s): *Euphorbia hernandez-pacheco*i Caball. (1935), *Euphorbia echinus* var. *Hernandez-pachecoi* (Caball.) Maire (1936), and *Euphorbia echinus f. Macracantha* Maire [22].

### 3.2. Botanical Description, Distribution, and Taxonomy

#### 3.2.1. Botanical Description

*Euphorbia officinarum* L. is a medicinal species characterized by a variation of the length of stems and number of ribs in different populations. This monoecious succulent is about 1–1.5 m tall. The stems are branching from the base, shorter than the branches (6–8 cm in diameter, up to 1 m tall), while the younger branches are starry in section, with 9 to 13 deep ± straight angles. The stems are up to 2 m in height and 6 cm thick. Its spines are strong, rigid, arranged in pairs on the angles, usually up to 2 cm long, and grow at intervals along the stems. The spines shield is elongated and joined in a horny margin forming a continuous line along the angle. The leaves are located on thorns, reduced to tiny tubercles located on thorns. *E. officinarum* blooms from summer to autumn and fruits from the end of summer and throughout the autumn.

**Figure 2:**
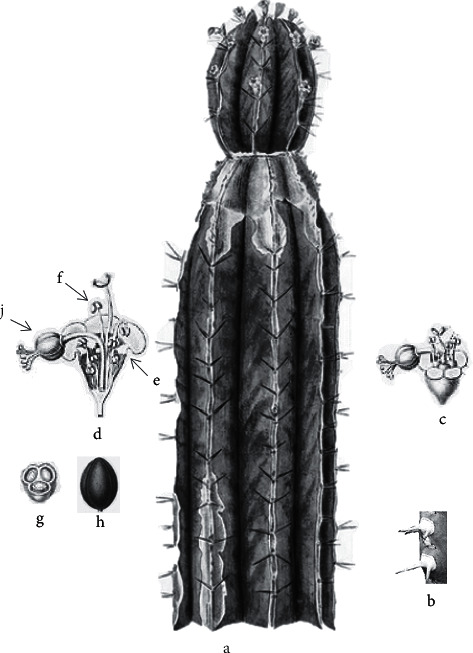
The different parts of a stem of *E. officinarum* L. (a) *E. officinarum* L., (b) thorns, (c) inflorescence, (d) vertical section of inflorescence, (e) involucre, (f) sterile flower, (j) fertile flower, (h) ovary, and (g) longitudinal section of ovary (http://www.llifle.com).

In addition, the flowers are simple, arranged in spherical structures known as cyathia. The cyathia are attractive, brownish-red [1]. While the lateral cyathia are hermaphrodites, central cyathia are usually male, solitary but sometimes up to 5 on sessile or shortly pedunculate cymes arranged in the upper half of the branches. The cyathium may be yellow to red-purple, depending on the subspecies. Furthermore, the fruits are subglobular, obtusely lobed 2.5–5  ×  2.2–4 mm hairless, smooth or finely stippled, green or red-purple. One seed per capsule variable in size depending on the subspecies, coarsely wrinkled whitish, yellowish, or greyish (http://www.llifle.com) [1] (Figure 2).

#### 3.2.2. Taxonomy

*Euphorbia officinarum* L. belongs to the family Euphorbiaceae; subfamily Euphorbioideae; Euphorbieae tribe; and *Euphorbia* genus (https://www.ncbi.nlm.nih.gov). The genus *Euphorbia* is the largest in the spurge family, including more than 2000 species [2].

Tree subspecies belonging to the *E. officinarum* group are *E. officinarum* L., *E. officinarum* subs. Echinus, the spurge (*Euphorbia officinarum* L. subsp. Echinus (Hooker fil. and Cosson)) is a species with very fleshy stems, cactiform [11], and finally *E. officinarum* subs. Echinus f. Cristata [22].

#### 3.2.3. Geographic Distribution

Mauritania, Western Sahara, Morocco, and Algeria are the origin and the habitat of *E. officinarum* L. and *E. officinarum* subs. Echinus in the southwest of Morocco from coast to Anti-Atlas Mountains cactiform; it can, therefore, be considered endemic to Morocco [11]. There are two Moroccan endemic spurges: the general area of the urchin spurge borders the Atlantic Ocean and extends from the south of the Oued Souss to Cape Barbas. In addition, it constitutes, shortly after the branch towards Tassila, a large settlement that extends on either side of the main Agadir-Tiznit road [23]. The terrain decreases in altitude to 800 m; the tip of the Anti-Atlas offers less steep relief. The originality of the sector, therefore, comes from thermophilic vegetation made up of succulent species with *E. officinarum* Hook. f. and Coss. (subsp. *Officinarum* and subsp. *Echinus)* adapted to this environment [24]. The appearance of the cactoid spurge (*E. officinarum* Hook. f. and Coss.) extends along the plains of the Draa over 200 km in length. For a time, in the most northerly zone of the Southern Anti-Atlas zone, one can find on the same ground euphorbia, *Senecio anteuphorbium* L. and argan trees [24]. Beyond, by approaching the borders of Tan Tan in the middle of the land, the Argan tree decays to make way for monovegetation of spurge and cactoids. The subspecies of this spurge are particularly difficult to distinguish and hybridize perfectly with each other [25]. It spreads in the northern and western parts of the Anti-Atlas. The species is very abundant. Its area substantially covers that of the Argan tree with which it is generally associated. However, it climbs higher than its altitude and then reaches 1600 m. It is found on the various substrate but escapes as Argan trees from clayey soils (areas of spreading wadis and basins) [23].

### 3.3. Local and Conventional Medicinal Uses

*Euphorbia officinarum* is used in traditional medicine to treat skin diseases and ophthalmological diseases. Nevertheless, the plant extract should be used in low concentrations due to its high toxicity [6,25]. The main interest of these large areas of *Euphorbia* for beekeepers is to produce pure Tikiout (tachelhit) or Daghmus (Arabic) honey. These succulents have a large number of tiny flowers at the top of each spiny appendage. Filled with nectar, these flowers provide enough material to make honey known for its therapeutic properties [25]. The honey is used for the sterility, intestinal gas, eczema, psoriasis, skin diseases, antiseptic, against cancer, asthma, ulcer, hot water burns, chickenpox “Cold” bowel, and throat [25]. On the other hand, it is recommended for pyelonephritis and cystitis to use the powder of *E. officinarum*, mixed with honey [26].

### 3.4. Isolation and Identification of Phytoconstituents

The chemical study of the latex of the Moroccan endemic plant *E. officinarum* L. has been used vastly as a source for new different chemical compounds such as terpenes, diterpenes, and steroids (Table 1), with the aim of treating various conditions.

In 1985, for the first time, Ben Harref and Lavergne [16] used latex for isolation of nine compounds with triterpenic and steroidal skeleton (1–9): lupeol (1), lupeol acetate (2), lanostenol (3), lanosterol (4), 24-methylene lanostenol (5), 4*α*,14*α*-dimethyl-24-methylen-5 *a* -cholest-8-en-3*β*-ol (6), 4 *α*, 14 *α*,24(R)-trimethyl-5 *a* -cholesta-8,25(27)-dien-3 *ß*-ol (7), 4 *α*, 14 *a* -dimethyl-5 *a* -cholesta-8,24-dien-3 *ß-*ol (8), and 4 *α*, 14 *a* -dimethyl-5 *a* -cholest-8-en-3 *ß-*ol (9). Again, the examination of the latex of *E. officinarum* collected in May 1999 from plants of the North Atlantic coast of Agadir led to the isolation of two new steroids: 3 *β*,7 *a* -dihydroxy-4 *α*, 14 *a* -dimethyl-5 *a* -cholest-8-en-11-one (10), and 3 *β*,7 *ß* -dihydroxy-4 *α*, and 14 *a* -dimethyl-5 *a* -ergost-8-en-11-one (11) [17]. Likewise, the study of Mazoir et al. [18] identified two new terpenes: obtusifoliol (12) and 31-norlanostenol 2 (13).

Moreover, three new highly functionalized ingol diterpenes have been isolated from *E. officinarum* latex: ingol 7,8,12-triacetate 3-phenylacetate (14), ingol 7,8,12-triacetate 3-(4-methoxyphenyl)acetate (15), and 8-methoxyingol 7,12-diacetate 3-phenylacetate (16), together with the novel spirotriterpene,3S,4S,5R,7S,9R,14R-3,7-dihydroxy-4,14-dimethyl-7[8->9]-Abeo-cholestan-8-one (17) [19].

### 3.5. Phytochemical and Biological Activities of Bioactive Derivatives

The genus *Euphorbia* has been the subject of abundant phytochemical and pharmacological investigations, due to its potential medical applications. This plant is characterized by the presence of latex, which is widely used as major compounds for the semisynthetic of new bioactive compounds that has been found to exhibit a spectrum of biological activities such as antibacterial, antifungal, and antiparasitic effects (Table 2).

#### 3.5.1. Antimicrobial Activity

In some preliminary studies, it was reported that the antimicrobial activity of major natural compounds from *E. officinarum* latex such as lupeol acetate and 31-norlanostenol did not show any noticeable activity. Nonetheless, the study of Smaili et al. [10] evaluated the antimicrobial activity of two semisynthetic bioactive triterpene derivatives from *E. officinarum* latex against fungal and bacterial phytopathogens, which are responsible for several diseases.

These derivative compounds were obtained by chemical modifications using the oxidation of lupeol acetate using ruthenium trichloride and sodium periodate yielded 3*β*-acetoxy-norlup-20-one (18); the treatment of 31-norlanostenol using tosyl chloride, on the other hand, resulted in 3-chloro-4*α*,14*α*-dimethyl-5*α*-cholest-8-ene (19). The antifungal activity was tested by evaluating the effect of these derivatives through the three phases of the life cycle (mycelial growth, conidia production, and conidia germination) of 6 strains of *V. dahlia (*SH, SE, SJ, SA, SB, and E4)*, Penicillium expansum*, and *Fusarium oxysporum* fsp. *melonis*. *V. dahliae* and *F. oxysporum,* which are the causal agent of several crops and economic damage. The results showed that although mycelial growth of several strains of *Verticillium dahlia* (six strains of *V. dahliae* and *Fusarium oxysporum* ssp. *melonis* and *P. expansum*) were affected only moderately of all the strains were achieved and only at elevated concentrations (100 and 200 *μ*g mL), whereas the tested compounds 18 and 19 reduced significantly conidia formation when reduction ranged from 39 to 54% for the strain SA, from 48 to 63% for the strain SB, from 57 to 69% for SH, and from 55 to 67% for E4. However, reductions were very much higher for the strains SE and SJ of *V. dahliae* as well as for *Penicillium expansum* and *Fusarium oxysporum* fsp. *melonis* since they oscillated between 70 and 96%.

Using the disc diffusion method, the antibacterial activity was tested against *Pseudomonas syringae* pv. *syringae* (Pss), *P. syringae* pv. *tabacci* (Pst), *Erwinia amylovora* (Ea), and *Agrobacterium tumefaciens* (At). As a result, compound 19 was more effective, while the growth of *P. syringae* pv. *syringae* was highly inhibited by compound 19 (16 mm of inhibition) conversely to compound 18 showed moderate inhibition. Comparatively, moderate growth inhibitions were obtained for *P. syringae* pv. *tabaci* with both of them (6 mm of inhibition). In addition, compound 19 was more effective in inhibiting the growth of *Erwinia amylovora* than compound 18. As can be seen, the semisynthetic triterpenes derived from *E. officinarum* latex act as fungistatic and antibactericide compounds. As reported by Smaili et al. [10] and Abboud et al. [27], these triterpenes influence the permeability of bacterial membrane by inserting themselves into the lipid bilayer of the cell membrane. Indeed, pentacyclic triterpenes are more hydrophobic than tetracyclic ones which allowed the deep incorporation in the lipid bilayer. These semisynthetic triterpenes derived from *E. officinarum* latex contain remarkable antimicrobial agents that may be drug candidates against phytopathogen microorganism diseases.

#### 3.5.2. Antifeedant and Toxic Activities

The antifeedant activity of obtusifoliol and 31-norlanostenol, main compounds of *E. officinarum* latex and their derivatives, was investigated [20]. Indeed, the chemical modifications of obtusifoliol, 31-norlanostenol natural triterpenes yielded 10 semisynthetic terpenoid compounds (20–29) and their antifeedant activities were tested on several insect species (*Spodoptera littoralis*, *Myzus persicae*, and *Rhopalosiphum padi*), as well as their selective cytotoxicity on insect Sf9 and mammalian CHO cells. The fundings showed that several of the test compounds were moderate antifeedants in choice tests compared with the positive controls when tested at an initial dose of 50 *µ*g/cm^2^. The percentage of feeding or settling inhibition (%FI or %SI) was % SI = 98 for polygodial on *M. persicae* and *R. padi* and % FI = 100 for ryanodine on *S. littoralis* at the same dose. Specifically, 31-norlanostenol (2), and the compounds 28, 21, 25, were active to *M. persicae* (% SI between 73 ± 10 and 67 ± 9, *p* < 0.05 Wilcoxon's paired test). The compounds 2 and 29 were active on *R. padi* (% SI between 70 ± 12 and 65 ± 10; *p* < 0.05 Wilcoxon's paired test) and the compound 23 revealed moderate-low antifeedant effects to *S. littoralis* (% FI of 55 ± 10 and 52 ± 9, resp., *p* < 0.05 Wilcoxon's paired test). Therefore, these effects did not merit further dose-response experiments (% FI/SI < 75). Bioactive compounds of our plant exhibited a strong postinfective toxicants, with 29, 23 being less potent. Overall, Sf9 cells were more sensitive to the active compounds than CHO, while the compounds 21, 22, 25, 23 with 22 and 25 were selective to this cell line. Notably, compound 21 had the strongest cytotoxicity to mammalian CHO.

To sum up, a series of semisynthetic triterpene derivatives of obtusifoliol and 31-norlanostenol have been found and a few of the test compounds (18–20%) acted as antifeedants on *M. persicae* and *R. padi*, and a larger number of the test substances (80%) had postingestive effects on *S. littoralis,* affecting insect growth.

#### 3.5.3. Antiparasitic Activity

The antiparasitic effects were tested *via* evaluating the leishmanicidal and trypanocidal activities. The chemical modifications of the major component of *E. officinarum* (obtusifoliol and 31-norlanosterol) allowed the obtention of 13 semisynthetic terpenoid derivatives and then the evaluation of their leishmanicidal and trypanocidal activities on *L. infantum* and *T. cruzi,* respectively [8].

The findings of Mazoir et al. [8] showed that 72% of the test compounds had important leishmanicidal activity against *L. infantum.* However, the strongest leishmanicidal compounds were 38, 34, 35, 41 (<8 *μ*g/mL) > 36, 37, and 39 (<15 *μ*g/mL).

Furthermore, 36% of the tested compounds revealed a trypanocidal activity against *T. cruzi*. Overall *T. cruzi* was less sensitive to these compounds, with 35, 37, and 38. On the other hand, the results of cytotoxicity assays indicated that the mammalian CHO cells were moderately affected by 44% of tested compounds.

Compounds from *E. officinarum* exhibit a strong antiparasitic effects. Indeed, as shown, the compounds 34, 36, 39, and 41 showed significant selective leishmanicidal activity with a low or moderate nonspecific cytotoxicity, and the compound 37 presented a significant activity against both parasites (*L. infantum* and *T. cruzi*), without nonspecific cytotoxicity associated.

The study of Bailen et al. [7] is a continuation of the investigated antifeedant, antiparasitic, and toxic effects. Fifteen additional semisynthetic terpenoid derivatives from 31-norlanostenol and obtusifoliol were tested against the insect *Spodoptera littoralis* and their antiparasitic effects against two protozoa causing disease in humans, *T. cruzi* and *L. infantum*, and the selective cytotoxicity of these fifteen compounds on insect Sf9 and mammalian CHO cells, while the results showed that 40% of the test substances were postingestive toxicants to *S. littoralis*, 87% of the test compounds had antiparasitic effects on both *L. infantum* and *T. cruzi*, with some of them being selective parasite toxicants and 47% of compounds were affected mammalian CHO cells. Compounds 15 and 2 had the strongest cytotoxic effects, followed by 47 > 52 > 58, 50, 54, 55, 48 > 53.

As reported by Mazoir et al. [18], the antiparasitic mechanisms of terpenes are not clear [28–31]. The authors suggested that the action might (i) impact the synthesis of proteins and (ii) nucleic acids by their inhibition, (iii) with the inhibition of a membrane-associated calcium-dependent ATPase pump, (vi) or by the action of the triterpenes on endogenous sterol metabolism of *T. cruzi* and *Leishmania* parasites, which have a strict requirement for survival and growth of these parasites; the last suggestion was the most convincing according to Mazoir et al. [18].

In summary, the high antileishmanial, trypanocidal, and the toxicity activities indicate an interesting role of these compounds, which can be used to discover new drugs with high activity.

### 3.6. Plant Growth Promoters and Inducers of Disease Resistance

Different organic molecules such as polyamines, phenols, steroids, and terpenoid compounds play a crucial role in plant development as plant growth regulators. Indeed, they can activate cell division, root and shoot growth, and the germination and act also as a chemical defense. Some secondary metabolites of *E. officinarum* showed their potential against phytopathogens. The triterpene derivatives isolated from the latex of *E. officinarum* enhance resistance against wilt disease at lower concentrations [21].

The study of Smaili et al. [32] which used an approach based on seed treatment showed the capacity of this plant to enhance the resistance of *Nicotiana benthamiana* against wildfire disease caused by *Pseudomonas syringae* pv. *tabaci*. Molecular investigations revealed that the compounds 18 and 19 reduced disease severity, which was correlated with a reduction of bacterial populations in the plant. Analysis of plant defense markers revealed that H_2_O_2_ and guaiacol peroxidase were only slightly activated but were primed after pathogen infiltration. However, polyphenol oxidase, catalase, and ascorbate peroxidase were directly induced by the triterpenic derivatives [32].

In addition, Smaili et al. [21] evaluated, *in vitro*, the ability of triterpenes derivatives to protect tomato seedlings against *V. dahliae* in the greenhouse. In this study, tomato seedlings derived from seeds that germinated in the presence of 10 or 50 mg/mL of 2 semisynthesized compounds (59–60) yielded of the oxidation of 31-norlanostenol were root-inoculated with *V. dahliae*. However, the finding of this work showed, at low concentrations, an important reduction of disease severity (10 and 50 mg/mL). Indeed, disease assessment was carried out based on leaf alteration index, stunting index and browning index; as a result, the reduction of leaf alteration index and of stunting index ranged from 52 to 68% and from 43 to 67%, respectively, and vessel discoloration was reduced by at least 95%. On the other hand, the compounds were also able to elicit H_2_O_2_ accumulation before and after fungal inoculation and also increase peroxidase and polyphenol oxidase activities [21].

Moreover, Smaili et al. [33] evaluated two new semisynthetic derivatives from *E. officinarum* (61–62) on the growth of tomato seedlings under stress of the pathogens *Verticillium dahliae* and *Agrobacterium tumefaciens*. The use of the foliar spray of derivatives 61 and 62 **e**nhanced disease resistance against *V. dahliae* and *A. tumefaciens*. In addition, their application significantly improved growth rate, fresh weight, dry weight, and leaf area. Moreover, they boosted the photosynthetic pigments, proline content, and nitrate reductase activity [33].

Furthermore, the findings showed also that these derivatives induce H_2_O_2_ accumulation and increase the activity of several antioxidant defense enzymes including catalase, ascorbate peroxidase, and guaiacol peroxidase. These results suggest that the semisynthetic triterpenes from *E. officinarum* represent new plant growth regulators and inducers of plant disease resistance and suggest their use as plant defense inducers in crop protection.

## 4. Concluding Remarks and Perspectives

*E. officinarum* is an endemic Moroccan medicinal plant used mainly in traditional medicine. This plant possesses various chemical diversity including polyphenols, flavonoids, and alkaloids. Moreover, several other active compounds were synthesized chemically from this species. On the other hand, the biological exploration of *E. officinarum* showed that this plant exhibit antibacterial and antifungal, cytotoxic, and antifeedant properties. Indeed, extracts from *E. officinarum* inhibited importantly the growth of several bacterial strains, which could constitute an important source as antibacterial drugs. In fact, the isolation of *E. officinarum* compounds and/or the use of those synthesized can remarkably identify bioactive substances, exhibiting interested antibacterial effects. They could also decipher molecular mechanisms by which these molecules exerted their antibacterial action. Moreover, further investigations regarding molecular insights of antifungal and antiparasitic activities of *E. officinarum* and their bioactive compounds are also needed and could importantly show promising results. The cytotoxic effect of *E. officinarum* showed also remarkable findings, but these activities were carried out *in vitro* on the cellular model and could not justify the use of plant and/or their derivatives as anticancer drugs. Furthermore, the evaluation of anticancer effects using in vivo system and clinical tests could importantly identify some molecules using specifically in chemotherapy. Although this species is rich in bioactive compounds, this plant or its derivatives compounds have not been yet evaluated. This is the case of antiviral, antidiabetic, anticancer, antioxidant, and anti-inflammatory properties. The exploration of *E. officinarum* and their bioactive compounds in further investigations by Moroccan and/or other research groups are required. In addition, a lack of data on *in vivo* studies was observed. It will be interesting to investigate the *in vivo* biological activities of this species and its derivatives on animal models. Thus, toxicological evidence should also be evaluated for checking the security of this medicinal plant before its use in clinical tests. Therefore, several calls for papers should be addressed to carry out all cited perspectives and remarks. In fact, Moroccan researches are often limited by the financial support, and in this case of an endemic Moroccan medicinal plant, the establishment of collaboration between other research groups has a great interest.

## Figures and Tables

**Table 1:** Phytoconstituents from *E. officinarum* latex.

No./name	Extraction solvent	Isolated compounds	Structure	References
1. Lupeol	Methanolic extract	Triterpenic (a) and steroidal skeleton (b)	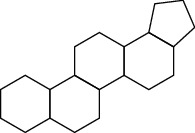 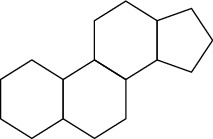	[16]
2. Lupeol acetate				
3. Lanostenol				
4. Lanosterol				
5. 24-methylene lanostenol				
6. 4*α*,14*α*-dimethyl-24-methylen-5 *a* -cholest-8-en-3*β*-ol				
7. 4 *α*, 14 *α*,24(R)-trimethyl-5 *a* -cholesta-8,25(27)-dien-3 *ß*-ol				
8. 4 *α*, 14 *a* -dimethyl-5 *a* -cholesta-8,24-dien-3 *ß* -ol				
9. 4 *α*, 14 *a* -dimethyl-5 *a* -cholest-8-en-3 *ß* -ol				

10. 3 *β*,7 *a* -Dihydroxy-4 *α*, 14 *a* -dimethyl-5 *a* -cholest-8-en-11-one	Methanolic extract	Steroids	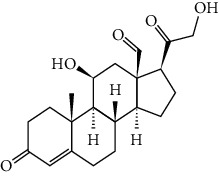	[17]
11. 3 *β*,7 *ß* -Dihydroxy-4 *α*, 14 *a* -dimethyl-5 *a* -ergost-8-en-11-one				

12. Obtusifoliol (4*α*,14*α*-diméthyl-5*α*-ergosta-8,24-dièn-3*β*-ol)	Methanolic extract	Triterpene	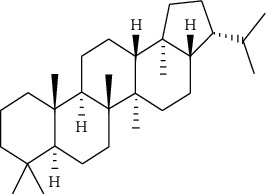	[18]
13. 31-Norlanostenol (4*α*,14*α*-diméthyl-5*α*-cholesta-8-én-3*β*-ol)				

14. Ingol 7,8,12-triacetate 3-phenylacetate	Methanolic extract	Ingol skeleton	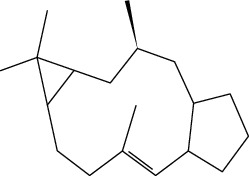	[19]
15. Ingol 7,8,12-triacetate 3-(4-methoxyphenyl)acetate				
16. 8-Methoxyingol 7,12-diacetate 3-phenylacetate				

17. 3S,4S,5R,7S,9R,14R-3,7-dihydroxy-4,14-dimethyl-7[8->9]-Abeo-cholestan-8-one		Spirotriterpene	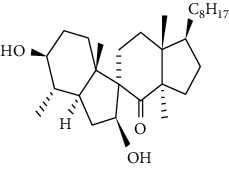	[19]

**Table 2:** Semisynthetic of new bioactive compounds from *E. officinarum* latex.

No. name	Tested activity	References
18. 3*β*-Acetoxy-norlup-20-one (1) 19. 3-Chloro-4*α*,14*α*-dimethyl-5*α*-cholest-8-ene (2)	*Antibacterial activity*	[10]
	(i) *Pseudomonas syringae* pv. *syringae* (pss)	
	(ii) *P. syringae* pv. *tabacci* (pst)	
	(iii) *Erwinia amylovora* (Ea)	
	(iv) *Agrobacterium tumefaciens* (At)	
	*Antifungal activity*	
	(i) *V. dahliae (*SH, SE, SJ, SA, SB, and E4)	
	(ii) *Penicillium expansum*	
	(iii) *Fusarium oxysporum* fsp. *melonis*. *V. dahliae*	
	(iv) *F. oxysporum*	

20. 3b-Tosyloxy-4a,14a-dimethyl-5a-ergost-8-en-24-one (C36H54O4S) (1)	*Antifeedant activity* (i) *Spodoptera littoralis* (ii) *Myzus persicae* (iii) *Rhopalosiphum padi*	[20]
21. 4a,14a-dimethyl-5a-ergost-8-en-3,24-dione (C30H48O2) 6		
22. 4a,14a-Dimethyl-5a-ergosta-8,24-dien-3-one(C30H48O) (7)		
23. 4a,14a-Dimethyl-5a-cholest-8-ene-3,11-dione-7-thiadiazoline (C34H51O4N3S) (9)		
24. 4a,14a-dimethyl-5acholest-8-ene-7,11-dione-3-thiadiazoline (C34H51O4N3S) (11)		
25. 4a,14a-Dimethyl-5a-cholest-8-ene-3,11-dione-7 thiosemicarbazone (C30H45O2N3S) (8)		
26. 4a,14a-Dimethyl-5a-cholesta-7,9-diene-3-thiosemicarbazone(C30H49N3S) (10)		
27. 3b-Tosyloxy-4a,14a-dimethyl-5a-cholest-8-ene(C36H56O3S) (3)		
28. 3b-Tosyloxy-4a,14a-dimethyl-5acholest-8-ene-7,11-dione (C36H52O5S( (4(		
29. 3b-Acetoxy-4a,14a-dimethyl-5a-cholest-8-ene-7,11-dione (C31H48O4) (5)		

30. 1 3*β*-Tosyloxy-4*α*,14*α*-dimethyl-5*α*-ergosta-8-en-24-one (C36H54O4S)	*Leishmanicidal activity*	[8]
31. 2 31-Norlanostenol (C29H50O)	(i) *L. infantum*	
32. 3 3*β*-Tosyloxy-4*α*,14*α*-dimethyl-5*α*-cholest-8-ene (C36H56O3S)	*Trypanocidal activity*	
33. 4 3*β*-Tosyloxy-4*α*,14*α*-dimethyl-5*α*-cholest-8-ene-7,11-dione (C36H52O5S)	(i) *T. cruzi*	
34. 5 3*β*-Acetoxy-4*α*,14*α*-dimethyl-5*α*-cholest-8-ene-7,11-dione (C31H48O4)	*Cytotoxicity test*	
35. 6 4*α*,14*α*-Dimethyl-5*α*-ergosta-8-ene-3,24-dione (C30H48O2)	(i) Mammalian CHO cells	
36. 7 4*α*,14*α*-Dimethyl-5*α*-ergosta-8,24-dien-3-one (C30H48O)		
37. 8 4*α*,14*α*-Dimethyl-5*α*-cholest-8-ene-3,7,11-trione-7-thiosemicarbazone (C30H43O2N3S)		
38. 9 4*α*,14*α*-Dimethyl-5*α*-cholest-8-ene-3,7,11-trione-7-thiadiazoline (C34H51O4N3S)		
39. 10 4*α*,14*α*-Dimethyl-5*α*-cholesta-7,9-dien-3-one thiosemicarbazone (C30H49N3S)		
40. 11 3*β*-Acetoxy-norlup-20-one (C31H50O3)		
41. 12 3*β*-Hydroxy-norlup-20-one (C29H48O2)		
42. 13 4*α*,14*α*-Dimethyl-5*α*-cholest-8-ene-3,7,11-trione-3-thiadiazoline (C34H51O4N3S)		

43. 8*α*,9*α*-Epoxy-4*α*,14*α*-dimethyl-5*α*-cholest-3*β*-ol 2	*Antifeedant activity*	[7]
44. 4*α*,14*α*-Dimethyle-5*α*-cholesta-7,9-dien-3*β*-ol (3) 3	(i) *Spodoptera littoralis*	
45. 3-Chloro-4*α*,14*α*-dimethyl-5*α*-cholest-8-en-7-one 4	*Antiparasitic activity*	
46. 4*α*,14*α*-Dimethyl-5*α*-cholestat-8-en-3-one (C29H48O) 5	(i) *Trypanosoma cruzi*	
47. 2-Carbomethoxy-4*α*,14*α*-dimethyl-5*α*-cholesta-2,8-dien-3-ol 6	(ii) *Leishmania infantum*	
48. 8*α*,9*α*-Epoxy-4*α*,14*α*-dimethyl-5*α*-cholest-3-one 7	*Cytotoxicity test*	
49. 4*α*,14*α*-Dimethyl-5*α*-cholest-8-ene-3,7-dione 8	(i) Mammalian CHO cells	
50. 4*α*,14*α*-Dimethyl-5*α*-cholest-8-en-3-one 9	(ii) Insect Sf9	
51. 4*α*,14*α*-Dimethyl-7-oxo-5*α*-cholest-8-ene-3,4-lactone 10		
52. 4*α*,14*α*-Dimethyl-7,11-dioxo-5*α*-cholest-8-ene-3,4-lactone 11		
53. 8*α*,9*α*-Epoxy-4*α*,14*α*-dimethyl-5*α*-cholesta-3,4-lactone 12		
54. 4*α*,14*α*-Dimethyl-5*α*-cholesta-7,9-diene-3,4-lactone 13		
55. 4*α*,14*α*-Dimethyl-3,4-seco-5*α*-cholesta-7,9-diene-3,4-diol 14		
56. 3-Carbomethoxy-4-hydroxy-4*α*,14*α*-dimethyl-3,4-seco-5*α*-cholesta-7,9-diene 15		
57. 8*α*,9*α*,24,28-Diepoxy-4*α*,14*α*-dimethyl-5*α*-ergost-3*β*-ol 17		
58. 8*α*,9*α*,24,28-Diepoxy-4*α*,14*α*-dimethyl-5*α*-ergosta-3,4-lactone 18		

59. 3b-Tosyloxy-4a,14adimethyl-	Plant growth promoters activity	[21]
5a-cholesta-7,9-diene F1		
60. 4a,14a-dimethyl-5a-cholesta-7,9-dien-3b-ol F2		
61. 3*β*-Acetoxynorlup-		[21]
20-one (F4)		
62. 3-Chloro-4*α*,14*α*-dimethyl-5*α*-cholest-		
8-ene (F6)		

63. 3*β*-Acetoxy-norlup-20-one		[21]
64. 3-Chloro-4*α*,14*α*-dimethyl-5*α*-cholest-8-ene		

## Data Availability

Some data used in this study are indicated in Supplementary Materials part.

## Supplementary Materials

Supplementary Materials This part contains PRISMA checklist references; all used links to papers cited and highlighted in this manuscript; and also links of other papers that were excluded.
