# Supplementary material for: Conventional Medicinal Uses, Phytoconstituents, and Biological Activities of Euphorbia officinarum L.: A Systematic Review
Source: Adv Pharmacol Pharm Sci. 2022 Jan 6;2022:9971085. doi: 10.1155/2022/9971085 (PMC8758299; doi:10.1155/2022/9971085)
Supplement: Supplementary Materials — This part contains PRISMA checklist references; all used links to papers cited and highlighted in this manuscript; and also links of other papers that were excluded. [file 9971085.f1.docx]

**Database research strategy**

**Google scholar**

Daoubi, M., Benharref, A., Hernandez-Galan, R., Macías-Sánchez, A. J., & Collado*, I. G. (2004). Two novel steroids from Euphorbia officinarum latex. *Natural product research*, *18*(2), 177-181.

Smaili, A., Mazoir, N., Rifai, L. A., Koussa, T., Makroum, K., Benharref, A., ... & Faize, M. (2017). Antimicrobial activity of two semisynthetic triterpene derivatives from Euphorbia officinarum latex against fungal and bacterial phytopathogens. Natural product communications, 12(3), 1934578X1701200305.

Daoubi, M., Marquez, N., Mazoir, N., Benharref, A., Hernández-Galán, R., Munoz, E., & Collado, I. G. (2007). Isolation of new phenylacetylingol derivatives that reactivate HIV-1 latency and a novel spirotriterpenoid from Euphorbia officinarum latex. *Bioorganic & medicinal chemistry*, *15*(13), 4577-4584.

Smaili, A., Rifai, L. A., Mazoir, N., Koussa, T., Faize, L., Alburquerque, N., ... & Faize, M. (2019). Semisynthetic Triterpenes Derived from Euphorbia officinarum as Plant Growth Promoters and Inducers of Disease Resistance. *Journal of plant growth regulation*, *38*(1), 262-272.

Terrab, A., Marconi, A., Bettar, I., Msanda, F., & Díez, M. J. (2014). Palynological characterisation of Euphorbia honeys from Morocco. Palynology, 38(1), 138-146.

Bettar, I., González-Miret, M. L., Hernanz, D., Marconi, A., Heredia, F. J., & Terrab, A. (2019). Characterisation of Moroccan Spurge (Euphorbia) honeys by their physicochemical characteristics, mineral contents and colour. *Arabian Journal of Chemistry*, *12*(8), 2052-2060.

Mazoir, N., & Benharref, A. Hemisynthesis of new thiosemicarbazone derivatives resulting from latex of Moroccan endemic plant: Euphorbia officinarum.

Mazoir, N., Benharref, A., Bailén, M., Reina, M., & González-Coloma, A. (2008). Bioactive triterpene derivatives from latex of two Euphorbia species. Phytochemistry, 69(6), 1328-1338.

Mazoir, N., Benharref, A., Bailén, M., Reina, M., González-Coloma, A., & Martínez-Díaz, R. A. (2011). Antileishmanial and antitrypanosomal activity of triterpene derivatives from latex of two Euphorbia species. Zeitschrift für Naturforschung C, 66(7-8), 360-366.

Mazoir, N., Liazid, A., Auhmani, A., Daoubi, M., Dakir, M., Benharref, A., ... & Pierrot, M. (2005). D’EUPHORBIA OFFICINARUM.

Bailen, M., Khamlichi, M. D., Benharref, A., Martinez-Diaz, R. A., & Gonzalez-Coloma, A. (2016). New bioactive semisynthetic derivatives of 31-norlanostenol and obtusifoliol from Euphorbia officinarum. Natural product communications, 11(6), 1934578X1601100609.

Wang, S. Y., Huang, C., Sun, R. K., Lu, L. N., Liang, H. G., Gao, L., ... & Yang, B. F. (2019). New tirucallane triterpenoids from the dried latex of Euphorbia resinifera. Phytochemistry Letters, 29, 220-224.

Wang, S. Y., Huang, C., Sun, R. K., Lu, L. N., Liang, H. G., Gao, L., ... & Yang, B. F. (2019). New tirucallane triterpenoids from the dried latex of Euphorbia resinifera. Phytochemistry Letters, 29, 220-224.

Avila, L., Perez, M., Sanchez-Duffhues, G., Hernández-Galán, R., Muñoz, E., Cabezas, F., ... & Echeverri, F. (2010). Effects of diterpenes from latex of Euphorbia lactea and Euphorbia laurifolia on human immunodeficiency virus type 1 reactivation. Phytochemistry, 71(2-3), 243-248.

Smaili, A., Mazoir, N., Rifai, L. A., Koussa, T., Makroum, K., Belfaiza, M., ... & Faize, M. (2018). Induced resistance to wild fire disease of Nicotiana benthamiana using seed treated with triterpene derivatives from Euphorbia. *Journal of Plant Pathology*, *100*(1), 75-83.

Mazoir, N., Benharref, A., Vaca, L., Reina, M., & González‐Coloma, A. (2020). Optimization of Insecticidal Triterpene Derivatives by Biomimetic Oxidations with Hydrogen Peroxide and Iodosobenzene Catalyzed by MnIII and FeIII Porphyrin Complexes. *Chemistry & Biodiversity*, *17*(9), e2000287.

Fischer, E., Lobin, W., & Mutke, J. (2011). Striga barthlottii (Orobanchaceae), a new parasitic species from Morocco. Willdenowia, 41(1), 51-56.

Idm’hand, E., Msanda, F., & Cherifi, K. (2020). Ethnopharmacological review of medicinal plants used to manage diabetes in Morocco. *Clinical Phytoscience*, *6*(1), 1-32.

Barkaoui, M., Katiri, A., Boubaker, H., & Msanda, F. (2017). Ethnobotanical survey of medicinal plants used in the traditional treatment of diabetes in Chtouka Ait Baha and Tiznit (Western Anti-Atlas), Morocco. *Journal of ethnopharmacology*, *198*, 338-350.

Barkaoui, M., Katiri, A., Boubaker, H., & Msanda, F. (2017). Ethnobotanical survey of medicinal plants used in the traditional treatment of diabetes in Chtouka Ait Baha and Tiznit (Western Anti-Atlas), Morocco. *Journal of ethnopharmacology*, *198*, 338-350.

Mazoir, N., Belahyan, A., & Benharref, A. New 1, 3, 4-Thiadiazolines Hemisynthesized from Moroccan Endemic Plants: Euphorbia officinarum latex.

Carter, S. (2005). Euphorbias of southern Morocco. *Cactus and Succulent Journal*, *77*(1), 34-37.

Mazoir, N., Auhmani, A., Daoubi, M., Collado, I. G., & Benharref, A. (2007). Hemisynthesis of New Triterpene Derivatives using Oxidation by CrO3 and NaIO4‐(RuCl3, 3H2O). *Synthetic communications*, *37*(8), 1289-1299.

Mazoir, N., Giorgi, M., & Benharref, A. (2005). (4S, 5S, 10S, 13R, 14R, 17R)-8α, 9α-Epoxy-4α, 14α-dimethyl-5α-cholestan-3-one. *Acta Crystallographica Section E: Structure Reports Online*, *61*(11), o3709-o3711.

Auhmani, A., Giorgi, M., & Mazoir, N. (2005). (3S, 4S, 5S, 10S, 13R, 14R, 17R)-4α, 14α-Dimethyl-3β-tosyl-5α-cholest-8-ene-7, 11-dione. *Acta Crystallographica Section E: Structure Reports Online*, *61*(4), o1190-o1192.

Thiede, J. The genus Striga (Orobanchaceae)–a root parasite on succulent euphorbias.

Farah, H., Ech-chahad, A., & Lamiri, A. (2014). Semi-synthesis and Antimicrobial activities of some new Euphorbioside derivatives. *Int. J. Chem. Tech. Res.*, *6*, 763-767.

Idm'hand, E., Msanda, F., & Cherifi, K. (2020). Ethnobotanical study and biodiversity of medicinal plants used in the Tarfaya Province, Morocco. *Acta Ecologica Sinica*, *40*(2), 134-144.

Lavergne, J. P. (1985). Triterpenes from the latex of Moroccan euphorbia: Euphorbia resinifera, Euphorbia echinus and Euphorbia officinarum. Isolation and comparative 13C NMR study of the 4 tetracyclic triterpene classes: eupho-lanostane, elemo-lanostane, lanostane and 31-nor lanostane. *Bulletin de la Societe Chimique de France*.

Mazoir, N., Itto, M. Y. A., Artiles, M. R., & Benharref, A. (2006). 4α, 14α, Dimethyl-5α-cholest-8-en-3-one thiosemicarbazone. *Molbank*, *2006*(6), M504.

KUMAR, M. B., & BALAKRISHNAN, N. (1997). ETHNOBOTANICAL STUDIES OF THE GENUS EUPHORBIA L. *Biotechnological Approaches in Soil Microorganisms for Sustainable Crop Production*, 46.

KUMAR, M. B., & BALAKRISHNAN, N. (1997). ETHNOBOTANICAL STUDIES OF THE GENUS EUPHORBIA L. *Biotechnological Approaches in Soil Microorganisms for Sustainable Crop Production*, 46.

Crous, P. W., Wingfield, M. J., Guarro, J., Hernández-Restrepo, M., Sutton, D. A., Acharya, K., ... & Groenewald, J. Z. (2015). Fungal Planet description sheets: 320–370. *Persoonia: Molecular Phylogeny and Evolution of Fungi*, *34*, 167.

Wingfield, M. J., Guarro, J., & Madrid, H. (2015). Fungal Planet description sheets: 320-370.

Attalla, S. I. (1999). Weed flora distribution in sugar cane Saccharum officinarum L. fields at Esna, Quena. *Bulletin of Faculty of Agriculture, University of Cairo*, *50*(1), 33-40.

Mauseth, J. D. (2004). The structure of photosynthetic succulent stems in plants other than cacti. International Journal of Plant Sciences, 165(1), 1-9.

Bensusan, K., Gdaniec, A., Guillem, R., & Taheri, A. (2015). Chasing Atlas Dragons. *Cactus and Succulent Journal*, *87*(3), 136-144.

BENSUSAN, K., GDANIEC, A., GUILLEM, R., & TAHERI, A. Atlas Dragons.

Lopez-Rodriguez, M., Mazoir, N., Daoubi, M., Reina, M., & Benharref, A. (2007). 1-(1, 5-Dimethylhexyl)-3a, 5b, 12a, 14a-tetramethyl-2, 3, 3a, 4, 5, 5a, 5b, 11, 12, 13, 14, 14a-dodecahydro-1H, 12aH-cyclopenta [1, 2] phenanthro [7, 8-b] indole. *Acta Crystallographica Section E: Structure Reports Online*, *63*(12), o4911-o4911.

Zeroual, A., Mazoir, N., Benharref, A., & El Hajbi, A. (2015). Understanding of the stereoselective epoxidation on triterpene derivative using transition state theory. *Journal of Computational Methods in Molecular Design*, *5*(4), 158-161.

Peltier, J. P., & Msanda, F. (1995). Diversité et originalité de la steppe à Euphorbia officinarum L. subsp. echinus (HOOKER fil. et COSSON) VINDT du sud‐ouest Marocain. *Feddes Repertorium*, *106*(3‐4), 215-229.

Lamb, B. M., Bensusan, K., Amezian, M., & Wagner, D. R. (2009). Moroccan Asclepiads following autumn storms. *Cactus and Succulent Journal*, *81*(5), 240-255.

Connolly, J. D., & Hill, R. A. (2007). Triterpenoids. *Natural Product Reports*, *24*(2), 465-486.

Msanda, F., El Aboudi, A., & Peltier, J. P. (2002). Originalité de la flore et de la végétation de l'Anti‐Atlas sud‐occidental (Maroc). *Feddes Repertorium: Zeitschrift für botanische Taxonomie und Geobotanik*, *113*(7‐8), 603-615.

Sanchez-Duffhues, G., Q Vo, M., Perez, M., A Calzado, M., Moreno, S., Appendino, G., & Munoz, E. (2011). Activation of latent HIV-1 expression by protein kinase C agonists. A novel therapeutic approach to eradicate HIV-1 reservoirs. *Current drug targets*, *12*(3), 348-356.

Achour, A., Aroui, A., Defaa, C., El Mousadik, A., & Msanda, F. (2011). Effet de la mise en défens sur la richesse floristique et la densité dans deux arganeraies de plaine. *Actes du Premier Congrès International de l’Arganier, Agadir*, 15-17.

Mothana, R. A., Al-Musayeib, N. M., Matheeussen, A., Cos, P., & Maes, L. (2012). Assessment of the in vitro antiprotozoal and cytotoxic potential of 20 selected medicinal plants from the island of Soqotra. *Molecules*, *17*(12), 14349-14360.

Genin, M., Alifriqui, M., Fakhech, A., Hafidi, M., Ouahmane, L., & Genin, D. (2017). Back to forests in pre-Saharan Morocco? When prickly pear cultivation and traditional agropastoralism reduction promote argan tree regeneration. *Silva Fennica*, *51*(1B).

Msanda, F., Saadi, B., Elmousadik, A., & Cherifi, K. La flore des arganeraies: élément de la biodiversité du Sud-ouest du Maroc. *Recherches sur les plantes aromatiques et médicinales*, 23.

Msanda, F., Saadi, B., Elmousadik, A., & Cherifi, K. La flore des arganeraies: élément de la biodiversité du Sud-ouest du Maroc. *Recherches sur les plantes aromatiques et médicinales*, 23.

Ghourri, M., Zidane, L., & Douira, A. (2013). Catalogue des plantes médicinales utilisées dans le traitement de la lithiase rénale dans la province de Tan-Tan (Maroc saharien). *International Journal of Biological and Chemical Sciences*, *7*(4), 1688-1700.

Burnett, G. T. (1852). *An Encyclopaedia of Useful and Ornamental Plants: Consisting of Beautiful and Accurate Coloured Figures of Plants Used in the Arts, in Medicine, and for Ornament, with Copious Scientific and Popular Descriptions of Each, Accounts of Their Uses, and Mode of Culture, and Numerous Interesting Anecdotes*. G. Willis.

Aboutayeb, H., Beraaouz, M., & Ezaidi, A. (2016). The great catchment of souss-massa wadi (Morocco): Relationship between protected areas and ecotourism. In *The Souss‐Massa River Basin, Morocco* (pp. 285-302). Springer, Cham.

Haenni, J. P., & Kettani, K. (2011). Première note sur les Scatopsidae du Maroc, avec la description d'une espèce nouvelle (Diptera). *Bulletin de la Société entomologique de France*, *116*(1), 73-79.

ScienceDirect database

Keyword : Euphorbia officinarum

Meyanungsang Kichu, Teresa Malewska, Kaisarun Akter, Imchawati Imchen, David Harrington, James Kohen, Subramanyam R. Vemulpad, Joanne F. Jamie,

An ethnobotanical study of medicinal plants of Chungtia village, Nagaland, India,

Journal of Ethnopharmacology,

Volume 166,

2015,

Pages 5-17,

ISSN 0378-8741,

https://doi.org/10.1016/j.jep.2015.02.053.

(https://www.sciencedirect.com/science/article/pii/S0378874115001415)

Abstract: Ethnopharmacological relevance

Traditional medicinal plant knowledge is an integral and very important part of Indigenous cultures worldwide. For many communities there is a great urgency in recording this knowledge in written form. This is the first ethnobotanical report of medicinal plant knowledge of the Nagaland Ao tribe of Chungtia village and is an important step in the preservation of this culturally and medicinally significant knowledge.

Aim of the study

The aim of the presented work was to perform an ethnobotanical study on plants of medicinal and other significance to the Chungtia villagers of Nagaland, North East India.

Materials and methods

Ethnobotanical data were collected from traditional practitioners and Elders of Chungtia village by means of open group discussions and semi-structured interviews of groups and individuals using questionnaires. The interviews were also recorded in an audio format in the local Mongsen language. The gathered ethnobotanical knowledge was compared with reported ethnobotanical usages worldwide and reported biological properties and phytochemical studies relevant to the Chungtia villagers׳ applications.

Results

A total of 135 plant species of 69 families and 123 genera were recorded for medicinal and household maintenance applications. Those applications were grouped into 13 categories based on Chungtia villagers׳ classification system. The families most represented were Asteraceae, Euphorbiaceae and Solanaceae. The most reported uses were for gastrointestinal problems, followed by dermatological problems. The most commonly used plant parts were leaves, followed by fruits and stems and they were most commonly administered as a paste, decoction, infusion, juice or poultice, or taken orally with no preparation. There was strong agreement among the informants as to the usages of the plants (informant consensus factor 0.80–0.91). The use value of 6 for Cassia floribunda, Dolichos lablab, Hedyotis scandens, Phyllanthus urinaria and Rhus javanica indicated these are the most important species. Forty four of the 135 plants had a fidelity level of 100%.

Conclusion

This study has helped to document and preserve in written format important traditional plant knowledge of 135 plants of the Chungtia villagers, assisting them in the continued preservation of their cultural values.

Keywords: Ao tribe; Biodiversity; Chemical constituents; Ethical engagement; Ethnopharmacology; Traditional knowledge

José Hélio Costa, Allison E. McDonald, Birgit Arnholdt-Schmitt, Dirce Fernandes de Melo,

A classification scheme for alternative oxidases reveals the taxonomic distribution and evolutionary history of the enzyme in angiosperms,

Mitochondrion,

Volume 19, Part B,

2014,

Pages 172-183,

ISSN 1567-7249,

https://doi.org/10.1016/j.mito.2014.04.007.

(https://www.sciencedirect.com/science/article/pii/S1567724914000452)

Abstract: A classification scheme based on protein phylogenies and sequence harmony method was used to clarify the taxonomic distribution and evolutionary history of the alternative oxidase (AOX) in angiosperms. A large data set analyses showed that AOX1 and AOX2 subfamilies were distributed into 4 phylogenetic clades: AOX1a–c/1e, AOX1d, AOX2a–c and AOX2d. High diversity in AOX family compositions was found. While the AOX2 subfamily was not detected in monocots, the AOX1 subfamily has expanded (AOX1a–e) in the large majority of these plants. In addition, Poales AOX1b and 1d were orthologous to eudicots AOX1d and then renamed as AOX1d1 and 1d2. AOX1 or AOX2 losses were detected in some eudicot plants. Several AOX2 duplications (AOX2a–c) were identified in eudicot species, mainly in the asterids. The AOX2b originally identified in eudicots in the Fabales order (soybean, cowpea) was divergent from AOX2a–c showing some specific amino acids with AOX1d and then it was renamed as AOX2d. AOX1d and AOX2d seem to be stress-responsive, facultative and mutually exclusive among species suggesting a complementary role with an AOX1(a) in stress conditions. Based on the data collected, we present a model for the evolutionary history of AOX in angiosperms and highlight specific areas where further research would be most beneficial.

Keywords: Alternative oxidase; Flowering plants; Taxonomic distribution; Classification; Evolution

Paolo Maria Guarrera,

Traditional antihelmintic, antiparasitic and repellent uses of plants in Central Italy,

Journal of Ethnopharmacology,

Volume 68, Issues 1–3,

1999,

Pages 183-192,

ISSN 0378-8741,

https://doi.org/10.1016/S0378-8741(99)00089-6.

(https://www.sciencedirect.com/science/article/pii/S0378874199000896)

Abstract: The uses of 51 plants of Marche, Abruzzo and Latium, distributed in 28 families, are listed here. Memories and news of continued use of the plants in these sectors were collected from farmers and shepherds in person (mostly old people). The plants most frequently used as antiparasitics and repellents are Juglans regia, Lupinus albus, Ruta graveolens, Fraxinus ornus, Datura stramonium, Artemisia absinthium, Allium cepa, while R. graveolens, Cucurbita maxima, A. absinthium, Allium sativum are the most commonly used as antihelmintics.

Keywords: Antihelmintic; Antiparasitic; Repellent; Marche; Abruzzo; Latium

Carmen Van Mechelen, Koenraad Van Meerbeek, Thierry Dutoit, Martin Hermy,

Functional diversity as a framework for novel ecosystem design: The example of extensive green roofs,

Landscape and Urban Planning,

Volume 136,

2015,

Pages 165-173,

ISSN 0169-2046,

https://doi.org/10.1016/j.landurbplan.2014.11.022.

(https://www.sciencedirect.com/science/article/pii/S0169204614003065)

Abstract: Novel urban ecosystems have recently gained importance and a consensus grows that ecosystem services are linked to ecosystem properties that in turn can be predicted by functional diversity (FD) of plant communities. Ensuring quality of life in cities is essential and approaches to improve ecosystem services should be encouraged. Here we analyzed the initial plant composition of commercial extensive green roof systems in terms of FD and propose two methods to compose species lists that maximize FD. Clustering techniques grouped the systems in three green roof types that differ in species richness and composition, substrate depth and installation method. Differences in FD and species richness between the types were considerable and correlations between FD and species richness were strongly positive, suggesting that species-rich systems generally have higher FD and thus offer better ecosystem services. Green roof systems that comprise only succulents have significant lower FD values compared to systems containing more diverse vegetation. Within the green roof types there was room for improvement of FD. Based on a species trait dissimilarity matrix, species lists that maximize FD for every green roof type were created. FD analysis of novel ecosystems enables the design of both species-poor and species-rich green roof systems with improved overall ecosystem services value. The described approach is generic in nature and can be used for a broad range of novel ecosystems and urban green elements. We believe that designing functional diverse plant systems will support a more sustainable urban planning and improve the quality of urban life.

Keywords: Ecosystem services; Functional diversity; Green roofs; Novel ecosystems; Plant traits; Species richness

Amal Smaili, Noureddine Mazoir, Lalla Aicha Rifai, Tayeb Koussa, Kacem Makroum, El Mostafa Kabil, Ahmed Benharref, Mohamed Faize,

Triterpene derivatives from Euphorbia enhance resistance against Verticillium wilt of tomato,

Phytochemistry,

Volume 135,

2017,

Pages 169-180,

ISSN 0031-9422,

https://doi.org/10.1016/j.phytochem.2016.12.017.

(https://www.sciencedirect.com/science/article/pii/S0031942216303028)

Abstract: Oxidation of α-euphorbol and 31-norlanostenol, two triterpenic compounds isolated from the latex of Euphorbia resinifera and Euphorbia officinarum respectively, yielded four products named 3β-tosyloxy-4α,14α-dimethyl-5α-cholesta-7,9-diene; 4α,14α-dimethyl-5α-cholesta-7,9-dien-3β-ol; 24-methylen-elemo-lanosta-8,24-dien-3-one and elemo-lanost-8-en-3,11,24-trione. They were evaluated for protection of tomato plants against Verticillium dahliae in a greenhouse. The four semisynthesized products were phytotoxic at higher concentrations as they completely inhibited tomato germination at 100 and 500 μg/ml. However at lower concentrations (10 and 50 μg/ml) germination and root length were not affected. Disease resistance against Verticillium wilt was assessed in tomato plants derived from seeds that germinated in the presence of 10 and 50 μg/ml of the four products. All of them were able to reduce significantly disease severity, with 10 μg/ml being more effective than 50 μg/ml. Reduction of leaf alteration index and of stunting index ranged from 52 to 68% and from 43 to 67%, respectively, while vessel discoloration was reduced by at least 95%. The compounds were also able to elicit H2O2 accumulation before and after fungal inoculation and to significantly enhance peroxidase and polyphenol oxidase activities. These results suggest that the hemisynthetized triterpenes can be used as elicitors of disease resistance.

Keywords: Euphorbia officinarum; Euphorbia resinifera; Euphorbiaceae; Solanum lycopersicum; Solanaceae; Triterpene derivatives; Verticillium dahliae; Elicitor; Resistance

Bradley C. Bennett, Rocío Alarcón,

Hunting and hallucinogens: The use psychoactive and other plants to improve the hunting ability of dogs,

Journal of Ethnopharmacology,

Volume 171,

2015,

Pages 171-183,

ISSN 0378-8741,

https://doi.org/10.1016/j.jep.2015.05.035.

(https://www.sciencedirect.com/science/article/pii/S0378874115003670)

Abstract: Ethnopharmacological relevance

Cultures throughout the world give plants to their dogs in order to improve hunting success. These practices are best developed in lowland Ecuador and Peru. There is no experimental evidence for the efficacy of these practices nor critical reviews that consider possible pharmacological effects on dogs based on the chemistry of the ethnoverterinary plants.

Aim

This review has three specific aims: (1) determine what plants the Ecuadorian Shuar and Quichua give to dogs to improve their hunting abilities, (2) determine what plants other cultures give to dogs for the same purpose, and (3) assess the possible pharmacological basis for the use of these plants, particularly the psychoactive ones.

Methods

We gathered Shuar (Province of Morona-Santiago) and Quichua (Napo and Orellano Provinces) data from our previous publications and field notes. All specimens were vouchered and deposited in QCNE with duplicates sent to NY and MO. Data presented from other cultures derived from published studies on ethnoveterinary medicine. Species names were updated, when necessary, and family assignments follow APG III (Angiosperm Phylogeny Group, 2009. An update of the Angiosperm Phylogeny Group classification for the orders and families of flowering plants: APG III. Bot. J. Linn. Soc. 161, 105–121). Chemical data were found using PubMed and SciFinder.

Results

The Shuar and Quichua of Ecuador use at least 22 species for ethnoveterinary purposes, including all but one of their principal hallucinogens. Literature surveys identified 43 species used in other cultures to improve hunting ability. No published studies have examined the pharmacological active of these plant species in dogs. We, thus, combined phytochemical data with the ethnobotanical reports of each plant and then classified each species into a likely pharmacological category: depuratives/deodorant, olfactory sensitizer, ophthalmic, or psychoactive.

Conclusions

The use of psychoactive substances to improve a dog׳s hunting ability seems counterintuitive, yet its prevalence suggests that it is both adaptive and that it has an underlying pharmacological explanation. We hypothesize that hallucinogenic plants alter perception in hunting dogs by diminishing extraneous signals and by enhancing sensory perception (most likely olfaction) that is directly involved in the detection and capture of game. If this is true, plant substances also might enhance the ability of dogs to detect explosives, drugs, human remains, or other targets for which they are valued.

Keywords: Ecuador; Hallucinogens; Hunting; Psychoactive plants; Shuar; Quichua

B. Bading Taika, M. Bouckandou, A. Souza, H.P. Bourobou Bourobou, L.S. MacKenzie, L. Lione,

An overview of anti-diabetic plants used in Gabon: Pharmacology and toxicology,

Journal of Ethnopharmacology,

Volume 216,

2018,

Pages 203-228,

ISSN 0378-8741,

https://doi.org/10.1016/j.jep.2017.12.036.

(https://www.sciencedirect.com/science/article/pii/S0378874117316677)

Abstract: Ethnopharmacological relevance

The management of diabetes mellitus management in African communities, especially in Gabon, is not well established as more than 60% of population rely on traditional treatments as primary healthcare. The aim of this review was to collect and present the scientific evidence for the use of medicinal plants that are in currect by Gabonese traditional healers to manage diabetes or hyperglycaemia based here on the pharmacological and toxicological profiles of plants with anti-diabetic activity. There are presented in order to promote their therapeutic value, ensure a safer use by population and provide some bases for further study on high potential plants reviewed.

Materials and methods

Ethnobotanical studies were sourced using databases such as Online Wiley library, Pubmed, Google Scholar, PROTA, books and unpublished data including Ph.D. and Master thesis, African and Asian journals. Keywords including ‘Diabetes’, ‘Gabon’, ‘Toxicity’, ‘Constituents’, ‘hyperglycaemia’ were used.

Results

A total of 69 plants currently used in Gabon with potential anti-diabetic activity have been identified in the literature, all of which have been used in in vivo or in vitro studies. Most of the plants have been studied in human or animal models for their ability to reduce blood glucose, stimulate insulin secretion or inhibit carbohydrates enzymes. Active substances have been identified in 12 out of 69 plants outlined in this review, these include Allium cepa and Tabernanthe iboga. Only eight plants have their active substances tested for anti-diabetic activity and are suitables for further investigation. Toxicological data is scarce and is dose-related to the functional parameters of major organs such as kidney and liver.

Conclusion

An in-depth understanding on the pharmacology and toxicology of Gabonese anti-diabetic plants is lacking yet there is a great scope for new treatments. With further research, the use of Gabonese anti-diabetic plants is important to ensure the safety of the diabetic patients in Gabon.

Keywords: Diabetes mellitus; Gabon; Medicinal plants; Anti-diabetic activity; Toxicity

Frederick W. Fraunfelder,

Ocular side effects from herbal medicines and nutritional supplements,

American Journal of Ophthalmology,

Volume 138, Issue 4,

2004,

Pages 639-647,

ISSN 0002-9394,

https://doi.org/10.1016/j.ajo.2004.04.072.

(https://www.sciencedirect.com/science/article/pii/S0002939404005100)

Abstract: Purpose

To review the more significant herbal and nutritional agents of clinical importance to ophthalmologists and describe the ocular side effects for each. World Health Organization (WHO) classification and guidelines for clinicians are provided.

Design

Retrospective observational case series.

Methods

A retrospective observational case series of reports of ocular side effects or systemic side effects from medications used for the eye from herbal medicines and nutritional supplements. Cases were collected from spontaneous reports submitted to the WHO, the Food and Drug Administration, and the National Registry of Drug-Induced Ocular Side Effects. A review of the world's literature was performed to obtain additional case reports and insight into adverse ocular reactions. Data were collected on age, gender, duration of therapy, concomitant medications, dosage, and dechallenge and rechallenge results.

Results

The National Registry of Drug-Induced Ocular Side Effects received 263 spontaneous reports, in addition to 60 case reports from the literature. Canthaxanthine, chamomile, Datura, Echinacea purpurea, Ginkgo biloba, licorice, niacin, and vitamin A are all associated with clinically significant ocular side effects.

Conclusion

Herbal medicines and nutritional supplements can cause ocular side effects. Clinicians need to recognize these adverse events, because a large segment of the population uses them, many times without the treating physician's knowledge.

François Chassagne, Sovanmoly Hul, Eric Deharo, Geneviève Bourdy,

Natural remedies used by Bunong people in Mondulkiri province (Northeast Cambodia) with special reference to the treatment of 11 most common ailments,

Journal of Ethnopharmacology,

Volume 191,

2016,

Pages 41-70,

ISSN 0378-8741,

https://doi.org/10.1016/j.jep.2016.06.003.

(https://www.sciencedirect.com/science/article/pii/S0378874116303555)

Abstract: Ethnopharmacological relevance

In this paper we present a comprehensive ethnomedicinal study conducted in Mondulkiri province. Traditional knowledge about natural medicine (plants, animals, mushrooms) was investigated in Cambodia’s largest indigenous community: the Bunong people. The survey aims to document the medicinal plant use of this ethnic, by focusing on the eleven most frequent diseases encountered in the area, in order to highlight species that could be recommended in public health programs.

Materials and methods

During the years 2013 and 2014, 202 villagers were interviewed in 28 villages from the five districts in Mondulkiri. Two types of methodology were employed: (1) an ethnobotanical field survey (walk-in-the-wood interviews) and (2) semi-structured household interviews with a special emphasis on the treatment of 11 most common ailments encountered in the area. Medicinal plants and mushrooms were collected and identified together with medicinal animals. The factor informant consensus (FIC) and fidelity level (FL) were calculated.

Results

Bunong people use a total of 214 plants belonging to 72 families, 1 mushroom and 22 animal species in their traditional healthcare practices in order to treat 51 different ailments. Among the medicinal plants, Fabaceae was the most predominant family; Chromolaena odorata (L.) R.M. King and H.Rob. (Asteraceae), Zingiber montanum (J.Koenig) Link ex A.Dietr. (Zingiberaceae) and Kalanchoe pinnata (Lam.) Pers. (Crassulaceae) were the most cited medicinal plants; and four ailments (cold/fever, diarrhea, postpartum disorders and stomachache) were described as major ailments in the community. The root was the most important part of plants used, and decoction was the most cited method of preparation. During our survey, we also discovered a “new to science” plant species called Ardisia mondulkiriensis Hul and Chassagne, and we recorded for the second time the plant species recently described, Solanum sakhanii Hul.

Conclusion

Most of the species reported for the treatment of the 11 most frequent ailments have already been proven to be efficient and safe. Furthermore, 10 plant species are reported for the first time as medicinal and some of them are widely used in the community. Further pharmacological and phytochemical investigations should be undergone to assess the pharmaceutical potential of these species. While undergoing considerable changes, Bunong people have maintained extensive traditional medicine knowledge. As this indigenous hill tribe depend mainly on natural remedies for their daily healthcare, environmental preservation is of high importance for the community.

Keywords: Cambodia; Bunong people; Ethnobotanical survey; Medicinal plant; Indigenous knowledge

Katrin Henning, Antje Lorenz, Goddert von Oheimb, Werner Härdtle, Sabine Tischew,

Year-round cattle and horse grazing supports the restoration of abandoned, dry sandy grassland and heathland communities by supressing Calamagrostis epigejos and enhancing species richness,

Journal for Nature Conservation,

Volume 40,

2017,

Pages 120-130,

ISSN 1617-1381,

https://doi.org/10.1016/j.jnc.2017.10.009.

(https://www.sciencedirect.com/science/article/pii/S1617138117302261)

Abstract: The spread of competitive grasses, changes in species composition and vegetation structure are direct consequences of grassland and heathland abandonment. As an alternative to more costly management measures such as traditional pastoralism, year-round low-intensity grazing with large herbivores is increasingly used to restore and maintain semi-open habitats. However, the suitability of this grazing regime has not yet been investigated for long-abandoned, highly degraded but nutrient-poor sandy grassland and heathland communities. In particular, it is unclear if year-round grazing is suitable for preventing the further spread of highly competitive grasses such as Calamagrostis epigejos while simultaneously maintaining or improving characteristic species richness and vegetation structure. Hence, we conducted a comprehensive field study on two spatial scales (plot-level: 25m2, macroplot-level: 1ha) to analyse the impacts of year-round low-intensity cattle and horse grazing on the development of the highly competitive grass Calamagrostis epigejos, as well as the vegetation structure and plant species richness of long-abandoned but nutrient-poor dry sandy grassland and heathland communities, their mosaics and Calamagrostis stands within an 800ha heathland between 2008 and 2015. Finally, we assessed the local conservation status of the habitat types after seven years of grazing in comparison to long-abandoned sites. Grazing successfully reduced the coverage of Calamagrostis epigejos, whereby Calamagrostis stands developed towards species-rich sandy grasslands after seven years of grazing. In addition, the quality of the vegetation structure was improved by enhancing the proportion of bare soil, while litter and grass cover, litter thickness and height of the field layer as well as the coverage of ruderal indicators were significantly reduced on grazed sites in comparison to ungrazed sites in 2015. Moreover, we found an overall positive grazing effect on species richness: Total species number, number of target species as well as subordinated target species significantly increased within the vegetation types over time. Thus, year-round low-intensity cattle and horse grazing is a suitable management tool for restoring, maintaining and even improving long-abandoned, nutrient-poor sandy grassland and heathland communities, and thus to enhance the local conservation status of the habitat types. However, if there is a high initial cover of woody species (e.g. shrubs, tree rejuvenation), then an extensive shrub and tree clearance will be necessary, with manual shrub cutting being crucial to reduce the heavy regrowth of the woody species. In addition, a one-time mowing should be implemented in highly degraded heaths to facilitate the vegetative rejuvenation of degenerate stands of Calluna vulgaris, thus improving its attractiveness for the grazing animals.

Keywords: Calamagrostis epigejos; Large and small-scale surveys; Low-intensity grazing; Species richness; Vegetation structure

Raphaële Boydron-Le Garrec, Evelyne Benoit, Martin-Pierre Sauviat, Richard J. Lewis, Jordi Molgó, Dominique Laurent,

Ability of some plant extracts, traditionally used to treat ciguatera fish poisoning, to prevent the in vitro neurotoxicity produced by sodium channel activators,

Toxicon,

Volume 46, Issue 6,

2005,

Pages 625-634,

ISSN 0041-0101,

https://doi.org/10.1016/j.toxicon.2005.07.002.

(https://www.sciencedirect.com/science/article/pii/S0041010105002370)

Abstract: The effects of 31 plant extracts, which most are traditionally used to treat ciguatera fish poisoning in the Pacific area, were studied on the cytotoxicity of mouse neuroblastoma cells produced by ouabain, veratridine and/or brevetoxin-3 or Pacific ciguatoxin-1. The cell viability was determined using a quantitative colorimetric method. A marked cytotoxicity of seven of the 31 plant extracts studied, was observed. Despite this, these plant extracts were suspected to contain active compound(s) against the cytotoxicity produced by brevetoxin (2 extracts), brevetoxin, ouabain and/or veratridine (3 extracts), or only against that of ouabain and/or veratridine (2 extracts). Among the 24 plant extracts that exhibited by themselves no cytotoxicity, 22 were active against the effect of brevetoxin or against that of both veratridine and brevetoxin. Similar results were obtained when the seven most active plant extracts were reassayed using ciguatoxin instead of brevetoxin. In conclusion, the present work reports the first activity assessment of some plant extracts, achieved in vitro on a quite large scale. The fact that 27 plant extracts were found to exert, in vitro, a protective effect against the action of ciguatoxin and/or brevetoxin, paves the way for finding new active compounds to treat ciguatera fish poisoning, provided these compounds also reverse the effects of sodium channel activators.

Keywords: Ciguatera fish poisoning; Treatment; Traditional remedies; Neuroblastoma cell assay; Voltage-sensitive sodium channels; Brevetoxin; Ciguatoxin; Veratridine

Plant index,

Fitoterapia,

Volume 74, Issues 1–2,

2003,

Pages 198-205,

ISSN 0367-326X,

https://doi.org/10.1016/S0367-326X(02)00326-X.

(https://www.sciencedirect.com/science/article/pii/S0367326X0200326X)

Marion Mackonochie, Michael Heinrich,

Materia medica chests: Investigating the 19th century use of botanicals by different medical professions,

Journal of Herbal Medicine,

Volume 16,

2019,

100255,

ISSN 2210-8033,

https://doi.org/10.1016/j.hermed.2019.100255.

(https://www.sciencedirect.com/science/article/pii/S2210803319300016)

Abstract: The use of herbal substances was an element of everyday medicine until the advent of synthetic medicines from the late 19th Century onwards. Medicinal chests were used as teaching and examination tools for apothecaries and pharmacists. The contents of two 19th Century materia medica chests that are owned by the Worshipful Society of Apothecaries in London (LSA) were analysed and compared with written texts from the time in order to understand botanical drug knowledge in 19th Century Britain. The samples in the chests and any associated information was documented and analysed. The pharmacists' examination chest contained 84 botanical drug samples, while 94 botanical drugs could be identified in the apothecaries' chest; 45 of these were present in both chests. Minimal changes in herb use over the 19th Century and limited variation of the botanical drugs used by different medical professionals were found. There are some differences in respect to the therapeutic areas which were of importance - the pharmacists' chest contained some potent purgatives and toxic drugs, while botanical drugs unique to the apothecaries' chest were gentle digestives and tonic herbs. Comparison of the chest contents with texts from the 19th Century has indicated that no single historical source provides a complete picture of botanical drug use at the time. This is a pilot study highlighting the potential of such chests in research on the history of European herbal medicine, and a more systematic study including research on the botanical drugs' chemical composition and authenticity is warranted.

Keywords: Apothecaries; Herbal; History; Medicine chest; Pharmacy; European phytotherapy

Lucia Viegi, Andrea Pieroni, Paolo Maria Guarrera, Roberta Vangelisti,

A review of plants used in folk veterinary medicine in Italy as basis for a databank,

Journal of Ethnopharmacology,

Volume 89, Issues 2–3,

2003,

Pages 221-244,

ISSN 0378-8741,

https://doi.org/10.1016/j.jep.2003.08.003.

(https://www.sciencedirect.com/science/article/pii/S0378874103003003)

Abstract: We report folk veterinary phytotherapy in Italy collected from ethnobotanical scientific literature of the second half of the 20th Century. References are cited together with unpublished data gathered recently in the field by the authors. The data have been placed in two databases: one organized by the names of the plant species (>260) and the other organized by bibliographic references. This represents the basis for the first national databank for ethnoveterinary botany in Europe. Plants not yet sufficiently studied in pharmacology and veterinary phytotherapy were also identified.

Keywords: Ethnoveterinary; Ethnobotany; Medicinal plants; Italy

D.S. Manamgoda, A.Y. Rossman, L.A. Castlebury, P.W. Crous, H. Madrid, E. Chukeatirote, K.D. Hyde,

The genus Bipolaris,

Studies in Mycology,

Volume 79,

2014,

Pages 221-288,

ISSN 0166-0616,

https://doi.org/10.1016/j.simyco.2014.10.002.

(https://www.sciencedirect.com/science/article/pii/S0166061614000311)

Abstract: The genus Bipolaris includes important plant pathogens with worldwide distribution. Species recognition in the genus has been uncertain due to the lack of molecular data from ex-type cultures as well as overlapping morphological characteristics. In this study, we revise the genus Bipolaris based on DNA sequence data derived from living cultures of fresh isolates, available ex-type cultures from worldwide collections and observation of type and additional specimens. Combined analyses of ITS, GPDH and TEF gene sequences were used to reconstruct the molecular phylogeny of the genus Bipolaris for species with living cultures. The GPDH gene is determined to be the best single marker for species of Bipolaris. Generic boundaries between Bipolaris and Curvularia are revised and presented in an updated combined ITS and GPDH phylogenetic tree. We accept 47 species in the genus Bipolaris and clarify the taxonomy, host associations, geographic distributions and species’ synonymies. Modern descriptions and illustrations are provided for 38 species in the genus with notes provided for the other taxa when recent descriptions are available. Bipolaris cynodontis, B. oryzae, B. victoriae, B. yamadae and B. zeicola are epi- or neotypified and a lectotype is designated for B. stenospila. Excluded and doubtful species are listed with notes on taxonomy and phylogeny. Seven new combinations are introduced in the genus Curvularia to accomodate the species of Bipolaris transferred based on the phylogenetic analysis. A taxonomic key is provided for the morphological identification of species within the genus.

Keywords: Brown spot of rice; Field crop diseases; Graminicolous fungi; Helminthosporoid genera; molecular phylogeny; Pleosporales; Southern corn leaf blight; Taxonomy

N.A. Noukeu, R.J. Priso, S.D. Dibong, D. Ndongo, L. Kono, D. Essono,

Floristic diversity of receiving environments polluted by effluent from agri-food industries,

Heliyon,

Volume 5, Issue 11,

2019,

e02747,

ISSN 2405-8440,

https://doi.org/10.1016/j.heliyon.2019.e02747.

(https://www.sciencedirect.com/science/article/pii/S2405844019364072)

Abstract: Till date, there are few studies on the flora found in receiving environments polluted by effluent from agri-food industries. Floristic inventories of ten receiving environments in Cameroon. were carried out using the line transect method from upstream to downstream discharge areas in the Littoral and Center regions during the dry and rainy season. The abundance/dominance (AD) of each floristic survey was assessed using Braun–Blanquet scale. Species richness of the different receiving environments is marked by higher and lower Shannon Weaver (H′) diversity index values, respectively in the rainy season and dry season from upstream to downstream. Regularity values (R) show that the maximum number of species is involved in the covering of the surface. In terms of floristic composition, the Simpson's diversity index (D) shows similarities between the different receiving environments. The Sorensen index (Q) shows similar number of common species between upstream and downstream zones of the same site. Nitrophilous species are abundant. Some could be organic pollution indicators, namely: Pennisetum purpureum, Cynodon dactylon, Commelina benghalensis, Lemna minor, Acroceras zizanoides, Echinochloa pyramidalis and Panicum maximum. The Poaceae family dominates the ten receiving environments.

Keywords: Agriculture; Environmental science; Receiving environments; Species richness; Nitrophilous species; Agri-food industries; Organic pollution

Plant index Volume 77 (2006),

Fitoterapia,

Volume 77, Issues 7–8,

2006,

Pages 636-639,

ISSN 0367-326X,

https://doi.org/10.1016/S0367-326X(06)00245-0.

(https://www.sciencedirect.com/science/article/pii/S0367326X06002450)

Tuhin K. Biswas,

Chapter 1 - Drug Discovery From Ayurveda: Mode of Approach and Applications,

Editor(s): Subhash C. Mandal, Vivekananda Mandal, Tetsuya Konishi,

Natural Products and Drug Discovery,

Elsevier,

2018,

Pages 3-28,

ISBN 9780081020814,

https://doi.org/10.1016/B978-0-08-102081-4.00001-0.

(https://www.sciencedirect.com/science/article/pii/B9780081020814000010)

Abstract: For thousands of years, Ayurveda, the science of life, has dealt with the promotion of health, prevention of diseases, and cure of various ailments through natural ways, utilizing drugs of medicinal plants, mineral and metals, and animal derivatives. Ayurveda is one of the important axes among all other components of AYUSH (Ayurveda, Yoga, Unani, Siddha, and Homeopathy), practiced in India. Diseases and their pathogenesis are described in Ayurveda in a systematic manner on the basis of individual variations, which is now explained under the parlance of personalized and predictive medicines. Different therapeutics and procedures are designed from natural resources on the basis of this fundamental principle of individualism, which is unique among all other traditional and complementary systems of medicine in the world. The development of ideal drug(s) for a specific disease from Ayurveda depends upon the approach of its pharmacodynamics, as explained in Ayurvedic theories, and therapeutic application of such can be exemplified through modern technologies of screening. Drugs of Ayurvedic origin for the treatment of diseases under lifestyle disorders such as joint pathology, metabolic syndrome, chronic respiratory problems, gastrointestinal upset, geriatric problems, gynecological abnormalities, wound management, psychobehavioral upset, or nutritional deficiencies are the best instances to explore the basic theories of its application. The avenue of drug development involves a literature survey from Ayurveda, chemical screening of natural resources, pharmacological evaluation, genetic stratification, and clinical application in certain areas of biomedical science, which will be detailed in this chapter.

Keywords: Ayurveda; AYUSH; Drug discovery; Lifestyle disorders; Nutritional deficiencies; Psychobehavioral problems

Editor(s): Christophe Wiart,

Lead Compounds from Medicinal Plants for the Treatment of Cancer,

Academic Press,

2013,

Pages 403-410,

ISBN 9780123983718,

https://doi.org/10.1016/B978-0-12-398371-8.00019-2.

(https://www.sciencedirect.com/science/article/pii/B9780123983718000192)

Anita Jain, S.S. Katewa, B.L. Chaudhary, Praveen Galav,

Folk herbal medicines used in birth control and sexual diseases by tribals of southern Rajasthan, India,

Journal of Ethnopharmacology,

Volume 90, Issue 1,

2004,

Pages 171-177,

ISSN 0378-8741,

https://doi.org/10.1016/j.jep.2003.09.041.

(https://www.sciencedirect.com/science/article/pii/S0378874103003647)

Abstract: An ethnobotanical survey of tribal area of southern Rajasthan was carried out during the year 2001–2002 for ethnosexicological herbal medicines. The information on ethnosexicological herbs is based on the exhaustive interview with local medicine-men and -women, birth attendants and other knowledgeable persons who prescribe their own herbal preparation to check birth control, including abortion at initial stages, preventing conception or by making either member of the couple sterile and to cure various sexual diseases like leucorrhoea, gonorrhoea, menorrhagia, to regularize menses and syphilis in both the sexes. During ethnobotanical survey, 53 plants belonging to 33 families have been reported from the study area, which are used to cure sexual diseases, and for family planning. A list of plant species along with their local name, habit, flowering and fruiting period, plant part/s used and the mode of administration to cure the sexual diseases are given.

Keywords: Ethnobotany; Folk herbal medicines; Tribals; Antifertility; Sexual diseases; Southern Rajasthan

Swee-Ling Lim, Suhaila Mohamed,

Functional food and dietary supplements for lung health,

Trends in Food Science & Technology,

Volume 57, Part A,

2016,

Pages 74-82,

ISSN 0924-2244,

https://doi.org/10.1016/j.tifs.2016.08.006.

(https://www.sciencedirect.com/science/article/pii/S0924224416303478)

Abstract: Background

The World Health Organisation reported the global leading causes of death in the past decade were ischaemic heart disease (7.4 million), stroke (6.7 million), lower respiratory infections (3.1 million) and chronic obstructive lung disease (3.1 million). Lung cancers (along with trachea and bronchus cancers) caused 1.6 million (2.9%) deaths in 2012, up from 1.2 million (2.2%) deaths in 2000. Lung-related ailments (asthma, chronic obstructive pulmonary diseases, influenza and lung cancer) are common worldwide with high mortality rates. The major killer diseases in the world are related to the lungs (8 million) which statistically makes it the top cause of death.

Scope and approach

Various plants contain metabolites that help against lung ailments and have been demonstrated in in vitro, in vivo and clinical studies. This review compiles the scientific reports and findings resulting from the use of plant extracts as a complementary therapy or dietary supplement for lung health and their underlying mechanisms.

Key findings and conclusions

These plant metabolites benefit lung health by anti-inflammatory, immune-modulatory, and antioxidant effects. Many pungent flavor components in edible plants activate the mucous membranes of the respiratory tract to help fight microbes and remove undesirable agents. These flavor and aroma compounds can behave as expectorants, decongestant, antitussive, and antimicrobial agents. They enhance mucous membrane secretion, kill pathogens, reduce inflammation and decrease mucous viscosity by weakening the hydrogen bonds in the mucous. For lung cancer, certain plant metabolites help induce apoptosis, suppress angiogenesis (cancer-related new blood vessel growth) and suppress inflammatory pathways.

Keywords: Plant; Lung cancer; Asthma; Influenza; Chronic obstructive pulmonary diseases

Sonja Sytwala, Florian Günther, Matthias F. Melzig,

Lysozyme- and chitinase activity in latex bearing plants of genus Euphorbia – A contribution to plant defense mechanism,

Plant Physiology and Biochemistry,

Volume 95,

2015,

Pages 35-40,

ISSN 0981-9428,

https://doi.org/10.1016/j.plaphy.2015.07.004.

(https://www.sciencedirect.com/science/article/pii/S0981942815300504)

Abstract: Occurrence of latices in plants is widespread, there are 40 families of plants characterized to establish lactiferous structures. Latices exhibit a constitutive part of plant defense due to the stickiness. The appearance of proteins incorporated in latices is well characterized, and hydrolytic active proteins are considerable. A lot of plants constitute so-called pathogenesis-related (PR) proteins, to overcome stressful conditions. In our investigation we are focused on latex bearing plants of Euphorbiaceae Juss., and investigated the appearance of chitinase- and lysozyme activity in particular. The present outcomes represent a comprehensive study, relating to the occurrence of lysozyme and chitinase activity of genus Euphorbia at the first time. 110 different species of genus Euphorbia L. were tested, and the appearance of chitinase and lysozyme were determined in different quantities. The appearance itself, and the physicochemical properties of latices indicate an efficient interaction for plant defense against pathogen attack.

Keywords: Plant latex; Euphorbiaceae; Chitinase; Lysozyme; Plant defense

Ismail Bettar, M. Lourdes González-Miret, Dolores Hernanz, Alfredo Marconi, Francisco J. Heredia, Anass Terrab,

Characterisation of Moroccan Spurge (Euphorbia) honeys by their physicochemical characteristics, mineral contents and colour,

Arabian Journal of Chemistry,

Volume 12, Issue 8,

2019,

Pages 2052-2060,

ISSN 1878-5352,

https://doi.org/10.1016/j.arabjc.2015.01.003.

(https://www.sciencedirect.com/science/article/pii/S1878535215000155)

Abstract: The quality of 27 Moroccan Spurge (Euphorbia) honey samples was assessed. Eight physicochemical parameters and mineral composition were analysed and the CIELAB colour parameters (L∗, a∗, b∗, Cab∗ and hab) were determined. Results show no significant differences between the two Euphorbia honey types (Euphorbia officinarum subsp. echinus and Euphorbia regis-jubae honeys) regarding the physicochemical parameters. Sodium and magnesium show average values that can help to differentiate between E. officinarum subsp. echinus and E. regis-jubae honeys. Potassium was quantitatively the most important mineral (66% of the total minerals quantified), while sodium and calcium were present in moderate amounts (20% and 11% of the minerals, respectively). The colour parameters also have shown significant differences between E. officinarum subsp. echinus and E. regis-jubae honeys. Considering the total information from physicochemical, mineral and colour data, Principal Component Analysis (PCA) and Stepwise Discriminant Analysis (SDA) were carried out to distinguish between the two Euphorbia honey types. PCA showed that the cumulative variance was approximately 56%. The results of SDA showed that parameters with a higher discriminant power were Na, Mg, L∗, Cab∗ and hab, and almost 100% of the samples were properly classified in their corresponding group, except for one sample.

Keywords: Unifloral honey; Euphorbia officinarum subsp. echinus; Euphorbia regis-jubae; PCA; SDA

Claudio Leto, Teresa Tuttolomondo, Salvatore La Bella, Mario Licata,

Ethnobotanical study in the Madonie Regional Park (Central Sicily, Italy)—Medicinal use of wild shrub and herbaceous plant species,

Journal of Ethnopharmacology,

Volume 146, Issue 1,

2013,

Pages 90-112,

ISSN 0378-8741,

https://doi.org/10.1016/j.jep.2012.11.042.

(https://www.sciencedirect.com/science/article/pii/S0378874112008197)

Abstract: Ethnopharmacological relevance

This paper illustrates the results of an ethnobotanical study carried out in the Madonie Regional Park (Central Sicily, Italy). It specifies the medicinal uses of plants in the study area and contains the results of a quantitative analysis carried out for the first time in an area noted for its high degree of biodiversity. It also introduces 28 species not previously accounted for in the area of study for their medicinal uses, highlighting Silene flos-cuculi L. Greuter & Burdet, little known as medicinal in the Mediterranean area.

Aim of the study

To understand to what extent current knowledge on medicinal-use plants is still an element of the culture within the elderly population of the Madonie Regional Park.

Methodology

The information was obtained using a semi-structured interview format performed on 150 informants over the age of 60 who were considered experts in plants and rural traditions. The taxa were identified and the results were analysed also using a range of quantitative ethnobotanical indices.

Results

A census was made of 174 wild plant species, 100 of which with medicinal and veterinary uses, belonging to 49 botanical families. Of the 170 endemic species found in the Madonie Regional Park, only 2 species were cited in this study for medicinal purposes. Most of the species were used against dermatological diseases, general health and metabolic disorders. The leaves were the most-used parts of the plant and the most common preparation methods were decoction and infusion. The level of knowledge on medicinal uses of the plants was not found to be high within the elderly population, demonstrating an ongoing process of cultural erosion.

Conclusions

Only very few medicinal uses are widely known by all the informants and, on many occasions, a specific medicinal use was cited by only very few people. Further study is required in order to find out to what extent knowledge on the medicinal use of plants is still present in the younger generations in this area of Sicily, and what methods might be adopted in order to halt this gradual loss in knowledge.

Keywords: Ethnobotany; Medicinal plant uses; Madonie Regional Park; Sicily; Traditional medicine

Editor(s): Christophe Wiart,

Lead Compounds from Medicinal Plants for the Treatment of Cancer,

Academic Press,

2013,

Pages 397-401,

ISBN 9780123983718,

https://doi.org/10.1016/B978-0-12-398371-8.00018-0.

(https://www.sciencedirect.com/science/article/pii/B9780123983718000180)

James H. Diaz,

Poisoning by Herbs and Plants: Rapid Toxidromic Classification and Diagnosis,

Wilderness & Environmental Medicine,

Volume 27, Issue 1,

2016,

Pages 136-152,

ISSN 1080-6032,

https://doi.org/10.1016/j.wem.2015.11.006.

(https://www.sciencedirect.com/science/article/pii/S1080603215004305)

Abstract: The American Association of Poison Control Centers has continued to report approximately 50,000 telephone calls or 8% of incoming calls annually related to plant exposures, mostly in children. Although the frequency of plant ingestions in children is related to the presence of popular species in households, adolescents may experiment with hallucinogenic plants; and trekkers and foragers may misidentify poisonous plants as edible. Since plant exposures have continued at a constant rate, the objectives of this review were (1) to review the epidemiology of plant poisonings; and (2) to propose a rapid toxidromic classification system for highly toxic plant ingestions for field use by first responders in comparison to current classification systems. Internet search engines were queried to identify and select peer-reviewed articles on plant poisonings using the key words in order to classify plant poisonings into four specific toxidromes: cardiotoxic, neurotoxic, cytotoxic, and gastrointestinal-hepatotoxic. A simple toxidromic classification system of plant poisonings may permit rapid diagnoses of highly toxic versus less toxic and nontoxic plant ingestions both in households and outdoors; direct earlier management of potentially serious poisonings; and reduce costly inpatient evaluations for inconsequential plant ingestions. The current textbook classification schemes for plant poisonings were complex in comparison to the rapid classification system; and were based on chemical nomenclatures and pharmacological effects, and not on clearly presenting toxidromes. Validation of the rapid toxidromic classification system as compared to existing chemical classification systems for plant poisonings will require future adoption and implementation of the toxidromic system by its intended users.

Keywords: poisonous plants; poisonous herbs; intentional poisonings; unintentional poisonings; poisonous ingestions; poisonous foods

Index,

Editor(s): Azamal Husen, Mohammad Jawaid,

In Micro and Nano Technologies,

Nanomaterials for Agriculture and Forestry Applications,

Elsevier,

2020,

Pages 519-532,

ISBN 9780128178522,

https://doi.org/10.1016/B978-0-12-817852-2.00027-5.

(https://www.sciencedirect.com/science/article/pii/B9780128178522000275)

M. Barkaoui, A. Katiri, H. Boubaker, F. Msanda,

Ethnobotanical survey of medicinal plants used in the traditional treatment of diabetes in Chtouka Ait Baha and Tiznit (Western Anti-Atlas), Morocco,

Journal of Ethnopharmacology,

Volume 198,

2017,

Pages 338-350,

ISSN 0378-8741,

https://doi.org/10.1016/j.jep.2017.01.023.

(https://www.sciencedirect.com/science/article/pii/S0378874117301964)

Abstract: Ethnopharmacological relevance

In Morocco, diabetes mellitus is a major public health problem with more than 1.5 million cases in 2014. Medicinal plants are widely used by the Moroccan population to treat the illness.

Aim of the study

The aim of this work is to make an inventory of plant species used in folk medicine for the management of diabetes in Chtouka Ait Baha and Tiznit provinces.

Materials and methods

The survey was carried out by means of semi-structured questionnaires. A total of 380 interviews were conducted with traditional health practitioners and knowledgeable villagers. The data were analyzed through use value (UV), fidelity level (FL) and relative frequency of citation (RFC).

Results

In total, 48 plant species belonging to 25 families were reported. Lamiaceae, Asteraceae and Apiaceae were reported as the most represented families. Six plants are reported for the first time as used in traditional treatment of diabetes and one plant species was previously unknown for its medicinal use to treat diabetes in Morocco. The most frequently cited plant species are Allium sativum L., Salvia officinalis L., Marrubium vulgare L. and Lavandula dentata L. Leaves were the most cited plant part used, decoction is the preferred mode of preparation.

Conclusion

This study showed the importance of folk medicine in the healthcare system for the local people living in the study area. The current study represents a useful documentation, which can contribute to preserving knowledge on the use of medicinal plants in this region and to explore the phytochemical and pharmacological potential of medicinal plants.

Keywords: Ethnobotanical survey; Diabetes; Chtouka Ait Baha; Tiznit; Morocco

Muhammad Nauman Khan, Lal Badshah,

Floristic diversity and utility of flora of district Charsadda, Khyber Pakhtunkhwa,

Acta Ecologica Sinica,

Volume 39, Issue 4,

2019,

Pages 306-320,

ISSN 1872-2032,

https://doi.org/10.1016/j.chnaes.2018.10.003.

(https://www.sciencedirect.com/science/article/pii/S1872203218302373)

Abstract: The District Charsadda has a wide diversification with nature beauty and greenery. The area has rich biodiversity and natural resources. The District is geographically organized into two primary parts: Hashtnagar and Do Aaba. Research was carried out during 2017–18, in blooming season to collect different taxa from the area. The study depicted 253 taxa floristically with 189 genera and 71 families in which 4 families were pteridiophytes, 3 were gymnosperms and 64 were angiospermic families included 11 Monocot and 53 Dicot. The number of taxa per family varies from 1 to 56. Poaceae was the leading family in term of number of genera and species with 36 genera (19.04%) and 56 species (22.13%) followed by Asteraceae with 15 genera (7.936%) and 17 species (6.719%). The important groups in terms of species percentage were Dicots with a share of (53 species) followed by Monocots (11 species), Pteridophytes (5 species) and Gymnosperms (4 species). Herbaceous layer consists of 173 species (68.37%) followed by trees 46 species (18.18%), 25 species (9.881%) were shrub, 8 species (3.162%) were climbers and only 1 species (0.395%) was parasitic in the area. Biological spectrum depicted that Therophytes (126 species, 49.80%) and Microphyll (78 species, 30.83%) were the leading life form and leaf size classes. Ethnobotanical study proved that majority of species 164 were used as fodder/forage, 27 species were fruits, 28 species were vegetables, 28 species ornamental, 47 species fuel, 66 species were medicinals, 21 species used as timber, 8 species for thatching, 29 species for insect attractant and only 4 species used as spices (condiments) while some species have no local use in research area like Dryopteris stewartii, Equisetum arvense L., Marsilea L., Eichhornia crassipes (Mart.) Solms, and Potamogeton nodosus Poir. This is the first ever record of the area, no prior work exists in this regard. This work might help for future intensive and extensive researches.

Keywords: Floristic knowledge; Utility; Diversity; Life form; Leaf size; Charsadda

E. Valiakos, M. Marselos, N. Sakellaridis, Th. Constantinidis, H. Skaltsa,

Ethnopharmacological approach to the herbal medicines of the “Elements Alpha to Delta” in Nikolaos Myrepsos׳ Dynameron. Part II,

Journal of Ethnopharmacology,

Volume 205,

2017,

Pages 246-260,

ISSN 0378-8741,

https://doi.org/10.1016/j.jep.2017.04.021.

(https://www.sciencedirect.com/science/article/pii/S0378874117303185)

Abstract: Ethnopharmacological relevance

Dynameron is a Byzantine medical compendium, divided into 24 sections, the “Elements”, containing 2667 recipes, most of which inherited by previous physicians of the classic ancient Greek and Hellenistic, and imperial Roman periods.

Aim of the study

In continuation to our previous study concerning the first and largest chapter of the “Element Alpha” of Nikolaos Myrepsos׳ Dynameron (Valiakos et al., 2015), this paper focuses on the plants quoted in the recipes of the eight following chapters entitled “About Salts”, “About Honeypacks” and “About Spreads”, all belonging to the same “Element Alpha”; “About Antitussives” and “About Suppositories” belonging to the “Element Beta”; “About women's Cathartics” belonging to the “Element Gamma”; “About Drossaton” and “About Diachrisma”, both belonging to the “Element Delta”.

Materials and methods

Our main primary source material was the codex kept in the National Library of France (in Paris) under the number grec. 2243, which is the older and larger codex of Dynameron (Valiakos et al., 2015).

Results

The present study led us to the interpretation of 277 plants under different names, among which we recognized 57 medicinal plants listed by the European Medicines Agency, one of them with negative monograph (i.e. Chelidonium majus). In addition, there are identified taxa related to those quoted by EMA as herbal medicines. The plants appearing in the examined Elements belong to various families of which the most frequent are: Apiaceae 10.11%; Lamiaceae 7.22%; Asteraceae 6.86%; Rosaceae 6.5% and Fabaceae 6.14%.

Conclusions

A total of 277 species have been catalogued, most of which are referred in our previous publication (Valiakos et al., 2015). Among them, 56 plants still play a very important role in medical practice, as they are used as traditional herbal medicines (www.ema.eu). This evidence is a proof that the use of medicinal plants remains valuable from the ancient times until today. The recipes, in contrast to older medical compendia, contain precise measurements of ingredients and dosages for every drug, which seem to reflect empirical logic.

Keywords: Dynameron; “About Salts”; “About Honeypacks”; “About Spreads”; “About Antitussives”; “About Suppositories”; “About women's Cathartics”; “About Drossatōn”; “About Diachrisma”; Herbal medicines

Rebecca Clarke, Monica A. Kehoe, Sonya Broughton, Roger A.C. Jones,

Host plant affiliations of aphid vector species found in a remote tropical environment,

Virus Research,

Volume 281,

2020,

197934,

ISSN 0168-1702,

https://doi.org/10.1016/j.virusres.2020.197934.

(https://www.sciencedirect.com/science/article/pii/S0168170219308895)

Abstract: The Ord River Irrigation Area (ORIA) produces annual crops during the dry season (April to October), and perennial crops all-year-round, and is located in tropical northwestern Australia. Sandalwood plantations cover 50 % of the ORIA’s cropping area. Aphids cause major crop losses through transmission of viruses causing debilitating diseases and direct feeding damage. During 2016–2017, in both dry and wet seasons a total of 3320 leaf samples were collected from diverse types of sites on cultivated and uncultivated land and 1248 (38 %) of them were from aphid-colonized plants. In addition, aphids were found at 236 of 355 sampling sites. The 62 plant species sampled came from 23 families 19 of which contained aphid-colonized species. Aphid hosts included introduced weeds, Australian native plants, and volunteer or planted crop plants. Six aphid species were identified by light microscopy and CO1 gene sequencing, but there was no within species nucleotide sequence diversity. Aphis nerii, Hysteroneura setariae, Rhopalosiphum maidis and Schoutedenia ralumensis each colonized 1–3 plant species from a single plant family. A. craccivora colonized 14 species in five plant families. A. gossypii was the most polyphagous species colonizing 19 species in 11 plant families. A. gossypii, A. craccivora, A. nerii and S. ralumensis were found in both wet and dry seasons. Because of A. craccivora’s prevalence and high incidences on understory weeds and host trees, sandalwood plantations were important reservoirs for aphid spread to wild and crop plant hosts growing in cultivated and uncultivated land. Alternative hosts growing in rural bushland, irrigation channel banks, vacant or fallow land, and orchard plantation understories also constituted significant aphid reservoirs. This study provides new knowledge of the ecology of aphid vector species not only in the ORIA but also in tropical northern Australia generally. It represents one of relatively few investigations on aphid ecology in tropical environments worldwide.

Keywords: Aphids; Virus vectors; Landscape ecology; Hosts; Tropics; Seasons; Introduced weeds; Native plants; Crops; Sandalwood; Northwest Australia

P.J. Holloway, C.E. Jeffree,

Epicuticular Waxes,

Editor(s): Brian Thomas, Brian G Murray, Denis J Murphy,

Encyclopedia of Applied Plant Sciences (Second Edition),

Academic Press,

2017,

Pages 374-386,

ISBN 9780123948083,

https://doi.org/10.1016/B978-0-12-394807-6.00075-7.

(https://www.sciencedirect.com/science/article/pii/B9780123948076000757)

Abstract: Waxes are compounds or mixtures of organic substances that are insoluble in water and can form hard glossy non-wettable films on plant surfaces. Most are found in association with the cuticle that covers the aerial surfaces of land plants. Waxes are synthesized in epidermal cells and are secreted into or on to the cuticle. The chemical composition of waxes can vary between species and also between different surfaces in the same species. Waxes can be divided roughly between those containing predominantly long chain aliphatic compounds and those containing cyclic compounds, particularly triterpenoids. The physical form of waxes also varies, ranging from thin, amorphous films to complex layers of microcrystals. Waxes provide a hydrophobic covering to leaves and other plant parts that discourages the adhesion of dirt and other hydrophilic particles. There is some evidence that waxes may help protect plants from attack by certain pests and diseases. Some waxes have commercial importance, the best known being carnauba wax, which is one of the hardest waxes known.

Keywords: Aliphatic; carnauba; cuticle epicuticular; epidermis hydrophobic microcrystals polish secretion triterpenes water-repellant wax

Raphael Anue Mensah, Dan Li, Fan Liu, Na Tian, Xueli Sun, Xiangyang Hao, Zhongxiong Lai, Chunzhen Cheng,

Versatile Piriformospora indica and Its Potential Applications in Horticultural Crops,

Horticultural Plant Journal,

Volume 6, Issue 2,

2020,

Pages 111-121,

ISSN 2468-0141,

https://doi.org/10.1016/j.hpj.2020.01.002.

(https://www.sciencedirect.com/science/article/pii/S2468014120300029)

Abstract: Modern horticultural crop production systems target the exploitation of sustainable techniques for crop improvement while maintaining balance with the environment. Several beneficial microbes have been investigated and have yielded remarkable results. The endophytic fungus, Piriformospora indica, is one microbe group with the potential to offer numerous benefits and opportunities in modern horticultural crop production. This plant growth-promoting mycorrhizal fungus is of particular interest due to its beneficial implications in plant growth and development. The fungus has been experimentally proven to significantly improve water and nutrient/mineral absorption, early flowering, seed production/germination and plant photosynthetic capability, growth rates; especially in nutrient-deprived soils, alter the production of secondary metabolites, and promote adaptation, tolerance and/or resistance to biotic and abiotic stressors. Its unique capability to be axenically cultured makes it feasible for research and application. These attributes in combinations with its broad host range, offer immense potential for this fungus for research in horticultural plant improvement. Thus, this review highlights the potential applications of P. indica in horticultural crops research and production.

Keywords: Piriformospora indica; root endophyte; root colonization; stress response; plant growth and development

Cecilia M. Armas-Herrera, David Badía-Villas, Juan Luis Mora, Daniel Gómez,

Plant-topsoil relationships underlying subalpine grassland patchiness,

Science of The Total Environment,

Volume 712,

2020,

134483,

ISSN 0048-9697,

https://doi.org/10.1016/j.scitotenv.2019.134483.

(https://www.sciencedirect.com/science/article/pii/S0048969719344742)

Abstract: Approximately half of the area in the Spanish Central Pyrenees is dedicated to pastures. A decrease in stocking rate coupled with changes in livestock management in recent decades have favoured the expansion of Nardus grasslands, which are considered undesirable for grazing use and for diversity conservation. The objective of this study was to analyse how topsoil properties are related to grassland plant composition occurring in erosion-disturbed (chalk grasslands) and undisturbed (Nardus mat-grasslands) soils in a subalpine area of the Spanish Central Pyrenees. We selected six paired sampling points for a side-by-side comparison of both communities. At each point, we 1) estimated the plant cover of each species through inventories and 2) analysed a set of physical–chemical topsoil properties (0–5 and 5–10 cm depth). Data were analysed through multivariate analysis. We found typical species of Nardus mat-grasslands in the undisturbed sites growing on non-eroded and well-structured soils that were low in calcium and acidic, with high contents of organic matter. In turn, we found earlier-successional grassland communities growing on slopes recently affected by soil erosion processes. The species composition was mainly species from stony slope grasslands and, to a lesser extent, from the long-term snow-covered environments of the high mountains. These soils were shallower and stonier and had a less-stable structure, higher pH, and lower organic matter and calcium content than undisturbed soils. Our results suggest that the differences between both communities emerge and are maintained by soil–plant feedback mechanisms mediated in Nardus mat-grasslands through soil stabilization and acidification and in chalk grasslands through soil erosion and basification. These findings suggest that the subalpine grassland mosaic results from a model of non-equilibrium plant coexistence due to soil disturbance and inexorable succession. Management should be focused on maintaining a disturbance regime, through grazing, sufficient to prevent the spreading of Nardus mat-grasslands.

Keywords: Nardus mat-grasslands; Chalk grasslands; Grazed landscapes; Soil properties; Soil erosion; Plant succession

Zahra Noohpisheh, Hamzeh Amiri, Saeed Farhadi, Abdolnaser Mohammadi-gholami,

Green synthesis of Ag-ZnO nanocomposites using Trigonella foenum-graecum leaf extract and their antibacterial, antifungal, antioxidant and photocatalytic properties,

Spectrochimica Acta Part A: Molecular and Biomolecular Spectroscopy,

Volume 240,

2020,

118595,

ISSN 1386-1425,

https://doi.org/10.1016/j.saa.2020.118595.

(https://www.sciencedirect.com/science/article/pii/S1386142520305746)

Abstract: In the present study, biological synthesis of Ag-ZnO nanocomposites was performed using hydroalcoholic extract of fenugreek leaves. Metal/semiconductor oxide nanocomposites are excellent owing to their optical, electrical, magnetic, and chemical properties that are not detected in single individual constituents. The synthesized Ag-ZnO nanocomposites were investigated through the use of methods such as FTIR, UV vis DRS, SEM-EDX, TEM, XRD, zeta potential analysis, and DLS. The synthesized Ag-ZnO nanocomposites had an average particle size of about 75 nm and a zeta potential of −37.5 mV. The XRD results confirmed that Ag was successfully introduced into the Ag-ZnO nanocomposites via a hydrothermal method. The antimicrobial and antifungal activities of Ag-ZnO nanocomposites were evaluated by agar well diffusion method against three microbial and fungal strains; it was found that the Ag-ZnO nanocomposites were toxic against all the tested microbial and fungal strains. Ag-ZnO nanocomposites was observed to have significant antioxidant activity against DPPH (2,2-diphenyl-1-picrylhydrazyl) free radicals. The Ag-ZnO nanocomposites exhibited excellent photocatalytic activity and stability against the degradation of malachite green under visible light irradiation. The study successfully applied a simple and eco-friendly method for synthesizing efficient multifunctional Ag-ZnO nanocomposites using green synthetic approach.

Keywords: Ag-ZnO nanocomposites; Trigonella foenum-graecum; Leaf extract; Antibacterial activity; Antifungal activity; Photocatalytic property

Jing An, Dingjun Hao, Qian Zhang, Bo Chen, Rui Zhang, Yi Wang, Hao Yang,

Natural products for treatment of bone erosive diseases: The effects and mechanisms on inhibiting osteoclastogenesis and bone resorption,

International Immunopharmacology,

Volume 36,

2016,

Pages 118-131,

ISSN 1567-5769,

https://doi.org/10.1016/j.intimp.2016.04.024.

(https://www.sciencedirect.com/science/article/pii/S1567576916301588)

Abstract: Excessive bone resorption plays a central role on the development of bone erosive diseases, including osteoporosis, rheumatoid arthritis, and periodontitis. Osteoclasts, bone-resorbing multinucleated cells, are differentiated from hemopoietic progenitors of the monocyte/macrophage lineage. Regulation of osteoclast differentiation is considered an effective therapeutic target to the treatment of pathological bone loss. Natural plant-derived products, with potential therapeutic and preventive activities against bone-lytic diseases, have received increasing attention in recent years because of their whole regulative effects and specific pharmacological activities, which are more suitable for long-term use than chemically synthesized medicines. In this review, we summarized the detailed research progress on the active compounds derived from medical plants with potential anti-resorptive effects and their molecular mechanisms on inhibiting osteoclast formation and function. The active ingredients derived from natural plants that are efficacious in suppressing osteoclastogenesis and bone resorption include flavonoids, terpenoids (sesquiterpenoids, diterpenoids, triterpenoids), glycosides, lignans, coumarins, alkaloids, polyphenols, limonoids, quinones and others (steroid, oxoxishhone, fatty acid). Studies have shown that above natural products exert the inhibitory effects via regulating many factors involved in the process of osteoclast differentiation and bone resorption, including the essential cytokines (RANKL, M-CSF), transcription factors (NFATc1, c-Fos), signaling pathways (NF-κB, MAPKs, Src/PI3K/Akt, the calcium ion signaling), osteoclast-specific genes (TRAP, CTSK, MMP-9, integrin β3, OSCAR, DC-STAMP, Atp6v0d2) and local factors (ROS, LPS, NO). The development of osteoclast-targeting natural products is of great value for the prevention or treatment of bone diseases and for bone regenerative medicine.

Keywords: Natural products; Medical plants; Osteoclastogenesis; Bone resorption; Molecular mechanism

Javad Sharifi-Rad, Bahare Salehi, Zorica Z. Stojanović-Radić, Patrick Valere Tsouh Fokou, Marzieh Sharifi-Rad, Gail B. Mahady, Majid Sharifi-Rad, Mohammad-Reza Masjedi, Temitope O. Lawal, Seyed Abdulmajid Ayatollahi, Javid Masjedi, Razieh Sharifi-Rad, William N. Setzer, Mehdi Sharifi-Rad, Farzad Kobarfard, Atta-ur Rahman, Muhammad Iqbal Choudhary, Athar Ata, Marcello Iriti,

Medicinal plants used in the treatment of tuberculosis - Ethnobotanical and ethnopharmacological approaches,

Biotechnology Advances,

Volume 44,

2020,

107629,

ISSN 0734-9750,

https://doi.org/10.1016/j.biotechadv.2020.107629.

(https://www.sciencedirect.com/science/article/pii/S0734975020301312)

Abstract: Tuberculosis is a highly infectious disease declared a global health emergency by the World Health Organization, with approximately one third of the world's population being latently infected with Mycobacterium tuberculosis. Tuberculosis treatment consists in an intensive phase and a continuation phase. Unfortunately, the appearance of multi drug-resistant tuberculosis, mainly due to low adherence to prescribed therapies or inefficient healthcare structures, requires at least 20 months of treatment with second-line, more toxic and less efficient drugs, i.e., capreomycin, kanamycin, amikacin and fluoroquinolones. Therefore, there exists an urgent need for discovery and development of new drugs to reduce the global burden of this disease, including the multi-drug-resistant tuberculosis. To this end, many plant species, as well as marine organisms and fungi have been and continue to be used in various traditional healing systems around the world to treat tuberculosis, thus representing a nearly unlimited source of active ingredients. Besides their antimycobacterial activity, natural products can be useful in adjuvant therapy to improve the efficacy of conventional antimycobacterial therapies, to decrease their adverse effects and to reverse mycobacterial multi-drug resistance due to the genetic plasticity and environmental adaptability of Mycobacterium. However, even if some natural products have still been investigated in preclinical and clinical studies, the validation of their efficacy and safety as antituberculosis agents is far from being reached, and, therefore, according to an evidence-based approach, more high-level randomized clinical trials are urgently needed.

Keywords: Mycobacterium; Multi drug-resistance; Traditional healing systems; Herbal medicine; Antimycobacterial agents; Evidence-based medicine

Ana H. Ladio, Marina Acosta,

Urban medicinal plant use: Do migrant and non-migrant populations have similar hybridisation processes?,

Journal of Ethnopharmacology,

Volume 234,

2019,

Pages 290-305,

ISSN 0378-8741,

https://doi.org/10.1016/j.jep.2019.01.013.

(https://www.sciencedirect.com/science/article/pii/S0378874118328903)

Abstract: Ethnopharmacological relevance

Urban migrant herbal medicine is an important topic on a global scale. Through bibliographical analysis of published studies we can get an overview of the different hybridisation processes at work in cities around the world, the main medicinal plants used and the principal ailments treated. We analysed the differential characteristics of urban ethnobotanical studies involving transnational migrant and non-migrant populations, in order to contribute useful information for the design of public health policies.

Materials and methods

A systematic and integrative revision was conducted, leading to a final selection of 66 primary sources, including studies with and without immigrants. In both cases, richness (S), considered as the sum of all species cited in the work, botanical families and reported ailments were recorded. Based on the work of Ladio and Albuquerque (2014) the main hybridisation processes identifiable in the literature were assessed. These were: fusion, relocation, re-combination of different species, their restructuring as medicinal targets, spatial segregation in usage, innovations found in the circulation and consumption of the plants, and the presence of simultaneous coexistence of different symbolic universes in plant medical practices.

Data Analysis

This was qualitative and quantitative, including both in-depth interpretative content analysis of the studies and frequency analysis of numerical data, such as species richness, botanical families, ailments and the hybridisation processes detected. A multinomial logistic regression model was used to analyse whether the probability of medicinal plant hybridisation processes occurring was the same in literature with and without migrants.

Results

A total of 522 medicinal species formed part of the main urban ethnobotany worldwide; the majority were cosmopolitan in distribution and belonged to the Asteriaceae and Lamiaceae families. Only 21% of these species appeared in both migrant and non-migrant studies. Most were used for gastrointestinal and hepatic ailments. Surprisingly, culture-related illnesses were not frequently mentioned, probably due to lack of recognition and re-interpretation by authors. Logistic analysis showed that in the studies with migrants, relocation and restructuring of plant use were the most frequently identified processes, while in the studies involving only non-migrants fusion was 4 times more likely to be found than in studies with migrants.

Conclusions

Our research on hybridisation processes shows that cities constitute an environment that fosters a rapid exchange of practices and knowledge about the available species. Studies with migrants have shown that they reproduce traditional models in their use of plants, and so relocation and restructuring of their herbal medicine are the principal processes. Health risks in this case are related to the difficulties faced by these groups in obtaining their plants and reproducing their practices. In the case of fusion processes observed in non-migrants, who do not normally have a long history or much experience of plant use, errors or poisoning may result from misuse. This information highlights the importance of considering these processes in health policies, particularly when there are no significant quality controls of these resources.

Keywords: Urban society; Urban ethnobotany; Innovations; Fusion, change

Muhammad Zakariyyah Aumeeruddy, Mohamad Fawzi Mahomoodally,

Traditional herbal therapies for hypertension: A systematic review of global ethnobotanical field studies,

South African Journal of Botany,

Volume 135,

2020,

Pages 451-464,

ISSN 0254-6299,

https://doi.org/10.1016/j.sajb.2020.09.008.

(https://www.sciencedirect.com/science/article/pii/S0254629920310644)

Abstract: The worldwide burden of hypertension and its associated cardiovascular diseases have urged researchers to explore for alternative therapies. Together with laboratory studies, field surveys on the documentation of traditional antihypertensive therapies have expanded across the world. However, a compilation and analysis of these field studies has not been done so far. This review aim to document all traditional medicinal plants used globally for the management of hypertension. Field studies were retrieved from main databases including Sciencedirect, Medline/PubMed, and Google Scholar. 433 surveys were obtained which dated from 1970 to 2019. A total of 1329 traditionally used plant species (823 genera and 176 families) were reported across 90 countries against hypertension. Compositae (Genera: 68; Species:103) and Lamiaceae (Genera:39; Species:103) were the families with the most used species. The main species (in order of number of reports) were Allium sativum L., followed by Olea europaea L., Allium cepa L., Annona muricata L., Persea americana Mill., Citrus aurantiifolia (Christm.) Swingle, Catharanthus roseus (L.) G.Don, Moringa oleifera Lam., Cymbopogon citratus (DC.) Stapf, and Carica papaya L. Pakistan (Family:81; Species:246) reported the highest number of plant species followed by Mexico, Nigeria, India, Algeria, Morocco, Thailand, Suriname, Benin, and Iran. Leaf (35%), fruit (12%), and root (10%) were the most preferred plant parts while the main methods of preparation were decoction (50%) and infusion (22%). This study is the first of its kind to compile all traditionally used hypotensive medicinal plants across the world. It is recommended that another systematic review be conducted on the biological properties of all identified species in our review to identify those which have been scientifically validated in in vitro, in vivo, and clinical studies before they can be considered as alternative or complementary antihypertensive therapies.

Keywords: Cardiovascular diseases; Ethnomedicine; Hypertension; Hypotensive; Medicinal plants; Traditional

Elhassan Idm'hand, Fouad Msanda, Khalil Cherifi,

Ethnobotanical study and biodiversity of medicinal plants used in the Tarfaya Province, Morocco,

Acta Ecologica Sinica,

Volume 40, Issue 2,

2020,

Pages 134-144,

ISSN 1872-2032,

https://doi.org/10.1016/j.chnaes.2020.01.002.

(https://www.sciencedirect.com/science/article/pii/S1872203218302385)

Abstract: Background

This study was carried out among the inhabitants of the province of Tarfaya (Moroccan Center South), in order to make an inventory of the medicinal plants used in traditional herbal medicine by the local population.

Methods

Information was obtained by means of open interviews with local people using the questionnaires. The data was analyzed using Use Value (UV), Relative Frequency of Citation (RFC), Fidelity Level (FL) and Informant Consensus Factor (ICF).

Results

The analysis of the results allowed us to identify 130 vascular plant species in 57 families with a significant representativeness of Lamiaceae (10%), Asteraceae (9.23%), Fabaceae (8.46%), Apiaceae (6.15%), Poaceae (3.85%), Solanaceae (3.07%) and Amaranthaceae (3.07%). These species are mainly used in the care of the digestive and genito-urinary disorders. The UV ranged from 0.01 (Aframomum melegueta) to 0.34 (Maerua crassifolia). The RFC ranged from 0.01 (Aframomum melegueta) to 0.32 (Maerua crassifolia). The highest FL (100%) was found for 38 species, while the highest values of ICF were recorded for gastrointestinal pains (0.972).

Conclusion

This study revealed rich ethnomedicinal knowledge in the Tarfaya province. Furthermore, ethnobotanical analysis will provide data for further pharmacological studies.

Keywords: Medicinal plants; Ethnobotanical; Fidelity level; Informant consensus factor; Morocco

Ammaiyappan Selvam, Ayyamperumal Mahadevan,

Distribution of mycorrhizas in an abandoned fly ash pond and mined sites of Neyveli Lignite Corporation, Tamil Nadu, India,

Basic and Applied Ecology,

Volume 3, Issue 3,

2002,

Pages 277-284,

ISSN 1439-1791,

https://doi.org/10.1078/1439-1791-00107.

(https://www.sciencedirect.com/science/article/pii/S1439179104700857)

Abstract: Summary

Presence of arbuscular mycorrhizal (AM) species was surveyed in an abandoned lignite fly ash pond, overburden dumps and reclaimed overburden dumps. In ash pond, 15 AM (Acaulospora gerdemannii, Gigaspora decipiens, Gi. gigantea, Gi. margarita, Glomus citricola, Gl. fasciculatum, Gl. formosanum, Gl. fulvum, Gl. maculosum, Gl. magnicaule, Gl. mosseae, Gl. tenebrosum, Sclerocystis pachycaulis, Scutellospora erythropa and Sc. fulgida) were isolated. From overburden and reclaimed overburden dumps, 4 (Gl. fasciculatum, Gl. mosseae, Sclerocystis microcarpus, and Scutellospora verrucosa) and 13 AM fungal species (Acaulospora gerdemannii, Entrophospora colombiana, Gigaspora gigantea, Gi. margarita, Glomus fasciculatum, Gl. macrocarpum, Gl. mosseae, Gl. vesiculiferum, Sclerocystis pachycaulis, S. sinuosa, Scutellospora coralloidea, Sc. erythropa and Sc. persica) were isolated, respectively. In all the sites, Glomus mosseae was the dominant AM fungus. From Acacia auriculiformis plantation in the overburden dumps, fruit bodies of ectomycorrhizal fungus Pisolithus tinctorius were recorded. Soil pH, electrical conductivity, organic carbon and N, P, K content of rhizosphere soils were analyzed to find their influence on mycorrhizal colonization. In einem aufgegebenen Braunkohle-Flugasche-Teich, in Abraumhalden und rekultivierten Abraumhalden wurde das Vorkommen arbuskulärer Mykorrhiza (AM) untersucht. Im Asche-Teich wurden 14 Arten AM isoliert (Acaulospora gerdemannii, Gigaspora decipiens, Gi. gigantea, Gi. margarita, Glomus citricola, Gl. fasciculatum, Gl. formosanum, Gl. fulvum, Gl. maculosum, Gl. magnicaule, Gl. mosseae, Gl. tenebrosum, Sclerocystis pachycaulis, Scutellospora erythropa und Sc. fulgida). Aus den Abraumhalden wurden 4 Arten (Gl. fasciculatum, Gl. mosseae, Sclerocystis microcarpus und Scutellospora verrucosa) und aus den rekultivierten Abraumhalden 13 AM-Pilzarten isoliert (Acaulospora gerdemannii, Entrophospora colombiana, Gigaspora gigantea, Gi. margarita, Glomus fasciculatum, Gl. macrocarpum, Gl. mosseae, Gl. vesiculiferum, Sclerocystis pachycaudalis, S. sinuosa, Scutellospora coralloidea, Sc. erythropa und Sc. persica). An allen Probeorten war Glomus mosseae der dominante AM-Pilz. In den Acacia auriculiformis-Pflanzungen auf den Abraumhalden wurden Fruchtkörper des ektomykorrhizalen Pilzes Pisolithus tinctorius gefunden. Der Boden-pH, die elektrische Leitfähigkeit, der organische Kohlenstoff und die N-, P- und K-Gehalte des Rhizosphären-Bodenswurden analysiert, um ihren Einfluss auf die Besiedlung durch Mykorrhiza zu finden.

Keywords: Disturbed soils; AM fungi; Glomus; Fly ash; Ectomycorrhiza

Mira Syahfriena Amir Rawa, Zurina Hassan, Vikneswaran Murugaiyah, Toshihiko Nogawa, Habibah A. Wahab,

Anti-cholinesterase potential of diverse botanical families from Malaysia: Evaluation of crude extracts and fractions from liquid-liquid extraction and acid-base fractionation,

Journal of Ethnopharmacology,

Volume 245,

2019,

112160,

ISSN 0378-8741,

https://doi.org/10.1016/j.jep.2019.112160.

(https://www.sciencedirect.com/science/article/pii/S0378874119320847)

Abstract: Ethnopharmacological relevance

Enhancement of cholinergic functions in the brain via acetylcholinesterase inhibition is one of the main therapeutic strategies to improve symptoms associated with Alzheimer's or related cognitive deficits. There is a pathophysiological correlation between Alzheimer's and Diabetes Mellitus, as well as inflammation and oxidative stress that may cause cognitive decline.

Aim of the study

The present study was intended to evaluate anti-cholinesterase potential of 177 Malaysian plant extracts from 148 species known to have related ethnomedicinal uses such as anti-inflammatory, anti-oxidant, anti-diabetic, epilepsy, headache, memory enhancement and anti-aging.

Materials and methods

Anti-cholinesterase screening against both acetylcholinesterase (AChE) and butyrylcholinesterase (BChE) enzymes was performed on the basis of in-vitro colorimetric 96-well microplate-based assay method. Potent active plant extracts were subjected to liquid-liquid extraction and acid-base fractionation for further analysis.

Results

Fifty-seven plant extracts exhibited potent anti-cholinesterase activities (50–100% inhibition) at 200 μg/ml. Majority of the active plants originated from Fabaceae family. Coccoloba uvifera (L.) L. stem extract manifested the lowest IC50 of 3.78 μg/ml for AChE and 5.94 μg/ml for BChE. A few native species including Tetracera indica (Christm. & Panz.) Merr., Cyrtostachys renda Blume and Ixora javanica (Blume) DC. showed cholinesterase inhibition despite limited local medical applications. Further anti-AChE evaluation (50 μg/ml) of 18 potent plant extracts harbored active polar components in butanol and water fractions, except Senna pendula (Willd.) H.S.Irwin & Barneby (leaves and stems), Acacia auriculiformis Benth. (leaves), Artocarpus altilis (Parkinson ex F.A.Zorn) Fosberg (leaves), and Macaranga tanarius (L.) Mull.Arg. (leaves) that showed inhibitory activity in less polar fractions. The acidic extraction of these four plant species improved their inhibition level against AChE.

Conclusion

This study rendered a preliminary overview of anti-cholinesterase activity from diverse Malaysian botanical families in which provided the medical relevance toward these native plant species, especially ones with limited ethnobotanical record or practice.

Keywords: Anti-cholinesterase; Acetylcholinesterase; Butyrylcholinesterase; In-vitro; Plant extracts

Balan Banumathi, Baskaralingam Vaseeharan, Balasubramanian Malaikozhundan, Palaniappan Ramasamy, Marimuthu Govindarajan, Naiyf S. Alharbi, Shine Kadaikunnan, Angelo Canale, Giovanni Benelli,

Green larvicides against blowflies, Lucilia sericata (Diptera, Calliphoridae): Screening of seven plants used in Indian ethno-veterinary medicine and production of green-coated zinc oxide nanoparticles,

Physiological and Molecular Plant Pathology,

Volume 101,

2018,

Pages 214-218,

ISSN 0885-5765,

https://doi.org/10.1016/j.pmpp.2017.02.003.

(https://www.sciencedirect.com/science/article/pii/S0885576517300425)

Abstract: Dipteran flies are responsible of myiasis, a common parasitic infestation leading to the invasion of living, necrotic or dead tissues. They also transmit mycobacterial infections to livestock. In the present investigation, seven plant species employed in Southern India for medical and ethno-veterinary purposes were extracted using ethanol or water and tested against second instar larvae of Lucilia sericata. 100% larval mortality was observed testing Lobelia leschenaultiana ethanol extract at 60 mg/L (LC50 = 3.4 mg/L). Based on the highest percentage of larval mortality, we selected L. leschenaultiana for fabrication of ZnO nanoparticles to control L. sericata. Zinc acetate showed 82.2% mortality at 60 mg/L (LC50 = 38.2 mg/L) whereas L. leschenaultiana-coated ZnO nanoparticles (Ll-ZnO NPs) showed 100% mortality at 6 mg/L, the LC50 was 0.78 mg/L. Overall, our research represents an attempt to integrate current ethno-veterinary knowledge from native tribes of Southern India, in order to identify effective botanicals for the development of nano-biopesticides against livestock pests and parasites.

Keywords: Biosafety; Diptera; Ethnopharmacology; Green synthesis; Myiasis; Nilgiri

Volume 75 (2004),

Fitoterapia,

Volume 76, Issue 1,

2005,

Pages 134-141,

ISSN 0367-326X,

https://doi.org/10.1016/j.fitote.2004.11.001.

(https://www.sciencedirect.com/science/article/pii/S0367326X04002497)

A.M. Baldé, M.S. Traoré, M.A. Baldé, M.S. Barry, A. Diallo, M. Camara, S. Traoré, M. Kouyaté, S. Traoré, S. Ouo-Ouo, A.L. Myanthé, N. Keita, N.L. Haba, K. Goumou, F. Bah, A. Camara, M.S.T. Diallo, M. Sylla, E.S. Baldé, S. Diané, L. Pieters, K. Oularé,

Ethnomedical and ethnobotanical investigations on the response capacities of Guinean traditional health practioners in the management of outbreaks of infectious diseases: The case of the Ebola virus epidemic,

Journal of Ethnopharmacology,

Volume 182,

2016,

Pages 137-149,

ISSN 0378-8741,

https://doi.org/10.1016/j.jep.2016.02.021.

(https://www.sciencedirect.com/science/article/pii/S0378874116300654)

Abstract: Ethnopharmacological relevance

The recent outbreak of Ebola virus infections has mostly remained confined to the West African countries Guinea-Conakry, Sierra-Leone and Liberia. Due to intense national and international mobilizations, a significant reduction in Ebola virus transmission has been recorded. While international efforts focus on new vaccines, medicines and diagnostics, no coherent national or international approach exists to integrate the potential of the traditional health practitioners (THPs) in the management of infectious diseases epidemics. Nevertheless, the first contact of most of the Ebola infected patients is with the THPs since the symptoms are similar to those of common traditionally treated diseases or symptoms such as malaria, hemorrhagic syndrome, typhoid or other gastrointestinal diseases, fever and vomiting.

Materials and methods

In an ethnomedical survey conducted in the 4 main Guinean regions contacts were established with a total of 113 THPs. The socio-demographic characteristics, the professional status and the traditional perception of Ebola Virus Disease (EVD) were recorded.

Results

The traditional treatment of the main symptoms was based on 47 vegetal recipes which were focused on the treatment of diarrhea (22 recipes), fever (22 recipes), vomiting (2 recipes), external antiseptic (2 recipes), hemorrhagic syndrome (2 recipes), convulsion and dysentery (one recipe each). An ethnobotanical survey led to the collection of 54 plant species from which 44 identified belonging to 26 families. The most represented families were Euphorbiaceae, Caesalpiniaceae and Rubiaceae. Literature data on the twelve most cited plant species tends to corroborate their traditional use and to highlight their pharmacological potential.

Conclusions

It is worth to document all available knowledge on the traditional management of EVD-like symptoms in order to evaluate systematically the anti-Ebola potential of Guinean plant species.

Keywords: Ebola virus disease; Traditional health practitioners; Guinea-Conakry

Paolo Maria Guarrera, Giovanni Salerno, Giulia Caneva,

Folk phytotherapeutical plants from Maratea area (Basilicata, Italy),

Journal of Ethnopharmacology,

Volume 99, Issue 3,

2005,

Pages 367-378,

ISSN 0378-8741,

https://doi.org/10.1016/j.jep.2005.01.039.

(https://www.sciencedirect.com/science/article/pii/S0378874105000887)

Abstract: Field ethnobotanical survey was undertaken for the period of 2002–2003 in the Tyrrhenian part of the Basilicata region of southern Italy. Data of 56 species of plants belonging to 29 families where gathered through interviews; among the species, 47 are used in human therapy, 6 as insect repellents, 15 in veterinary medicine, 1 for its ichthyotoxic properties and 3 for magic therapeutic purposes. The most important findings in ethnomedicine relate to Nasturtium officinale (renal colic, liver diseases), Foeniculum vulgare subsp. piperitum (mouth ulcers), Leopoldia comosa (toothache, headache), Micromeria graeca subsp. graeca (coughs) and Ceterach officinarum (malaria), while in the ethnoveterinary field, we have Pteridium aquilinum (wolf bites) and Spartium junceum (fractures of animal limbs).

Keywords: Ethnobotany; Antiparasitic plants; Medicinal plants; Veterinary plants; Basilicata

Frederick W. Fraunfelder,

Part 6 - Herbal medicines and dietary supplements – an overview,

Editor(s): Frederick “Fritz” T. Fraunfelder, Frederick “Rick” W. Fraunfelder, Wiley A. Chambers, Bree Jensvold-Vetsch,

Drug-Induced Ocular Side Effects (Seventh Edition),

W.B. Saunders,

2015,

Pages 43-45,

ISBN 9780323319843,

https://doi.org/10.1016/B978-0-323-31984-3.00006-4.

(https://www.sciencedirect.com/science/article/pii/B9780323319843000064)

M.M.P. Mogale, D.C. Raimondo, B.-E. VanWyk,

The ethnobotany of Central Sekhukhuneland, South Africa,

South African Journal of Botany,

Volume 122,

2019,

Pages 90-119,

ISSN 0254-6299,

https://doi.org/10.1016/j.sajb.2019.01.001.

(https://www.sciencedirect.com/science/article/pii/S0254629918320064)

Abstract: The ethnobotany of Sekhukhuneland and the plants used by rural Bapedi people for their everyday needs have not yet been systematically recorded. Available information is mostly focussed on medicinal plants that are used by traditional healers. The aim of this study was to accurately record extant indigenous knowledge on the most important useful plants within Central Sekhukhuneland. The study was conducted from 2015 to 2017 in three rural villages: Frisgewaght, Ga-Moretsele/Tsehlwaneng and Ga-Sekhele. A total of 27 participants of different age groups were interviewed using the matrix method and a flip-file of composite photographs of 152 local useful plants. Sixty-six species (44%) had food uses, 71 (46%) had medicinal uses and 62 (40%) had various craft uses. A total 185 use-records (107 medicinal, 21 food and 57 other) and 98 vernacular names were newly recorded at the time the study was completed. We used the Species Popularity Index (SPI) to quantify the relative importance of the species in the three communities, as well as the Ethnobotanical Knowledge Index (EKI) to assess the level of indigenous knowledge amongst the participants. The study revealed that a rich local culture of everyday plant uses that have not yet been systematically recorded.

Keywords: Bapedi; Indigenous knowledge; Inventory; Matrix method; Useful plants; Quantitative ethnobotany; Traditional uses

Apsara Wijenayake, Amarasooriya Pitawala, Ratnayake Bandara, Charmalie Abayasekara,

The role of herbometallic preparations in traditional medicine – A review on mica drug processing and pharmaceutical applications,

Journal of Ethnopharmacology,

Volume 155, Issue 2,

2014,

Pages 1001-1010,

ISSN 0378-8741,

https://doi.org/10.1016/j.jep.2014.06.051.

(https://www.sciencedirect.com/science/article/pii/S0378874114004991)

Abstract: Ethnopharmocological relevance

Biotite mica enriched with Fe2+ ions are widely used as a major mineral ingredient in traditional pharmaceutical science of alchemy (Rasashastra). Abhrak bhasma (mica ash), a pharmaceutical product containing treated mica, is utilized, for example, in Ayurvedic treatments for ailments such as gastritis, renal disease, skin disease and mainly in rejuvenation formulations. However, the untreated mica minerals may be harmful when used directly, as they carry considerably high amounts of trace-elements that can cause undesirable effects in the human body. In order to remove toxic factors and produce readily absorbable materials having high nutrient capacity, specific thermal and chemical treatments (purification, detoxification, particle size reduction and incineration) are performed during the preparation of Rasashastra. This review evaluates the chemical and pharmacological aspects of mica ash as well as the technological aspects of mica ash production.

Materials and methods

The detailed literature review on the chemistry and scientific basis of mica ash, its preparation techniques, mica alterations and pharmaceutical applications was carried out by using published Ayurvedic text books and research articles, available from Science Direct, on mica minerals, mica ash and their physico-chemical alteration processes and pharmacological applications.

Results

During the purification and detoxification procedures, heating followed by quenching (in ionic medium) influences the structural distortion and the development of stress-induced cracks and spallations of the micaceous plates. Thus, the efficient diffusion of the external medium takes place at successive heating and quenching steps. Acidic organic liquids and animal byproducts can enhance the cation exchange capacity and solubility of mica. Further, these natural compounds facilitate the removal of toxic-elements in the structure. When treated-mica and paddy husks are tied up in a cloth and squeezed, particle size reduction and further detoxification takes place. Leaching out of oxidized iron coatings is accelerated when the mixtures are immersed in acidic media, by which the filtrate is enriched with oxidized iron-silicate particles. These nano-oxide particles are converted into a more favorable oxidation form for human consumption when the herbometallic mixture is incinerated in closed vessels. Recent analytical data reveals that major and minor elements in mica ash are within the limits of pharmacopoeial standards for Ayurvedic formulations. Further, recent studies show that mica ash has hypoglycemic, hepatoprotective, anthelminthic and antimicrobial properties.

Conclusions

Chemical and structural modifications in mica occur during mica-based drug preparation in traditional medicine. Purification steps particularly influence the structural distortion while heating and quenching can form nano-size particles. Carboxylic acids and other organic molecules present in quenching media serve as chemical modifiers of mica. At the same time the toxic elements are leached out from mica to the quenching media through an ion exchange process. Mica ash has been successfully used for treating liver, kidney and skin related ailments in traditional medicine, and mica ash alone or its herbo-metallic formulations have different applications. Further, the recent toxicological and analytical studies validate the traditional uses of mica ash and mica ash bearing products. Further scientific studies are needed to fully establish that mica-based pharmaceuticals are safe and devoid of toxic and long term side effects.

Keywords: Rasashastra; Detoxification; Herbometallic; Bhasma; Biotite; Mica ash

S. Suroowan, K.B. Pynee, M.F. Mahomoodally,

A comprehensive review of ethnopharmacologically important medicinal plant species from Mauritius,

South African Journal of Botany,

Volume 122,

2019,

Pages 189-213,

ISSN 0254-6299,

https://doi.org/10.1016/j.sajb.2019.03.024.

(https://www.sciencedirect.com/science/article/pii/S0254629918311116)

Abstract: Bestowed with a rich floral diversity and singularity, the tropical island of Mauritius is home to several exotic and endemic plant species. Since the first settlement of man over the island more than 300 years ago, the local inhabitants have been in proximity with nature and have exploited plants as a major source of medicine to assuage suffering emanating from a wide range of minor to chronic ailment conditions. Over the years, sufficient experience surrounding the medicinal use of plant species has been gathered by the local inhabitants through trial and error as well as sharing of traditional knowledge from one generation to the other. Such valuable knowledge has been preserved since the first documentation in 1864. Nonetheless, there is no single compilation of plant species employed since the first documentation. In addition, no comparative study has been conducted to highlight plant species which are still being employed extensively. This review therefore endeavors to document medicinal plants reported since the first establishment of man over the island alongside highlighting plant species deserving due attention regarding the evaluation of their pharmacological potential. Following a comprehensive data mining, 561 plant species were found to have been used and/or still being used for the prophylaxis, management and/or cure of an innumerable number of human ailment conditions. Interestingly, the traditional uses of plant species such as Gomphocarpus fruticosus (L.) W.T. Aiton, Gomphocarpus physocarpus E. Mey, Paederia foetida L., Ravenala madagascariensis Sonn., and Wikstroemia indica (L.) C.A. Mey. have been maintained over the years with noticeable use value (UV). In furtherance, other plant species employed locally (Launaea sarmentosa (Willd.) Sch.Bip. ex Kuntze, Grangeria borbonica Lam., Adiantum rhizophorum Schrad., Antirhea borbonica J.F.Gmel., Ageratina riparia (Regel) R.M. King et H. Robinson, Cnestis glabra Lam., Artemisia verlotiorum Lamotte and Aleurites fordii Hemsl.) also deserve to be evaluated pharmacologically by the scientific community. Similarly, numerous endemic and indigenous plant species (Agarista salicifolia (Lam.) G. Don, Asparagus umbellulatus Bresler, Jumellea fragrans (Thouars) Schltr, Gymnosporia pyria (Willemet) Jordaan, Mimusops maxima (Poiret) Vaughan, Tambourissa quadrifida Sonnerat and Pittosporum senacia Putt. subsp. senacia) are potential candidates for future in vitro, in vivo and in silico studies. Notably, studies focusing on the safety profile of medicinal plants is also warranted to minimize the risk of side effects, adverse events as well as the occurrence of herb–drug interactions among local inhabitants.

Keywords: Ethnobotany; Dissemination; Endangered species; Drug discovery; Safety

Ibrahim Babangida Abubakar, Angela Nnenna Ukwuani-Kwaja, Folami Sulaimon Olayiwola, Ibrahim Malami, Aliyu Muhammad, Sanusi Jega Ahmed, Quadri Olaide Nurudeen, Mansurat Bolanle Falana,

An inventory of medicinal plants used for treatment of cancer in Kwara and Lagos state, Nigeria,

European Journal of Integrative Medicine,

Volume 34,

2020,

101062,

ISSN 1876-3820,

https://doi.org/10.1016/j.eujim.2020.101062.

(https://www.sciencedirect.com/science/article/pii/S1876382019312387)

Abstract: Introduction

The southwestern and northcentral parts of Nigeria are highly endowed with medicinal plants that have been used for decades to treat cancer. However, these herbal recipes and traditional medicinal practices have been scarcely investigated and documented. New uninvestigated plants could serve as potential sources for novel cytotoxic agents. This study was aimed at documenting the traditional medicinal practices used for treating cancers in Ilorin and Lagos metropolis.

Methods

Information on herbal practices, medicinal plants and personal information form herbal practitioners was collected using questionnaires and oral interviews. Plants cited were collected, identified and assigned voucher numbers. The names of plants were further authenticated using the plant list (www.theplantlist.org) and the world flora (www.worldfloraonline.org).

Results

A total of 41 plants were identified via oral interviews and questionnaires from 65 willing respondents within Ilorin and Lagos metropolis, respectively. Pistia stratiotes was the most frequently cited plant with a citation frequency of 57.1 % whereas, Mangifera indica Linn was the least cited plant with a citation frequency of 2.9 %. Plants parts including leaf, bark, root, and seed were prepared as concoctions, decoctions or powders and administered topically or orally to treat breast, prostate, cervical, skin and ovarian cancer.

Conclusion

The study revealed that medicinal plants previously shown to have cytotoxicity in vitro are currently used for cancers in traditional medicine practice. Twenty six medicinal plants are mentioned here for the first time as anticancer plants and could serve as sources for novel cytotoxic agents against cancers.

Keywords: Medicinal plants; Cancer; Traditional medicine; Nigeria; Cytotoxicity; Ethnopharmacology

Alexandre Specht, Silvana Vieira de Paula-Moraes, Daniel Ricardo Sosa-Gómez,

Host plants of Chrysodeixis includens (Walker) (Lepidoptera, Noctuidae, Plusiinae),

Revista Brasileira de Entomologia,

Volume 59, Issue 4,

2015,

Pages 343-345,

ISSN 0085-5626,

https://doi.org/10.1016/j.rbe.2015.09.002.

(https://www.sciencedirect.com/science/article/pii/S0085562615001089)

Abstract: This work has the objective to catalogue the information of Chrysodeixis includens (Walker, [1858]) (Lepidoptera: Noctuidae: Plusiinae) host plants. The list of plants comprehends new reports of host plants in Brazil and information from literature review around the world. It is listed 174 plants which are from 39 botanic families. The higher number of host plants of C. includens are in Asteraceae (29), Solanaceae (21), Fabaceae (18) and Lamiaceae (12).

Keywords: Caterpillar; Inventory; Polyphagy; Soybean looper

Selvaraj Selin-Rani, Sengottayan Senthil-Nathan, Annamalai Thanigaivel, Prabhakaran Vasantha-Srinivasan, Edward-Sam Edwin, Athirstam Ponsankar, Jalasteen Lija-Escaline, Kandaswamy Kalaivani, Ahmed Abdel-Megeed, Wayne B. Hunter, Rocco T. Alessandro,

Toxicity and physiological effect of quercetin on generalist herbivore, Spodoptera litura Fab. and a non-target earthworm Eisenia fetida Savigny,

Chemosphere,

Volume 165,

2016,

Pages 257-267,

ISSN 0045-6535,

https://doi.org/10.1016/j.chemosphere.2016.08.136.

(https://www.sciencedirect.com/science/article/pii/S0045653516311717)

Abstract: A novel flavonoid, quercetin, was isolated from the medicinal plant Euphorbia hirta L. through chromatography techniques including: TLC, Column chromatography, NMR and then screened for toxicity to larvae of Spodoptera litura Fab. Bioassays were used to analyze pupal weight, survival rate, fecundity, egg hatchability, population growth index, Nutritional index and histopathology of treated larvae at a range of E. hirta extract concentrations. Results of toxicity assays demonstrated that, 6 ppm of quercetin caused 94.6% mortality of second, 91.8% of third, 88% of fourth, and 85.2% of fifth instars respectively. The lethal concentrations (LC50 and LC90) was calculated as 10.88 and 69.91 ppm for fourth instar larvae. The changes in consumption ratio and approximate digestibility produced a reduction in growth rates. Histopathology examinations revealed that the cell organelles were severely infected. Analyses of earthworm toxicity effects resulted in significantly lower rates compared to synthetic insecticides (chloropyrifos and cypermethrin). These results suggests that the botanical compound (quercetin), could have a part as a new biorational product which provides an ecofriendly alternative. Validation of the potential of quercetin, still needs to be demonstrated under field conditions, where formulation will be important in maintaining the activity.

Keywords: Asthma-weed; Biorational insecticide; Secondary metabolites; Cut worm; Nutritional indices; Mortality; Earthworm toxicity; Histology

Mahmoud Nasrollahzadeh, Monireh Atarod, Mohaddeseh Sajjadi, S. Mohammad Sajadi, Zahra Issaabadi,

Chapter 6 - Plant-Mediated Green Synthesis of Nanostructures: Mechanisms, Characterization, and Applications,

Editor(s): Mahmoud Nasrollahzadeh, S. Mohammad Sajadi, Mohaddeseh Sajjadi, Zahra Issaabadi, Monireh Atarod,

Interface Science and Technology,

Elsevier,

Volume 28,

2019,

Pages 199-322,

ISSN 1573-4285,

ISBN 9780128135860,

https://doi.org/10.1016/B978-0-12-813586-0.00006-7.

(https://www.sciencedirect.com/science/article/pii/B9780128135860000067)

Abstract: In recent years, the development of efficient green chemistry methods, used for the synthesis of metal nanoparticles, has received exponentially increasing interest because biomaterial-based routes eliminate the need to use harsh or toxic chemicals. This chapter presents the advances made in green nanostructures and the biological mechanisms of nanoparticle biosynthesis, as well as providing characterization of nanoparticles. Many interesting biological tools, such as bacteria, yeasts, fungi, and plants, have been developed for the synthesis of greener nanoparticles. Among these reported organisms, plants owing to their diversity and sustainability, seem to be the best candidates for use as economic and valuable alternatives for the large-scale biosynthesis of nanoparticles. The preparation of nanoparticles via these approaches provides higher reduction rates of metal precursors and nanoparticles with better-defined sizes, morphologies, and stabilities. Herein, we consider and discuss some recent advances in terms of types of green nanostructures, plant sources used for the synthesis of nanostructures, phytochemicals involved in the green synthesis of nanostructures, green synthesis of nanoparticles, the stability of green synthesized nanoparticles, mechanisms proposed for the synthesis of nanostructures, and finally the characterization of green nanostructures.

Keywords: Green nanostructures; Biological mechanisms; Plant sources; Characterization

Shakeel Ijaz, Naveed Akhtar, Muhammad Shoaib Khan, Abdul Hameed, Muhammad Irfan, Muhammad Adeel Arshad, Sajid Ali, Muhammad Asrar,

Plant derived anticancer agents: A green approach towards skin cancers,

Biomedicine & Pharmacotherapy,

Volume 103,

2018,

Pages 1643-1651,

ISSN 0753-3322,

https://doi.org/10.1016/j.biopha.2018.04.113.

(https://www.sciencedirect.com/science/article/pii/S0753332218318560)

Abstract: Plants have been used as medicinal agents since the origin of mankind. High cost and severe side effects associated with conventional chemotherapy has limited their general acceptability and fuel up the search for alternate options. The alternative treatment options like phytochemicals have come up with ease of availability and cost effectiveness. Owing to their general acceptance, safety, low side effects and multistep targeting in signal transduction pathways, plant derived phyto-constituents have promising anti-carcinogenic potential for skin related cancers. This leads to the surge in research of new phytochemicals for the prevention and cure of a variety of skin cancers which are major cause of morbidity and mortality in present world. Although very limited clinical data involving humans is available in literature to demonstrate favorable eﬀ ;ects of phyto-constituents on various types of skin carcinomas yet the topical treatment with these plant derived anticancer phytochemicals is very promising. There are various mechanisms and pathways responsible for antitumor activity of plant derived medicinal compounds such as loss of mitochondrial membrane potential, release of cytochrome-c, Down regulation of Anti-apoptotic proteins and Up regulation of pro-apoptotic proteins, Activation of Caspase, Fas, FADD, p53 and c-Jun signaling pathway, Inhibition of Akt signaling pathway, phosphorylation of ERK, P13K, Raf, survivin gene, STAT 3 and NF-kB. In-vitro testing of skin cancer cell lines models offers the opportunity for identifying mechanisms of action of compounds from plant origin against variety of skin related cancers. This review thus aims at providing an overview of plant derived anti-cancer compounds which have been reported to show promising anti-carcinogenic effects against various skin cancer cell lines and on animal models. Phytochemicals that are discussed in this review include steroids, coumarines, trepenes, essential oils, alkaloids, esters, ethers, resins, phenols and flavonoids. This review also provides information about marketed formulations developed so far from plant derived compounds for skin cancer prevention and treatment.

Keywords: Skin cancer; Phytochemicals; Phyto-constituents; Plant derived; Anti-carcinogenic

Appendix D - Chinese Herb Cross Reference Table,

Editor(s): Susan G. Wynn, Steve Marsden,

Manual of Natural Veterinary Medicine,

Mosby,

2003,

Pages 656-667,

ISBN 9780323013543,

https://doi.org/10.1016/B978-0-323-01354-3.50028-0.

(https://www.sciencedirect.com/science/article/pii/B9780323013543500280)

Maja Dal Cero, Reinhard Saller, Caroline S. Weckerle,

The use of the local flora in Switzerland: A comparison of past and recent medicinal plant knowledge,

Journal of Ethnopharmacology,

Volume 151, Issue 1,

2014,

Pages 253-264,

ISSN 0378-8741,

https://doi.org/10.1016/j.jep.2013.10.035.

(https://www.sciencedirect.com/science/article/pii/S0378874113007423)

Abstract: Ethnopharmacological relevance

This analysis of documented medicinal plants of the Swiss Flora over the last two millennia provides a rich source of knowledge on earlier uses of plants and use patterns of the local flora. We ask which local plant species were used during different time periods of the last 2000 years and how the numbers of species and the use intensity of specific plant families, growth forms and habitats changed over time.

Materials and methods

Totally 25 herbals from the antiquity, monastic medicine, Renaissance, early modern era and the contemporary time as well as five recent ethnobotanical studies were considered. Use patterns were analysed with the Bayesian approach.

Results

A total of 768 species, i.e. 32% of the vascular plants of the Swiss Flora have been documented as medicinal plants. Numbers increase until the monastic period (366 spp.) and the Renaissance (476) and remain relatively stable since then (modern and contemporary era: 477). But, 465 formerly documented species do not occur in the ethnobotanical studies and thus seem not to be used any more. Overall, 104 species are documented through all time periods. Archeophytes, trees and forest plants are generally overrepresented in herbals from all time periods while plants from above the timberline are generally underrepresented. Most widely used are the Lamiaceae and Apiaceae.

Conclusion

A constant body of medicinal plant knowledge in Switzerland exists since ancient time. This knowledge was always influenced by knowledge from neighboring countries and no “typical Swiss specialties” seem to exist. Medicinal plants are not randomly chosen from the available flora. Certain species are deliberately introduced others are neglected. This process, which is still ongoing, can be traced back with the help of herbals to the antiquity.

Keywords: Ethnobotany; Herbals; Medical history; Medicinal plants; Swiss flora

Pavithra Chinnasamy, Rajendran Arumugam, Sarvalingam Ariyan,

In silico validation of the indigenous knowledge of the herbal medicines among tribal communities in Sathyamangalam wildlife sanctuary, India,

Journal of Traditional and Complementary Medicine,

Volume 9, Issue 2,

2019,

Pages 143-155,

ISSN 2225-4110,

https://doi.org/10.1016/j.jtcme.2018.01.008.

(https://www.sciencedirect.com/science/article/pii/S2225411018300087)

Abstract: The ethno-botanical documentation among ethnic people in Sathyamangalam wildlife sanctuary, Tamil Nadu, India has been investigated for the first time. A total of 61 medicinal plants having new combination uses were reported with adjuvant in the treatment of dermatological, Genitourinary and gastrointestinal ailments. This study could help in the recovery and conservation of traditional medicine system among educated generation. The present study was aimed to: (1) documentation of the traditional knowledge (2) quantitative analysis using Use value (UV), Informant consensus factor (ICF), Index of agreement on remedies (IAR), Relative frequency citation (RFC) and Cultural Importance index (CII) (3) validation of ethno-botanical data using in silico biological activity and toxicity prediction studies. Semi-structured direct interviews were conducted to acquire information from the study area tribes. Total of 89 tribes including both gender among various communities were interviewed and their ethno-botanical knowledge was documented. The data were assessed using ethno-botanical indices methods to estimate the consistency of usage herbal knowledge in various ailments. A total of 61 species were recorded for treatment of categorized ailments. The collected medicinal information from ethnic groups shows remarkable new usage of medicinal plants to particular ailments. Our comparative in silico studies also supported the traditional medicine results with correspondence to their bioactive. Traditional knowledge of ethnic people also linked to their culture and history. This study also infers the usage of traditional plant based medicine. Further research related to the bioactivities of reported plants should be encouraged to explore the importance in pharmaceutical industry.

Keywords: Ailments; Documentation; In silico; Quantitative indices; Traditional knowledge

Ali Parsaeimehr, Elmira Sargsyan, Amir Reza Jassbi,

Chapter 12 - Perspectives and Key Factors Affecting the Use of Herbal Extracts against Multidrug-Resistant Gram-Negative Bacteria,

Editor(s): Mahendra Kumar Rai, Kateryna Volodymyrivna Kon,

Fighting Multidrug Resistance with Herbal Extracts, Essential Oils and Their Components,

Academic Press,

2013,

Pages 181-190,

ISBN 9780123985392,

https://doi.org/10.1016/B978-0-12-398539-2.00012-4.

(https://www.sciencedirect.com/science/article/pii/B9780123985392000124)

Abstract: In today’s world, multidrug resistance appears to be one of the major health problems; however, screening, discovering and presenting novel effectual phytochemicals from medicinal plants have provided a promising outlook. Phytomedicines have been used for treating diseases and infections since time immemorial, but identifying new phytochemicals and effectual treatment strategies are essential in the fight against multidrug-resistant bacteria. Currently, isolated compounds such as α-mangostin, carnosic acid, epigallocatechin gallate, linalool, myricetin, novoimanin, α-terpineol, and totarol, have exhibited synergistic activity with antibiotics against resistant bacteria. This chapter presents information on multidrug-resistant Gram-negative bacteria and the use of herbal extracts against them.

Keywords: multidrug resistance; herbal extracts; Gram-negative bacteria; secondary metabolites

Jeroni Galmés, Sebastià Capó-Bauçà, Ülo Niinemets, Concepción Iñiguez,

Potential improvement of photosynthetic CO2 assimilation in crops by exploiting the natural variation in the temperature response of Rubisco catalytic traits,

Current Opinion in Plant Biology,

Volume 49,

2019,

Pages 60-67,

ISSN 1369-5266,

https://doi.org/10.1016/j.pbi.2019.05.002.

(https://www.sciencedirect.com/science/article/pii/S1369526619300019)

Abstract: The enhancement of the photosynthetic capacity of crops by the expression of more efficient Rubisco versions has been a main target in the field of plant photosynthesis improvement. However, such an increase in the photosynthetic efficiency will depend on the environmental conditions and on the responsiveness of Rubisco to temperature and CO2 availability. After an exhaustive compilation and standardization of the data published so far, a large natural variability in the thermal responses of Rubisco kinetic parameters in higher plant species was revealed. The variability observed was related to the photosynthetic type but a limited adaptation to the species thermal environment was found. We provide theoretical evidence that the existence of distinctive Rubisco responses to varying temperature and CO2 concentration constitutes a promising avenue for increasing the photosynthetic capacity of important crops under future climatic conditions.

Antu Das, Junaid Jibran Jawed, Manash C. Das, Padmani Sandhu, Utpal C. De, Biswanath Dinda, Yusuf Akhter, Surajit Bhattacharjee,

Antileishmanial and immunomodulatory activities of lupeol, a triterpene compound isolated from Sterculia villosa,

International Journal of Antimicrobial Agents,

Volume 50, Issue 4,

2017,

Pages 512-522,

ISSN 0924-8579,

https://doi.org/10.1016/j.ijantimicag.2017.04.022.

(https://www.sciencedirect.com/science/article/pii/S0924857917302339)

Abstract: Visceral leishmaniasis (VL) is one of the most severe forms of leishmaniasis, caused by the protozoan parasite Leishmania donovani. Nowadays there is a growing interest in the therapeutic use of natural products to treat parasitic diseases. Sterculia villosa is an ethnomedicinally important plant. A triterpenoid was isolated from this plant and was screened for its antileishmanial and immunomodulatory activities in vitro and in vivo. Biochemical colour test and spectroscopic data confirmed that the isolated pure compound was lupeol. Lupeol exhibited significant antileishmanial activity, with IC50 values of 65 ± 0.41 µg/mL and 15 ± 0.45 µg/mL against promastigote and amastigote forms, respectively. Lupeol caused maximum cytoplasmic membrane damage of L. donovani promastigote at its IC50 dose. It is well known that during infection the Leishmania parasite exerts its pathogenicity in the host by suppressing nitric oxide (NO) production and inhibiting pro-inflammatory responses. It was observed that lupeol induces NO generation in L. donovani-infected macrophages, followed by upregulation of pro-inflammatory cytokines and downregulation of anti-inflammatory cytokines. Lupeol was also found to reduce the hepatic and splenic parasite burden through upregulation of the pro-inflammatory response in L. donovani-infected BALB/c mice. Strong binding affinity of lupeol was observed for four major potential drug targets, namely pteridine reductase 1, adenine phosphoribosyltransferase, lipophosphoglycan biosynthetic protein and glycoprotein 63 of L. donovani, which also supported its antileishmanial and immunomodulatory activities. Therefore, the present study highlights the antileishmanial and immunomodulatory activities of lupeol in an in vitro and in vivo model of VL.

Keywords: Leishmania donovani; IC50; Membrane potential; Nitric oxide; Cytokines; Molecular docking

E. Valiakos, M. Marselos, N. Sakellaridis, Th. Constantinidis, H. Skaltsa,

Ethnopharmacological approach to the herbal medicines of the “Antidotes” in Nikolaos Myrepsos׳ Dynameron,

Journal of Ethnopharmacology,

Volume 163,

2015,

Pages 68-82,

ISSN 0378-8741,

https://doi.org/10.1016/j.jep.2015.01.005.

(https://www.sciencedirect.com/science/article/pii/S0378874115000136)

Abstract: Ethnopharmacological relevance:This paper focuses on the plants quoted in the recipes of the first chapter entitled “About the Antidotes” belonging to the first and largest section “Element Alpha” of Nikolaos Myrepsos׳ Dynameron, a medieval medical manuscript. Nikolaos Myrepsos was a Byzantine physician at the court of John III Doukas Vatatzes at Nicaea (13th century). He wrote in Greek a rich collection of 2667 recipes, the richest number known in late Byzantine era, conventionally known as Dynameron and divided into 24 sections, the “Elements”. The only existing translation of this work is in Latin, released in 1549 in Basel by Leonhart Fuchs. Since no other translation has ever been made in any language, this work still remains poorly known. Materials and Methods:Our primary source material was the codex written in 1339 and kept in the National Library of France (in Paris) under the number grec. 2243. For comparison, all the other codices, which contain the entire manuscript, have also been studied, namely the codices EBE 1478 (National Library of Greece, Athens), grec. 2237 and grec. 2238 (both in Paris), Lavra Ε 192 (Mont Athos, Monastery of Megisti Lavra), Barocci 171 (Oxford) and Revilla 83 (Escorial). Results: The exhaustive study of the “About the Antidotes” led us to the interpretation of 293 plant names among which we recognized 39 medicinal plants listed by the European Medicines Agency, (Herbal Medicines, www.ema.eu); the therapeutic indications of some of them provided by Myrepsos were similar or related to their current ones, as given in their monographs. The plants belong to various families of which the most frequent are: Apiaceae 10.6%; Lamiaceae 9.2%; Asteraceae 8.9%; Fabaceae 6.8% and Rosaceae 5.1%. The most frequently mentioned plants even under several different names are the following: Apium graveolens L., Crocus sativus L., Nardostachys jatamansi (D. Don) DC., Zingiber officinale Roscoe, Rosa centifolia L., Syzygium aromaticum (L.) Merr. & L.M. Perry, Papaver somniferum L., Costus sp., Petroselinum crispum (Mill.) Fuss, Anethum graveolens L., Foeniculum vulgare Mill., Daucus carota L. Conclusions: This research led us to the conclusion that the content of “About the Antidotes” is a valuable source for the study of recipes based mainly on medicinal plants, most of them inherited from classic ancient Greek and Hellenistic periods.

Keywords: Historical texts; Dynameron; About the Antidotes; Nikolaos Myrepsos; Byzantine medicine; Herbal medicines

Zohreh Abolhasanzadeh, Hajar Ashrafi, Parmis Badr, Amir Azadi,

Traditional neurotherapeutics approach intended for direct nose to brain delivery,

Journal of Ethnopharmacology,

Volume 209,

2017,

Pages 116-123,

ISSN 0378-8741,

https://doi.org/10.1016/j.jep.2017.07.026.

(https://www.sciencedirect.com/science/article/pii/S0378874117303215)

Abstract: Ethnopharmacological relevance

Nasal delivery systems have a significant role in Persian traditional medicine. Most of them were utilized for central nervous system (CNS)-related disorders. In modern medicine, nasal drug delivery systems for brain delivery are highly regarded.

Aim of the study

Despite recent advances in drug delivery to the (CNS), delivery of therapeutics to the brain remains a major challenge because of the blood brain barrier (BBB). There are several mechanisms which regulate the drug transfer across the BBB. Local administration methods of therapeutic agents are often associated with adverse events, while the intranasal pathway has been suggested as a non-invasive alternative route to deliver drugs to the brain. This route can bypass the BBB and deliver drug molecules directly to the CNS. There are different nasal formulations have been addressed in Persian traditional pharmacopeias. The present review attempt to explore the famous and practical Qarabadin to find ancient nasal dosage forms.

Materials and methods

With an explore on traditional herbs in google scholar, scopus and science direct, we have found some original and review articles which have demonstrated our findings on the use of traditional herbs for CNS disorders. Four encyclopedia of multi-component formulations, including Qarabadin Salehi (1766), Qarabadin kabir (1781),Qarabadin Ghaderi (18th century), and Qarabadin Azam (1853), were searched for nasal formulations having CNS-related indications. Formulations were categorized based on dosage forms, and also, diseases which they were suggested for. While the names of illnesses were in ancient terminology of Traditional Medicine, they were translated to modern medical terminology by comparing their definitions, signs, and symptoms from two medical systems. Typical samples of each dosage form have been mentioned with details like amount of ingredients, scientific names of plants, and considerations pertaining to preparation or usage.

Results

Among all traditional nasal formulations, seven types were found that is used for sicknesses relating to CNS including Saoot, Nafookh, Atoos, Nashoogh, Shamoom, Lakhlakheh, and Bakhoor.

Conclusions

The findings of this study reveal the physicochemical characteristics of each formulation, route of administration, and type of disease which they are suitable and also present some famous formulations.

Keywords: Traditional nasal formulation; Saoot; Nafookh; Atoos; Nashoogh; Shamoom; Lakhlakheh; Bakhoor

Gregg P. Robideau, Nicolas Rodrigue, C. André Lévesque,

Codon-based phylogenetics introduces novel flagellar gene markers to oomycete systematics,

Molecular Phylogenetics and Evolution,

Volume 79,

2014,

Pages 279-291,

ISSN 1055-7903,

https://doi.org/10.1016/j.ympev.2014.04.009.

(https://www.sciencedirect.com/science/article/pii/S1055790314001377)

Abstract: Oomycete systematics has traditionally been reliant on ribosomal RNA and mitochondrial cytochrome oxidase sequences. Here we report the use of two single-copy protein-coding flagellar genes, PF16 and OCM1, in oomycete systematics, showing their utility in phylogenetic reconstruction and species identification. Applying a recently proposed mutation–selection model of codon substitution, the phylogenetic relationships inferred by flagellar genes are largely in agreement with the current views of oomycete evolution, whereas nucleotide- and amino acid-level models produce biologically implausible reconstructions. Interesting parallels exist between the phylogeny inferred from the flagellar genes and zoospore ontology, providing external support for the tree obtained using the codon model. The resolution achieved for species identification is ample using PF16, and quite robust using OCM1, and the described PCR primers are able to amplify both genes for a range of oomycete genera. Altogether, when analyzed with a rich codon substitution model, these flagellar genes provide useful markers for the oomycete molecular toolbox.

Keywords: Oomycete; Flagella; Taxonomic marker; Codon substitution model; Phylogenetics

Ping Kong, Patricia A. Richardson, Gary W. Moorman, Chuanxue Hong,

Single-strand conformational polymorphism analysis of the ribosomal internal transcribed spacer 1 for rapid species identification within the genus Pythium,

FEMS Microbiology Letters,

Volume 240, Issue 2,

2004,

Pages 229-236,

ISSN 0378-1097,

https://doi.org/10.1016/j.femsle.2004.09.034.

(https://www.sciencedirect.com/science/article/pii/S0378109704006962)

Abstract: Single-strand conformational polymorphism (SSCP) of the ribosomal internal transcribed spacer 1 (ITS-1) was characterized for 58 isolates of Pythium, representing 41 species from the five groups of Plaats-Niterink. Thirty-one species each produced a distinct SSCP pattern. Three species produced more than one unique pattern, corresponding to morphological subgrouping. The remaining seven species produced three distinct patterns with two or three morphologically similar species sharing a pattern. A successful blind test with four samples and the identification of eight previously unknown isolates from irrigation water demonstrated the reliability of this technique for species identification. Each SSCP pattern was defined and described by the positions of the top and bottom bands and the number of bands in between, which allows laboratories to use this technique without need to access the type isolates of Pythium species.

Keywords: Pythium; Species differentiation; ITS-1; SSCP

Alex Asase, Daniel T. Yohonu,

Ethnobotanical study of herbal medicines for management of diabetes mellitus in Dangme West District of southern Ghana,

Journal of Herbal Medicine,

Volume 6, Issue 4,

2016,

Pages 204-209,

ISSN 2210-8033,

https://doi.org/10.1016/j.hermed.2016.07.002.

(https://www.sciencedirect.com/science/article/pii/S2210803316300574)

Abstract: Background

Diabetes mellitus is a major threat to human health in the 21st century. Despite existing conventional drugs, it is still difficult to attain optimum glycaemic control among many diabetic patients. The aim of this study was to investigate traditional knowledge and medicinal plants used for management of diabetes mellitus in Dangme West District of southern Ghana.

Methods

Data was collected from 30 traditional healers by face –to –face questionnaire interviews. Plant samples were collected and processed as voucher specimens following standard ethnobotanical practice.

Results

About 75.8% of healers mentioned that excessive intake of sugar was the principal cause of diabetes mellitus, 12% mentioned witchcraft while 6% attributed cause of the disease to family history. Twenty species of plants were identified as being used for the management of diabetes mellitus in the study area and the most commonly cited plants were Vernonia amygdalina, Aloe vera and Launaea taraxacifolia. Leaves were the part of the plants commonly used for the preparation of mostly decoctions (75%) and infusions (10%). About 15% of the plant materials were added to food and directly eaten. The main route of administration of herbal preparations was orally by drinking the decoctions.

Conclusion

In this study, eight of the plants reported as being used for the management of diabetes mellitus had been previously documented as being used for this condition and 40% of the plants had been confirmed as possessing hypoglycemic effects. Nevertheless, scientific investigations in regard to the anti-diabetic effects and toxicity of the herbal preparations traditionally used are needed.

Keywords: Medicinal plants; Diabetes mellitus; Dangme West District

Appendix 3 - Glossary of Āyurvedic Herbs, Minerals and Animal Products,

Editor(s): Todd Caldecott,

Ayurveda,

Mosby,

2006,

Pages 318-323,

ISBN 9780723434108,

https://doi.org/10.1016/B978-0-7234-3410-8.50072-6.

(https://www.sciencedirect.com/science/article/pii/B9780723434108500726)

I.T. Oyeyemi, K.M. Akinseye, S.S. Adebayo, M.T. Oyetunji, O.T. Oyeyemi,

Ethnobotanical survey of the plants used for the management of malaria in Ondo State, Nigeria,

South African Journal of Botany,

Volume 124,

2019,

Pages 391-401,

ISSN 0254-6299,

https://doi.org/10.1016/j.sajb.2019.06.003.

(https://www.sciencedirect.com/science/article/pii/S0254629919306520)

Abstract: An ethnobotanical survey of the plants used for the traditional management of malaria by the residents of Ondo State, Nigeria was carried out. Data was collected by interviews using a semi-structured questionnaire. A total of 162 indigenous people were interviewed [male, 110 (68%); female, 52(32%)]. A total of 97 plant species belonging to 52 different families were identified. Detailed information about their vernacular names, modes of preparation and previous investigations of their antiplasmodial/antimalarial effects was also compiled. Of the plants identified, the family Euphorbiaceae is predominant while Mangifera indica, Anacardium occidentale and Cymbopogon citratus are the most cited species. Only a few (37%) of the plants identified have been subjected to scientific investigations, most of which are only preliminary. Of the tested plants, some have shown in vivo activities comparable with that of orthodox antimalarial drugs. There is, therefore, a need to scientifically investigate the antimalarial potential of these plants as they serve as potential source of lead compounds for malaria therapy.

Keywords: Ethnobotanical survey; Malaria; Medicinal plants

Tiara da Silva Coelho Bortolo, Rogério Marchiosi, Joselaine Viganó, Rita de Cássia Siqueira-Soares, Ana Paula Ferro, Gabriela Elen Barreto, Graciene de Souza Bido, Josielle Abrahão, Wanderley Dantas dos Santos, Osvaldo Ferrarese-Filho,

Trans-aconitic acid inhibits the growth and photosynthesis of Glycine max,

Plant Physiology and Biochemistry,

Volume 132,

2018,

Pages 490-496,

ISSN 0981-9428,

https://doi.org/10.1016/j.plaphy.2018.09.036.

(https://www.sciencedirect.com/science/article/pii/S0981942818304352)

Abstract: Grasses producing trans-aconitic acid, a geometric isomer of cis-aconitic acid, are often used in Glycine max rotation systems. However, the effects of trans-aconitic acid on Glycine max are unknown. We conducted a hydroponic experiment to evaluate the effects of 2.5–10 mM trans-aconitic acid on Glycine max growth and photosynthesis. The results revealed that the enhanced H2O2 production in the roots increased the membrane permeability and reduced the water uptake. These effects culminated with a reduced stomatal conductance (gs), which seems to be the main cause for a decreased photosynthetic rate (A). Due to low gs, the limited CO2 assimilation may have overexcited the photosystems, as indicated by the high production of H2O2 in leaves. After 96 h of incubation, and due to H2O2-induced damage to photosystems, a probable non-stomatal limitation for photosynthesis contributed to reducing A. This is corroborated by the significant decrease in the quantum yield of electron flow through photosystem II in vivo (ΦPSII) and the chlorophyll content. Taken together, the damage to the root system and photosynthetic apparatus caused by trans-aconitic acid significantly reduced the Glycine max plant growth.

Keywords: Photosynthetic rate; Chlorophyll a fluorescence; Gas exchange; Reactive oxygen species

Sami Asir Al-Robai, Haidar Abdalgadir Mohamed, Abdelazim Ali Ahmed, Abdul Wali Ahmed Al-Khulaidi,

Effects of elevation gradients and soil components on the vegetation density and species diversity of Alabna escarpment, southwestern Saudi Arabia,

Acta Ecologica Sinica,

Volume 39, Issue 3,

2019,

Pages 202-211,

ISSN 1872-2032,

https://doi.org/10.1016/j.chnaes.2018.09.008.

(https://www.sciencedirect.com/science/article/pii/S1872203218300544)

Abstract: The study was conducted on Alabna escarpment, southwestern Saudi Arabia, for evaluating the effects of elevation and soil components on the species diversity and plant community. Among 241 species (167 genera and 53 families) recorded in total, therophytes and chamaephytes prevailed in the area while bryophytes were rare. Clustering analysis revealed the presence of four community types which had a remarkable overlapping in species composition. Group 2 was the most diverse and represented by one stand with 58 species in which 19 plant species were only recorded in this group. Group 1 comprised two stands and the other two groups had more than two stands. Diversity and distribution of species were affected by elevation and element contents in the soil. The soil was slightly alkaline, not saline, contained a reasonable amount of elements and very poor in P content. Heavy metals were found in neglected quantities indicating that the area was unpolluted.

Keywords: Alabna escarpment; Elevation gradient; Plant community; Soil components

Bianca Tribess, Gabrielli Melatto Pintarelli, Larissa Alida Bini, Anderson Camargo, Luís Adriano Funez, André Luís de Gasper, Ana Lúcia Bertarello Zeni,

Ethnobotanical study of plants used for therapeutic purposes in the Atlantic Forest region, Southern Brazil,

Journal of Ethnopharmacology,

Volume 164,

2015,

Pages 136-146,

ISSN 0378-8741,

https://doi.org/10.1016/j.jep.2015.02.005.

(https://www.sciencedirect.com/science/article/pii/S0378874115000768)

Abstract: Ethnopharmacological relevance

Atlantic Forest is a biome in dangerous situation and it lacks wider information on species with medicinal purposes used by people in this area. In this study an ethnobotanical survey was conducted in Apiúna district, Brazil with the goal of assessing traditional knowledge of medicinal plants used by rural communities in a region covered by Atlantic Forest.

Materials and methods

The ethnobotanical data were collected through semi-structured interviews and a free list of plants used for medicinal purposes. The respondents were selected by snow ball method. Therefore, the therapeutic use of plants was investigated and the species cited was collected and identified. Local plant uses were evaluated using ethnobotanical indices of diversity and equitability, and then compared with those obtained in other regions of Atlantic Forest in Brazil. Besides, the informant consensus factor (ICF) was calculated.

Results

A total of 162 species belonging to 61 families were recorded, mainly Asteraceae and Lamiaceae. Furthermore, the species cited, 45.06% were native and 54.94% were considered exotic. The most frequently reported medicinal uses were the symptoms and signs (17.42%), digestive system (15.33%) and, infectious and parasitic diseases (12.73%). Although, the ICF calculation showed that mental and behavioral (0.85), respiratory system (0.79) and, digestive and genitourinary system diseases (0.78 for both) were the categories with higher values reached. Usually, the administration is oral from leaves preparations.

Conclusions

Folk medicine in rural communities in this region of Atlantic Forest is an important source of primary health care. The results indicate an available knowledge of medicinal plants uses in this area, when compared to other regions previously studied. The fact that this research was conducted next to a conservation area makes it possible to dispose the knowledge organized here into a tool for environmental education as well as preservation. Moreover, the pharmacological information will further contribute for the validation and the use of these species in Brazilian health programs benefiting the population.

Keywords: Ethnobotany; Therapeutic species; Atlantic Forest; Serra do Itajaí National Park

Mohamed Soliman Elshikh, Soo-Yong Kim, Mohammad Ajmal Ali, Fahad Al-Hemaid, Shen-Ming Chen, Sangho Choi, Mohammad Oliur Rahman, Meena Elangbam, Joongku Lee,

Comparative analysis of cp genome of Fagonia indica growing in desert and its implications in pattern of similarity and variations,

Saudi Journal of Biological Sciences,

Volume 27, Issue 1,

2020,

Pages 229-232,

ISSN 1319-562X,

https://doi.org/10.1016/j.sjbs.2019.08.016.

(https://www.sciencedirect.com/science/article/pii/S1319562X19301573)

Abstract: The chloroplasts genome encodes several key proteins that involves in the process of the photosynthesis and also in other metabolic processes important for growth and development, yield, biomass, and plant interactions with their environment. The present study aimed to sequencing of cp genome of Fagonia indica Burm.f (Zygophyllaceae), -a plant that occurs even in the hot desert condition of the inner zone of Rub′ al-Khali (the Empty Quarter) of south-central Arabia, and its comparative analyses with the representative of the sequence of the different categories [viz. (a) with the other member of the family Zygophyllaceae, and with the representatives from: (b) different clade of the angiosperms, (c) flowering plants occurs in different major habitats, (d) different groups of plants, (e) different group of plants having range of biomass, (f) C3 and C4 plants, and (g) the representative from very common, rare and major high yielding crop of the world] to unravel the genetic pattern of similarity and variations. The comparison of F. indica genome in different categories showed strong evidence and further support for the conservative pattern of chloroplast genome, the coding and non-coding region remains conserved even in phylogenetically distant eukaryotic clades, and might not have the sole roles in organism′s yield, rarity or abundance and biomass, and in encountering the stress. Nevertheless, the result could be useful for molecular phylogenetic and molecular ecological and molecular mechanism of photosynthesis.

Keywords: cp DNA; Fagonia indica; Zygophyllaceae; Photosynthesis; Habitats; Biomass; Crop

Oulaid Touloun, Moulay Abdelmonaim El Hidan, Ali Boumezzough,

Species composition and geographical distribution of Saharan scorpion fauna, Morocco,

Asian Pacific Journal of Tropical Disease,

Volume 6, Issue 11,

2016,

Pages 878-881,

ISSN 2222-1808,

https://doi.org/10.1016/S2222-1808(16)61150-7.

(https://www.sciencedirect.com/science/article/pii/S2222180816611507)

Abstract: Objective

To describe the species composition of scorpions and to study its geographical distribution in Laayoune-Sakia El Hamra and Dakhla-Oued Ed Dahab regions in July 2014.

Methods

To locate scorpions, the ground was examined through searching the places under the stones, rocks and in burrows. The nocturnal missions were also conducted using portable ultraviolet lamps. The scorpions were subsequently identified in the laboratory.

Results

The results of the investigations in these regions showed the presence of five scorpion species, two of which Androctonus gonneti and Buthus bonito were endemic in Morocco.

Conclusions

This work is allowed to complete the inventory of the studied scorpion fauna and provides some considerations on the distribution patterns in the study area.

Keywords: Scorpions; Inventory; Distribution; Saharan regions; Morocco

Appendix 2 - Weeds Cited in Text11With a few exceptions, weeds are listed alphabetically the way the name is used in the original source. That is, field bindweed is listed with those plants beginning with “f” rather than as bindweed, “field.” 2The authority for the scientific name is given when it could be found after a reasonable search.Alphabetized by Common Name,

Editor(s): Robert L. Zimdahl,

Fundamentals of Weed Science (Fifth Edition),

Academic Press,

2019,

Pages 691-708,

ISBN 9780128111437,

https://doi.org/10.1016/B978-0-12-811143-7.15002-4.

(https://www.sciencedirect.com/science/article/pii/B9780128111437150024)

R.M. Dharmadasa, G.C. Akalanka, P.R.M. Muthukumarana, R.G.S. Wijesekara,

Ethnopharmacological survey on medicinal plants used in snakebite treatments in Western and Sabaragamuwa provinces in Sri Lanka,

Journal of Ethnopharmacology,

Volume 179,

2016,

Pages 110-127,

ISSN 0378-8741,

https://doi.org/10.1016/j.jep.2015.12.041.

(https://www.sciencedirect.com/science/article/pii/S0378874115302944)

Abstract: Ethnopharmacological relevance

Sri Lanka has a great diversity of snake species. In this relation, over 40,000 cases of snakebite accidents are reported annually from different agro-ecological regions of the country. Since more than 95% of victims rely on traditional treatments, there is an urgent necessity to improve the system. Traditional knowledge on snakebite treatments has been passed on from generation to generation within families. Unfortunately, there has been a limited update of information on pertinent issues related to this subject. In the present study we conducted a comprehensive survey on the types of medicinal plant materials, including the specific plant parts that are available for this purpose. In addition, various treatment types, frequency index, heavily used and rare materials, family wise distribution, challenges faced by traditional practitioners and future prospects were also explored.

Materials and methods

The present survey covered two provinces with a high population of traditional practitioners for snakebites treatment in Sri Lanka.Information was gathered from a total of seventy-four (74) traditional practitioners from the Sabaragamuwa and Western provinces. A questionnaire was prepared and pre-tested by 10–15 respondents prior to the survey. Actual data were gathered through face-to-face interviews. Collected data were tabulated and analyzed.

Results

A total of 341 different plant species belonging to 99 families were documented. The highest number of plants was reported from the family Fabaceae (32 species). This was followed by Malvaceae (16 species), Asteraceae (15 species), Rutaceae (13 species Apocyanaceae (14 species), Lamiaceae (11 species), Poaceae, Euphorbaceae and Phyllanthaceae (10 species per each) respectively. Different parts of the plant such as leaves (53.67%), barks (26.10%), entire plant (14.08%), roots (10.26%), bulbs (8.80%), seeds (7.62%), fruits (6.45%), buds (5.87%), flowers (3.23%) stems (2.93%) and latex (2.05%) were used for the preparation of nine different types of formulae. These formulae include oral administration (172 plant species), external bandaging (167 plant species), oiling for external application (34 plant species), steaming (33 plant species), creaming for wounds (6 plant species), nasal treatments (40 plant species), head treatments (23 plant species), treatment for eyes (4 plant species) and washing of wounds (9 plant species). Moreover, plants used for the different snake types, constraints faced by traditional practitioners, and their constructive suggestions were also discussed.

Conclusion

A pioneering attempt was made to exploit local knowledge on snakebite treatments for the conservation of valued medicinal plants and to promote primary health care needs in Sabaragamuwa and Western provinces in Sri Lanka. The documented plants together with the traditional knowledge could be effectively utilized for the isolation and characterization of antivenom for different snake species.

Keywords: Antivenom; Ethnomedicine; Medicinal plants; Snakebite; Sri Lanka Traditional knowledge

J.-P. Dumur,

Les pollinoses tropicales,

Revue Française d'Allergologie,

Volume 59, Issue 8,

2019,

Pages 610-616,

ISSN 1877-0320,

https://doi.org/10.1016/j.reval.2019.07.006.

(https://www.sciencedirect.com/science/article/pii/S1877032019303562)

Abstract: Résumé

La zone tropicale englobe géographiquement les territoires situés de chaque côté de l’équateur entre Tropique du Cancer au nord et Tropique du Capricorne au sud. Cet espace est caractérisé par un climat chaud rythmé par l’alternance d’une saison sèche et d’une saison des pluies. Ce climat spécifique influence notablement la pollinisation des plantes en termes de saisonnalité et de concentration et donc les caractéristiques cliniques des pathologies allergiques liées aux pollens. On constate d’ailleurs de très grandes différences avec les pollinoses des zones tempérées en termes d’épidémiologie et de clinique. En outre, les publications sur les pollinoses tropicales sont rares et peu documentées, rendant ce sujet peu connu des allergologues.

Geographically, the Tropics comprise the regions on either side of the equator between the Tropic of Cancer in the north and the Tropic of Capricorn in the south. This area is characterized by a warm climate punctuated by the alternation of a dry season and a rainy season. This specific climate significantly influences plant pollination in terms of seasonality and concentration, and thus affects the clinical characteristics of pollen-related allergies. There are also very marked epidemiological and clinical differences in relation to pollinoses in temperate zones. Further, publications on tropical pollinosis are scarce and poorly documented, resulting in limited knowledge of this subject among allergists.

Keywords: Pollens; Pollinoses tropicales; Allergie; Calendrier pollinique; Diagnostic; Pollens; Tropical pollinosis; Allergy; Pollen Counts; Diagnosis

Himanshu Patel, R.T. Vashi,

Chapter 3 - Feasibility of Naturally Prepared Adsorbent,

Editor(s): Himanshu Patel, R.T. Vashi,

Characterization and Treatment of Textile Wastewater,

Elsevier,

2015,

Pages 73-110,

ISBN 9780128023266,

https://doi.org/10.1016/B978-0-12-802326-6.00003-4.

(https://www.sciencedirect.com/science/article/pii/B9780128023266000034)

Abstract: This chapter starts with the basic introduction of adsorption, its types, and its mechanism. Investigated different sorbents for batch and column treatment are tabulated with respective references. Isotherms for batch (Freundlich and Langmuir) and columns (Thomas, Yoon-Nelson, bed depth service time, and Adams and Bohart) are discussed. Feasibility and comparison of naturally prepared adsorbents, that is, neem (Azadirachta indica) leaf powder, guava (Psidium guajava) leaf powder, and tamarind (Tamarindus indica) seed powder, and their activated forms using different acids are determined using various sophisticated analytic facilities like Fourier transform infrared, particle size distribution, scanning electron microcopy, and surface area, porosity, pore diameter, and pore volume analyses and also adsorptive batch treatment on dye solution. Treatment data using Freundlich and Langmuir isotherm are analyzed and compared. It is concluded that activated neem leaf powder, activated guava leaf powder using sulfuric acid, and normal tamarind seed powder are more suitable than their investigated analog adsorbents for the adsorption process to remove dyes and other contaminations.

Keywords: Adsorption; Naturally prepared adsorption; Analytic technique; Adsorption isotherm; Dye removal

Victor Kimpouni, Josérald Chaîph Mamboueni, Feldane Gladrich Mboussy Tsoungould, Elie Nsika Mikoko,

Ethnobotanical and phytotherapeutic study from Kouni community of the sub-prefecture of Kayes (Bouenza – Congo),

Heliyon,

Volume 5, Issue 8,

2019,

e02007,

ISSN 2405-8440,

https://doi.org/10.1016/j.heliyon.2019.e02007.

(https://www.sciencedirect.com/science/article/pii/S2405844019356622)

Abstract: The ethnobotanical and phytotherapeutic study conducted in Mvouandzi (4°10′00″ S, 13°25′00″ E), sub-prefecture of Kayes (Bouenza - Congo), is based on the floristic inventory, the personalized interviews and focus groups. The target population, aged between 15 to 70 years or more, is divided into 3 age groups, and consists of 46 informants (12 men and 34 women) who possess the plant secrets. The floristic inventory lists 60 useful species, corresponding to 53 genera and 35 families. The medicinal cohort is associated with 109 recipes and 57 diseases and symptoms. Classified as a sphere of diseases and symptoms, infectious and parasitic diseases predominate (27.11%) and retain 30.27% of recipes. All organs (vegetative and generative) intervene in the daily satisfaction of the needs of the populations. Ethno-sociological analysis reveals that the level of endogenous knowledge is proportional to the subjects' age and in this matter, women by virtue of their role as manager and guardian of morals, excel in the exploitation of empirical knowledge. Data on the value of ethnobotanical use, the informant consensus factor and the level of fidelity show that these plants are strongly involved in the primary care of this society. Notwithstanding the inseparable link between man and his environment, the value associated with this biodiversity, the socio-cultural foundation of the Kouni ethnic-linguistic community, is inevitably eroded. The reasons for this are the rural exodus, the main corollary of which is the ageing of the population, and the effects inherent in the construction of physical communication infrastructures.

Keywords: Public health; Ethnotherapy; Traditional pharmacopoeia; Phytodiversity; Socio-cultural value; Congo

E. Tuzlacı, M.K. Erol,

Turkish folk medicinal plants. Part II: Eğirdir (Isparta)1For Part I in this series, see Fitoterapia 1996;67:307.1,

Fitoterapia,

Volume 70, Issue 6,

1999,

Pages 593-610,

ISSN 0367-326X,

https://doi.org/10.1016/S0367-326X(99)00074-X.

(https://www.sciencedirect.com/science/article/pii/S0367326X9900074X)

Abstract: Sixty-six folk medicinal plants from E∂irdir (Turkey) are reported. Among them 56 species are wild and 10 species are cultivated plants. They are mostly used for kidney stones, ulcer, hemorrhoids, rheumatism, cold and as analgesic, diuretic and carminative.

Keywords: Folk medicine; Medicinal plants; Turkey

Rapanui glossary,

Editor(s): Valentí Rull,

Paleoecological Research on Easter Island,

Elsevier,

2020,

Pages ix-xi,

ISBN 9780128227275,

https://doi.org/10.1016/B978-0-12-822727-5.09986-4.

(https://www.sciencedirect.com/science/article/pii/B9780128227275099864)

O.S. Olorunnisola, A. Adetutu, A.J. Afolayan,

An inventory of plants commonly used in the treatment of some disease conditions in Ogbomoso, South West, Nigeria,

Journal of Ethnopharmacology,

Volume 161,

2015,

Pages 60-68,

ISSN 0378-8741,

https://doi.org/10.1016/j.jep.2014.10.001.

(https://www.sciencedirect.com/science/article/pii/S0378874114007041)

Abstract: Ethnopharmacological relevance

This study was designed to take an inventory of medicinal plants, recipes and methods commonly used traditionally to treat some cardiovascular and inflammatory diseases in five local government areas in Ogbomoso, Oyo State, Nigeria.

Material and methods

First-hand field survey through semi-structured questionnaire was employed in the 5 months study.

Results

A total of 101 plant species (medicinal plants (80.90%), spices (17.5%) and vegetables (1.53%)) belonging to 51 different families were mentioned for the treatment of various types of cardiovascular and inflammatory diseases. The survey revealed that 51.5% of the plants mentioned are used for the management of inflammatory diseases, 34.7% for the treatment of cardiovascular diseases and 11.9% of the plants are used for the treatment of both diseases. Euphorbiaceae (7.9%) are the most frequently used families of plants for the treatment of the various types of diseases mentioned, followed by Caesalpiaceae, (4.9%), Apocynoceae (4.9%) and Poaceae (4.9%). Fifty-nine recipes are usually prepared for the treatment of the six types of inflammatory diseases while twenty-three recipes are reportedly used for the treatment of the four types of cardiovascular diseases mentioned in this study. The recipes covered in the survey were mostly prepared from leaves (37.6%) and roots (23.8%) decoction or infusions. Medications are mostly administered orally with few numbers of the recipes showing side effect.

Conclusion

The study has documented indigenous plants in Ogbomoso as a potential source for the development of new drugs for the treatment of cardiovascular and inflammatory diseases.

Keywords: Cardiovascular diseases; Inflammatory diseases; Medicinal plants; Traditional medicine; Ogbomoso

Saleh Al-Qura'n,

Ethnobotanical survey of folk toxic plants in southern part of Jordan,

Toxicon,

Volume 46, Issue 2,

2005,

Pages 119-129,

ISSN 0041-0101,

https://doi.org/10.1016/j.toxicon.2005.04.010.

(https://www.sciencedirect.com/science/article/pii/S0041010105001388)

Abstract: Thirteen representative phytogeographical sites in the southern part of Jordan was surveyed to recognize the folk toxic plant wealth by calculating of FL, RPL, and ROP values. One hundred and twenty-five toxic species were recorded in the area of study belonging to 56 plant families that were mentioned by three informants or more, 53 toxic species (42.4%) were popular, since they were cited by more than half of the maximum number of informants which is 30; therefore have Rank-Order Priority (ROP) value 50 or more, while 72 species (57.6%) were unpopular, since they were cited by less than half of the maximum number of informants; therefore have ROP value less than 50. Eighty species (63.5%) have known toxicity in neighboring countries while 46 species (36.5%) have not such known toxicity. Major toxic effects cited by popular species have exhibited major symptoms ranging from skin irritation, gastric and abdominal disturbances, abortion, sterility, neuralgic pains including hysteria, and fatigue. The studied area has been shown a high level of toxic species diversity, since it is dominated by at least four phytogeographical elements, which requires certain ecological awareness to protect and reserve the wild and endemic species from further threats to enhance the sustainable development.

Keywords: Ethnobotany; Toxicology; Toxic plants; Active constituents; Flok medicine

Habiba Ramzan, Zubaida Yousaf,

Chapter 4 - Green fabrication of metallic nanoparticles,

Editor(s): Alexandru Mihai Grumezescu,

Inorganic Frameworks as Smart Nanomedicines,

William Andrew Publishing,

2018,

Pages 137-183,

ISBN 9780128136614,

https://doi.org/10.1016/B978-0-12-813661-4.00004-3.

(https://www.sciencedirect.com/science/article/pii/B9780128136614000043)

Abstract: Nanoparticles (NPs) are fabricated under the broad term nanobiotechnology and are of variable types, such as metallic and nonmetallic. The process of fabrication and the source of metallic NPs bring a lot of difference in the properties of NPs. Many different methodologies are examined under chemical and physical methods. But the revolutionary step was taken and biological methods were used, which have proved ecofriendly and more efficient. Metallic NPs, when synthesized by nonbiological resources have size ranging 50–450nm, whereas ecofriendly NPs exhibit sizes 5–100nm. The metallic NPs fabricated by green methods include iron, titanium, cadmium, cobalt, silver, gold and nickel. The methods by which metallic NPs are fabricated using green sources include phytological fabrication (plant), phycological fabrication (algae), mycological fabrication (fungi), and bacteriological fabrication. The metallic NPs fabricated by biological means exhibit particular attributes, as they have broad surface-to-volume ratio, spatial dispersion, and high surface energy. The green fabricated metallic NPs are ecofriendly and bear fine and versatile shapes and sizes. Applications of metallic NPs are widespread in electronics, medicine technology (MRI, chemotherapy, etc.), and pharmaceutical (drug production and drug carriers) applications.

Keywords: Ecofriendly; fabrication; metallic nanoparticles MNPs; nanoparticles NPs

Sérgio Roberto Rodrigues, Miguel Angel Morón, Elias Soares Gomes, José Maurício Simões Bento,

Morphology of immature stages and mating behavior in Liogenys fusca (Blanchard) (Coleoptera, Melolonthidae, Melolonthinae),

Revista Brasileira de Entomologia,

Volume 60, Issue 4,

2016,

Pages 284-289,

ISSN 0085-5626,

https://doi.org/10.1016/j.rbe.2016.06.005.

(https://www.sciencedirect.com/science/article/pii/S0085562616301054)

Abstract: Liogenys fusca is a rizophagous insect pest in various crops of economic importance in Brazil. Here we investigated the morphology of immature stages and mating behavior of this species. The redescription of the 3rd instar larvae of L. fusca in this work allows identification and registration of occurrence independently of adults, which occur sporadically in a certain period of the year. Male and female of L. fusca remained confined in the soil during the day and exited between 19:00 and 23:30h. The copulations occurred between 19:30 and 21:00h, and were characterized by a typical behavioral sequence. Copulation durations in L. fusca lasted on average 512.23s. Adults were observed feeding before the copulations on leaves and inflorescences of plant species belonging to the family Anacardiaceae, Myracrodruon urundeuva, Schinus terebinthifolius, Astronium fraxinifolium and Anacardium occidentale.

Keywords: Insect morphology; Rizophagous insect; Scarabaeoidea; Sexual behavior; White grub

Goffredo Filibeck, Marta G. Sperandii, Luca Bragazza, Alessandro Bricca, Stefano Chelli, Simona Maccherini, Camilla Wellstein, Antonio L. Conte, Marta Di Donatantonio, T'ai G.W. Forte, Lorenzo Lazzaro, Tania Macchiavelli, Samuele Maestri, Roberta Marchesini, Michela Marignani, Gabriele Midolo, Ludovica Oddi, Leonardo Rosati, Giulia Silan, Laura Cancellieri,

Competitive dominance mediates the effects of topography on plant richness in a mountain grassland,

Basic and Applied Ecology,

Volume 48,

2020,

Pages 112-123,

ISSN 1439-1791,

https://doi.org/10.1016/j.baae.2020.09.008.

(https://www.sciencedirect.com/science/article/pii/S1439179120300980)

Abstract: Small-scale landforms influence plant species richness, but their mechanisms and effects in semi-natural dry grasslands have been poorly investigated. In this study we compared vascular plant richness, species composition, plant traits, soil properties and biomass nutrient content of convex (hillocks) and concave (hollows) karst landforms in a mountain pasture of the Central Apennines (Italy), at a small spatial scale (1 m2 plots). We found hillocks had significantly higher species richness than hollows. On hillocks, smaller Specific Leaf Area and Lateral Width, together with greater allocation of resources to Below-Ground Organs, indicated lower water availability, whereas hollows had deeper (thus moister), more acidic and more fertile soils, with aboveground plant biomass displaying higher nutrient levels. Partial correlation and regression tree models suggested that fine-scale richness patterns were not directly determined by abiotic properties, but were rather the result of competition levels associated with the cover of Agrostis capillaris (=A. tenuis) – a calcifuge and drought-sensitive grass able to achieve dominance only in hollows. The higher functional convergence exhibited by hollows suggests that A. capillaris is a strong competitor both above- and below-ground, mediating the effects of topography by imposing a biotic filter. On hillocks, competition is released by lower levels of available soil water in summer and higher soil pH, resulting in higher species richness and a more functionally divergent assemblage.

Keywords: Biodiversity; Cover; Ecological filter; Functional trait; Landform; Secondary grassland; Soil nutrient

Gayoung Cho, Hyo-Min Park, Won-Mo Jung, Woong-Seok Cha, Donghun Lee, Younbyoung Chae,

Identification of candidate medicinal herbs for skincare via data mining of the classic Donguibogam text on Korean medicine,

Integrative Medicine Research,

Volume 9, Issue 4,

2020,

100436,

ISSN 2213-4220,

https://doi.org/10.1016/j.imr.2020.100436.

(https://www.sciencedirect.com/science/article/pii/S2213422020300688)

Abstract: Background

Korean cosmetics are widely exported throughout Asia. Cosmetics exploiting traditional Korean medicine lead this trend; thus, the traditional medicinal literature has been invaluable in terms of cosmetic development. We sought candidate medicinal herbs for skincare.

Methods

We used data mining to investigate associations between medicinal herbs and skin-related keywords (SRKs) in a classical text. We selected 26 SRKs used in the Donguibogam text; these referred to 626 medicinal herbs. Using a term frequency-inverse document frequency approach, we extracted data on herbal characteristics by assessing the co-occurrence frequencies of 52 medicinal herbs and the 26 SRKs.

Results

We extracted the characteristics of the 52 herbs, each of which exhibited a distinct skin-related action profile. For example Ginseng Radix was associated at a high-level with tonification and anti-aging, but Rehmanniae Radix exhibited a stronger association with anti-aging. Of the 52 herbs, 46 had been subjected to at least one modern study on skincare-related efficacy.

Conclusions

We made a comprehensive list of candidate medicinal herbs for skincare via data mining a classical medical text. This enhances our understanding of such herbs and will help with discovering new candidate herbs.

Keywords: Cosmetic development; Data mining; Skincare; Traditional herbal medicine

Yendube T. Kantati, K. Magloire Kodjo, Koffi S. Dogbeavou, David Vaudry, Jérôme Leprince, Messanvi Gbeassor,

Ethnopharmacological survey of plant species used in folk medicine against central nervous system disorders in Togo,

Journal of Ethnopharmacology,

Volume 181,

2016,

Pages 214-220,

ISSN 0378-8741,

https://doi.org/10.1016/j.jep.2016.02.006.

(https://www.sciencedirect.com/science/article/pii/S0378874116300502)

Abstract: Ethnopharmacological relevance

Neurological diseases are rising all around the world. In a developing country such as Togo, although plant-based medicines are the only means, still very little is known regarding the nature and efficiency of medicinal plants used by indigenous people to manage central nervous system (CNS) disorders.

Aim of the study

This study, an ethnobotanical survey, aimed to report plant species used in traditional medicine (TM) for the management of various CNS disorders in Togo.

Materials and methods

52 traditional actors (TA) including 33 traditional healers (TH) and 19 medicinal plant sellers (MPS) were interviewed, using a questionnaire mentioning informants' general data and uses of medicinal plants.

Results

The present study reports 44 medicinal plant species distributed into 26 families, mentioning scientific and common local names, plant organs used, preparation method, root of administration and putative applications.

Conclusion

It appears that there is a real knowledge on medicinal plants used for traditional treatment of CNS disorders in Togo and that the local flora abounds of potentially neuroactive plants which could be useful for the discovery of antipsychotic or neuroprotective molecules.

Keywords: Ethnopharmacological survey; Togo; Traditional medicine; CNS disorders

Al-Baraa El-Saied, Abass El-Ghamry, Om-Mohammed A. Khafagi, Owen Powell, Ramadan Bedair,

Floristic diversity and vegetation analysis of Siwa Oasis: An ancient agro-ecosystem in Egypt’s Western Desert,

Annals of Agricultural Sciences,

Volume 60, Issue 2,

2015,

Pages 361-372,

ISSN 0570-1783,

https://doi.org/10.1016/j.aoas.2015.10.010.

(https://www.sciencedirect.com/science/article/pii/S0570178315000470)

Abstract: The rapid development and expansion of modern irrigation schemes across arid environments have radically transformed both natural environments and existing agricultural systems over the past century. The consequences for natural and cultural values are often severe, but remain poorly documented for many regions. The present study describes the floristic diversity of an Oasis agro-ecosystem located in Egypt’s hyper-arid Western Desert. A total of 132 sites were chosen to represent the flora of Siwa Oasis agro-ecosystem and 154 species were recorded of which 52 were cultivated. Non-cultivated taxa consisted predominately of therophytes whereby the flora of Siwa is represented by monoregional, biregional and pluriregional elements as well as some cosmopolitan species. During field survey, 55 species were recorded for the first time suggesting the recent introduction of new weeds. Based on previous studies, 36 wetland and orchard species may have become locally extinct due to loss of habitat and extensive transformation of the Oasis agro-ecosystem. Although Siwa does not support any endemic species, this study documents a unique and complex agro-ecosystem shaped by natural and human agents over millennia. Descriptive floristic studies such as presented here are important records during a time of continuing and increasing change throughout arid regions of the world.

Keywords: Floristic diversity; Oasis; Desert reclamation; Environmental change; Siwa; Egypt

Appendix 1 - List of Crop and Other Nonweedy Plants Cited in Text, Alphabetized by Common Name11With some exceptions (e.g., clovers), plants are listed alphabetically the way the name is used in the text. That is, white ash is listed with those plants beginning with “w” rather than as “ash, white”.,

Editor(s): Robert L. Zimdahl,

Fundamentals of Weed Science (Fifth Edition),

Academic Press,

2019,

Pages 683-690,

ISBN 9780128111437,

https://doi.org/10.1016/B978-0-12-811143-7.15001-2.

(https://www.sciencedirect.com/science/article/pii/B9780128111437150012)

Usman Zulfiqar, Muhammad Farooq, Saddam Hussain, Muhammad Maqsood, Mubshar Hussain, Muhammad Ishfaq, Muhammad Ahmad, Muhammad Zohaib Anjum,

Lead toxicity in plants: Impacts and remediation,

Journal of Environmental Management,

Volume 250,

2019,

109557,

ISSN 0301-4797,

https://doi.org/10.1016/j.jenvman.2019.109557.

(https://www.sciencedirect.com/science/article/pii/S0301479719312757)

Abstract: Lead (Pb) is the second most toxic heavy metal after arsenic (As), which has no role in biological systems. Pb toxicity causes a range of damages to plants from germination to yield formation; however, its toxicity is both time and concentration dependent. Its exposure at higher rates disturbs the plant water and nutritional relations and causes oxidative damages to plants. Reduced rate of seed germination and plant growth under stress is mainly due to Pb interference with enzymatic activities, membrane damage and stomatal closure because of induction of absicic acid and negative correlation of Pb with potassium in plants. Pb induced structural changes in photosynthetic apparatus and reduced biosynthesis of chlorophyll pigments cause retardation of carbon metabolism. In this review, the noxious effects of Pb on germination, stand establishment, growth, water relations, nutrient uptake and assimilation, ultra-structural and oxidative damages, carbon metabolism and enzymatic activities in plants are reported. The Pb dynamics in soil rhizosphere and role of remediation strategies i.e. physical, chemical and biological to decontaminate the Pb polluted soils has also been described. Among them, biological strategies, including phytoremediation, microbe-assisted remediation and remediation by organic amendments, are cost effective and environmentally sound remedies for cleaning Pb contaminated soils. Use of organic manures and some agricultural practices have the potential to harvest better crops yield of good quality form Pb contaminated soils.

Keywords: Contamination; Toxicity; Chlorosis; Remediation; Biochar; Phytoremediation

Sami Asir Al-Robai, Haidar Abdalgadir Mohamed, Saad Mohammed Howladar, Abdelazim Ali Ahmed,

Vegetation structure and species diversity of Wadi Turbah Zahran, Albaha area, southwestern Saudi Arabia,

Annals of Agricultural Sciences,

Volume 62, Issue 1,

2017,

Pages 61-69,

ISSN 0570-1783,

https://doi.org/10.1016/j.aoas.2017.04.001.

(https://www.sciencedirect.com/science/article/pii/S0570178317300064)

Abstract: The aim of this work was to study the vegetation structure of Wadi Turbah Zahran, Albaha area, Saudi Arabia and some biodiversity indices. Paleontological Statistics (PAST) Software Package was used for data analysis. A total of 266 species (201 genera, 71 families) were identified and most of the species were herbs (87%). Therophytes (32.7%) and Chamaephytes (30.45%) were the most prominent groups. Asteraceae family was represented by the highest number of species (15.4%) followed by Poaceae (9.4%). Thirty-one families (43.7%) were represented by a single genus and species. Thirty-four species (12.8%) were common to all sites. The genus Solanum was the most speciose followed by Rumex. Most of the calculated indices showed variations among the sites. Shannon (3.71–4.06), Menhenick (2.271–4.746) and Chao-1 (106–319.6) diversity indices markedly varied among the sites while Simpson values (0.96–0.97) were almost the same. Berger-Parker values revealed the dominance of Hyparrhenia hirta in four sites. Beta diversity values indicated high diversity between site 1 and 4 and less diversity between site 1 and 6.

Keywords: Vegetation structure; Diversity indices; Wadi Turbah Zahran; Albaha; Saudi Arabia

S.S. Semenya, A. Maroyi,

Ethnobotanical survey of plants used by Bapedi traditional healers to treat tuberculosis and its opportunistic infections in the Limpopo Province, South Africa,

South African Journal of Botany,

Volume 122,

2019,

Pages 401-421,

ISSN 0254-6299,

https://doi.org/10.1016/j.sajb.2018.10.010.

(https://www.sciencedirect.com/science/article/pii/S0254629918308330)

Abstract: The present study explored the utilisation of medicinal plants by Bapedi traditional healers (THs) to treat and manage tuberculosis (TB) and its opportunistic infections in the three districts of Limpopo Province, South Africa. Data were gathered using a semi-structured questionnaire as a guide for conversation with 202 THs after obtaining informed consent. One hundred and eighty-four (n = 184) plant species distributed in 149 genera and 77 botanical families were used by these THs. Amongst the diverse botanical families noted, the Fabaceae (21 spp.), Asteraceae (12 spp.) and Malvaceae (11 spp.) were dominant. Remedies were predominantly prepared from roots (63.8%) and leaves (13.8%). Overall, a total of 275 recipes (71.2% = mono and 28.7% = poly), prepared chiefly via boiling (50.9%) and pounding (40.7%) were documented. The foremost methods of administering these formulae were orally (87.2%) and nasally (11.2%). The highest fidelity level value (100%) was recorded for Capparis tomentosa, Cassipourea garcini, Catha edulis, Citrullus lanatus, Combretum hereroense, Datura stramonium, Dicoma anomala, Diospyros lycioides, Enicostema axillare, Gossypium herbaceum, Solanum catombelense, Stylochaeton natalensis, Zingiber officinale and Ziziphus zeyheriana across the studied districts. Cryptocarya transvaalensis, Lasiosiphon caffer and Warburgia salutaris notched the highest integer according to use value index. In general, a large number of species used by Bapedi THs are, for the first time, documented as medicines for TB and its opportunistic infections. These new additions to the scientific literature emphasise the need to conduct more ethnobotanical studies focusing on TB.

Keywords: Bapedi; Limpopo Province; Traditional healers; Tuberculosis

Species index,

Editor(s): Valentí Rull,

Paleoecological Research on Easter Island,

Elsevier,

2020,

Pages 271-274,

ISBN 9780128227275,

https://doi.org/10.1016/B978-0-12-822727-5.09985-2.

(https://www.sciencedirect.com/science/article/pii/B9780128227275099852)

Sravani Karri, Sanjay Sharma, Ketan Hatware, Kiran Patil,

Natural anti-obesity agents and their therapeutic role in management of obesity: A future trend perspective,

Biomedicine & Pharmacotherapy,

Volume 110,

2019,

Pages 224-238,

ISSN 0753-3322,

https://doi.org/10.1016/j.biopha.2018.11.076.

(https://www.sciencedirect.com/science/article/pii/S0753332218345554)

Abstract: In the present scenario, obesity is a challenging health problem and its prevalence along with comorbidities are on the rise around the world. According to world health organization and organisation for economic co-operation and development epidemiology reports, overweight and obesity are the fifth foremost causes of deaths globally. The increasing rate of obesity is becoming a mammoth problem which enormously affects an individual’s quality of life. The conventional therapy of obesity mainly involves synthetic moieties and surgical procedures, which has many harmful side effects and chances of recurrence with severity. Hence, the Present review is a metanalysis of all the available data on the use of the plants with their biological source, active phytochemical constituents and a probable mechanism of action as natural anti-obesity agents. The metanalysis of data during the period of 2000–2018 was performed with the help of scientific data search engine National Center for Biotechnology Information (NCBI/PubMed). This data reveals the need and scope of further research in the development of new natural phytoconstituents for the management of obesity.

Keywords: Obesity; Phytoconstituents; Mechanism; Management; Natural products

M.Teresa Palmese, Rita E. Uncini Manganelli, Paolo E. Tomei,

An ethno-pharmacobotanical survey in the Sarrabus district (south-east Sardinia),

Fitoterapia,

Volume 72, Issue 6,

2001,

Pages 619-643,

ISSN 0367-326X,

https://doi.org/10.1016/S0367-326X(01)00288-X.

(https://www.sciencedirect.com/science/article/pii/S0367326X0100288X)

Abstract: The therapeutic uses and methods of administration of 70 plants in the traditional medicine of Sarrabus (south-east Sardinia, Italy) are documented. Among these species, some were not reported previously for Sardinia, while others turn out to have an original therapeutic use.

Keywords: Ethnobotany; Sardinia; Italy

Lala Nirina Ranjarisoa, Noëline Razanamihaja, Herintsoa Rafatro,

Use of plants in oral health care by the population of Mahajanga, Madagascar,

Journal of Ethnopharmacology,

Volume 193,

2016,

Pages 179-194,

ISSN 0378-8741,

https://doi.org/10.1016/j.jep.2016.07.076.

(https://www.sciencedirect.com/science/article/pii/S0378874116305062)

Abstract: Background

The use of medicinal plants to address oral health problems is not well documented in Madagascar, yet the country is full of endemic flora. The aim of this study was to collect information on the use of plants in the region of Mahajanga, Madagascar, for the treatments of oral diseases mainly tooth decay.

Methods

The ethnobotanical survey with respect to the use of plants for curing dental problems was carried out in 2012. A cluster sampling at three levels was applied when choosing the study sites. The target population was made up of heads of household. The following data were collected from a semi-structured questionnaire: name of plants, part used, mode of preparation, and administration. The Informant Consensus Factor and Fidelity Level indexes were calculated for each condition treated and used plants. The Results revealed that 93 per cent of the targeted population has used plants to calm dental pain, whereas 44.2% have reported using plants due to financial problems. About 65 species of plants are commonly used for oral health care and 63 of them treated caries. Cajanus cajan (L.) Millsp. was the most plant used. It was mostly used in crushed form of 5 to 9 leaves which were prepared and placed directly on the affected oral part or in the tooth cavity. In general, the treatment lasted about 5 days or minus. The ICF were 0.83 for caries and 0.81 for periodontal diseases.

Conclusion

This ethnobotanical survey will serve as database for further phytochemical and pharmacological study of plants in order to identify their active components and advise the population on the most effective administration.

Keywords: Ethnobotanical survey; Medicinal plants; Oral care; Mahajanga

I Blanckaert, R.L Swennen, M Paredes Flores, R Rosas López, R Lira Saade,

Floristic composition, plant uses and management practices in homegardens of San Rafael Coxcatlán, Valley of Tehuacán-Cuicatlán, Mexico,

Journal of Arid Environments,

Volume 57, Issue 2,

2004,

Pages 179-202,

ISSN 0140-1963,

https://doi.org/10.1016/S0140-1963(03)00100-9.

(https://www.sciencedirect.com/science/article/pii/S0140196303001009)

Abstract: Homegardens preserve much of the local cultural history and reveal information about plant management decisions by individual holders. A survey was conducted in the homegardens of San Rafael Coxcatlán, a rural village in the semi-arid valley of Tehuacán-Cuicatlán, Mexico. Two hundred and thirty three different plant species were collected in 30 studied homegardens. 65.7% were ornamental, 29.6% edible and 8.6% were medicinal. Sixty eight percent of the plants were cultivated, while 22% were spared and 10% protected. The results confirm that homegardens are rich in biodiversity and are interesting for ethnobotanical research. Moreover, they need to be considered for in situ conservation and development programs in future.

Keywords: Biodiversity; Ethnobotany; In situ conservation; Quantitative analysis; Rural development; Semi-arid

A.T. Mbaveng, V. Kuete,

Chapter 29 - Syzygium aromaticum,

Editor(s): Victor Kuete,

Medicinal Spices and Vegetables from Africa,

Academic Press,

2017,

Pages 611-625,

ISBN 9780128092866,

https://doi.org/10.1016/B978-0-12-809286-6.00029-7.

(https://www.sciencedirect.com/science/article/pii/B9780128092866000297)

Abstract: Syzygium aromaticum is a tree in the family Myrtaceae, native to Indonesia with the aromatic flower buds known as cloves, and commonly used as a spice. The plant is commercially harvested in Indonesia, as well as in India, Pakistan, Sri Lanka, Comoro Islands, Madagascar, Seychelles, and Tanzania. In the present chapter, we have reported the anticancer, antidiabetic antiinflammatory, antinociceptive, antibacterial, antifungal, antiprotozoal, antioxidant, and antithrombotic properties, as well as other biological activities and constituents of this plant. Due to its numerous pharmacological activities, S. aromaticum can be considered as a potential drug candidate for many ailments.

Keywords: Africa; clove; constituents; eugenol; pharmacology; Syzygium aromaticum

Francisco López-Muñoz, Cecilio Alamo, Pilar García-García,

“The herbs that have the property of healing…,”: The phytotherapy in Don Quixote,

Journal of Ethnopharmacology,

Volume 106, Issue 3,

2006,

Pages 429-441,

ISSN 0378-8741,

https://doi.org/10.1016/j.jep.2006.03.020.

(https://www.sciencedirect.com/science/article/pii/S0378874106001644)

Abstract: Don Quixote, the most outstanding novel of the Spanish literature, represents a documentary source widely used among those specialists who intend to deepen in the knowledge of the late Renaissance society. In this sense, Don Quixote has been also studied from a medical perspective, including a general therapeutical view (oils, ointments, balms, poultices, syrups and other pharmacy preparations). We have tackled Don Quixote from the phytotherapeutic and ethnopharmacological perspective, a barely explored field. In this work, we intend to study the medicinal plants used during the Cervantine time for the treatment de multiples diseases (sedatives like opium, laxatives and emetics like hellebore, tonics and irritants) and we analyze the specific herbal therapies (balms, purgatives and emetics, ointments and poultices), which Cervantes reveals to us in his novel. Among them, the rhubarb root (Rheum spp. or Rumex spp.) should be highlighted, as well as the seeds of gopher spurge (Euphorbia lathyris), chicory (Cichorium intybus) and rosemary (Rosmarinus officinalis), primary component of the famous Balsam of Fierabras. Also, we have examined the possible scientific influences, which might have inspired Cervantes in this field, mainly the work of Andrés Laguna (Dioscorides’ Materia Medica).

Keywords: Phytotherapy; History of pharmacology; Don Quixote

Roman Pavela, Angelo Canale, Heinz Mehlhorn, Giovanni Benelli,

Application of ethnobotanical repellents and acaricides in prevention, control and management of livestock ticks: A review,

Research in Veterinary Science,

Volume 109,

2016,

Pages 1-9,

ISSN 0034-5288,

https://doi.org/10.1016/j.rvsc.2016.09.001.

(https://www.sciencedirect.com/science/article/pii/S0034528816302934)

Abstract: Ticks transmit at least the same number or even more pathogens than any other group of blood-feeding arthropods worldwide affecting humans and animals. The eco-friendly control and management of tick vectors in a constantly changing environment is a crucial challenge. Besides the development of vaccines against ticks, IPM practices aimed at reducing tick interactions with livestock, emerging pheromone-based control tools, and few biological control agents, the extensive employment of acaricides and tick repellents still remain the most effective and ready-to-use strategies. However, the former is limited by the development of growing resistances as well as environmental concerns. Exploiting plants and plant products as sources of effective tick repellents and acaricides represents a promising strategy. In this scenario, the preservation of ethnobotanical information on repellent and acaricidal potential of plants is crucial. Here, we evaluated relevant information published in recent years, focused on plants used as repellents and acaricides against tick vectors in different regions worldwide. We selected a total of 238 plant species, which are traditionally used against ticks by native and local communities of Africa (Kenya, Uganda, Zimbabwe, South Africa), Europe (Serbia, Macedonia, Romania), Asia (Pakistan, India) and America (Brazil, Canada), from 56 families. However, only 7 families (i.e. Asteraceae, Euphorbiaceae, Fabaceae, Lamiaceae, Meliaceae, Apocynaceae and Solanaceae) represent the major quote (46%) of all plant species. We evaluated the differences in acaricidal and repellent efficacy of different formulations used. In the final section, implications arising from the surveyed anti-tick ethnobotanical knowledge and challenges for its future are discussed.

Keywords: Argasidae; DEET; Icaridin; Ixodidae; Lyme disease; Rickettsiales

Gaurav Pal, Priya Rai, Anjana Pandey,

Chapter 1 - Green synthesis of nanoparticles: A greener approach for a cleaner future,

Editor(s): Ashutosh Kumar Shukla, Siavash Iravani,

In Micro and Nano Technologies,

Green Synthesis, Characterization and Applications of Nanoparticles,

Elsevier,

2019,

Pages 1-26,

ISBN 9780081025796,

https://doi.org/10.1016/B978-0-08-102579-6.00001-0.

(https://www.sciencedirect.com/science/article/pii/B9780081025796000010)

Abstract: Nanotechnology, the study of matter at the nanoscale (i.e., between 1–100nm), has opened up novel dimensions in the field of biotechnology and nanomedicine, along with various other important applications such as drug delivery, electronics, cosmetics, and biosensors. Nanoparticles of varied shapes and sizes can be synthesized by using physical, chemical, or biological pathways. However, exploiting physical and chemical routes lead to high energy consumption, low yield, high cost, and environmental damage by employing harsh reducing agents. The biological pathways involve the use of microorganisms (bacteria, fungi, yeast, algae, etc.) or plants, and using microorganisms is riskier because of the pathogenicity issue; it also requires maintenance of large cultures. Therefore, synthesis of nanoparticles with greener methods is preferred. In this chapter, we present a generalized view of green synthesis for the generation of nanoparticles involving plants or their parts as a cost-effective, simpler, and eco-friendly approach. The various factors affecting the green synthesis of nanoparticles are also considered and explained.

Keywords: Green synthesis; Nanoparticles; Nanomedicine; Phytocompounds; Quantum confinement

Gustav Komlaga, Christian Agyare, Rita Akosua Dickson, Merlin Lincoln Kwao Mensah, Kofi Annan, Philippe M. Loiseau, Pierre Champy,

Medicinal plants and finished marketed herbal products used in the treatment of malaria in the Ashanti region, Ghana,

Journal of Ethnopharmacology,

Volume 172,

2015,

Pages 333-346,

ISSN 0378-8741,

https://doi.org/10.1016/j.jep.2015.06.041.

(https://www.sciencedirect.com/science/article/pii/S0378874115300064)

Abstract: Ethnopharmacological relevance

Ethnobotanical survey was performed to document medicinal plants employed in the management of malaria in the Bosomtwe and Sekyere East Districts of the Ashanti Region (Ghana), in comparison with the plant ingredients in herbal antimalarial remedies registered by the Ghana Food and Drug Administration.

Materials and methods

Two hundred and three (203) herbalists from 33 communities within the two districts were interviewed on medicinal plants they use to manage malaria. A literature search was made to determine already documented plants. In addition, 23 finished marketed herbal products indicated for the management of malaria were identified and their labels examined to find out which of the plants mentioned in our survey were listed as ingredients and whether these products are in anyway regulated.

Results

Ninety-eight (98) species of plants were cited for the management of malaria. In comparison with literature citations, 12 (12.2%) species were reported for the management of malaria for the first time and 20 (20.4%) others for the first time in Ghana. Twenty-three (23) finished marketed herbal antimalarial products examined contained aerial or underground parts of 29 of the plants cited in our survey as ingredients. Twenty-two (22) of these products have been registered by the Ghana Food and Drugs Authority, four (4) of which were included in the recommended herbal medicine list for treating malaria in Ghana.

Conclusion

This study provides new additions to the inventory of medicinal plants used for the management of malaria and reports the commercial availability and regulation of finished marketed labelled herbal products intended for the treatment of malaria in Ghana.

Keywords: Malaria; Antimalarial; Treatment; Medicinal plants; Finished marketed herbal products

Teresa Tuttolomondo, Mario Licata, Claudio Leto, Giuseppe Bonsangue, Maria Letizia Gargano, Giuseppe Venturella, Salvatore La Bella,

Popular uses of wild plant species for medicinal purposes in the Nebrodi Regional Park (North-Eastern Sicily, Italy),

Journal of Ethnopharmacology,

Volume 157,

2014,

Pages 21-37,

ISSN 0378-8741,

https://doi.org/10.1016/j.jep.2014.08.039.

(https://www.sciencedirect.com/science/article/pii/S0378874114006333)

Abstract: Ethnopharmacological relevance

The area of the Nebrodi Regional Park (North-Eastern Sicily, Italy) has been quantitatively investigated in an ethnobotanical study for the first time. A total of 90 wild species are used for medicinal purposes and the uses of 5 species have not previously been reported in ethnobotanical studies in Italy (e.g., the use of Arisarum vulgare O. Targ. Tozz. for the treatment of rheumatic pains, the use of Silene vulgaris (Moench) Garcke for the treatment of liver diseases). The aim of this paper was to understand to what extent current knowledge on the medicinal use of plants is still an element of the culture within the rural populations of the Nebrodi Park. Materials and methods Semi-structured interviews were carried out in the local communities within the Nebrodi Regional Park with local people retained experts in rural traditions. A total of 226 people over the age of 60 were interviewed. Local plant uses were evaluated using ethnobotanical indices (e.g., cultural importance index, index of ethnobotanical diversity, informant consensus factor) and then compared with uses in other localities in Sicily, Italy and the Mediterranean area. Results Local communities use a total number of 90 wild species belonging to 44 plant families as medicinal remedies. The majority of the species are used as treatments against gastrointestinal system. The cultural importance index showed that Malva sylvestris (1.31) and Clinopodium nepeta (0.86) are the most important species to the Nebrodi area in terms of medicinal use. The use of Brassica rupestris Raf. for therapeutic purposes is limited to Sicily and it is an innovative finding of this study. Conclusions The research shows an ongoing process of cultural erosion in an advanced stage, but quantitative indices still highlight only for those species was a natural remedy deemed highly effective.

Keywords: Mediterranean folk medicine; Sicily; Nebrodi Regional Park; Brassica rupestris Raf.; Ethnobotanical indices; Cultural erosion

Kanika Patel, Vikas Kumar, Mahfoozur Rahman, Amita Verma, Dinesh Kumar Patel,

New insights into the medicinal importance, physiological functions and bioanalytical aspects of an important bioactive compound of foods ‘Hyperin’: Health benefits of the past, the present, the future,

Beni-Suef University Journal of Basic and Applied Sciences,

Volume 7, Issue 1,

2018,

Pages 31-42,

ISSN 2314-8535,

https://doi.org/10.1016/j.bjbas.2017.05.009.

(https://www.sciencedirect.com/science/article/pii/S2314853517301415)

Abstract: Herbal medicines have been used for the treatment of various disorders in the world since very early age. A large number of phytochemicals have been derived from the natural sources in the form of food supplement, neutraceuticals, oils and colour pigments. Flavonoids are a widely distributed group of phytochemicals having benzo-pyrone nucleus. Till so far more than 4000 different types of flavonoids have been described and categorized into different groups like flavonols, flavones, flavanones, isoflavones, catechins and anthocyanidins. Flavonoids have an attractive candidate in the food industry as natural colorants due to its health beneficial effect and safety issue. Hyperin, an important flvonoid were found in the different plants have various pharmacological activities like antioxidant, anti-inflammatory, anticancer, antiviral, antibacterial, antiparasitic, cardioprotective, hepatoprotective and antispasmodic. The aim of this review is to collect all the valuable scientific information of hyperin regarding its medicinal importance, pharmacological activities, isolation and analytical techniques. From the presented data’s in this review it will be beneficial to the scientific person, pharmaceutical manufacturer and consumers in order to it health beneficial effect of hyperin. Thus this valuable information will be helpful to the researchers for developing alternative methods of treatment for different diseases.

Keywords: Analytical techniques; Flavonoid; Hyperin; Isolation techniques; Pharmacological activities; Phytoconstituents

Pamela K. Anderson, Andrew A. Cunningham, Nikkita G. Patel, Francisco J. Morales, Paul R. Epstein, Peter Daszak,

Emerging infectious diseases of plants: pathogen pollution, climate change and agrotechnology drivers,

Trends in Ecology & Evolution,

Volume 19, Issue 10,

2004,

Pages 535-544,

ISSN 0169-5347,

https://doi.org/10.1016/j.tree.2004.07.021.

(https://www.sciencedirect.com/science/article/pii/S0169534704002186)

Abstract: Emerging infectious diseases (EIDs) pose threats to conservation and public health. Here, we apply the definition of EIDs used in the medical and veterinary fields to botany and highlight a series of emerging plant diseases. We include EIDs of cultivated and wild plants, some of which are of significant conservation concern. The underlying cause of most plant EIDs is the anthropogenic introduction of parasites, although severe weather events are also important drivers of disease emergence. Much is known about crop plant EIDs, but there is little information about wild-plant EIDs, suggesting that their impact on conservation is underestimated. We conclude with recommendations for improving strategies for the surveillance and control of plant EIDs.

Efraim Lev, Zohar Amar,

Reconstruction of the inventory of materia medica used by members of the Jewish community of medieval Cairo according to prescriptions found in the Taylor–Schechter Genizah collection, Cambridge,

Journal of Ethnopharmacology,

Volume 108, Issue 3,

2006,

Pages 428-444,

ISSN 0378-8741,

https://doi.org/10.1016/j.jep.2006.06.005.

(https://www.sciencedirect.com/science/article/pii/S0378874106002960)

Abstract: The Taylor–Schechter (T–S) collection at Cambridge University Library is the biggest of all Cairo Genizah collections in the world. The importance and the potential of research into the medical aspects of the Genizah documents were clear to researcher since the early 1960s. A few works have been published since, usually focusing on one subject, or even important single manuscripts. The current research concerned mainly with one aspect of the history of medicine of the Jewish community of Cairo (as a reflection of Eastern medieval societies), namely the practical uses of natural substances for medicine. The most interesting and original information is undoubtedly to be found in the 141 prescriptions, as they reflect the medical reality that actually existed. And indeed, 242 substances were recorded in the prescriptions identified: 195 substances of plants origin (80.6%), 27 inorganic materials (11.2%) and 20 substances of animal origin (8.2%) were recorded as being in practical used for medicinal purposes. The most frequently mentioned substances were the rose, myrobalan, sugar, almonds, and endive. The most prevalent ailments: eye diseases, headache, constipations (purgative), cough, skin diseases, stomach, fever, gynaecological problems, haemorrhoids, liver ailments, lice, swellings, dental trouble, ulcers, and problems of the urinary tract.

Keywords: Genizah; Cairo; Jewish community; Materia medica; Prescription; Ethnopharmacology; Mediterranean; Medieval

Efraim Lev,

Reconstructed materia medica of the Medieval and Ottoman al-Sham,

Journal of Ethnopharmacology,

Volume 80, Issues 2–3,

2002,

Pages 167-179,

ISSN 0378-8741,

https://doi.org/10.1016/S0378-8741(02)00029-6.

(https://www.sciencedirect.com/science/article/pii/S0378874102000296)

Abstract: This article presents the results of a study of the medicinal uses of natural substances in medieval and Ottoman al-Sham (the Levant). It involved a meticulous survey of a wide range of historical sources spanning approximately 1100 years and including medical and pharmacological literature, travelogues, geographical and agricultural literature, dictionaries, archives, the Genizah and other medieval sources. Our main goal was to arrive at a reconstruction of the unwritten materia medica of the medieval and Ottoman Levant. Of the many and varied medicinal substances on which we were able to extract information, we were able to identify 286. These are presented according to the following classification: 234 species of plants (81.8%); 27 species of animals (9.5%); 15 kinds of minerals (5.2%) and 10 substances of other or mixed origin (3.5%). Analysis of the data showed that the region under study served as the geographic origin of the majority of the substances, only a minority of the materials was imported. The main reason for this is the geographic location of the Levant as a junction between three continents, as a cultural meeting point and as trade center. Finally, our data revealed that the al-Sham region was an independent source of production and marketing of medicinal substances during the medieval and Ottoman periods.

Keywords: materia medica; Medicinal substances; Medieval; Ottoman; al-Sham; Levant

P.B. Yazbek, P. Matta, L.F. Passero, G.dos Santos, S. Braga, L. Assunção, T. Sauini, F. Cassas, R.J.F. Garcia, S. Honda, E.H.P. Barreto, E. Rodrigues,

Plants utilized as medicines by residents of Quilombo da Fazenda, Núcleo Picinguaba, Ubatuba, São Paulo, Brazil: A participatory survey,

Journal of Ethnopharmacology,

Volume 244,

2019,

112123,

ISSN 0378-8741,

https://doi.org/10.1016/j.jep.2019.112123.

(https://www.sciencedirect.com/science/article/pii/S0378874118342144)

Abstract: Ethnopharmacological relevance

Participatory research can help to broaden the understanding of medical systems and beliefs of traditional communities. An ethnopharmacological survey in collaboration with local people focused on plants used in quilombos located in Southeast Region in Brazil identified cultural factors that influence plant and recipe choice.

Aim of the study:

To investigate the factors related to the therapeutic efficiency of medicinal plants from the perspective of Quilombo da Fazenda residents.

Materials and methods

University researchers collaborated with community residents for both aims and methods of the study. The local partners were trained in the gathering of ethnopharmacological data and then selected and interviewed the residents considered experts on the use of medicinal plants. Data on the use of each species were supported by voucher specimens collected by the local partners and university researchers. Participant observations and field diaries by the university researchers supplemented the data.

Results

Eight interviewees mentioned 92 medicinal species with 60 therapeutic uses, applied in 208 recipes or remedies. Asteraceae (13 species), Lamiaceae (5) and Urticaceae (5) contributed most medicinal plant species. Of the 12 etic categories of use, the circulatory system category had the highest number of plants mentioned. Decoction was the most commonly used preparation method (66.8%), and most remedies were administered orally (76.4%). Eighty-six recipes included more than one plant species and/or the addition of other components, such as sugar, salt or animal products. Several cultural factors influence medicinal plant use. Popular beliefs on the quality of blood or the humoral properties of plants and illnesses, characteristics of the plants and other factors determine which plant is used and why.

Conclusions

The participatory method identified a large number of factors that influence medicinal plant use: the patient's blood type; the condition of the plant and the disease (hot-cold system); the route of administration and dosage; the preventive uses of the plants; and the influence of other factors, such as the sun, the moon and dew. The participatory approach is useful for gaining insight on the decision processes of medicinal plant use in traditional societies, and also for those communities wanting to document their knowledge with or without the participation of the academy.

Keywords: Participatory ethnobotany; Medicinal plants; Local knowledge; Quilombolas; Atlantic forest

Johan Edqvist, Elin Rönnberg, Sara Rosenquist, Kristina Blomqvist, Lenita Viitanen, Tiina A. Salminen, Matts Nylund, Jessica Tuuf, Peter Mattjus,

Plants Express a Lipid Transfer Protein with High Similarity to Mammalian Sterol Carrier Protein-2*,

Journal of Biological Chemistry,

Volume 279, Issue 51,

2004,

Pages 53544-53553,

ISSN 0021-9258,

https://doi.org/10.1074/jbc.M405099200.

(https://www.sciencedirect.com/science/article/pii/S0021925818819986)

Abstract: This is the first report describing the cloning and characterization of sterol carrier protein-2 (SCP-2) from plants. Arabidopsis thaliana SCP-2 (AtSCP-2) consists of 123 amino acids with a molecular mass of 13.6 kDa. AtSCP-2 shows 35% identity and 56% similarity to the human SCP-2-like domain present in the human D-bifunctional protein (DBP) and 30% identity and 54% similarity to the human SCP-2 encoded by SCP-X. The presented structural models of apo-AtSCP-2 and the ligand-bound conformation of AtSCP-2 reveal remarkable similarity with two of the structurally known SCP-2s, the SCP-2-like domain of human DBP and the rabbit SCP-2, correspondingly. The AtSCP-2 models in both forms have a similar hydrophobic ligand-binding tunnel, which is extremely suitable for lipid binding. AtSCP-2 showed in vitro transfer activity of BODIPY-phosphatidylcholine (BODIPY-PC) from donor membranes to acceptor membranes. The transfer of BODIPY-PC was almost completely inhibited after addition of 1-palmitoyl 2-oleoyl phosphatidylcholine or ergosterol. Dimyristoyl phosphatidic acid, stigmasterol, steryl glucoside, and cholesterol showed a moderate to marginal ability to lower the BODIPY-PC transfer rate, and the single chain palmitic acid and stearoyl-coenzyme A did not affect transfer at all. Expression analysis showed that AtSCP-2 mRNA is accumulating in most plant tissues. Plasmids carrying fusion genes between green fluorescent protein and AtSCP-2 were transformed with particle bombardment to onion epidermal cells. The results from analyzing the transformants indicate that AtSCP-2 is localized to peroxisomes.

James G. Speight,

Chapter 7 - Hydrocarbons from biomass,

Editor(s): James G. Speight,

Handbook of Industrial Hydrocarbon Processes (Second Edition),

Gulf Professional Publishing,

2020,

Pages 293-342,

ISBN 9780128099230,

https://doi.org/10.1016/B978-0-12-809923-0.00007-2.

(https://www.sciencedirect.com/science/article/pii/B9780128099230000072)

Abstract: Abstracts

Biomass is the detritus or remains of living and recently dead biological material which can be used as fuel or for industrial production. Biomass also refers to (i) energy crops grown specifically to be used as fuel, such as fast-growing trees or switch grass, (ii) agricultural residues and byproducts, such as straw, sugarcane fiber, and rice hulls, and (iii) residues from forestry, construction, and other wood-processing industries. Biomass is a renewable energy source unlike other resources such as crude oil, natural gas, tar sand, coal, and oil shale which can be depleted with time and may have up to 50 years of use at current rates of depletion of these resources. Agricultural products specifically grown for biofuel production include crops such as corn, soybeans, rapeseed, wheat, sugar beet, sugar cane, palm oil, and Jatropha oil, as well as wood. This chapter describes the production of biofuels to replace oil and natural gas as sources of hydrocarbon derivatives and hydrocarbon fuels is in active development, focusing on the use of cheap organic matter (usually cellulose, agricultural and sewage waste) in the efficient production of liquid and gas biofuels which yield high net energy. One advantage of biofuel over most other fuel types is that it is biodegradable, and so relatively harmless to the environment if spilled.

Keywords: Biochemical conversion; Biomass feedstocks; Biorefining; Carbohydrates; Hydrocarbon from nonwoody plants; Hydrocarbons by anaerobic digestion; Hydrocarbons via synthesis gas; Naturally occurring hydrocarbons; Plant fibers; Production of hydrocarbons; Steroids; Vegetable oils; Waste

D. Priyamka Sreekeesoon, M. Fawzi Mahomoodally,

Ethnopharmacological analysis of medicinal plants and animals used in the treatment and management of pain in Mauritius,

Journal of Ethnopharmacology,

Volume 157,

2014,

Pages 181-200,

ISSN 0378-8741,

https://doi.org/10.1016/j.jep.2014.09.030.

(https://www.sciencedirect.com/science/article/pii/S0378874114006849)

Abstract: Ethnopharmacological relevance

Pain is a multi-faceted and multi-factorial condition which is challenging to manage and treat. Conventional therapies such as analgesics, Non-steroidal anti-inflammatory drugs (NSAIDs), and corticosteroids amongst others have been successful to some extent in its management and treatment. Nonetheless, such therapies tend to be accompanied by undesirable effects and have a limited therapeutic range. Consequently, there is a pressing need to probe for novel analgesic and anti-nociceptive drugs from traditional medicines (TM). This study was designed to record, document and analyze herbal and animal-based therapies used for the management and treatment of pain in the tropical of Mauritius.

Materials and methods

Data was collected via face-to-face interviews with TM users (n=332) and practitioners (n=20). Seven quantitative ethnopharmacological indexes, namely family use value (FUV), use value (UV), informant agreement ratio (IAR), relative frequency of citation (RFC), fidelity level (FL), relative importance (RI) and ethnobotanicity index (EI) were calculated.

Results

A total of 79 plant species distributed within 40 families and 20 polyherbal preparations was recorded. Interestingly, 6 indigenous/endemic plants have been reported for the first time to be in common use for pain management and treatment in Mauritius. The most significant biologically important plant family was Xanthorrhoeaceae with highest FUV. The species which ranked highest according to its UV was Morinda citrifolia L. Morinda citrifolia L. and Ricinus communis L. also scored the highest RFC. The IAR values for the disease categories were high (0.95–0.97). Based on EI, plants species which are known to be useful in TM accounted for 11.5% of the total flora in Mauritius. Coix lacryma-jobi L. (FL=100%) had highest FL for lower back ache. Morinda citrifolia L. scored highest on most of the quantitative indices calculated including RI, which is endorsed by extensive documentation on its versatility and particularly its anti-nociceptive properties. Seven animal species were recorded to be in common use.

Conclusion

The present ethnopharmacological study revealed a panoply of TM to be in common use for pain management and treatment in Mauritius. This study has documented for the first time medicinal plants and animal species with potential analgesic and/or anti-nociceptive properties. This study has therefore provided important baseline primary data for the discovery of new lead molecules for drug development geared towards pain management and treatment.

Keywords: Traditional medicines; Pain; Herbal remedies; Zootherapy; Mauritius

L.V.Y. Weerarathne, B. Marambe, Bhagirath S. Chauhan,

Intercropping as an effective component of integrated weed management in tropical root and tuber crops: A review,

Crop Protection,

Volume 95,

2017,

Pages 89-100,

ISSN 0261-2194,

https://doi.org/10.1016/j.cropro.2016.08.010.

(https://www.sciencedirect.com/science/article/pii/S0261219416302101)

Abstract: Integrated weed management (IWM) strategies composed of non-chemical methods provide many beneficial effects to the agro-ecosystems, including growth and development of crops. The focus of this review is to explore the potential of intercropping as a non-chemical weed management technique in IWM in selected tropical root and tuber crops namely, sweet potato, yam, and cocoyam. Fast-growing and short-duration crops are suggested to be intercropped with root and tuber crops cautiously to achieve high intercrop productivity in such systems. Yam-pumpkin intercrop has reduced weed growth by 70% and increased the yield of component crops by 30–50% whereas sweet potato-maize-cocoyam intercrop has reported a 50–90% of yield depression in component crops in the absence of weed control measures. This signifies the importance of selecting a spatially and temporally compatible intercrop combination for weed control and higher yields of component crops in an intercrop. Sweet potato-maize-soybean system, sweet potato-peanut system, yam-fluted pumpkin/melon-okra/maize system and sun hemp-taro system have shown evidence to be compatible within each system and resulted in better weed control and higher crop yields. It is recommended to plant sweet potato at a density of 33,333 plants ha−1 with peanut at a density of 66,667 plants ha−1 for the lowest weed density and the highest yield advantage whereas ideal density levels of selected intercrops are yet to be researched in most of the intercropping systems with root and tuber crops. A limited number of researches have been successfully conducted to find out compatibility levels of intercrops focusing on the degree of weed management and yield advantage of sweet potato-, yam-, and cocoyam-based intercrops. Hence, further research is mandatory prior to endorsing intercropping as an effective weed management technique in root and tuber crops.

Keywords: Sweet potato; Yam; Cocoyam; Intercropping; Compatibility; Weed control

Zhen Li, Vera Pospelova, Hui-Ling Lin, Lejun Liu, Bing Song, Wenping Gong,

Seasonal dinoflagellate cyst production and terrestrial palynomorph deposition in the East Asian Monsoon influenced South China Sea: A sediment trap study from the Southwest Taiwan waters,

Review of Palaeobotany and Palynology,

Volume 257,

2018,

Pages 117-139,

ISSN 0034-6667,

https://doi.org/10.1016/j.revpalbo.2018.07.007.

(https://www.sciencedirect.com/science/article/pii/S0034666717302063)

Abstract: The South China Sea (SCS), influenced by the tropical East Asian Monsoon (EAM), experiences winter monsoon and summer monsoon shifts in near-surface wind, air temperature, sea-surface temperature, salinity, primary productivity, and other oceanographic conditions. To understand how monsoon seasons influence palynological dynamics and which palynological index could be a reliable indicator of winter or summer monsoons, we studied palynological records of sediment trap samples collected in March–April (winter monsoon season) and July–August (summer monsoon season). Fluxes and assemblages of terrestrial pollen and spores, as well as marine dinoflagellate cysts, were investigated using sediment traps in the southwest Taiwan waters of the SCS. The pollen and spores of 109 taxa and dinoflagellate cysts of 53 taxa were identified in 24 sediment trap samples that were collected at 3-day intervals. The average abundance of Pinus pollen was notably higher in March–April at ~40%, which was double that in July–August. This trend was associated with transport by the northwest wind in March–April when Pinus pollen are produced by the coniferous vegetation in the South China and Taiwan Island. The pollen abundances of Chenopodiaceae/Amaranthaceae and Compositae seemed to be greatly reduced in July–August, with an opposite pattern observed for Poaceae, Artemisia, Cyperaceae, Typha, and fern spores. Fluvial transport is likely the controlling factor since river runoff intensifies in summer. High relative abundances and fluxes of Poaceae pollen are not indicators of summer monsoons but related to cultivated activities. Dinoflagellate cyst assemblages were dominated by cysts produced by heterotrophic taxa, especially Brigantedinium spp. at 68–91%. Proportionally higher increases than those of cysts produced by autotrophic dinoflagellates resulted in lower heterotrophic to autotrophic ratios in July–August. The highest total cyst fluxes occurred in late July of ~20,000 cysts m-2 d-1 together with the highest fluxes of autotrophic Operculodinium centrocarpum, Spiniferites spp., and Lingulodinium hemicystum. The increased fluxes of almost all dinoflagellate cyst taxa during July–August were related to the decreased sea-surface salinity (SSS) due to the greater river water inputs and nutrients.

Keywords: Palynology; Dinoflagellate cysts; East Asian monsoon; South China Sea; Sediment trap; Seasonal change

F.U. Afifi, B. Abu-Irmaileh,

Herbal medicine in Jordan with special emphasis on less commonly used medicinal herbs,

Journal of Ethnopharmacology,

Volume 72, Issues 1–2,

2000,

Pages 101-110,

ISSN 0378-8741,

https://doi.org/10.1016/S0378-8741(00)00215-4.

(https://www.sciencedirect.com/science/article/pii/S0378874100002154)

Abstract: The use of herbal medicine in Jordan is very common. In order to evaluate the knowledge and the attitudes of the herbalists and acquire the common and less common medicinal herbs sold by the Jordanian herbalists, more than 100 herbalists throughout the country were interviewed and data concerning herbs present in their shops, the herbalists’ recommendations and other observations were collected. The present paper reports on the less common herbs and problems observed dealing with them.

Keywords: Herbal medicine; Less common herbs; Jordan

Mona Nazish Rehman, Mushtaq Ahmad, Shazia Sultana, Muhammad Zafar, Sarah Edwards,

Relative popularity level of medicinal plants in Talagang, Punjab Province, Pakistan,

Revista Brasileira de Farmacognosia,

Volume 27, Issue 6,

2017,

Pages 751-775,

ISSN 0102-695X,

https://doi.org/10.1016/j.bjp.2017.09.004.

(https://www.sciencedirect.com/science/article/pii/S0102695X17302053)

Abstract: This is the first ethnobotanical exploration with the aim to document the traditional medicinal usage of plants with the therapeutic values in Tehsil Talagang of Punjab province, Pakistan. The study shows the dependence of local people on medicinal plants in their daily life and provides practical evidence regarding the traditional usage of medicinal plants in health care practices. A total of 196 respondents including residents of the study area with gender representation and traditional healers were interviewed by using visual appraisal approach and rapid rural appraisal methods along semi-structured interviews and open-ended questionnaire. The data was quantitatively analyzed by using quantitative indices like use value, the relative frequency of citation, informant consensus factor, fidelity level and relative importance. A comparison with 25 published ethnobotanical and pharmacological studies was carried out to authenticate the ethnomedicinal relevance of the data recorded. The ethnomedicinal practices of 101 medicinal plants belonging to 36 families were reported. The results indicated that the dominant family was Brassicaceae (nine species). Herbs (57%) were the most dominant life form and leaves (29%) were the frequently used plant part with 45 reports. Mentha arvensis was found as highly cited plant species by respondents. The highest informant consensus factor value (0.65) was found for gastrointestinal disease category. There are 25 plant species having 100% fidelity level value. Use value and relative frequency of citation ranges from 0.04 to 0.16 and 0.15 to 0.36, respectively. The majority of the plant species were found to have strong pharmacological evidence. The current study will provide the basis for the preservation of ethnomedicinal heritage, knowledge and practices as well as for the further scientific investigations regarding the development of new herbal drugs.

Keywords: Ethnobotany; Medicinal plants; Talagang; Punjab; Pakistan

S. Prabhu, S. Vijayakumar, J.E. Morvin Yabesh, K. Ravichandran, B. Sakthivel,

Documentation and quantitative analysis of the local knowledge on medicinal plants in Kalrayan hills of Villupuram district, Tamil Nadu, India,

Journal of Ethnopharmacology,

Volume 157,

2014,

Pages 7-20,

ISSN 0378-8741,

https://doi.org/10.1016/j.jep.2014.09.014.

(https://www.sciencedirect.com/science/article/pii/S0378874114006680)

Abstract: Ethnopharmacological relevance

The aim of the present study was to document the medicinal plants by the traditional medical practitioners from Kalrayan hills of Villupuram district in Tamil Nadu, India. Quantitatively analyses of the data were made to acquire some useful leads for further studies.

Materials and methods

Successive free listing was the method adopted for the interview. In this study, 54 traditional healer medical practitioners were included and their knowledge on medicinal plants was gathered. The data were assessed with the help of two indices viz., informant consensus factor (Fic) and Informant Agreement on Remedies (IAR).

Results

The present survey is in accordance with some of the aspects of our previous surveys. Regarding the demography of the informants, it exhibited unevenness in male–female ratio and majority of the informants were poorly educated. Practicing this system of medicine as part time job by majority of the informants might indicate the reduced social status of this medicinal system. The present study had recorded the usage of 81 species, which in turn yielded 1073 use reports. The major illness category ‘aphrodisiac, hair care and endocrinal disorders’ hold a high Fic values. Among the other illness categories, gastro-intestinal ailments, genito-urinary ailments and dermatological infection ailments have a high percentage of use reports. Eye ailments, general health, kapha ailments, psychological ailments and skeleton muscular system ailments were the other illness categories with high Fic values. Some of the claims viz., Argyrolobium roseum (aphrodisiac ailments), Rosa brunonii (eye ailments) Hibiscus surattensis (dermatological infections ailments), Bauhinia variegata (neurology Ailments), Cotinus coggygria (circulatory system/cardiovascular ailments) and Uvaria narum (gastro-intestinal ailments) which have relatively high consensus can be taken up for further biomedical studies, since no substantial studies have been conducted on them.

Conclusion

Based on the results of our present study, we have highlighted some claims which are at high use in the study area but having little scientific support. Studies on such claims will provide scientific base to some extent which in turn will be useful to improve the health of indigenous people.

Keywords: Traditional healer practitioners; Kalrayan hills; Villupuram district; Tamil Nadu; India

Index,

Editor(s): L.P. Awasthi,

Applied Plant Virology,

Academic Press,

2020,

Pages 797-821,

ISBN 9780128186541,

https://doi.org/10.1016/B978-0-12-818654-1.00063-3.

(https://www.sciencedirect.com/science/article/pii/B9780128186541000633)

K. Vijayaraghavan, T. Ashokkumar,

Plant-mediated biosynthesis of metallic nanoparticles: A review of literature, factors affecting synthesis, characterization techniques and applications,

Journal of Environmental Chemical Engineering,

Volume 5, Issue 5,

2017,

Pages 4866-4883,

ISSN 2213-3437,

https://doi.org/10.1016/j.jece.2017.09.026.

(https://www.sciencedirect.com/science/article/pii/S2213343717304645)

Abstract: Nanoparticles exhibit unique properties that enable them to find potential applications in various fields. Accordingly, significant research attention is being given to the development of novel strategies for the synthesis of nanoparticles. Among these, biological route of nanoparticle synthesis has been portrayed as an efficient, low-cost and environmental friendly technique. Biological materials such as bacteria, fungi, yeast, algae and plant have been reported to possess high bioreduction ability to synthesize various size and shape of metallic nanoparticles. Of these biomaterials, this review focuses on plant-mediated biosynthesis of metallic nanoparticles. The biomolecules present in the plants such as terpenoids, flavones, ketones, aldehydes, proteins, amino acids, vitamins, alkaloids, tannins, phenolics, saponins, and polysaccharides play a vital role in reduction of metals. A systematic comparison of literature, based on the bioreduction capacity of various plant biomass/extract towards various metals under different experimental conditions, is also provided. Various instrumental techniques utilized to characterize nanoparticles are also discussed. Finally, this review also highlights the application of biosynthesized nanoparticles in different fields such as medicine, agriculture, catalytic, cosmetic and food. Thus, this article reviews the achievements and current status of plant-mediated biosynthesis, and hopes to provide insights into this exciting research frontier.

Keywords: Biosynthesis; Nanotechnology; Green chemistry; Biosorption; Plant biotechnology

Duncan McCollin, Linda Moore, Tim Sparks,

The flora of a cultural landscape: environmental determinants of change revealed using archival sources,

Biological Conservation,

Volume 92, Issue 2,

2000,

Pages 249-263,

ISSN 0006-3207,

https://doi.org/10.1016/S0006-3207(99)00070-1.

(https://www.sciencedirect.com/science/article/pii/S0006320799000701)

Abstract: In a novel approach to the use of archive biological information, qualitative vascular plant abundance categories from the 1930 Flora of Northamptonshire were matched with quantitative distribution data from the 1995 Flora in order to reveal the species that have changed in commonness relative to their pre-1930 populations. Factors thought to be responsible for these changes were analysed by investigating differences in habitat preference, dispersal ability and climate change indicators, using information from published sources. Changes in status were most dramatic for plant species associated with arable, wetland and woodland habitats. A highly significant factor was the trophic status of preferred habitats: burgeoning species were associated with higher soil nitrogen levels. In addition, evidence for the effects of habitat fragmentation were detected both in terms of changes in abundance being correlated with dispersal-related ecological characteristics and to the number of habitats with which particular species were associated. The discussion concerns the changing land use and farming practices in the county of Northamptonshire, which are widely held to be linked with a deterioration in plant biodiversity since 1930.

Keywords: Land use change; Habitat fragmentation; Eutrophication; Plants; Ellenberg indicators

A. Moteetee, R.O. Moffett, L. Seleteng-Kose,

A review of the ethnobotany of the Basotho of Lesotho and the Free State Province of South Africa (South Sotho),

South African Journal of Botany,

Volume 122,

2019,

Pages 21-56,

ISSN 0254-6299,

https://doi.org/10.1016/j.sajb.2017.12.012.

(https://www.sciencedirect.com/science/article/pii/S0254629917313595)

Abstract: This review is a comprehensive compilation of the ethnobotany of the Basotho (residing both in Lesotho and the Free State Province of South Africa) in its entirety. It includes all the known traditional uses ranging from food, through clothing, crafts, and textiles, to medicine and magic and is based on an extensive literature survey of both published and unpublished data from books, journals, dissertations, theses, available online databases, as well as past experiences of the authors. A total of 712 species is utilised for various purposes, comprising 22 pteridophytes and 690 flowering plants (509 dicotyledons, and 181 monocotyledons). The Asteraceae, with 115 species is the most commonly used family, followed by Poaceae (61) and Fabaceae (50). The largest number of plants (437 species) are utilised for all kinds of medical conditions (in both animals and humans) ranging from a simple cold to more complex ailments such as cancer and diabetes. There are 193 plant species utilised as food including staple food, vegetables, fruits, and beverages. Several species have functional uses such as thatching, clothing, textiles, building, cosmetics, crafts, fuel, musical instruments, and other cultural applications. 197 species are used for magic, mostly as a variety of charms, for example love charms, good luck charms, and protective charms.

Keywords: Food plants; Functional plants; Magic plants; Medicinal plants; Traditional uses

J.M.-A.S. Ouachinou, G.H. Dassou, R. Idohou, A.C. Adomou, H. Yédomonhan,

National inventory and usage of plant-based medicine to treat gastrointestinal disorders with cattle in Benin (West Africa),

South African Journal of Botany,

Volume 122,

2019,

Pages 432-446,

ISSN 0254-6299,

https://doi.org/10.1016/j.sajb.2019.03.037.

(https://www.sciencedirect.com/science/article/pii/S0254629918315850)

Abstract: Gastrointestinal disorders remained recurrent with livestock in Benin despite huge import of veterinary drugs at high costs. Nevertheless, the country abounds rich and varied anti-gastrointestinal flora which are hardly known, neglected and underutilized. The present study investigated the diversity of plants used to treat gastrointestinal disorders and documented the traditional knowledge associated with them. A total of 690 breeders and farmers were interviewed using open-ended and semi-structured interviews. Data were collected on the identity of the informants, plants and plant parts used, gastrointestinal disorders treated and usage types. Data were analyzed through calculation of relative frequency of citation (RFC), and use of descriptive statistics, multivariate analysis, bar charts and balloonplot. A total of 158 medicinal plant species belonging to 60 families and 130 genera were identified. The most represented were Leguminosae (18%) and Combretaceae (6.4%). Thirty-one plant families were mentioned to be highly utilized, among which the most important were Zygophyllaceae, Phytolaccaceae, Rubiaceae, Lamiaceae, Loranthaceae, Thymelaeaceae and Flacourtiaceae. The species were reported to treat seven gastrointestinal disorders. The most frequently cited were intern parasitosis (35%), diarrhea (29%) and constipation (17%). Leaves (40%) and stem barks (28%) are the plant parts mostly used to treat those disorders. The species with the highest value for RFC were: Khaya senegalensis, Anacardium occidentale, Cassia sieberiana, Pterocarpus erinaceus and Vitellaria paradoxa. Socioeconomic factors influencing ethnobotanical knowledge about these species were: age, profession and geographic location of the informants. Further analysis of chemical and pharmacological content of those species are necessary to ascertain the efficiency of their claimed properties and relieve farmers of these disorders.

Keywords: Benin; Ethnoveterinary plants; Gastrointestinal disorders; Cattle

Index,

Editor(s): James G. Speight,

Handbook of Industrial Hydrocarbon Processes (Second Edition),

Gulf Professional Publishing,

2020,

Pages 757-786,

ISBN 9780128099230,

https://doi.org/10.1016/B978-0-12-809923-0.20001-5.

(https://www.sciencedirect.com/science/article/pii/B9780128099230200015)

Suparmi Suparmi, Diana Widiastuti, Sebastiaan Wesseling, Ivonne M.C.M. Rietjens,

Natural occurrence of genotoxic and carcinogenic alkenylbenzenes in Indonesian jamu and evaluation of consumer risks,

Food and Chemical Toxicology,

Volume 118,

2018,

Pages 53-67,

ISSN 0278-6915,

https://doi.org/10.1016/j.fct.2018.04.059.

(https://www.sciencedirect.com/science/article/pii/S0278691518302825)

Abstract: The consumer risks of jamu, Indonesian traditional herbal medicines, was assessed focussing on the presence of alkenylbenzene containing botanical ingredients. Twenty-three out of 25 samples contained alkenylbenzenes at levels ranging from 3.8 to 440 μg/kg, with methyleugenol being the most frequently encountered alkenylbenzene. The estimated daily intake (EDI) resulting from jamu consumption was estimated to amount to 0.2–171 μg/kg bw/day for individual alkenylbenzenes, to 0.9–203 μg/kg bw/day when adding up all alkenylbenzenes detected, and to 0.9–551 μg/kg bw/day when expressed in methyleugenol equivalents using interim relative potency (REP) factors. The margin of exposure (MOE) values obtained were generally <10,000 indicating a priority for risk management when assuming daily consumption during a lifetime. Using Haber's rule it was estimated that two weeks consumption of these jamu only once would not raise a concern (MOE >10,000). However, when considering use for two weeks every year during a lifetime, 5 samples still raise a concern. It is concluded that the consumption of alkenylbenzene containing jamu can be of concern especially when consumed on a daily basis for longer periods of time on a regular basis.

Keywords: Alkenylbenzenes; Jamu; Margin of exposure (MOE); Risk assessment

R. Campos-Navarro, G.F. Scarpa,

The cultural-bound disease “empacho” in Argentina. A comprehensive botanico-historical and ethnopharmacological review,

Journal of Ethnopharmacology,

Volume 148, Issue 2,

2013,

Pages 349-360,

ISSN 0378-8741,

https://doi.org/10.1016/j.jep.2013.05.002.

(https://www.sciencedirect.com/science/article/pii/S0378874113003310)

Abstract: Ethnopharmacological relevance

Empacho is one of the most recognized cultural-bound syndromes in Argentina. It is a digestive disorder with many causes, being excessive food intake the most frequent. It is easily diagnosed in household medicine and there are different treatments applied for releasing the obstruction of the gastrointestinal tract. Therapeutics includes the use of medicinal plants and abdominal maneuvers, as well as rituals of magical and/or religious nature. The aim of this work is to analyze the compiled literature, considering documents from the XVIIIth century up to present, related to the employed plant species for the treatment of empacho.

Material and methods

The bibliographic and journal collections of several Argentinean and foreign libraries and bookstores were consulted, in addition to the comprehensive review of the specific information found online.

Results

Ninety (90) primary sources, spanning three hundred years (from 1710 to 2010) were found; most of them included ethnobotanical studies besides others of medical botany, pharmacobotanical and anthropological origin. A total of 152 plant species used to treat empacho were found in 360 total quotations, being Dysphania ambrosioides (L.) Mosyakin and Clemants; Alternanthera pungens Kunth; Ruta chalepensis L.; Clinopodium gilliesii (Benth.) Kuntze; Aloysia polystachya (Griseb.) Moldenke; Lippia turbinata Griseb., and Pluchea sagittalis (Lam.) Cabrera, the most frequently mentioned. The main therapeutic properties of the medicinal plants cited against empacho are stomachic, purgative, antispasmodic, bitter-tonic, carminative, and cholagogue-choleretic.

Conclusions

The variety of regions – spanning most of the country – from which the information comes, as well as the great variety of therapeutic strategies used, diversity of plant species and knowledge related to the treatment of empacho, is directly associated with the great significance that this disorder has within the system of medical-nosologic representations of the Argentinean popular medicine.

Keywords: Historical sources; Southern America's traditional medicine; Empacho; Gastrointestinal disease; Argentina

Nasir El Bassam, Preben Maegaard,

5 - Renewable Energy Resources and Technologies,

Editor(s): Nasir El Bassam, Preben Maegaard,

Integrated Renewable Energy for Rural Communities,

Elsevier,

2004,

Pages 71-191,

ISBN 9780444510143,

https://doi.org/10.1016/B978-044451014-3/50034-X.

(https://www.sciencedirect.com/science/article/pii/B978044451014350034X)

Abstract: Publisher Summary

This chapter discusses renewable sources of energy, such as biomass, solar energy, wind energy, hydropower, geothermal energy, marine energy, ocean thermal energy, tidal energy, wave energy, and hydrogen and the technologies involved in extracting these energies, as well as their environmental and economic impact. The world's biomass producing areas can be split into three distinct geographical regions: temperate regions, arid and semi-arid regions, and humid tropical regions. Energy plant species in these regions are listed. Ethanol, combustion, gasification, and pyrolysis are the important technologies used to extract energy from biomass. The use of windmills for yielding energy from wind is described. Photovoltaics (PV) are a reliable, renewable, environmentally safe, and cost-effective solar technology that converts sunlight directly into electricity. Similarly, turbines, water wheels, and pumps are used for generating hydropower. Geothermal heat pumps and drilling machines are used for accessing geothermal energy. The technologies for extracting marine energy involve horizontal axis turbines and vertical axis turbines. Ocean Thermal Energy Conversion (OTEC) is a technology, which converts ocean thermal energy into electricity—often while producing desalinated water. For wave energy conversion, there are three basic systems: channel systems that funnel the waves into reservoirs, float systems that drive hydraulic pumps, and oscillating water column systems that use the waves to compress air within a container. Systems generally used to produce hydrogen are electrochemical, photoelectrochemical, and photobiological. Fuel cells that convert chemical energy directly into electricity by combining hydrogen and oxygen in a controlled reaction are also discussed. Alternative transportation fuels, such as biodiesel, electricity, ethanol, hydrogen, methanol, natural gas, propane, p-series, and solar energy are briefly described. The chapter presents deployment of biogas in Denmark as a case study.

Species Index,

Editor(s): Alastair Fitter, Robert Hay,

Environmental Physiology of Plants (Third Edition),

Academic Press,

2002,

Pages 355-357,

ISBN 9780122577666,

https://doi.org/10.1016/B978-0-08-054981-1.50015-8.

(https://www.sciencedirect.com/science/article/pii/B9780080549811500158)

Andrea Pieroni, Cassandra L. Quave,

Traditional pharmacopoeias and medicines among Albanians and Italians in southern Italy: A comparison,

Journal of Ethnopharmacology,

Volume 101, Issues 1–3,

2005,

Pages 258-270,

ISSN 0378-8741,

https://doi.org/10.1016/j.jep.2005.04.028.

(https://www.sciencedirect.com/science/article/pii/S0378874105003090)

Abstract: A cross-cultural comparison of traditional household remedies in primary health care and ritual healing practices in two economically and socio-demographically similar communities in Lucania (inland southern Italy) was considered: Ginestra/Zhurë, inhabited by ethnic Albanians, who migrated to the area during the 15th century, and Castelmezzano, inhabited by autochthonous South-Italians. In Ginestra/Zhurë, the number of traditional natural remedies (mainly derived from local medicinal plants) was only half of that in the local folk pharmacopoeia quoted in Castelmezzano. However, ritual magic-healing practices still play a central role among the Albanians in Ginestra/Zhurë, while they do not in Castelmezzano. Reasons for this shift, as well as components that have affected cultural adaptation phenomena and transitions among the Albanians are discussed.

Keywords: Ethnopharmacy; Ethnobotany; Ethnomedicine; Albanians; Italy

Marie-Pierre Ruas, Jerôme Ros, Jean-Frédéric Terral, Sarah Ivorra, Hélène Andrianarinosy, Ahmed S. Ettahiri, Abdallah Fili, Jean-Pierre Van Staëvel,

History and archaeology of the emblematic argan tree in the medieval Anti-Atlas Mountains (Morocco),

Quaternary International,

Volume 404, Part A,

2016,

Pages 114-136,

ISSN 1040-6182,

https://doi.org/10.1016/j.quaint.2015.09.030.

(https://www.sciencedirect.com/science/article/pii/S1040618215008988)

Abstract: The argan tree [Argania spinosa (L.) Skeel.] is a spontaneous and xerophilous species endemic to south-western Morocco and the only representative species of the tropical Sapotaceae family in the country. It forms well-developed woodlands in the plain of Souss and open steppic vegetation on the semi-arid slopes of the Anti-Atlas Mountains. Currently, wild and managed argan trees are a staple firewood and timber resource, leaves and fruit are used for fodder and oil from the seeds for daily food. In the Anti-Atlas southeast of Taroudant, various tree growth forms can be observed in keeping with local management practices. We developed interdisciplinary research in this area by studying the bioarchaeological remains from the medieval site of Îgîlîz in conjunction with current farming practices in the neighbouring village of Tifigit. A plant inventory of 96 taxa was recorded from the archaeobotanical remains, including 13 wild and cultivated tree species and eight herbaceous crops (cereals, pulses, vegetables, condiments and fruit). The charred wood and seeds of Argania spinosa predominated in a wide range of contexts, indicating the major role of this species in the 10th–13th century economy. The ethnobotanical survey focused on the exploitation of argan trees and argan oil extraction techniques. In this paper, we discuss the past and present-day role of the argan tree in the agro-pastoral economy of the mountain hinterland.

Keywords: Archaeobotany; Argan forest; Ethnobotany; Middle ages; Morocco; Mountains

Dong-lin Li, Fu-wu Xing,

Ethnobotanical study on medicinal plants used by local Hoklos people on Hainan Island, China,

Journal of Ethnopharmacology,

Volume 194,

2016,

Pages 358-368,

ISSN 0378-8741,

https://doi.org/10.1016/j.jep.2016.07.050.

(https://www.sciencedirect.com/science/article/pii/S0378874116304743)

Abstract: Aims of the study

The aim of this study is to collect information on the use of medicinal plants by the local Hoklos people on Hainan Island, and compare medicinal traditions in the study area with Li medicines (LM) and traditional Chinese medicines (TCM).

Materials and methods

Ethnobotanical data were collected by 3 means: semi-structured interviews, personal conversation and guided field trips. There were 27 informants from 9 towns and 14 villages who were visited. Quantitative indices (Informant Consensus Factor – FIC, Use Value – UV, and Fidelity Level – FL) were calculated.

Results

In the present study, 264 species from 92 families and 233 genera were recorded, with Compositae (20 species), Leguminosae (19 species), Rubiaceae (12 species) and Gramineae (11 species) as predominate families. Leaves were the most frequently used parts in the preparation of local medicines. The most common preparation method was decoction (452 mentions). The plant with the highest values was Eclipta prostrata (0.46). The 6 plant species with the maximum FL (100%) were Atalantia buxifolia (Poir.) Oliv., Garcinia oblongifolia Champ. ex Benth., Hypericum japonicum Thunb. ex Murray, Imperata cylindrica (L.) Beauv., Microcos paniculata L., and Psidium guajava L. In addition, 120 investigated human ailments were grouped into 10 categories, within which symptoms and signs (184 mentions), diseases of the skin and subcutaneous tissue (139 mentions) and diseases of the digestive system (94 mentions) were the most mentioned in our investigation. The informant consensus about using medicinal plants ranged from 0.27 to 0.48, which showed a high level of agreement among the informants on symptoms and signs (0.48) and certain infectious and parasitic diseases (0.43). In comparison with TCM and LM, the results reflected a closer connection between local medicine and TCM.

Conclusion

The information reported by Hoklos people is of great value to ethnic medicinal culture. However, this precious medicinal knowledge is at risk of being lost due to rapid degradation of the environment. It is essential that more people engage in in-depth studies on local medicinal plants and relevant organizations address this serious problem before the damage is irreversible.

Keywords: Medicinal plants; Traditional knowledge; Hoklos people; Wenchang; Hainan Island

Jelena S. Matejić, Nikola Stefanović, Milan Ivković, Nemanja Živanović, Petar D. Marin, Ana M. Džamić,

Traditional uses of autochthonous medicinal and ritual plants and other remedies for health in Eastern and South-Eastern Serbia,

Journal of Ethnopharmacology,

Volume 261,

2020,

113186,

ISSN 0378-8741,

https://doi.org/10.1016/j.jep.2020.113186.

(https://www.sciencedirect.com/science/article/pii/S0378874120330683)

Abstract: Ethnopharmacological relevance

River and mountain regions in Eastern and South-Eastern Serbia are geographically interesting and, historically they represent an important resource of plants used as food, spices and as remedies for treating many diseases. Different cultures have lived in these regions for ages. They have used wild plants and the methods of their preparation and application, which has remained throughout the history and now is passed on from generation to generation. The aim of the study is a survey of herbal drug uses for the specific ailment categories and their comparison between the two research regions.

Methods

Semi-structured anonymous ethnobotanical interviews were conducted. The interviews took place in the River Timok region and Mountain Svrljig region as they make two of the most interesting centers of plant biodiversity. Volunteers in the Timok region were 64 median age and in the Svrljig region - 73 median age. People were interviewed about the local names of plants, the preparation process and about which disease the plants were used for.

Results and Discussion

161 Respondents from 10 Timok and 10 Svrljig municipality villages were interviewed and 2199 use-reports were recorded. The results of the ethnobotanical research showed 195 plant taxa from the Timok and Svrljig regions. In the Timok region, the recorded species were classified into 47 families and in the Svrljig region they were classified into 64 families. Out of 195 taxa used for medicinal purposes, only 52 species are also included in the European Pharmacopoeia 9.0. The most commonly used plants in Timok region were Hypericum spp., Matricaria chamomilla, Mentha x piperita, Urtica dioica, Juglans regia, while the residents of Svrljig region most frequently used Satureja montana, Sambucus nigra, Polygonum aviculare, Marrubium vulgare and Teucrium chamaedrys. Different statistical analyzes showed that Vlachs used more medical specimens per person than Serbs in the Timok region. The study demonstrated that female informants treated more body organs with medical species per person than male informants. There was a significant difference between the male and female respondents of the Svrljig region in terms of organ systems for which they used certain plant species.

Conclusion

The ethnopharmacological study showed a great importance of medicinal plants in the daily life of local communities. According to the analysis, it can be concluded that the village population of the Timok and Svrljig regions use medicinal plants to treat digestive tract problems rather than seeking professional medical attention in health facilities. The aerial part and rhizome of Elymus repens are used for digestive problems in both the Timok and Svrljig regions, and the use of this plant in for the treatment of digestive tract problems is not mentioned in the similar studies conducted in the Balkan region. Also, it can be observed that the population of the two different regions mainly use different herbal drugs to treat the same systems. The reason for that is the availability of certain plants that grow in the two different areas - river and mountain area.

Keywords: Ethnobotany; Traditional medicine; Eastern and south-eastern Serbia; River regions; Mountain regions

Fakchich Jamila, Elachouri Mostafa,

Ethnobotanical survey of medicinal plants used by people in Oriental Morocco to manage various ailments,

Journal of Ethnopharmacology,

Volume 154, Issue 1,

2014,

Pages 76-87,

ISSN 0378-8741,

https://doi.org/10.1016/j.jep.2014.03.016.

(https://www.sciencedirect.com/science/article/pii/S0378874114001937)

Abstract: Ethnopharmacological relevance

This document presents the uses of plants in traditional herbal medicines in Oriental Morocco. It also determines the homogeneity of informant knowledge in medicinal plants suitable for different ailment categories and the most preferred plant species used to treat each illness category in the study area.

Materials and methods

The ethnobotanical information was obtained from 3151 inhabitants who were 20 years and older in five different areas of Oriental Morocco region. The data were analyzed through informant consensus factor (ICF) and frequency of uses (FC).

Results

The results indicated that 65.7% of the participants interviewed used medicinal plants to treat 23 ailments. The inventory of medicinal plants is summarized in a synoptic table, which contains the scientific and vernacular names of the plant, the part of the plant and the preparation used and the therapeutic indication. Extensive investigations have brought to light 148 medicinal plants belonging to 60 families; of these, 108 are used for the disorders of the digestive system, 74 for diabetes, 73 for dermatological problems, 66 for allergy, 66 for cardiovascular disorders and 63 for respiratory problems. In this region, the most frequently used plants including Origanum compactum Benth., Trigonella foenum graecum L., Lavandula dentata L., Mentha pulegium L., Nigella sativa L., Rosmarinus officinalis L., Lippia citriodora L., Tetraclinis articulata Benth., and Atemisia herba-alba Asso. Lamiaceae and Asteraceae are the dominant locally used families. Most medicines were prepared in the form of powder and used orally. Leaves were the most frequently used plant part. Gastro-intestinal ailments have high ICF (0.92) whereas pathologies of the circulatory and ophthalmological uses have low ICF (0.22 and 0.24, respectively).

Conclusion

Oriental Morocco boasts an extensive phytotherapy knowledge base and ICF values indicated that there was high agreement in the use of plants in gastro-intestinal ailment category among the users. The frequency use value (FC) indicated that these plants are the most preferred species used in study areas. These preferred plant species could be prioritized for conservation and subjected to further studies related to chemical screening for their authenticity.

Keywords: Medicinal plant; Oriental Morocco; Management; Ailment; Informant consensus factor; Frequency

M.N.V. Prasad, Woranan Nakbanpote, Abin Sebastian, Natthawoot Panitlertumpai, Chaiwat Phadermrod,

Chapter 23 - Phytomanagement of Padaeng Zinc Mine Waste, Mae Sot District, Tak Province, Thailand,

Editor(s): Khalid Rehman Hakeem, Muhammad Sabir, Münir Öztürk, Ahmet Ruhi Mermut,

Soil Remediation and Plants,

Academic Press,

2015,

Pages 661-687,

ISBN 9780127999371,

https://doi.org/10.1016/B978-0-12-799937-1.00023-1.

(https://www.sciencedirect.com/science/article/pii/B9780127999371000231)

Abstract: Economic development of a nation is linked to richness of natural resources. Minerals are one of the important natural resources. Thailand is rich in minerals such as zinc (Zn), lead (Pb), tin (Sn) and gold (Au). Thailand’s largest zinc deposits are located in Phatat Phadaeng sub-district, Mae Sot, Tak. Mining operations generate huge amounts of waste which is detrimental to the quality of the local environment. Thus the waste generated by mine operations is often piled up in the vicinity of the mining site. Due to strong weather conditions, the residual metals from the piled up metallic waste get eroded and transported to the local ecosystem. Decontamination of contaminated ecosystems is complex and cost-prohibitive and thus several undesirable health effects are witnessed often. Phytomanagement deals with use of a wide variety of phytodiversity to stabilize the mine waste and thus reduce the bioavailability of toxic doses of metals. Phytomanagement also deals with control of leaching of mine waste to surrounding ecosystems and feasible options for production of safe food are dealt with in this manuscript. Phytomanagement is emerging as a potential field of phytotechnology for restoration of mine waste.

Keywords: Human health; Metal-tolerant plants; Metalliferous substrates; Mine water; Minimization of heavy metals in crops; Phytostabilization; Rehabilitation

RAI BAHADUR JAISING P. MODI,

CHAPTER XXVI - IRRITANT POISONS—(Contd.),

Editor(s): RAI BAHADUR JAISING P. MODI,

A Textbook of Medical Jurisprudence and Toxicology (Sixth Edition),

Butterworth-Heinemann,

2013,

Pages 627-657,

ISBN 9781483168241,

https://doi.org/10.1016/B978-1-4831-6824-1.50030-7.

(https://www.sciencedirect.com/science/article/pii/B9781483168241500307)

Jörg Romeis, Dirk Babendreier, Felix L. Wäckers, Thomas G. Shanower,

Habitat and plant specificity of Trichogramma egg parasitoids—underlying mechanisms and implications,

Basic and Applied Ecology,

Volume 6, Issue 3,

2005,

Pages 215-236,

ISSN 1439-1791,

https://doi.org/10.1016/j.baae.2004.10.004.

(https://www.sciencedirect.com/science/article/pii/S1439179104000891)

Abstract: Summary

Egg parasitoids of the genus Trichogramma are among the most important and best-studied natural enemies worldwide. Parasitism levels by Trichogramma vary greatly among different habitats, plants or plant structures on which the host eggs are located. Here we summarise the published evidence on mechanisms that may underlie the observed variation in parasitism rates. These mechanisms include plant spacing, plant structure, plant surface structure and chemistry, plant volatiles and plant colour. In addition, plants can affect parasitoid behaviour and activity by providing carbohydrate food sources such as nectar to the adult wasps, and by affecting the nutritional quality of the host eggs for progeny development. Knowledge of plant and habitat factors that affect Trichogramma spp. efficacy has important implications for biological control, and for assessing the risks that mass-released Trichogramma spp. may pose to non-target insects.

Zusammenfassung

Eiparasitoide der Gattung Trichogramma gehören weltweit zu den wichtigsten und am besten bekannten Nützlingen. Parasitierungsraten von Trichogramma variieren deutlich zwischen verschiedenen Habitaten, Wirtspflanzen bzw. Teilen einer Pflanze, auf denen sich die Wirtseier befinden. In der vorliegenden Arbeit fassen wir zusammen, was hinsichtlich der Mechanismen bekannt ist, die für die beobachteten Unterschiede in der Parasitierungsleistung verantwortlich sein können. Diese Mechanismen umfassen Faktoren wie den Abstand zwischen den Pflanzen, die Pflanzenstruktur, die strukturelle und chemische Beschaffenheit der Pflanzenoberfläche, pflanzliche Duftstoffe sowie die Farbe der Pflanze. Hinzu kommt, dass Pflanzen das Verhalten bzw. die Aktivität der adulten Parasitoide beeinflussen können indem sie zuckerhaltige Nahrung z.B. in Form von Nektar zur Verfügung stellen. Ausserdem haben die Pflanzen einen Einfluss auf die chemische Zusammensetzung der Wirtseier, was sich wiederum auf die Eiparasitoide auswirken kann. Ein gutes Verständnis der Habitat- und Pflanzen-Faktoren, welche die Eiparasitoide beeinflussen, ist wichtig um den Einsatz von Trichogramma spp. in der biologischen Schädlingsbekämpfung zu fördern und auch um die möglichen Umweltauswirkungen von im Pflanzenschutz eingesetzten Trichogramma spp. zu erfassen.

Keywords: Biological control; Conservation biological control; Food ecology; Multi-trophic interactions; Non-target effects; Plant volatiles; Trichomes

T.N.M. Kankanamalage, R.M. Dharmadasa, D.C. Abeysinghe, R.G.S. Wijesekara,

A survey on medicinal materials used in traditional systems of medicine in Sri Lanka,

Journal of Ethnopharmacology,

Volume 155, Issue 1,

2014,

Pages 679-691,

ISSN 0378-8741,

https://doi.org/10.1016/j.jep.2014.06.016.

(https://www.sciencedirect.com/science/article/pii/S0378874114004644)

Abstract: Ethnopharmacological relevance

Sri Lanka has rich traditional systems of medicine, which cater to 60–70% of the rural population׳s primary health care needs. However, development of existing systems has been hindered by the unavailability of up-to-date information on medicinal materials and other related issues. For streamlining purposes, we investigated the present-day scenario of country׳s medicinal plant industry by gathering up-to-date information on the types of raw materials required, their aggregate quantities, heavily used and rare materials, family wise distribution, challenges faced by stakeholders as well as other pertinent issues.

Materials and methods

The present survey covered the selected government Ayurveda hospitals, traditional and Ayurveda practitioners, large and small-scale herbal drug and cosmetic manufactures, importers, collectors and Ayurveda commissioners throughout the country. A systematic questionnaire was distributed and face-to-face interviews were conducted. Collected data were tabulated and analyzed.

Results

A diverse range of medicinal materials, including 290 species (64.73%) from dried plants, 59 (13.17%) from fresh plants, 69 (15.40%) from minerals, 18 (4.02%) from animal sources and 12 (2.68%) from other sources were recorded. A total of 302 plant species belonging to 95 families, dominated by Leguminosae family, was listed. Out of these, 46 species belonging to 35 families were used intensively. A large portion of herbal materials was of completely local origin (71.13%) while 26% were imported and the rest (2.87%) can be obtained by both routes. Leaves were the most highly used part of the plant (22.2%). High price, poor quality, insufficient or totally absence of continuous supply and adulteration were the main constraints faced by the stakeholders. The unavailability of systematic cultivation and processing protocols, incorrect identification, and lack of proper quality control methodologies were identified as major challenges of the industry.

Conclusion

The present study revealed a currently bleak scenario of the medicinal material industry in Sri Lanka. The results clearly demonstrated the need to implement a national strategy to address the major challenges faced by different stakeholders. Information generated through this study could be effectively incorporated for the formulation of a sustainable development strategy for this industry.

Keywords: Survey; Medicinal materials; Sri Lanka; Traditional medicine

South African Association of Botanists-Annual Meeting 2006,

South African Journal of Botany,

Volume 72, Issue 2,

2006,

Pages 313-347,

ISSN 0254-6299,

https://doi.org/10.1016/j.sajb.2005.01.001.

(https://www.sciencedirect.com/science/article/pii/S0254629906000020)

Subject Index,

Editor(s): Atta-ur-Rahman,

Studies in Natural Products Chemistry,

Elsevier,

Volume 22, Part C,

2000,

Pages 751-781,

ISSN 1572-5995,

ISBN 9780444505880,

https://doi.org/10.1016/S1572-5995(00)80039-3.

(https://www.sciencedirect.com/science/article/pii/S1572599500800393)

Subject Index,

Editor(s): Atta-ur-Rahman,

Studies in Natural Products Chemistry,

Elsevier,

Volume 29, Part J,

2003,

Pages 807-902,

ISSN 1572-5995,

ISBN 9780444515100,

https://doi.org/10.1016/S1572-5995(03)80019-4.

(https://www.sciencedirect.com/science/article/pii/S1572599503800194)

Mojtaba Salouti, Fatemeh Khadivi Derakhshan,

Chapter 3 - Phytosynthesis of Nanoscale Materials,

Editor(s): Mansour Ghorbanpour, Shabir Hussain Wani,

Advances in Phytonanotechnology,

Academic Press,

2019,

Pages 45-121,

ISBN 9780128153222,

https://doi.org/10.1016/B978-0-12-815322-2.00003-1.

(https://www.sciencedirect.com/science/article/pii/B9780128153222000031)

Abstract: Nanotechnology deals with the production and usage of materials with nanoscale dimensions. The nanosized particles make an imprint in our daily lives and it has great importance in numerous fields of biotechnology, such as the food industry, medical, and industrial fields. Conventionally, metal nanoparticles are synthesized by chemical methods, which later on become accountable for various biological risks due to their general toxicity, engendering serious concern to develop environment-friendly processes. Green synthesis is the most fascinating, comparatively simple, and attractive alternative to chemical synthesis as it offers more advantages. The use of plant extracts for synthesis of NPs is potentially advantageous over microorganisms due to the ease of scaling up the biohazards and elaborates process of maintaining cell cultures. Nontoxic and ecofriendly secondary metabolites from plants are also used as reducing and capping agents. In this chapter, we provide comprehensive information about the current status of plant-mediated synthesis of metal nanoparticles, factors responsible for reduction of metal nanoparticle, and their applications.

Keywords: Metal nanoparticles; Plant-mediated synthesis; Phytosynthesis; Metabolites; Characterization; Optimization; Application

Mirza Hasanuzzaman, Kamrun Nahar, Masayuki Fujita,

Chapter 16 - Silicon and Selenium: Two Vital Trace Elements that Confer Abiotic Stress Tolerance to Plants,

Editor(s): Parvaiz Ahmad, Saiema Rasool,

Emerging Technologies and Management of Crop Stress Tolerance,

Academic Press,

2014,

Pages 377-422,

ISBN 9780128008768,

https://doi.org/10.1016/B978-0-12-800876-8.00016-3.

(https://www.sciencedirect.com/science/article/pii/B9780128008768000163)

Abstract: Plants are sessile organisms and therefore must constantly adapt their growth and architecture to an ever-changing environment. The damages in growth and development of economic plants caused due to abiotic stresses like salinity, drought, extreme temperature, flooding, toxic metals, ozone, and UV radiation are quite alarming due to shrinking agricultural land area, recurrently expanding population, and rapid climate change throughout the world. To cope with adverse growing conditions, plant scientists are searching for ways to make plants adaptive. They are trying to understand the effect of environmental stresses on plants and to modify plants’ outer growing conditions and their internal cellular environment by applying different exogenous protectants. Silicon (Si) and selenium (Se) are widely studied trace elements and their roles in plant growth and physiology are well documented. Recently, these trace elements have been found to be protective under abiotic stress conditions. Silicon is the second most abundant element after oxygen in soils and its presence in the form of silicic acid allows its uptake by plants, so by nature plants have a great scope to uptake Si in their tissues. However, those plants not supplied with sufficient natural sources of Si may benefit from its exogenous application. Selenium, an essential element for animals and humans, has also been found to be beneficial to plants. Like Si, Se also plays a protective role in conferring tolerance to certain abiotic stresses when applied at lower concentrations, while higher concentrations show phytotoxicity. However, plant species differ strongly in Se uptake and accumulation as well as their tolerance capacity. Both Si and Se were reported to play roles in conferring oxidative stress tolerance by enhancement of the antioxidant defense system in plants. Although much research has been published on the effect of Si and Se on plants under abiotic stress, clear-cut underlying physiological mechanisms of the mode by which Si could protect plants from stressful conditions are elusive. In this chapter, we attempt to summarize the uptake and accumulation pattern of Si and Se in plants. Later, we discuss the recent reports regarding the role of Si and Se in conferring abiotic stress tolerance to plants.

Keywords: abiotic stress; antioxidant defense; climate change; oxidative stress; plant adaptation; plant stress responses; selenium; silicon; trace elements

A Pieroni,

Medicinal plants and food medicines in the folk traditions of the upper Lucca Province, Italy,

Journal of Ethnopharmacology,

Volume 70, Issue 3,

2000,

Pages 235-273,

ISSN 0378-8741,

https://doi.org/10.1016/S0378-8741(99)00207-X.

(https://www.sciencedirect.com/science/article/pii/S037887419900207X)

Abstract: An ethnopharmacobotanical survey of the medicinal plants and food medicines of the northern part of Lucca Province, north-west Tuscany, central Italy, was carried out. The geographical isolation of this area has permitted the survival of a rich folk phytotherapy involving medicinal herbs and also vegetable resources used by locals as food medicine. Among these are the uncommon use of Ballota nigra leaves as a trophic protective; the use of Lilium candidum bulbs as an antiviral to treat shingles (Herpes zoster); Parmelia sp. as a cholagogue; Crocus napolitanus flowers as antiseptic; Prunus laurocerasus drupes as a hypotensive; and the consumption of chestnut flour polenta cooked with new wine as bechic. Many wild gathered greens are eaten raw in salads, or in boiled mixtures, as ‘blood cleansing’ and ‘intestine cleansing’ agents. Of particular interest is the persistence of the archaic use of Bryonia dioica root against sciatica, and the use of ritual plant therapeuticals as good omens, or against the ‘evil eye.’ Over 120 species represent the heritage of the local folk pharmacopoeia in upper Garfagnana. Anthropological and ethnopharmacological considerations of the collected data are also discussed.

Keywords: Medicinal plants; Ethnobotany; Food medicine; Tuscany; Italy

Angkhana Inta, Paritat Trisonthi, Chusie Trisonthi,

Analysis of traditional knowledge in medicinal plants used by Yuan in Thailand,

Journal of Ethnopharmacology,

Volume 149, Issue 1,

2013,

Pages 344-351,

ISSN 0378-8741,

https://doi.org/10.1016/j.jep.2013.06.047.

(https://www.sciencedirect.com/science/article/pii/S0378874113004807)

Abstract: Ethnopharmacological relevance

We studied traditional knowledge of medicinal plants use of the Yuan in northern Thailand, documenting and analysing traditional medical practices and its trend in the younger generations.

Aim of the study

To providing useful information for appropriate and sustainable management under the urbanization and other developments and use of natural resources in their communities. In addition, traditional medicinal plant used, and knowledge that leads to discovery of new medicines can be promoted.

Materials and methods

Traditional medicinal plant knowledge of the Yuan in Lamphun province was studied from October 2009 through September 2011 in order to determine the important medicinal plant species and dominant use-categories in 5 villages. In each village, questionnaire interviews about medicinal plants uses were applied to 30 informants (5 informants per each of six stratified age groups). The relative importance of plant species was captured by calculation of use value (UV). Likewise, the dominant use-categories were determined by calculation of the informant agreement ratio (IAR). Correlations between informants’ age and number of medicinal plants known by them were determined with the coefficient of determination (R2).

Results

A total of 93 medicinal plant species in 82 genera and 49 families were recorded in the five villages. The most important species of medicinal plants were Aloe vera (L.) Burm.f., Andrographis paniculata Ness, Chromolaena odorata (L.) R.M.King and H.Rob., Jatropha podagrica Hook., and Thunbergia laurifolia Lindl. which had UVs of 1.02, 1.01, 0.75, 0.71, and 0.65, respectively. Likewise, the most dominant use-categories were injuries, which accounted for 0.91 of the IAR. The age of informants and medicinal plants reported by each of them were positively correlated (R2=0.96, p<0.01).

Conclusions

Most of the Yuan's traditional medicinal plant knowledge is used for treating basic ailments. However we should be concerned that there is an imminent danger that it will be lost in the near future because their lifestyle was changing.

Keywords: Ethnobotany; Traditional medicinal plants; Northern Thai; Use value (UV); Informant agreement ratio (IAR)

Peter Giovannini, Melanie-Jayne R. Howes, Sarah E. Edwards,

Medicinal plants used in the traditional management of diabetes and its sequelae in Central America: A review,

Journal of Ethnopharmacology,

Volume 184,

2016,

Pages 58-71,

ISSN 0378-8741,

https://doi.org/10.1016/j.jep.2016.02.034.

(https://www.sciencedirect.com/science/article/pii/S0378874116300770)

Abstract: Ethnopharmacological relevance

Globally 387 million people currently have diabetes and it is projected that this condition will be the 7th leading cause of death worldwide by 2030. As of 2012, its total prevalence in Central America (8.5%) was greater than the prevalence in most Latin American countries and the population of this region widely use herbal medicine. The aim of this study is to review the medicinal plants used to treat diabetes and its sequelae in seven Central American countries: Belize, Costa Rica, El Salvador, Guatemala, Honduras, Nicaragua and Panama.

Materials and methods

We conducted a literature review and extracted from primary sources the plant use reports in traditional remedies that matched one of the following disease categories: diabetes mellitus, kidney disease, urinary problems, skin diseases and infections, cardiovascular disease, sexual dysfunctions, visual loss, and nerve damage. Use reports were entered in a database and data were analysed in terms of the highest number of use reports for diabetes management and for the different sequelae. We also examined the scientific evidence that might support the local uses of the most reported species.

Results

Out of 535 identified species used to manage diabetes and its sequelae, 104 species are used to manage diabetes and we found in vitro and in vivo preclinical experimental evidence of hypoglycaemic effect for 16 of the 20 species reported by at least two sources. However, only seven of these species are reported in more than 3 studies: Momordica charantia L., Neurolaena lobata (L.) R. Br. ex Cass., Tecoma stans (L.) Juss. ex Kunth, Persea americana Mill., Psidium guajava L., Anacardium occidentale L. and Hamelia patens Jacq. Several of the species that are used to manage diabetes in Central America are also used to treat conditions that may arise as its consequence such as kidney disease, urinary problems and skin conditions.

Conclusion

This review provides an overview of the medicinal plants used to manage diabetes and its sequelae in Central America and of the current scientific knowledge that might explain their traditional use. In Central America a large number of medicinal plants are used to treat this condition and its sequelae, although relatively few species are widely used across the region. For the species used to manage diabetes, there is variation in the availability and quality of pharmacological, chemical and clinical studies to explain traditional use.

Keywords: Diabetes; Central America; Medicinal plants; Ecosystem services; Traditional medicine; Hypoglycaemic

Bruno Menale, Olga De Castro, Ciro Cascone, Rosa Muoio,

Ethnobotanical investigation on medicinal plants in the Vesuvio National Park (Campania, Southern Italy),

Journal of Ethnopharmacology,

Volume 192,

2016,

Pages 320-349,

ISSN 0378-8741,

https://doi.org/10.1016/j.jep.2016.07.049.

(https://www.sciencedirect.com/science/article/pii/S0378874116304731)

Abstract: Ethnopharmacological relevance

This paper illustrates the results of an ethnobotanical study carried out in the Vesuvio National Park (VNP) (Campania, Southern Italy). It describes the medicinal uses of the plants in an ancient area rich in ethnobiodiversity investigated for the first time.

Aim for the study

The main aim of the study was to understand at what extent current knowledge on medicinal plant uses is still alive in VNP.

Materials and methods

The informations were collected using semi-structured and unstructured interviews performed on 136 persons living in the investigated area from March to November 2014 and from April to October 2015. The age of the informants ranged from 47 to 85 years old; more than half of the informants aged between 61 and 70. Local plant uses were listed and analyzed in a table and compared with uses in other localities in Italy and in other regions of the Mediterranean basin.

Results

In VNP were recorded a total number of 132 plant species, belonging to 110 genera and 51 families mentioned for medicinal purposes. Among the recorded 132 plant species, 70 are spontaneous or subspontaneous and 62 are cultivated above all in the kitchen gardens or in the apartments, as food or as ornamental. Herbs represent the majority, followed by trees and shrubs or subshrubs. The investigated plants were used to cure 116 different human health diseases and 4 veterinary problems. The majority of plants are used in the treatment of gastrointestinal, skin and respiratory problems.

Conclusion

The number of medicinal plants reported in this paper reflects a well-preserved traditional popular knowledge (TPK) of the elderly people living in the rural areas and in the small villages of VNP. The conservation of TPK is owed to the persistence of an oral tradition that safeguard the use of plants as herbal medicine. We realized that while the use of some wild plants is decreasing, people continue to gather some cultivated and invasive plants for preparing remedies. Researches like this are necessary to protect ancient memories, to promote the transfer of information to the younger generations, to preserve ethno-biodiversity and to provide a starting point fur further biochemical investigations on medicinal entities.

Keywords: Ethnobotanical study; Ethno-biodiversity; Plant traditional uses; Medicinal plants; Vesuvio National Park

Mahmoud Nasrollahzadeh, Mohaddeseh Sajjadi, S. Mohammad Sajadi, Zahra Issaabadi,

Chapter 5 - Green Nanotechnology,

Editor(s): Mahmoud Nasrollahzadeh, S. Mohammad Sajadi, Mohaddeseh Sajjadi, Zahra Issaabadi, Monireh Atarod,

Interface Science and Technology,

Elsevier,

Volume 28,

2019,

Pages 145-198,

ISSN 1573-4285,

ISBN 9780128135860,

https://doi.org/10.1016/B978-0-12-813586-0.00005-5.

(https://www.sciencedirect.com/science/article/pii/B9780128135860000055)

Abstract: While various nanoparticles are being increasingly utilized in many sectors of the economy, there is growing interest in the environmental and biological safety linked to their preparation. Green nanotechnology provides tools for the transformation of biological systems to green approaches to nanomaterial synthesis, while preventing any associated toxicity. Due to the large number of toxic chemicals and extreme environments employed in the physicochemical production of these nanoparticles, green methods employ the use of biological sources. Through integrating the principles of green chemistry and engineering, green nanotechnology can produce safe and eco-friendly metal nanoparticles that do not use toxic chemicals in their synthesis. This chapter is a comprehensive study of the types of phytochemicals used in the synthesis of nanoparticles including proteins, polysaccharides, vitamins, glycosides, essential oils, and phenolic-based chemicals. It also provides the preparation methods used for different biological sources. Generally, the main role of natural plant biomolecules is involved with the bioreduction of metal salts during nanoparticle preparation. Furthermore, this chapter provides important examples of the effective use of plant extracts to obtain biosynthesized nanoparticles.

Keywords: Green nanotechnology; Phytochemical; Plant-derived nanostructures; Polysaccharide; Essential oils

R. Dutt, V. Garg, A.K. Madan,

Chapter 5 - Natural Chemotherapeutic Agents for Cancer,

Editor(s): Alejandro Speck-Planche,

Multi-Scale Approaches in Drug Discovery,

Elsevier,

2017,

Pages 99-126,

ISBN 9780081011294,

https://doi.org/10.1016/B978-0-08-101129-4.00005-9.

(https://www.sciencedirect.com/science/article/pii/B9780081011294000059)

Abstract: Natural products have been the source of numerous active ingredients of the present day medicines. The therapeutic use of plants against critical human illnesses can be traced back to early civilizations of the world and represents the most significant direct antecedent to modern era of drug discovery process. With the introduction of various computational techniques, the interest toward bioactive agents from natural sources into the cancer armamentarium has changed the anticancer drug discovery process. Numerous plants have the potential of anticancer activity but are still to be investigated. Diverse types of anticancer bioactive compounds have been isolated from plant sources that are currently in various phases of clinical or preclinical trials or undergoing further investigation. Various anticancer agents from natural sources reported/studied till date for anticancer activity have been briefly reviewed in the present article. An immediate need for screening plants for anticancer activity has been emphasized so as to accelerate development of effective but safe anticancer drugs.

Keywords: Anticancer drugs; Cancer; Carcinogens; Chemotherapeutic agents; Natural products

Kapitel 16 - Anhang,

Editor(s): Siegfried Bäumler,

Heilpflanzenpraxis Heute,

Urban & Fischer,

2006,

Pages 875-957,

ISBN 9783437572708,

https://doi.org/10.1016/B978-343757270-8.50022-6.

(https://www.sciencedirect.com/science/article/pii/B9783437572708500226)

Cumulative biological source index volumes 1–30,

Editor(s): Atta-ur- Rahman,

Studies in Natural Products Chemistry,

Elsevier,

Volume 31,

2005,

Pages 1225-1362,

ISSN 1572-5995,

ISBN 9780444518781,

https://doi.org/10.1016/S1572-5995(05)80102-4.

(https://www.sciencedirect.com/science/article/pii/S1572599505801024)

Abstract: Publisher Summary

This chapter lists the important subjects on biological source that are discussed in the publication, such as Abies grandis Acanthodris nanaimoensis, Baccharis crispa, acanthosterol sulfates, Agelas dispar, Plasmodium falciparum, Podophyllum hexandrum, Quillaja saponaria, and Rauwolfia serpentina. The terms are mentioned along with the page numbers in which they are discussed in the publication.

Bin CHEN, Yan-li TIAN, Yu-qiang ZHAO, Yuan-jie WANG, Jia-cheng CHUAN, Xiang LI, Bai-shi HU,

Genomic characteristics of Dickeya fangzhongdai isolates from pear and the function of type IV pili in the chromosome,

Journal of Integrative Agriculture,

Volume 19, Issue 4,

2020,

Pages 906-920,

ISSN 2095-3119,

https://doi.org/10.1016/S2095-3119(19)62883-2.

(https://www.sciencedirect.com/science/article/pii/S2095311919628832)

Abstract: Dickeya fangzhongdai, the causal agent of bleeding canker of pear, is a new member of the Dickeya genus and the only one that infects woody plants. Recent studies have reclassified several Dickeya isolates as D. fangzhongdai, which were isolated from various environments, including water, Phalaenopsis sp. and Aglaonema sp. To provide genomic characterization of D. fangzhongdai isolates from pear, the genomes of D. fangzhongdai strain JS5 (=China General Microbiological Culture Collection Center, CGMCC 1.15464T=DSM 101947T), along with two other isolates, LN1 and QZH3, were sequenced and compared to those of other Dickeya spp. Homology greater than 99% was observed among three D. fangzhongdai strains. Plasmid, type IV secretion system (T4SS) and type IV pili (TFPs) were found in genomes of D. fangzhongdai isolates. Comparative analysis of the type III secretion systems (T3SS), type III secretion effectors (T3SE), plant cell wall degradation enzymes (PCWDE) and membrane transport proteins of Dickeya spp. showed some differences which might reflect the variations of virulence, phylogenetic and phenotypic characteristics of Dickeya spp. In addition, deletion mutant of TFP in D. fangzhongdai JS5 showed no twitching motility and reduced virulence and biofilm formation. The fingdings of the distinctive plasmid, T4SS and TFPs, as well as the differences of T3SE, PCWDE and membrane transport proteins make D. fangzhongdai isolates unique. These results also suggested that acquisition of virulence genes by horizontal gene transfer might play some role in the genetic variation of D. fangzhongdai.

Keywords: Dickeya fangzhongdai; comparative genomics; virulence; type IV pili

Lilianna Głąb, Józef Sowiński, Raven Bough, Franck E. Dayan,

Chapter Two - Allelopathic Potential of Sorghum (Sorghum bicolor (L.) Moench) in Weed Control: A Comprehensive Review,

Editor(s): Donald L. Sparks,

Advances in Agronomy,

Academic Press,

Volume 145,

2017,

Pages 43-95,

ISSN 0065-2113,

ISBN 9780128124178,

https://doi.org/10.1016/bs.agron.2017.05.001.

(https://www.sciencedirect.com/science/article/pii/S0065211317300342)

Abstract: Weeds constitute the largest biotic threat affecting the yield of cultivated plants. While conventional agriculture relies principally on chemicals for weed control, alternative biological methods may be important tools to reduce weed pressure in agroecosystems. Furthermore, as the problem of excessive residue of plant protection agents in agroecosystems and the growing number of herbicide resistant weed biotypes continue to increase, new solutions that have smaller impacts on the environment are becoming increasingly desirable. One promising such method is the use of crops that exert a negative phytotoxic influence on weeds. This natural phenomenon describing the ability of certain plant species to produce compounds that affect the growth of other plants in their surroundings is called allelopathy. Managing weed infestations in cultivated fields by planting allelopathic crops is a sustainable, economic, and environmentally friendly approach that has been strongly articulated in the international arena. Among cultivated crops, sorghum (Sorghum bicolor (L.) Moench) has been intensively studied because of demonstrated allelopathic potential. This report provides a comprehensive literature review of the applications of sorghum allelopathy in agriculture. A critical analysis of the allelopathic properties of sorghum identified the following areas contributing to its ability to reduce weed infestation in agroecosystems:1.a large number of compounds produced by sorghum have allelopathic properties,2.allelopathic compounds can be applied in the form of mixed plant extracts or in combination with herbicides,3.sorghum extracts have a broad spectrum of activity,4.sorghum may be used to produce bioherbicides.

Keywords: Sorghum; Allelopathy; Sorgoleone; Sorgaab; Weed Control; Natural herbicide; Sustainable agriculture; Cover crop; Mulching; Organic agriculture

Kornkanok Tangjitman, Chalobol Wongsawad, Piyawan Winijchaiyanan, Treetip Sukkho, Kaweesin Kamwong, Wittaya Pongamornkul, Chusie Trisonthi,

Traditional knowledge on medicinal plant of the Karen in northern Thailand: A comparative study,

Journal of Ethnopharmacology,

Volume 150, Issue 1,

2013,

Pages 232-243,

ISSN 0378-8741,

https://doi.org/10.1016/j.jep.2013.08.037.

(https://www.sciencedirect.com/science/article/pii/S0378874113005916)

Abstract: Ethnopharmacological relevance

We studied traditional medicinal plant knowledge among the Karen in northern Thailand.

Aim of the study

To compare traditional medicinal knowledge in 14 Karen villages in northern Thailand and determine culturally important medicinal plant species in each Karen village.

Materials and methods

We interviewed 14 key informants and 438 non-specialist informants about their traditional knowledge of medicinal plants. We tested normality of the data and correlations with distance to the nearest city using Kolmogorov–Smirnov tests and Spearman's rank correlation coefficient. Cluster analysis and cultural importance index (CI) were calculated for the similarity of medicinal plant used and culturally importance medicinal plant species among Karen villages respectively.

Results

In total 379 medicinal plant species were used. Number of medicinal plants used positively correlate with distance to the nearest city. Relatively low similarities of medicinal plant species and different CI values for species among the different areas were found.

Conclusions

Traditional medicinal plants still play an important role in medicinal practice of the Karen. Local environments, availability of medicinal plant and distance between Karen villages and the nearest city affect the amount of traditional medicinal knowledge in each Karen village. The medicinal plants in this study with high CI values might give some useful leads for further biomedical research.

Keywords: Ethnobotany; Thai hill tribe; Cultural importance; Knowledge similarity; Distance to the nearest city; Chiang Mai

C.-F. Chau, S.-H. Wu,

The development of regulations of Chinese herbal medicines for both medicinal and food uses,

Trends in Food Science & Technology,

Volume 17, Issue 6,

2006,

Pages 313-323,

ISSN 0924-2244,

https://doi.org/10.1016/j.tifs.2005.12.005.

(https://www.sciencedirect.com/science/article/pii/S0924224405003560)

Abstract: Some Chinese herbal medicines (CHM) may be consumed for both the medicinal and food purposes in some traditional food dishes and herbal products, even though most of them are mainly considered as medicinal materials. The line between the medicinal and food characteristics of these herbal medicines is oftentimes blurred due to the dual uses. As natural herbal materials are not always safe, the regulation of CHM for food uses at the national level is for the protection of public health, as well as protecting consumers from potentially harmful herbal ingredients. This study compares and summarizes a considerable number of CHM that are allowed for food uses by the governmental agencies of mainland China and Taiwan. This article also discusses the development and provides an understanding of the regulations of CHM for both medicinal and food uses. The wealth of information may provide references for the development of food regulations on herbal products around the world.

Riccardo Di Novella, Nicola Di Novella, Laura De Martino, Emilia Mancini, Vincenzo De Feo,

Traditional plant use in the National Park of Cilento and Vallo di Diano, Campania, Southern, Italy,

Journal of Ethnopharmacology,

Volume 145, Issue 1,

2013,

Pages 328-342,

ISSN 0378-8741,

https://doi.org/10.1016/j.jep.2012.10.065.

(https://www.sciencedirect.com/science/article/pii/S0378874112007726)

Abstract: Aim of study

This paper reports an ethobotanical survey of the traditional uses of medicinal and useful plants in an area of the National Park of Cilento and Vallo di Diano, Campania, Southern Italy.

Materials and methods

This study conducted between 2009 and 2011, gathered information on the medicinal plants traditionally used in Southern Italy (Campania Region). In all, we interviewed 70 key informants, whose age ranged between 50 and 85 years. This people belonged to families which had strong links with traditional activities of the area.

Results

The research resulted to the identification of 192 plants belonging to 64 families. Among the species reported, 86 are used in human medicine, 15 in veterinary medicine, 69 as human foods, 18 as animal feed, 61 for domestic and 8 for agricultural uses.

Conclusion

A survey of the available literature on Southern Italy ethnobotany reveals that some species have been never reported and about 10% of cited uses are new. Data obtained show that in the studied area the folk use of plants is alive and still derives from daily practice.

Keywords: Plant traditional uses; Ethnomedicine; National Park of Cilento and Vallo di Diano

Index,

Editor(s): N. Amaresan, M. Senthil Kumar, K. Annapurna, Krishna Kumar, A. Sankaranarayanan,

Beneficial Microbes in Agro-Ecology,

Academic Press,

2020,

Pages 861-912,

ISBN 9780128234143,

https://doi.org/10.1016/B978-0-12-823414-3.20001-4.

(https://www.sciencedirect.com/science/article/pii/B9780128234143200014)

Memory Elvin‐Lewis,

Safety Issues Associated with Herbal Ingredients,

Advances in Food and Nutrition Research,

Academic Press,

Volume 50,

2005,

Pages 219-313,

ISSN 1043-4526,

ISBN 9780120164509,

https://doi.org/10.1016/S1043-4526(05)50007-X.

(https://www.sciencedirect.com/science/article/pii/S104345260550007X)

Abstract: Publisher Summary

This chapter discusses the safety issues associated with herbal ingredients. A wide range of conventional policies controls the availability of herbal products to the general public. The accomplishment of this depends on their derivation and their categorization as medicinals, drugs, botanicals, or dietary supplements. Herbal remedies used as medicines may be traditionally or serendipitously derived, varying in formulation, preparation and standardization, sometimes unreliable as to plant identification or to chemical composition, and depending on their cultural source, infrequently validated in conventional ways, as to efficacy or safety. The detection of adulterated or contaminated herbal products is not easy, because many policies prevent these events to be fully implemented and ways to identify the problems are not easy to achieve. The potential danger of using certain botanicals or their compounds is recognized. Use of herbal remedies by pregnant or nursing mothers can result in the transmission of certain phytochemicals to the fetus or infant. The capacity of children to absorb, distribute, metabolize, and excrete certain substances differs from that of adults. Herbal effects on absorption can also affect the bioavailability of antibiotics.

Index,

Editor(s): Brian Thomas, Brian G Murray, Denis J Murphy,

Encyclopedia of Applied Plant Sciences (Second Edition),

Academic Press,

2017,

Pages 493-539,

ISBN 9780123948083,

https://doi.org/10.1016/B978-0-12-394807-6.18001-3.

(https://www.sciencedirect.com/science/article/pii/B9780123948076180013)

María Jesús Lerma-García, Alfonsina D’Amato, Ernesto F. Simó-Alfonso, Pier Giorgio Righetti, Elisa Fasoli,

Orange proteomic fingerprinting: From fruit to commercial juices,

Food Chemistry,

Volume 196,

2016,

Pages 739-749,

ISSN 0308-8146,

https://doi.org/10.1016/j.foodchem.2015.10.009.

(https://www.sciencedirect.com/science/article/pii/S030881461530008X)

Abstract: Combinatorial peptide ligand library technology, coupled to mass spectrometry, has been applied to extensively map the proteome of orange pulp and peel and, via this fingerprinting, to detect its presence in commercial orange juices and drinks. The native and denaturing extraction protocols have captured 1109 orange proteins, as identified by LC–MS/MS. This proteomic map has been searched in an orange concentrate, from a Spanish juice manufacturer, as well as in commercial orange juices and soft drinks. The presence of numerous orange proteins in commercial juices has demonstrated the genuineness of these products, prepared by using orange fruits as original ingredients. However, the low number of identified proteins in sparkling beverages has suggested that they were prepared with scarce amounts of fruit extract, thus imparting lower quality to the final products. These findings not only increase the knowledge of the orange proteome but also present a reliable analytical method to assess quality and genuineness of commercial products.

Keywords: Orange fruit; Orange juice; LC–MS/MS; Combinatorial peptide ligand library; Protein; Proteomics

David Johnston-Monje, Diana Katherine Castillo-Avila, Manish N. Raizada, Luis Augusto Becerra Lopez-Lavalle,

4.64 - Paying the Rent: How Endophytic Microorganisms Help Plant Hosts Obtain Nutrients☆,

Editor(s): Murray Moo-Young,

Comprehensive Biotechnology (Third Edition),

Pergamon,

2019,

Pages 770-788,

ISBN 9780444640475,

https://doi.org/10.1016/B978-0-444-64046-8.00253-6.

(https://www.sciencedirect.com/science/article/pii/B9780444640468002536)

Abstract: Since plants first colonized land, endophytic bacteria and fungi have been inside them, contributing to their hosts' survival and evolution. Endophyte ecology is an active field of study seeking to understand principles of host strain selection and microbial provenance; although most plants were believed to take up endophytes from soil, transmission through seed or vegetative propagation are also important. This review discusses endophyte contributions to plant nutrient use efficiency (NUE) and their existing or potential applications to agriculture. Endophyte mechanisms to improve plant NUE include formation of extra-root hyphae for nutrient absorption; stimulating root growth; altering plant metabolism to promote nutrient uptake; fixing nitrogen; altering root exudates; colonizing rhizospheres and modifying soil chemistry directly, or even being digested by the root. Although many endophytic strains have been discovered, commercial endophytic inoculants are still mostly limited to arbuscular mycorrhizae, rhizobia, Azospirillum, Pseudomonas and Clavicipitaceous fungi sold in the form of infected grass seed. Wider adoption of endophyte products has been prevented by cheap fertilizer alternatives, unpredictable inoculant responses to host genotype or environmental conditions, competition from endogenous microbes, and poor inoculant establishment and persistence; technical difficulties that new plant microbiome ventures will have to overcome if they are to succeed. There is significant potential to improve agriculture if new strains continue to be discovered, mechanistic understanding of plant-microbe interactions increases, both endophytes and their hosts are genetically enhanced, relevant lab and greenhouse screens can be developed, and methods of effective formulation and deployment are engineered. Novel genes and metabolites from endophytes represent an additional largely untapped resource for future agricultural biotechnologies.

Keywords: ACC deaminase; Biofertilizer; Biological nitrogen fixation; Endophyte; Growth promotion; Inoculant; Microbiome; Mycorrhiza; Neotyphodium; Nutrient uptake; Nutrient use efficiency; Phosphate solubilization; Rhizobia; Rhizosphere; Root; Seed; Soil; Spermosphere

Diego Rivera, Alonso Verde, Concepción Obón, Francisco Alcaraz, Candelaria Moreno, Teresa Egea, José Fajardo, José Antonio Palazón, Arturo Valdés, Maria Adele Signorini, Piero Bruschi,

Is there nothing new under the sun? The influence of herbals and pharmacopoeias on ethnobotanical traditions in Albacete (Spain),

Journal of Ethnopharmacology,

Volume 195,

2017,

Pages 96-117,

ISSN 0378-8741,

https://doi.org/10.1016/j.jep.2016.11.040.

(https://www.sciencedirect.com/science/article/pii/S0378874116320517)

Abstract: Ethnopharmacological relevance

This paper has two overarching aims: (1) presenting the results of studying the Albacete tariff of medicines of 1526 and (2) broadly analyzing the origin and influences of medicinal traditional knowledge in the region of Albacete, Spain. We use historical and modern literature that may have influenced this knowledge. Our primary goal was to determine the ingredients used in the pharmacy in the 16th century CE in Albacete through the analysis of the tariff, and our secondary goal was to investigate until when ingredients and uses present in pharmacy and herbals persisted in later periods.

Methods

The identity of medicines and ingredients was determined by analyzing contemporary pharmacopoeias and classical pharmaceutical references. We analyzed further 21 sources (manuscripts, herbals, and books of medicines, pharmacopoeias, pharmacy inventories, and modern ethnobotanical records) for the presence/absence of ingredients and complex formulations of the tariff. Using factorial and cluster analysis and Bayesian inference applied to evolution models (reversible-jump Markov chain Monte Carlo), we compared textual sources. Finally, we analyzed the medicinal uses of the top 10 species in terms of frequency of citation to assess the dependence of modern ethnobotanical records on Renaissance pharmacy and herbals, and, ultimately, on Dioscorides.

Results

In Albacete 1526, we determined 101 medicines (29 simple drugs and 72 compound medicines) comprising 187 ingredients (85% botanical, 7.5% mineral, and 7.5% zoological substances). All composed medicines appear standardized in the pharmacopoeias, notably in the pharmacopoeia of Florence from 1498. However, most were no longer in use by 1750 in the pharmacy, and were completely absent in popular herbal medicine in Albacete 1995 as well as in Alta Valle del Reno (Italy) in 2014. Among the ingredients present in different formulation are the flowers of Rosa gallica, honey (Apis mellifera), the roots of Nardostachys jatamansi, and Convolvulus scammonia, pistils of Crocus sativus, grapes and raisins (Vitis vinifera), rhizomes of Zingiber officinale, bark of Cinnamomum verum, leaves and fruits of Olea europaea, mastic generally of Pistacia lentiscus, and wood of Santalum album. The statistical analysis of sources produces four well-separated clusters (Renaissance Herbals and Pharmacopoeias, Ethnobotany and Folk Medicine, Old phytotherapy, and Modern phytotherapy including Naturopathy) confirming our a priori classification. The clade of Renaissance Herbals and Pharmacopoeias appears separated from the rest in 97% of bootstrapped trees. Bayesian inference produces a tree determined by an initial set of two well-distinct core groups of ingredients: 64, locally used in Mediterranean Europe during centuries; and 45, imported, used in pharmacy during centuries. Complexity reached its maximum in Albacete 1526 and contemporary pharmacopoeias, gradually decreasing over time. The analysis of medicinal uses of the top 10 ingredients showed low coincidence between Dioscorides and different Renaissance herbals or medical treatises and of all of them with ethnobotany in Albacete.

Conclusions

Regarding our question: is there something new under the sun? In some aspects, the answer is “No”. The contrast between expensive drugs, highly valued medicines, and unappreciated local wild medicinal plants persists since the Salerno's school of medicine. Old medicine in Mediterranean Europe, as reflected by Albacete 1526 tariff of medicines, involved strict formulations and preferences for certain ingredients despite other ingredients locally available but underappreciated. This confirms the fact that any system of medicine does not get to use all available resources. Ethnobiological records of materia medica, in rural areas of Albacete, describe systems with a high degree of stability and resilience, where the use of local resources, largely wild but also cultivated, is predominant in contrast with the weight of imported exotic products in pharmacy.

Keywords: Ethnobotany; Herbals; Medicinal plants; Multivariate and Bayesian analysis; Pharmacopoeias; Traditional medicine

Diego Rivera, Alonso Verde, José Fajardo, Concepción Obón, Vicente Consuegra, José García-Botía, Segundo Ríos, Francisco Alcaraz, Arturo Valdés, Alejandro del Moral, Emilio Laguna,

Ethnopharmacology in the Upper Guadiana River area (Castile-La Mancha, Spain),

Journal of Ethnopharmacology,

Volume 241,

2019,

111968,

ISSN 0378-8741,

https://doi.org/10.1016/j.jep.2019.111968.

(https://www.sciencedirect.com/science/article/pii/S0378874119302661)

Abstract: Ethnopharmacological relevance

Determining traditional remedies for human pathologies is relevant, when compared with the standard materia medica of the pharmacopoeias and dietary supplement databases, because we can assess the species and uses that have been previously studied and target understudied species for further pharmacological investigation.

Background

The aim of this study was to systematically record and analyze medicinal uses of natural resources (Plantae, Animalia, Fungi and minerals), mostly local, in the territories adjoining the upper Guadiana River and its tributaries. We were particularly interested in recording resources and pathologies linked to wetland areas, especially in the National Parks Las Tablas de Daimiel and Cabañeros. Wetlands are interesting because they present a double face in relation with human health: Wetlands furnish hydration, safe water, nutrition, and medicinal resources; are places from which people derive their livelihood. However wetlands are also sites of exposure to pollution or toxicants, and infectious diseases; and sites of physical hazards. We wanted to identify procedures for preparation of medicinal formulae and routes of administration. We also intended to detect whether a geographical pattern exists or not in our records in relation to the use of local resources.

Methods

We used semi-structured interviews with one-to-one informants or groups, from 1998 to 2018. Raw data were introduced in a Firebird database and analyzed. To identify ingredients and pathologies we consulted local floras and epidemiological literature. Finally, we compared documented pathologies, remedies and ingredients in the historical context of medicinal uses of natural resources in Castile-La Mancha and especially in Ciudad Real.

Results

126 pathologies and 220 species furnishing ingredients have been recorded from the interviews. In total, 188 are plants and 20 animals. The most commonly used species include Malva sylvestris, Phlomis lychnitis, Genista tridentata and Thymus mastichina. Most records refer to flowers, or fruits, of locally available plant species, classified as Mediterranean, European or widespread that belong to the Lamiaceae, Compositae or Leguminosae. Ingredients which are collected in open shrublands, known as “garrigue”, and dry grasslands furnish a relevant proportion of records while the imported ingredients remain marginal. The contribution of wetlands, riverine habitats and irrigated fields and gardens as a source of medicinal resources is 36% of the records. It is relatively high considering its limited presence in terms of total extension within the study area. The most frequently reported diseases are respiratory, gastrointestinal, dermatological and infectious or parasitic.

Conclusions

Along the Guadiana River in the Ciudad Real province exists a wide and deep knowledge of traditional remedies for the treatment of common pathologies, based fundamentally on the use of local flora, fauna and mineral resources. The uses and ingredients documented are useful for further pharmacological investigation to improve health care for a wide range of pathologies.

Keywords: Ethnobotany; Herbals; Medicinal plants; Traditional medicine; Wetlands

APPENDIX - COMMON AND SCIENTIFIC NAMES FOR PLANTS, VERTEBRATES, AND SELECTED INVERTEBRATES,

Editor(s): ARTHUR C. BENKE, COLBERT E. CUSHING,

Rivers of North America,

Academic Press,

2005,

Pages 1105-1134,

ISBN 9780120882533,

https://doi.org/10.1016/B978-012088253-3/50028-6.

(https://www.sciencedirect.com/science/article/pii/B9780120882533500286)

Andrea Pieroni, Cassandra L. Quave, Rocco Franco Santoro,

Folk pharmaceutical knowledge in the territory of the Dolomiti Lucane, inland southern Italy,

Journal of Ethnopharmacology,

Volume 95, Issues 2–3,

2004,

Pages 373-384,

ISSN 0378-8741,

https://doi.org/10.1016/j.jep.2004.08.012.

(https://www.sciencedirect.com/science/article/pii/S0378874104003836)

Abstract: An ethnopharmacognostic survey on the traditional pharmaceutical knowledge (TPhK) of old and newly introduced natural remedies used for healing humans in a small mountainous area in Central Lucania, inland southern Italy, was carried out using classical ethnographical and ethnobiological methods. Approximately 110 remedies of plant origin (belonging to 103 botanical taxa), 30 of animal origin and 20 mineral or industrial (non-pharmaceutical) products were recorded. Among these remedies, the common use of the aerial parts of Hypericum hircinum and the leaves of Morus alba against cough, and the uncommon uses of Salvia argentea leaves as a haemostatic, of Erigeron acer roots to relieve tooth-aches and arthritic pains, and Elaphe quatuorlineata snake fat for rheumatism are reported for first time in Italy. Moreover, diverse medicinal plants used for uncommon medical purposes and a few biological ingredients used in food preparations with the aim to improve human health were identified. Pharmacological and toxicological considerations relating to possible applications of the recorded traditional knowledge in modern evidence-based medicine are discussed as well. The data that we present here could suggest new inputs for further phytochemical and pharmacological studies among Mediterranean folk pharmacopoeias, and also for sustaining environmentally integrated projects focused on of the maintenance of TPhK via breeding or controlled gathering activities of local medicinal species.

Keywords: Ethnopharmacy; Ethnobotany; Pharmacognosy; Medicinal plants; Italy

Gizem Bulut, Mehmet Zeki Haznedaroğlu, Ahmet Doğan, Halil Koyu, Ertan Tuzlacı,

An ethnobotanical study of medicinal plants in Acipayam (Denizli-Turkey),

Journal of Herbal Medicine,

Volume 10,

2017,

Pages 64-81,

ISSN 2210-8033,

https://doi.org/10.1016/j.hermed.2017.08.001.

(https://www.sciencedirect.com/science/article/pii/S2210803317300556)

Abstract: A comprehensive ethnobotanical study was conducted in Acipayam, situated in the western part of Turkey. This paper includes details of plants used in folk medicine and ethnopharmacological information obtained during this study. The aim of the authors was to collect and identify plants used by local people for therapeutic purposes and to present information about traditional herbal medicine. Plant specimens collected during field‐work form the subject of this investigation. Information was obtained by means of open and semi-structured interviews with local people. In addition, cultural importance index (CI) and use report (UR) values were calculated. Ninety-one taxa of plants used in folk medicine and belonging to 38 families were identified in this study. Of these, 82 species were wild, and 9 species were cultivated. The most common families were Lamiaceae (18.7%), Asteraceae (14.3%) and Rosaceae (6.6%). Consequently, 191 medicinal uses (remedies) of 91 taxa were recorded. According to the use reports (UR), the most important medicinal plants were Cydonia oblonga (99 UR), Juniperus oxycedrus subsp. oxycedrus (99 UR), Pinus brutia (98 UR), Hypericum perforatum (90 UR), Viscum album subsp. austriacum (82 UR) and Salvia tomentosa (80 UR). Infusion (38.2%) was the most common preparation method used within the research area. The ethnomedicinal capabilities of eight species (Amelanchier parviflora var. dentata, Echinops viscosus subsp. bithynicus, Onopordum sibthorpianum, Origanum hypericifolium, Quercus trojana, Salvia adenophylla, Sideritis montana. subsp. remota and Tamarix smyrnensis) have been recorded for the first time in Turkey.

Keywords: Ethnobotany; Folk medicinal plants; Acipayam; Denizli; Turkey

A. Merzouki, F. Ed-derfoufi, J. Molero Mesa,

Contribution to the knowledge of Rifian traditional medicine. II: Folk medicine in Ksar Lakbir district (NW Morocco),

Fitoterapia,

Volume 71, Issue 3,

2000,

Pages 278-307,

ISSN 0367-326X,

https://doi.org/10.1016/S0367-326X(00)00139-8.

(https://www.sciencedirect.com/science/article/pii/S0367326X00001398)

Abstract: An ethnobotanical survey of the medicinal plants used by the local population of the Ksar Lakbir district (NW Morocco) was conducted. One hundred and eighty-six species from 61 botanical families were recorded as well as their uses and modes of administration. Quantitative ethnopharmacological data (medicinal plant knowledge and use indices) were also evaluated and discussed.

Keywords: Ethnobotany; Herbal medicine; Ksar Lakbir; Morocco

Subject Index,

Editor(s): Atta-ur-Rahman,

Studies in Natural Products Chemistry,

Elsevier,

Volume 24, Part E,

2000,

Pages 1139-1156,

ISSN 1572-5995,

ISBN 9780444506436,

https://doi.org/10.1016/S1572-5995(00)80061-7.

(https://www.sciencedirect.com/science/article/pii/S1572599500800617)

Daniela L. Bordón, Leonardo D. Villalba, Mario L. Aimar, Juan J. Cantero, Ana M. Vázquez, Stella M. Formica, Claudio R. Krapacher, Laura I. Rossi,

Weeds as biocatalysts in the stereoselective synthesis of chiral phenylethanols used as key intermediates for pharmaceuticals,

Biocatalysis and Agricultural Biotechnology,

Volume 4, Issue 4,

2015,

Pages 493-499,

ISSN 1878-8181,

https://doi.org/10.1016/j.bcab.2015.08.001.

(https://www.sciencedirect.com/science/article/pii/S1878818115000900)

Abstract: This paper describes the search for novel vegetal biocatalysts for the stereoselective reduction of prochiral phenylketones. In this study, twenty native weeds were tested and Eryngium horridum Malme (Apiaceae) was proven to be an effective biocatalyst for the stereoselective reduction of acetophenone to (S)-1-phenylethanol (96% conversion, >99.9 e.e.%). Using this biocatalyst, fourteen chiral (S)-phenylethanols with excellent enantiomeric excesses (>98%) and variable conversions (30–100%) were obtained.

Keywords: Eryngium horridum; Biocatalysis; Bioreduction; Weeds; Substituted acetophenones; Chiral phenylethanols

Vinaya Chandran, Hitha Shaji, Linu Mathew,

7 - Endophytic microbial influence on plant stress responses,

Editor(s): Ajay Kumar, Radhakrishnan E.K,

Microbial Endophytes,

Woodhead Publishing,

2020,

Pages 161-193,

ISBN 9780128196540,

https://doi.org/10.1016/B978-0-12-819654-0.00007-7.

(https://www.sciencedirect.com/science/article/pii/B9780128196540000077)

Abstract: Endophytic microorganisms have diverse roles in promoting plant growth and adaptation. Their beneficial roles are manifold. Secretion of plant growth promoting substances, increasing nutrient availability, producing protective secondary metabolites, and improving biotic and abiotic stress resistance are some of the advantages of harboring endophytic microorganisms. They are particularly useful in tiding over abiotic stress responses like salinity, chilling, drought, heavy metal pollution, and other extremes of environment. Compared to rhizopheric bacteria, endophytes are more adept in habitat-associated symbiosis. Osmotic adjustment, detoxification, plant growth hormone production, and enhancing nutrient availability and solubility by them, help the host plants in tiding over salinity, drought, nutrient depletion, and heavy metal toxicity. Activating plant defense responses genes by the endophytes play a major role in preventing pathogen invasion and thereby, disease prevention. In the present chapter, the authors describe the plant stress alleviating effects of endophytes with their possible application in agriculture.

Keywords: plant stress; endophytes; plant defense gene activation; plant growth promotion; abiotic stress

Murali Mohan Sharaff, Gangavarapu Subrahmanyam, Amit Kumar, Ajar Nath Yadav,

Chapter 5 - Mechanistic understanding of the root microbiome interaction for sustainable agriculture in polluted soils,

Editor(s): Ali Asghar Rastegari, Ajar Nath Yadav, Neelam Yadav,

New and Future Developments in Microbial Biotechnology and Bioengineering,

Elsevier,

2020,

Pages 61-84,

ISBN 9780128205266,

https://doi.org/10.1016/B978-0-12-820526-6.00005-1.

(https://www.sciencedirect.com/science/article/pii/B9780128205266000051)

Abstract: In agricultural soils, the root microbiome plays a significant role in promoting plant growth for enhanced yield and also regulates soil fertility. It is important to understand the root microbiome of the different crop plants for improving the sustainable agriculture in contaminated soils. Plants and bacteria can form specific associations in which the plant provides the bacteria with a specific carbon source that induces the microbiome to reduce the phytotoxicity of heavy metals/organic pollutants in the contaminated soils. Thus, toxicity of organic/inorganic pollutants to plants can be relieved by introduction of plant growth-promoting bacteria (PGP) with beneficial effects on plant development. These microbiomes have the ability to promote plant growth directly or indirectly through release of hormones or enzymatic or organic acid release or by reducing the ethylene stress or by nutrient assimilation and supply to plants. The present chapter highlights the composition and diversity of the root microbiome in polluted sites/soils and their potential applications on alleviating heavy metal stress in different agricultural crops. Examples of promising PGP bacteria involved in the reduction of metal accumulation in plant aerial parts have been summarized. Microbe-mediated remediation of organic and inorganic pollutants are discussed. Further, the chapter highlights different mechanisms of plant growth-promoting rhizobacteria (PGPRs) in alleviating heavy metal toxicity in agricultural crops.

Keywords: Heavy metal contamination; Phytoremediation; Phytotoxicity; Plant growth-promoting rhizobacteria; Polluted soils; Root microbiome

I. Nyamwasa, K. Li, A. Rutikanga, D.N.T. Rukazambuga, S. Zhang, J. Yin, C. Ya-zhong, X.X. Zhang, X. Sun,

Soil insect crop pests and their integrated management in East Africa: A review,

Crop Protection,

Volume 106,

2018,

Pages 163-176,

ISSN 0261-2194,

https://doi.org/10.1016/j.cropro.2017.11.017.

(https://www.sciencedirect.com/science/article/pii/S0261219417303459)

Abstract: Soil-dwelling pests are generally poorly documented in East Africa due to their cryptic nature, which complicates monitoring, so the aim of this review is to compile and elucidate the current soil insect pest and management situation in this region. Fifty-five (55) soil insect pests are reported from across East Africa, and twenty (36%) and seventeen (30%) species of Scarabaeidae and Termitidae, respectively, and three families (Agromyzidae, Apionidae and Curculionidae) were found to be the most notorious soil insect pests in the region by far. Multiple species within the aforementioned families have been reported as the leading soil insect pests threatening the production of major crops in East Africa and include the banana weevil, Cosmopolites sordidus Germar; the sweet potato weevil, Cylas formicarius Fabricius; the bean maggots Ophiomyia spencerella Greathead, O. phaseoli Tryon and O. centrosematis de Meij; the cutworm, Agrotis segetum Schiff; and several species of termites and white grubs. The major control options rely heavily on preventive measures and to lesser extent, on direct control as a last resort. This review provides insight into the soil insect pest communities in East Africa as well as current control options and identifies knowledge gaps, such as an insufficient understanding of insect biology and ecology, highlights the lack of action threshold values as well as localized recommended rates of insecticides and underlines the need for education on pesticide use.

Keywords: Soil insect pests; Integrated pest management (IPM); Insecticides; East Africa

Pulok K. Mukherjee,

Chapter 14 - Therapeutic Evaluation of Herbs With Enzyme Inhibition Studies,

Editor(s): Pulok K. Mukherjee,

Quality Control and Evaluation of Herbal Drugs,

Elsevier,

2019,

Pages 539-571,

ISBN 9780128133743,

https://doi.org/10.1016/B978-0-12-813374-3.00014-4.

(https://www.sciencedirect.com/science/article/pii/B9780128133743000144)

Abstract: In the drug discovery and development process, there is a growing interest in the study of enzymes with the aim of identifying inhibitory molecules. Several drug molecules have been derived from natural resources that offer potential therapeutic benefits against several metabolic disorders. Enzymes offer essential catalytic roles in many physiological processes that may be altered in disease states. The development of enzyme inhibitors as therapeutic agents involves the optimization of multiple pharmacological properties beyond the affinity and selectivity of the molecule for its target enzyme. There are several enzymes involved in major biochemical pathways which play a great role in pathogenesis of disease viz. acetyl cholinesterase, butyl cholinesterase, alpha-glucosidase, angiotensin converting enzyme, pancreatic lipase, HMG-CoA reductase, carbonic anhydrase, aldose reductase, etc. corresponding to several disease conditions like Alzheimer's, high blood pressure, cardiovascular disease, hyperlipidemia, diabetes, etc. Inhibition or induction of this enzymatic activity has been found very effective to overcome the diseased state. This chapter highlights on several aspects of the role of the enzymes in disease pathophysiology, in vitro methods for assay of those enzyme inhibition with example of several medicinal plants having potential enzyme inhibitory activity.

Keywords: Enzyme kinetics; Inhibition; Alpha-glucosidase; Angiotensin converting enzyme; Pancreatic lipase; HMG-Coa reductase; Carbonic anhydrase; Aldose reductase

Rebecca Clarke, Craig G. Webster, Monica A. Kehoe, Brenda A. Coutts, Sonya Broughton, Mark Warmington, Roger A.C. Jones,

Epidemiology of Zucchini yellow mosaic virus in cucurbit crops in a remote tropical environment,

Virus Research,

Volume 281,

2020,

197897,

ISSN 0168-1702,

https://doi.org/10.1016/j.virusres.2020.197897.

(https://www.sciencedirect.com/science/article/pii/S0168170219308962)

Abstract: In the remote Ord River Irrigation Area (ORIA) in tropical northwest Australia, severe Zucchini yellow mosaic virus (ZYMV) epidemics threaten dry season (April-October) cucurbit crops. In 2016–2017, wet season (November-March) sampling studies found a low incidence ZYMV infection in wild Cucumis melo and Citrullus lanatus var. citroides plants, and both volunteer and garden crop cucurbits. Such infections enable its persistence in the wet season, and act as reservoirs for its spread to commercial cucurbit crops during the dry season. Tests on 1019 samples belonging to 55 species from 23 non-cucurbitaceous plant families failed to detect ZYMV. It was also absent from wild cucurbit weeds within sandalwood plantations. The transmission efficiencies of a local isolate by five aphid species found in the ORIA were: 10 % (Aphis craccivora), 7% (A. gossypii), 4% (A. nerii), and 0% (Rhopalosiphum maidis and Hysteroneura setariae). In 2016–2017, in all-year-round trapping at five representative sites, numbers of winged aphids caught were greatest in July-August (i.e. mid growing season) but varied widely between trap sites reflecting local aphid host abundance and year. Apart from one localised exception in 2017, flying aphid numbers caught and ZYMV spread in data collection blocks during 2015–2017 resembled what occurred commercial cucurbit crops. When ZYMV spread from external infection sources into melon blocks, its predominant spread pattern consisted of 1 or 2 plant infection foci often occurring at their margins. In addition, when plants of 29 cucurbit cultivars were inoculated with an ORIA isolate and two other ZYMV isolates and the phenotypes elicited were compared, they resembled each other in overall virulence. However, depending upon isolate-cultivar combination, differences in symptom expression and severity occurred, and one isolate caused a systemic hypersensitive phenotype in honeydew melon cvs Estilo and Whitehaven. When the new genomic RNA sequences of 19 Australian isolates were analysed, all seven ORIA isolates fitted within ZYMV phylogroup B, which also included two from southwest Australia, whereas the remaining 10 isolates were all within minor phylogroups A–I or A–II. Based on previous research and the additional knowledge of ZYMV epidemic drivers established here, an integrated disease management strategy targeting ZYMV spread was devised for the ORIA’s cucurbit industry.

Keywords: Epidemiology; Tropics; Cucurbit crops; Zucchini yellow mosaic virus; Aphid vectors; Epidemic drivers; Alternative hosts; Infection reservoirs; Aphid trapping; Data collection blocks; Spatiotemporal spread patterns; Cucurbit cultivar reactions; Phylogeny; Integrated disease management

Anushka Mootoosamy, M. Fawzi Mahomoodally,

Ethnomedicinal application of native remedies used against diabetes and related complications in Mauritius,

Journal of Ethnopharmacology,

Volume 151, Issue 1,

2014,

Pages 413-444,

ISSN 0378-8741,

https://doi.org/10.1016/j.jep.2013.10.069.

(https://www.sciencedirect.com/science/article/pii/S0378874113007952)

Abstract: Ethnopharmacological relevance

Notoriously, the tropical island of Mauritius has one of the highest prevalence of diabetes worldwide and the economic burden associated with it is alarming. The use of native remedies (NRs) is well anchored in the local culture and it continues to be the cornerstone of therapy for diabetic patients. However, there is currently a dearth of updated primary data on NRs used by Mauritians against diabetes and diabetes related complications (DRCs). This study was therefore designed to record, analyze and document orally transmitted ethnopharmacological knowledge from diabetic patients and traditional medicine practitioners (TMPs) in Mauritius concerning NRs commonly used against diabetes and DRCs which might open new avenues to initiate novel antidiabetic drugs discovery.

Materials and methods

Data was collected following interviews from diabetic patients (n=328) and TMPs (n=20). Eleven quantitative indexes, namely informant consensus factor (FIC), fidelity level (FL), use value (UV), relative frequency of citation (RFC), relative importance (RI), cultural importance index (CII), index of agreement on remedies (IAR), cultural agreement index (CAI), quality use value (QUV), quality use agreement value (QUAV) and ethnobotanicity index (EI) were calculated. Statistical analysis such as Pearson correlation and Chi-squared test were performed to determine any association.

Results

A total of 111 plant species distributed over 56 families, 30 polyherbal formulations and 16 animal species were documented to be traditionally used against diabetes and DRCs. For the first time 8 endemic plants have been recorded to be used against diabetes and DRCs from Mauritius. The most encountered medicinal plant family was Asteraceae. According to the EI, 16.2% of the native plants in Mauritius were used against diabetes and DRCs. As far as we know, Vangueria madagascariensis, Apium graveolens, Petroselinum crispum and Rubus alceifolius with high RFC values are recorded against diabetes and DRCs for the first time. Sociodemographic characteristics (age, gender, income, religious belief, education and residence) were found to significantly (p<0.05) influence the use of NRs. The average FIC for all ailments for plant and animal products were 0.94 and 0.87 respectively. Bryophyllum pinnatum, a native plant to Mauritius scored a high FL value (100%) used against diabetic neuropathy, Allium sativum had the highest RI value (2.00) due to its versatility, Aloe vera had the highest RFC (0.61), the CII (0.640) and the highest CAI value (0.635), Psidium guajava had the highest QUAV (0.961) which indicates its high bioactivity and Allium cepa was reported as the most effective plant species (QUV=0.965). According to UV, the most important species was Morinda citrifolia (1.21). Panoply of animal products were reported whereby fish (39.7%) was recorded as the most utilised zootherapy and Salmo salar scored the highest FL (100%) for diabetes. Some animal species (n=14) not previously documented against diabetes and DRCs are reported in the present study.

Conclusion

Our present investigation revealed that the use of NRs constitutes the common legacy of Mauritians and despite the penetration of allopathic medicine; NRs continue to play a crucial role in the primary health care system of Mauritius. To this effect, it is of uttermost importance to record this knowledge before it disappears. In addition, further experimental investigations are required to elucidate the pharmacological properties of the reported medicinal flora and fauna of Mauritius.

Keywords: Mauritius; Diabetes; Diabetes related complications; Native remedies; Herbal remedies; Animal-based remedies

Airy Gras, Teresa Garnatje, Neus Ibáñez, Jordi López-Pujol, Neus Nualart, Joan Vallès,

Medicinal plant uses and names from the herbarium of Francesc Bolòs (1773–1844),

Journal of Ethnopharmacology,

Volume 204,

2017,

Pages 142-168,

ISSN 0378-8741,

https://doi.org/10.1016/j.jep.2017.04.002.

(https://www.sciencedirect.com/science/article/pii/S0378874116317858)

Abstract: Ethnopharmacological relevance

Ethnobotany takes into account past uses to be projected into the present and future. Most current ethnobotanical research is focused, especially in industrialised countries, on obtaining information of plant uses from elderly people. Historical ethnobotany is less cultivated, although papers have demonstrated its interest. Particularly poor, but potentially very relevant, is the attention paid to historical herbaria as a source of data on useful plants.

Aims of the study

Bearing this in mind, we studied the herbarium of the Catalan pharmacist and naturalist Francesc Bolòs (1773–1844), which contains information on medicinal uses and folk names, with the aim of establishing a catalogue of plants and uses and tracing them through old and contemporary literature.

Methodology

The ca. 6000 plant specimens of this herbarium were investigated to assess those including plant uses and names. These taxa have been thoroughly revised. The data have been tabulated, their biogeographic profile, possible endemic or threatened status, or invasive behaviour have been assessed, and the content regarding medicinal uses, as well as folk names, has been studied. The medicinal terms used have been interpreted as per current days’ medicine. The popular names and uses have been compared with those appearing in a certain number of works published from 11th to 20th centuries in the territories covered by the herbarium and with all the data collected in 20th and 21st centuries in an extensive database on Catalan ethnobotany.

Results

A total of 385 plant specimens (381 taxa) have been detected bearing medicinal use and folk names information. We collected data on 1107 reports of plant medicinal properties (in Latin), 32 indications of toxicity, nine reports of food use, and 123, 302 and 318 popular plant names in Catalan, Spanish and French, respectively. The most quoted systems are digestive, skin and subcutaneous tissue (plus traumatic troubles) and genitourinary. Relatively high degrees of coincidence of plant names and uses in the herbarium and the literature comparison set have been found. Of the taxa contained in this medicinal herbarium, 294 were native to the Iberian Peninsula, and 86 were alien. Neither endemic nor threatened taxa have been detected, whereas a considerable portion of the alien taxa shows invasive behaviour at present.

Conclusions

Our analyses indicate a certain degree of consistency between the medicinal uses of plants recorded in this 18th and 19th century herbarium and the records found in the literature and in recent ethnobotanical datasets, accounting for the robustness of pharmaceutical ethnobotanical knowledge in the area considered. Data appearing on the specimen labels are numerous, pointing out the herbarium as a relevant source of ethnopharmacological information. Special attention should be paid to some original uses contained in the herbarium's labels for further investigation on plant properties and drug design.

Keywords: Catalonia; Herbaria; Historical ethnobotany; Historical ethnopharmacology; Iberian Peninsula; Medicinal plants

P.J. Holloway, C.E. Jeffree,

SECONDARY PRODUCTS | Epicuticular Waxes,

Editor(s): Brian Thomas,

Encyclopedia of Applied Plant Sciences,

Elsevier,

2003,

Pages 1190-1204,

ISBN 9780122270505,

https://doi.org/10.1016/B0-12-227050-9/00134-4.

(https://www.sciencedirect.com/science/article/pii/B0122270509001344)

Shafi Ullah, Muhammad Rashid Khan, Naseer Ali Shah, Sayed Afzal Shah, Muhammad Majid, Muhammad Asad Farooq,

Ethnomedicinal plant use value in the Lakki Marwat District of Pakistan,

Journal of Ethnopharmacology,

Volume 158, Part A,

2014,

Pages 412-422,

ISSN 0378-8741,

https://doi.org/10.1016/j.jep.2014.09.048.

(https://www.sciencedirect.com/science/article/pii/S037887411400703X)

Abstract: Aim of the study

Medicinal plants are regional treasures for the treatment of many ailments. The present research investigated and documented knowledge of indigenous commonly used medicinal plants, including traditional names, preparations and uses, in the Lakki Marwat District of Pakistan. The information gathered was statistically analyzed using the ICF method to establish baseline data for more comprehensive investigations of bioactive compounds of indigenous medicinal plants.

Materials and Methods

Direct interviews of 78 informants were conducted during 2013–2014 to identify the preparations and uses of indigenous medicinal plants. Data were analyzed using various quantitative tools, such as use value, factor informant consensus and fidelity level.

Results

A total of 62 species of flowering plants belonging to 34 families and 57 genera were reportedly used as ethnomedicines in the study area. Fabaceae, Brassicaceae, Apocynaceae, Solanaceae, Apiaceae, Poaceae, Zygophyllaceae, Asteraceae and Euphorbiaceae were the main plant families that comprised ethnobotanically important plant species. Traditional healers most frequently used aerial parts of plants. The following medicinal species were the most important in the present study with the highest use values (UV): Plantago ovata Forsk.(F. Plantaginaceae), Lawsonia inermis L.(F. Lythraceae), Calotropis procera (Aiton) Dryand.(F. Apocynaceae), Peganum harmala L.(F. Zygophyllaceae), Fagonia indica Burm.f. (F. Zygophyllaceae), Carthamus oxyacantha M.Bieb. (F. Asteraceae), Datura metel L. (F. Solanaceae) and Eruca vesicaria (L.) Cav. (F. Brassicaceae). Respiratory, otic, gastrointestinal and neurological ailments were the main categories that were classified as per factor informant consensus (Fic). The greatest number of species was used to cure gastrointestinal and andrological/gynecological problems. The highest fidelity level (Fl=100%) was achieved by Plantago ovata Forsk. (F. Plantaginaceae) to cure cardiovascular disorders.

Conclusion

The results of present study reveal that this enormous wealth of medicinal plants played an important role in the health care of the villagers in the study area. In addition, species with high use values (UV) might provide valuable leads for further pharmacological investigations.

Keywords: Lakki Marwat; Ethnomedicinal plants; Use value; Factor informant consensus; Fidelity level

Valentí Rull,

Chapter 1 - The island at present,

Editor(s): Valentí Rull,

Paleoecological Research on Easter Island,

Elsevier,

2020,

Pages 1-40,

ISBN 9780128227275,

https://doi.org/10.1016/B978-0-12-822727-5.00001-5.

(https://www.sciencedirect.com/science/article/pii/B9780128227275000015)

Abstract: This chapter is a succinct summary of the present-day Easter Island's conditions, whose aim is not to provide a thorough characterization but to situate the reader in context and to introduce the locations, terms, and concepts that are discussed throughout the book. The main subjects addressed are geography, geology, climate, hydrology, soils, land use, flora, vegetation, archaeological heritage, and conservation of both the natural systems and cultural legacy. Emphasis is placed on the different ecological and landscape features before and after human settlement—albeit not in the processes and causes involved, which are discussed in further chapters—and the handicap that this deep transformation may represent for paleoecological reconstruction.

Keywords: Geography; Geology; Climate; Soils; Flora; Vegetation; Cultural legacy; Conservation

Kazhila C. Chinsembu,

Diabetes mellitus and nature’s pharmacy of putative antidiabetic plants,

Journal of Herbal Medicine,

Volume 15,

2019,

100230,

ISSN 2210-8033,

https://doi.org/10.1016/j.hermed.2018.09.001.

(https://www.sciencedirect.com/science/article/pii/S221080331830040X)

Abstract: Globally, diabetes causes about 1.5 million deaths per year. Data predicts that the current global epidemiological burden of diabetes is increasing alongside its long-term life threatening sequelae and side-effects from synthetic antidiabetic drugs. Challenges in the public healthcare delivery system, inadequate human and financial resources, expensive antidiabetic drugs coupled with their limited availability, efficacy and tolerability, and a higher priority for subventions to the control of communicable diseases such as HIV/AIDS than to drugs for non-communicable conditions especially diabetes have opened new vistas for diabetics to seek complementary and alternative medicines. As a corollary, there is a renewed momentum backed by the World Health Organization to discover newer, cheaper and better antidiabetic agents from medicinal plants. As data on putative antidiabetic properties of plants remain scattered, this review provides a new synthesis of antidiabetic plants from Africa, Central America, Mexico, South Asia, and Iran. In the countries mentioned in this review, numerous plant species decrease blood sugar levels by inhibiting the enzymes α-amylase and α-glucosidase. Other antidiabetic plants restore pancreatic cells, improve insulin secretion and sensitivity, decrease metabolic syndrome in type 2 diabetes patients in addition to exerting antioxidant and hepatoprotective functions. Mechanisms of action are mediated by phytochemical agents including saponins, polyphenols, ellagitannins, triterpenes, and elements such as Mg, P, Ca, K, Mn, Cu, Zn, S, Cr, Co, Ni and V. These plant compounds and elements may be included into new and more efficacious herbal drugs and nutraceuticals to lessen the global burden of diabetes.

Keywords: Diabetes; Plants; Alpha-amylase; Alpha-glucosidase; Phytochemical compounds; Elements

J.R.S Tabuti, K.A Lye, S.S Dhillion,

Traditional herbal drugs of Bulamogi, Uganda: plants, use and administration,

Journal of Ethnopharmacology,

Volume 88, Issue 1,

2003,

Pages 19-44,

ISSN 0378-8741,

https://doi.org/10.1016/S0378-8741(03)00161-2.

(https://www.sciencedirect.com/science/article/pii/S0378874103001612)

Abstract: We present here an inventory of the medicinal plants of Bulamogi county in Uganda, including their medicinal use, preparation and administration modes. Fieldwork for this study was conducted between June 2000 and June 2001 using semi-structured interviews, questionnaires, and participant observation as well as transect walks in wild herbal plant collection areas. We recorded 229 plant species belonging to 168 genera in 68 families with medicinal properties. A large proportion of these plants are herbaceous. The medicinal plants are mainly collected from the wild. Some species, such as Sarcocephalus latifolius (Smith) Bruce, are believed by the community to be threatened by unsustainable intensities of use and patterns of harvesting. Particularly vulnerable are said to be the woody or the slow growing species. Herbal medicines are prepared as decoctions, infusions, powders, or as ash, and are administered in a variety of ways. Other concoctions consist of juices and saps. The purported therapeutic claims await validation. Validation in our opinion can help to promote confidence among users of traditional medicine, and also to create opportunities for the marketing of herbal medicines and generate incomes for the community. The processing, packaging and storage of herbal medicines is substandard and require improvement.

Keywords: Herbal medicines; Medicinal plants; Sustainable utilisation; Uganda

Carmen Van Mechelen, Thierry Dutoit, Martin Hermy,

Mediterranean open habitat vegetation offers great potential for extensive green roof design,

Landscape and Urban Planning,

Volume 121,

2014,

Pages 81-91,

ISSN 0169-2046,

https://doi.org/10.1016/j.landurbplan.2013.09.010.

(https://www.sciencedirect.com/science/article/pii/S0169204613001898)

Abstract: Offering a wide array of benefits, green roofs have become an important tool for improving urban environmental quality mainly in regions with a temperate climate. However, green roofs seem to perform relatively bad in the Mediterranean, as plant species commonly used are often not adapted to cope with the additional stress factors associated with this climate. The habitat template hypothesis states that potential species can be found in habitats with similar conditions as on extensive green roofs. In this study, natural open habitats in southern France are described and variation in species composition in relation to environmental factors is analyzed. 372 local species recorded in 20 locations were grouped in four major vegetation types. These results are compared with a list of species commonly used on green roofs in NW Europe. 79% of the species found in these open habitats are currently not used on green roofs. Ten highly relevant plant traits for extensive green roofs were then used to screen the species found during the field work. 28 species scored highly in this screening procedure, indicating good potential. Annual species are currently rarely regarded for green roof purposes but in the habitat template context, this life form is an important part of Mediterranean vegetation and should be considered in green roof design. This research offers the ecological fundamental knowledge necessary for further selection and testing of species and final implementation into a successful green roof system.

Keywords: Mediterranean climate; Habitat template; Southern France; Biodiversity; Vegetated roofs; Plant traits

Fernanda Leitão, Suzana Guimarães Leitão, Viviane Stern da Fonseca-Kruel, Ines Machline Silva, Karine Martins,

Medicinal plants traded in the open-air markets in the State of Rio de Janeiro, Brazil: an overview on their botanical diversity and toxicological potential,

Revista Brasileira de Farmacognosia,

Volume 24, Issue 2,

2014,

Pages 225-247,

ISSN 0102-695X,

https://doi.org/10.1016/j.bjp.2014.04.005.

(https://www.sciencedirect.com/science/article/pii/S0102695X14000179)

Abstract: Medicinal plants have been used for many years and are the source of new active substances and new drugs of pharmaceutical interest. The popular knowledge contained in the open- air markets is studied through urban ethnobotany, and is a good source of information for ethnobotanical research. In this context, we surveyed the literature on works concerning open-air markets in the State of Rio de Janeiro to gather knowledge of the commercialized plants therein. A literature search resulted in ten studies with 376 listed species, distributed in 94 families and 273 genera. Asteraceae family had the greater representation, followed by Lamiaceae and Fabaceae. Solanum was the most frequent genus. Two hundred and twenty four species could be considered potentially toxic or potentially interact with other drugs/medicines. Eighteen species are referred as “not for use during pregnancy”, and 3 “not for use while nursing”. These results are a source of concern since in Brazil, as it is worldwide, there is the notion that plants can never be harmful. The results for the Sørensen Coefficient showed greater similarity between works performed in very close study areas. Other studies presented low similarity, mainly because of the difficulty in plant identification or a very specific focus in methodology.

Keywords: Brazil; Medicinal plants; Open-air markets; Rio de Janeiro; Toxic plants; Urban Ethnobotany

Lynne Boddy,

Chapter 8 - Pathogens of Autotrophs,

Editor(s): Sarah C. Watkinson, Lynne Boddy, Nicholas P. Money,

The Fungi (Third Edition),

Academic Press,

2016,

Pages 245-292,

ISBN 9780123820341,

https://doi.org/10.1016/B978-0-12-382034-1.00008-6.

(https://www.sciencedirect.com/science/article/pii/B9780123820341000086)

Abstract: There is a wide variety of plant diseases caused by fungi and fungus-like organisms. Plants have different constitutive and induced defence mechanisms, and differ in their susceptibility to different pathogens. Likewise, pathogens have different mechanisms of attack and responses to plant defence. Whether fungi have the ability to establish themselves within living plant cells is determined at the genetic level. The disease cycle of pathogens varies with modes of arrival, attachment and entry, the establishment in and exploitation of the plant, different ways of exiting from the plant, and surviving until the pathogen finds another suitable host. This is illustrated with case studies, mainly of diseases of crop plants. Plants in natural environments also suffer from fungal diseases, as do other autotrophs such as lichens and seaweeds. Diseases are not static and new ones are emerging with potential threats to the security of our food supply

Keywords: Biotrophic pathogens; Effectors; Elictors; Emerging diseases; Hemibiotrophic pathogens; Host resistance; Mildew; Necrotrophic pathogens; Rusts; Smuts

R.E. Uncini Manganelli, F. Camangi, P.E. Tomei,

Curing animals with plants: traditional usage in Tuscany (Italy),

Journal of Ethnopharmacology,

Volume 78, Issues 2–3,

2001,

Pages 171-191,

ISSN 0378-8741,

https://doi.org/10.1016/S0378-8741(01)00341-5.

(https://www.sciencedirect.com/science/article/pii/S0378874101003415)

Abstract: Tuscany is an area rich in traditions, many of an ethnobotanical nature, and those of veterinary practice are of special interest. Almost a 100 different plant species are used to treat animals; sometimes old remedies are used to cure similar human ailments, other times the cure is used exclusively for veterinary treatment.

Keywords: Ethnobotany; Tuscany; Italy

Hafiz Haider Ali, Arslan Masood Peerzada, Zarka Hanif, Saima Hashim, Bhagirath Singh Chauhan,

Weed management using crop competition in Pakistan: A review,

Crop Protection,

Volume 95,

2017,

Pages 22-30,

ISSN 0261-2194,

https://doi.org/10.1016/j.cropro.2016.07.009.

(https://www.sciencedirect.com/science/article/pii/S026121941630165X)

Abstract: Agriculture occupies an important place in improving the living standards of farmers in Pakistan. About 90% of farm earnings rely on the cultivation of sugar, fibre, cereals and legumes. Due to lack of essential resources and technical expertise, every year thousands of farmers fail to reach maximum yield potential. Over 70% of farmers own less than 5 ha in Pakistan; therefore, it is uneconomic to employ costly mechanical and chemical strategies for the control of pests in their crops. Among these pests, we eds are considered to be the major obstacle to crop production, and can ultimately result in crop failure. Traditionally, manipulation of cropping techniques was employed for the control of weeds; later on, development of synthetic chemical herbicides made it easier to control weeds in a very short time period. However, over time the increased use of herbicides has led to the development of herbicide resistant weeds. Furthermore, increasing environmental concerns, weed population shifts, and increased managerial costs have made it difficult for farmers to control these weed species within their limited economic resources. Nowadays, scientists and research organizations are being urged to provide innovative weed management solutions, with minimal ecological impacts. Studies have revealed the importance of cultural strategies for the management of weeds in different cropping systems. Research has proved that alternation of cultural practices, and selection of competitive crop cultivars, could be a possible strategy to minimize the competitiveness of weeds. Increased crop densities, narrower row spacing, intercropping and alternation in row directions are among the weed control strategies gaining rapid attention in many countries. Unfortunately, limited information is available about weed management using crop competition in Pakistan. This review article focusses on the importance of these agronomic practices in reducing the competitive potential of weeds, for their effective and appropriate management in major crops of Pakistan. It is intended to assist researchers in the design of economically viable and eco-friendly weed management strategies, which will aid in eliminating the burden of herbicides and mechanical cultivation from farmer’s production costs.

Keywords: Row spacing; Seeding rate; Competitive cultivars; Rice; Wheat; Cotton; Sugarcane; Weeds

O Said, K Khalil, S Fulder, H Azaizeh,

Ethnopharmacological survey of medicinal herbs in Israel, the Golan Heights and the West Bank region,

Journal of Ethnopharmacology,

Volume 83, Issue 3,

2002,

Pages 251-265,

ISSN 0378-8741,

https://doi.org/10.1016/S0378-8741(02)00253-2.

(https://www.sciencedirect.com/science/article/pii/S0378874102002532)

Abstract: An extensive ethnopharmacological survey was conducted among the most well known Arabic indigenous herbal practitioners in Israel, the Golan Heights and the West Bank in order to evaluate the potential of local plants used in treating different diseases and illnesses. Thirty-one indigenous practitioners' of Arabic traditional medicine ranging in age from 40 to 116 years, were interviewed using a previously prepared questionnaire. The current survey revealed that 129 plant species are still in use in Arabic traditional medicine for the treatments of various diseases. Among these plants, there are 40 species used for treating skin diseases, 27 species for treating kidney and urinary system, 26 species for treating diabetes, 23 species for treating digestive system including stomach and intestinal pain and inflammation, 22 species for treating liver diseases, 16 species for treating respiratory system and coughing, 13 species for treating forms of cancer and nine species for treating weight loss and cholestrol reduction. Additional findings and implications of this current survey including preparation methods and route of use are discussed in this report.

Keywords: Medicinal plants; Arabic medicine; Herbs; Diabetes; Liver diseases; Skin diseases

Marc Alexandre Tareau, Marianne Palisse, Guillaume Odonne,

As vivid as a weed… Medicinal and cosmetic plant uses amongst the urban youth in French Guiana,

Journal of Ethnopharmacology,

Volume 203,

2017,

Pages 200-213,

ISSN 0378-8741,

https://doi.org/10.1016/j.jep.2017.03.031.

(https://www.sciencedirect.com/science/article/pii/S0378874116315781)

Abstract: Ethnopharmacological relevance

French Guiana is a French overseas territory with a rich history of migration that has led to a highly intercultural society. Today, its population is one of the youngest in the French territory and is rapidly increasing. Despite a context of cultural revival seeking “tradition”, a distanced baseline of local practices is still lacking. This work addresses some aspects of the cultural hybridizations in progress in urban areas.

Methods

Semi directed interviews were conducted with willing participants aged between 18 and 40. Interviews took place in French Guiana's two main urban centres: Cayenne and Saint Laurent du Maroni. People were interviewed about the last medicinal plant they used in the preceding year. Due to the high use of plant baths in French Guiana, a focus was made on baths.

Results and discussion

Eighty-three people answered: 43 women and 40 men (mean age of 28.7 years old). In total, 226 remedies were counted in our study, 155 single plant remedies and 71 compound remedies leading to 316 use reports of plants from 16 cultural groups. A surprising number of 108 botanical species were recorded. Eighty-one recipes for baths were also collected. Despite this high citation rate, a rather low proportion of people declare a systematic and regular recourse upon local pharmacopoeia (46%; 38/83). Although many interviewees used plants, far from the majority used them on a regular basis. In practice, 50% of the species (54/108 spp.; 99/316 URs) are non-native but domesticated exotic species, imported from Asia, Europe, Africa or remote parts of America, either during colonization, the slave trade era, or more recently with the latest migrations.

Conclusion

Although phytotherapy use is often thought to be related to countryside dwellers and older people, medicinal plants seem to play an important role in the lives of urban French Guianese youth. Research shows a large diversity of medicinal species used linked with the great cultural diversity of the Guianese cities. One characteristic of this population is the hybridization process leading to a perpetual renewal of practices, both in terms of species and practice.

Keywords: Guiana shield; Urban ethnobotany; Cultural hybridization; Creole; Maroon; bita; Medicinal baths; Migration

Thomas Vanhercke, John M. Dyer, Robert T. Mullen, Aruna Kilaru, Md. Mahbubur Rahman, James R. Petrie, Allan G. Green, Olga Yurchenko, Surinder P. Singh,

Metabolic engineering for enhanced oil in biomass,

Progress in Lipid Research,

Volume 74,

2019,

Pages 103-129,

ISSN 0163-7827,

https://doi.org/10.1016/j.plipres.2019.02.002.

(https://www.sciencedirect.com/science/article/pii/S0163782718300614)

Abstract: The world is hungry for energy. Plant oils in the form of triacylglycerol (TAG) are one of the most reduced storage forms of carbon found in nature and hence represent an excellent source of energy. The myriad of applications for plant oils range across foods, feeds, biofuels, and chemical feedstocks as a unique substitute for petroleum derivatives. Traditionally, plant oils are sourced either from oilseeds or tissues surrounding the seed (mesocarp). Most vegetative tissues, such as leaves and stems, however, accumulate relatively low levels of TAG. Since non-seed tissues constitute the majority of the plant biomass, metabolic engineering to improve their low-intrinsic TAG-biosynthetic capacity has recently attracted significant attention as a novel, sustainable and potentially high-yielding oil production platform. While initial attempts predominantly targeted single genes, recent combinatorial metabolic engineering strategies have focused on the simultaneous optimization of oil synthesis, packaging and degradation pathways (i.e., ‘push, pull, package and protect’). This holistic approach has resulted in dramatic, seed-like TAG levels in vegetative tissues. With the first proof of concept hurdle addressed, new challenges and opportunities emerge, including engineering fatty acid profile, translation into agronomic crops, extraction, and downstream processing to deliver accessible and sustainable bioenergy.

Keywords: Metabolic engineering; Biomass; Oil; Plant lipid metabolism

Hannington Gumisiriza, Grace Birungi, Eunice Apio Olet, Crispin Duncan Sesaazi,

Medicinal plant species used by local communities around Queen Elizabeth National Park, Maramagambo Central Forest Reserve and Ihimbo Central Forest Reserve, South western Uganda,

Journal of Ethnopharmacology,

Volume 239,

2019,

111926,

ISSN 0378-8741,

https://doi.org/10.1016/j.jep.2019.111926.

(https://www.sciencedirect.com/science/article/pii/S0378874119304982)

Abstract: Ethnopharmacological relevance

The application of ethnobotanical indigenous knowledge is very important in improving primary healthcare systems among the local communities living around and within protected areas in South Western Uganda. In this area, there are biodiversity endowed Queen Elizabeth National Park (QENP), Maramagambo Central Forest Reserve (MCFR) and Ihimbo Central Forest Reserve (ICFR). Despite the rich floral diversity and cultural heritage, there is no published documentation on the use of medicinal plants in this area. This information can be used as a basis for the selection of medicinal plants for further phytochemical and pharmacological studies.

Study aim

This study identified and documented the use of medicinal plants, plant parts used, and mode of preparation and administration by the local communities living around and within QENP, MCFR and ICFR.

Materials and methods

A cross-sectional study was used to collect data from 202 informants using semi-structured questionnaires, open interviews and field visits. Ethnobotanical data was analyzed using use reports (UR), frequency of citation (FC) and Informant Consensus Factor (FIC). The plants species were identified by botanists and voucher specimens were deposited.

Results

A total of 302 medicinal plant species were mentioned by informants, out of which only 211 species belonging to 65 families and 165 genera were collected, identified and documented. The remaining 91 species were not available for collection and informants stated that they had become very rare within the study area. Herbs (35.8%) were the main source of herbal medicine. Leaves (60.4%) were the most commonly used plant parts used in the preparation of herbal remedies. Most of the medicinal plants were harvested from the wild, either growing in abundance (41%) or as rare species (21%). The most common mode of administration was oral, while other exceptional modes such as touching with bare hands and sweeping over the affected part were reported for the first time. The medicinal plant species were reported to treat 134 physical ailments, which were grouped into 16 ICPC-2 disease categories. Digestive disorders (854 UR) and general and unspecified disorders (507) scored the highest FIC value of 0.83. The highest number of medicinal plants (146 plant species) was used for treatment of digestive disorders. Among the species with higher use reports, Gouania longispicata had the highest frequency of citation (FC = 174) and was mentioned to be used to treat 41 physical ailments. The most important ailment treated by Gouania longispicata was allergy with 102 use reports.

Conclusions

A variety of medicinal plants are used by communities living near protected areas in South Western Uganda. Most species were used in the treatment of digestive disorders, followed by general and unspecified disorders. Much as allergy has not been identified as a major threat by the health sector in Uganda, the study found out that it is one of the prevalent ailments in the study area. While the therapeutic value of some of the documented medicinal plant species, especially those with higher frequency of citation have been scientifically validated, the efficacy and safety of other species with wide application need to be investigated. In this study, we recommend further scientific studies on Gouania longispicata to validate its wide usage in the study area.

Keywords: Medicinal plants; Indigenous knowledge; Queen elizabeth national park; Maramagambo central forest reserve; Ihimbo central forest reserve

A.K. Welcome, B.-E. Van Wyk,

An inventory and analysis of the food plants of southern Africa,

South African Journal of Botany,

Volume 122,

2019,

Pages 136-179,

ISSN 0254-6299,

https://doi.org/10.1016/j.sajb.2018.11.003.

(https://www.sciencedirect.com/science/article/pii/S0254629918317046)

Abstract: The food plants of southern Africa have not yet been been systematically recorded and the main patterns of plant use have therefore never been studied. The book by Fox and Norwood Young (1982) entitled “Food from the Veld” has been the most comprehensive to date and has become the standard reference work on the subject. However, this publication has become outdated and it is evident that many species were not included. We present here, for the first time, a comprehensive inventory and checklist of the edible plants of southern Africa, i.e., the Flora of Southern Africa (FSA) region. Seventy-four literature sources, including books, journal articles, checklists, grey literature and published ethnobotanical surveys were used to compile the inventory, which includes 1740 species (more than double the number listed by Fox and Norwood Young). All edible plants were marked-off in an Excel spreadsheet of all southern African plant species, made available by the South African National Biodiversity Institute (SANBI). Using the Excel format, it was possible to easily explore the main patterns relating to the frequency of citation of the species, the number of species at different taxonomic ranks (families, genera and species), the plant parts that are used and the use categories within the region. For a selection of 13 indigenous cultural/language groups (for which adequate information was available), we quantified the local preferences (for the most species-rich families, the most commonly used plant parts and the most frequent categories of use. The food plant data for southern Africa were also compared to sub-Saharan Africa and the entire world. Surprisingly, it was found that the Apocynaceae was the most species-rich family of southern Africa food plants, with a total of 137 species, followed by the Fabaceae (135 species), Asteraceae (94 species) and Poaceae (73 species). A similar pattern was found in sub-Saharan Africa, but with Fabaceae in the first position, followed by Apocynaceae. The family-level pattern for the entire world is different, where the Apocynaceae is unimportant, being replaced by Rosaceae (the last-mentioned is of very low significance in Africa). The most species-rich food plant genus in southern Africa is Searsia, while Solanum nigrum and Sclerocarya birrea have the highest frequency of citation in the literature. The most popular plant part used as a food in southern Africa, as well as in the entire world, are fruits, followed by leaves and then underground storage organs. The most species-rich category of food plant use is those consumed raw (and mostly in situ) as snacks, followed by those which are cooked as vegetables. The Apocynaceae have mostly edible underground storage organs as the edible parts, while Fabaceae is more diverse. Fruits and leaves have been determined as the most important plant parts used as food for most of the cultural groups except for the Khoekhoe group, where underground parts are the most important. For all of the cultural groups, plants used for snacks were the most important. A chronological list of publications shows that there were seven major contributions since the first survey by Van der Stel in 1685. The comprehensive inventory provides profound new insights into foraging and human food ecology and may also have value in interpreting and reconstructing the availability of food plants during the evolution of early humans in southern Africa.

Keywords: Flora of southern African region; Edible plants; Checklist; Apocynaceae; Fabaceae; Fruits; Vegetables; Language groups

Juan Hernández Cano, Gabriele Volpato,

Herbal mixtures in the traditional medicine of Eastern Cuba,

Journal of Ethnopharmacology,

Volume 90, Issues 2–3,

2004,

Pages 293-316,

ISSN 0378-8741,

https://doi.org/10.1016/j.jep.2003.10.012.

(https://www.sciencedirect.com/science/article/pii/S0378874103003982)

Abstract: Herbal mixtures in the traditional medicine of Eastern Cuba. Traditional herbal mixtures in Eastern Cuba are investigated through interviews with 130 knowledgeable people and traditional healers of the provinces of Santiago de Cuba and Guantánamo. One hundred seventy plant species and other products are used in 199 formulas, galones being the more complex. Cocos nucifera L. (Arecaceae), Bidens pilosa L. (Asteraceae), Cissus sicyoides L. (Vitaceae), Erythroxylum havanense Jacq. (Erythroxylaceae) and Stachytarpheta jamaicensis (L.) Vahl. (Verbenaceae) are the species most frequently cited. The ecological distribution of the taxa and cultural and anthropological aspects of mixtures are highlighted; particularly American and African influences that have shaped local knowledge about plant combinations are discussed.

Keywords: Ethnobotany; Phytomedicine; Herbal mixtures; Eastern Cuba; Galones

Yelda Güzel, Mehmet Güzelşemme, Mahmut Miski,

Ethnobotany of medicinal plants used in Antakya: A multicultural district in Hatay Province of Turkey,

Journal of Ethnopharmacology,

Volume 174,

2015,

Pages 118-152,

ISSN 0378-8741,

https://doi.org/10.1016/j.jep.2015.07.042.

(https://www.sciencedirect.com/science/article/pii/S0378874115300593)

Abstract: Ethnopharmacological relevance

We have compiled information about the medicinal plants used in folk medicine in the district of Antakya. Since its establishment by King Seleucus I in 300 B.C., Antakya (old Antioch) has hosted nearly 20 civilizations. Antakya, neighboring Northwestern Syria, is located on the western end of the “Silk Road” and was one of the great centers of Graeco–Roman world. Today, Antakya is a cosmopolitan city in which Arabic and Turkish are widely spoken, and where distinct ethnic and religious communities, such as Arab Alawite, Arab Christian, Arab Sunni, Turk Sunni, Armenian, and Jewish, have been living together in harmony for centuries. In addition, the rich flora in the vicinity of Antakya also renders the area interesting in terms of ethnobotanical fieldwork.

Aim of the study

This study aimed to compile the information about plants used for medicinal purposes by local people in the district of Antakya. This city is a significant region in terms of ethnobotanical fieldwork, owing to its cosmopolitan structure, long history, relatively preserved traditional community structure, and rich flora. Furthermore, we sought to compare the ethnomedicinal data geographically, cross-culturally, and historically.

Materials and methods

The ethnobotany of medicinal plants used in the district of Antakya has been investigated through two separate studies; one was conducted in 1975, interviewing 29 people, and the other was conducted in 2011–2013, interviewing 182 people. The use value (UV) and informant consensus factor (FIC) values of the plants were calculated. In order to interpret the authenticity and sources of the compiled ethnomedicinal information, previous publications that contain information about the similar medicinal uses of plants identified in our region were reviewed and evaluated meticulously. A comparison with the data obtained from other regions of Turkey and from other Mediterranean regions, as well as a cross-cultural analysis between the ethnic groups within the study area, was performed by implementation of the Jaccard index (JI)

Results

Throughout the study, information about 202 medicinal plant taxa was compiled. Among these plants, 39 have either not yet been mentioned in ethnobotanical or medicinal studies, or have been used for a medicinal purpose other than those encountered in the literature review. The ethnomedicinal information we gathered from the study area exhibits close similarities to the ethnomedicinal information of other Southern and Eastern Mediterranean countries where Arabic is spoken, as well as to that of Northern and Western Mediterranean countries where Latin languages are spoken. In addition to these similarities, in most cases, this ethnomedicinal information shows hybrid features of ethnomedicinal knowledge from Eastern and Western Mediterranean countries.

Conclusion

Based on a literature survey, we found that the cited medicinal uses for 43 plants have also been corroborated by other various biological testings. This finding strongly suggests the importance of ethnobotanical studies in the development of new medicines. We believe that this study has compiled rich ethnomedicinal information that reflects the cosmopolitan structure of Antakya in a very good way.

Keywords: Ethnomedicine; Antakya; Multicultural old city; Diachronic analysis; Cross-cultural; Comparison; Turkey

Teresa Tuttolomondo, Mario Licata, Claudio Leto, Valentina Savo, Giuseppe Bonsangue, Maria Letizia Gargano, Giuseppe Venturella, Salvatore La Bella,

Ethnobotanical investigation on wild medicinal plants in the Monti Sicani Regional Park (Sicily, Italy),

Journal of Ethnopharmacology,

Volume 153, Issue 3,

2014,

Pages 568-586,

ISSN 0378-8741,

https://doi.org/10.1016/j.jep.2014.02.032.

(https://www.sciencedirect.com/science/article/pii/S0378874114001457)

Abstract: Ethnopharmacological relevance

The area of the “Monte Sicani Regional Park” (Central Western Sicily, southern Italy) has been quantitatively and extensively investigated in an ethnobotanical study for the first time. A total of 108 wild species are used for medicinal purposes, while, according to our study, the uses of 9 species have not previously been reported in ethnobotanical studies in Italy (e.g., the use of Kickxia elatine (L.) Dumort. for the treatment of hyperhydrosis of the feet, the use of Lavatera agrigentina Tineo for cough and bronchitis). Aim of the study: The aim of this paper is to analyze, through quantitative indicators, the extent of the current knowledge on medicinal uses of plants in the area, evaluating also the features of uniqueness and commonality of this knowledge in comparison with other Italian and Mediterranean areas.

Methodology

Semi-structured interviews were carried out in the local communities between 2009 and 2010 within the Monti Sicani Regional Park with local people retained experts in rural traditions. A total of 230 people were interviewed about their knowledge on medicinal plant uses. Local plant uses were evaluated using ethnobotanical indices (e.g., cultural importance index, ethnobotanicity index, informant consensus factor) and then compared with uses in other localities in Sicily, Italy and the Mediterranean basin.

Results

Local communities currently use a total number of 108 wild species (43 families) as remedies for human and livestock ailments. The majority of plants are used in the treatment of articular, skin and gastrointestinal problems. The use of some species is limited to Sicily (e.g., Rhus coriaria L., Athamanta sicula L., Senecio delphinifolius Vahl).

Conclusions

The research shows an ongoing process of cultural erosion in an advanced stage, but results still highlight an interesting cultural identity as regards the local folk medicine.

Keywords: Mediterranean folk medicine; Kickxia elatine (L.) Dumort; Lavatera agrigentina Tineo; Cultural erosion; Ethnobotanical indexes

Jayachandra Reddy Nakkala, Rani Mata, Arvind Kumar Gupta, Sudha Rani Sadras,

Biological activities of green silver nanoparticles synthesized with Acorous calamus rhizome extract,

European Journal of Medicinal Chemistry,

Volume 85,

2014,

Pages 784-794,

ISSN 0223-5234,

https://doi.org/10.1016/j.ejmech.2014.08.024.

(https://www.sciencedirect.com/science/article/pii/S022352341400751X)

Abstract: Nanomedicine utilize biocompatible nanomaterials for diagnostic and therapeutic purposes. This study reports the synthesis of silver nanoparticles using aqueous rhizome extract of Acorus calamus (ACRE) and evaluation of antioxidant, antibacterial as well as anticancer effects of synthesized A. calamus silver nanoparticles (ACAgNPs). The formation of ACAgNPs was confirmed by UV–visible spectroscopy and their average size was found to be 31.83 nm by DLS particle size analyzer. Scanning electron micrograph (SEM) revealed spherical shape of ACAgNPs and energy dispersive spectroscopy (EDX) data showed the presence of metallic silver. Fourier transform infrared spectroscopy (FTIR) analysis indicated the presence of phenol/alcohol, aromatic amine and carbonyl groups in ACRE that were involved in reduction and capping of nanoparticles. ACRE and ACAgNPs exhibited substantial free radical quenching ability in various in vitro antioxidant assays performed in this study. ACAgNPs also displayed appreciable antibacterial activity against three different pathogenic bacteria and the growth kinetic study with Escherichia coli designated the inhibition of bacterial growth at the log phase. The cytotoxic effect of ACAgNPs was assessed by MTT assay in HeLa and A549 cells. The IC50 value of ACAgNPs respectively after 24 and 48 h was found to be 92.48 and 69.44 μg/ml in HeLa cells and in A549 cells it was 53.2 and 32.1 μg/ml. Apoptotic cell death in ACAgNPs treated cells was indicated by acridine orange/ethidium bromide (AO/EB) and annexinV-Cy3 staining techniques. Staining with propidium iodide (PI) and 4′, 6-diamidino-2-phenylindole, dihydrochloride (DAPI) also confirmed nuclear changes such as condensation and fragmentation. Further, terminal deoxynucleotidyl transferase dUTP nick end labeling (TUNEL) assay showed distribution of ACAgNPs treated cells in the late apoptotic stage. These findings emphasize that such biocompatible green nanoparticles with multifaceted biological activities may find their applications in the field of nanomedicine.

Keywords: Acorus calamus rhizome; Silver nanoparticles; Antibacterial; Cytotoxic effect; DNA fragmentation; Apoptosis

Gideon M. Polya,

Protein and Non-Protein Protease Inhibitors from Plants,

Editor(s): Atta-ur-Rahman,

Studies in Natural Products Chemistry,

Elsevier,

Volume 29, Part J,

2003,

Pages 567-641,

ISSN 1572-5995,

ISBN 9780444515100,

https://doi.org/10.1016/S1572-5995(03)80015-7.

(https://www.sciencedirect.com/science/article/pii/S1572599503800157)

Abstract: ABSTRACT:

Plants are consumed by bacteria, fungi and animals and utilization of plant proteins requires their hydrolysis, a process which is catalyzed by proteases. Plants defend themselves against other organisms by elaborating physical and chemical defenses, the latter including protein and non-protein inhibitors of proteases. Plant protease inhibitor proteins can be either constitutive or inducible as a result of wounding or pathogen invasion. Proteases are classified into the aspartic proteases, cysteine proteases, metalloproteases and serine proteases on the basis of the involvement of aspartate, cysteine, metal ions and serine, respectively, in the catalytic mechanism. Extracellular proteases are involved in digestion, blood clotting, inflammatory responses to invasion, and extracellular matrix digestion required for angiogenesis and tissue remodelling. Intracellular proteolysis must be exquisitely regulated to avoid autolysis and intracellular proteases are involved in proprotein processing, lysosome- and proteasome-mediated protein destruction, cell division and apoptosis. A large variety of plant protease inhibitor proteins and peptides have been resolved including aspartic protease inhibitor proteins, cysteine protease inhibitory phytocystatins, metallocarboxypeptidase inhibitor proteins and serine protease inhibitor proteins such as the Bowman-Birk, cereal bifunctional, Kunitz, potato type I, potato type II, mustard family, squash family, serpin and other protease inhibitors. Plant protease inhibitor proteins have potential transgenic applications for crop plant defense. A variety of non-protein protease inhibitors have also been resolved from plants. Plant protease inhibitors have potential for pharmaceutical development especially in relation to Alzheimer’s disease, angiogenesis, cancer, inflammatory disease and viral and protozoal infection.

Index,

Editor(s): Arlen W. Frank,

Chemistry of Plant Phosphorus Compounds,

Elsevier,

2013,

Pages 623-669,

ISBN 9780124071940,

https://doi.org/10.1016/B978-0-12-407194-0.09989-3.

(https://www.sciencedirect.com/science/article/pii/B9780124071940099893)

Index,

Editor(s): Alexandru Mihai Grumezescu,

Inorganic Frameworks as Smart Nanomedicines,

William Andrew Publishing,

2018,

Pages 681-699,

ISBN 9780128136614,

https://doi.org/10.1016/B978-0-12-813661-4.00031-6.

(https://www.sciencedirect.com/science/article/pii/B9780128136614000316)

Index,

Editor(s): Parvaiz Ahmad, Saiema Rasool,

Emerging Technologies and Management of Crop Stress Tolerance,

Academic Press,

2014,

Pages 537-551,

ISBN 9780128008768,

https://doi.org/10.1016/B978-0-12-800876-8.00035-7.

(https://www.sciencedirect.com/science/article/pii/B9780128008768000357)

K. Magwede, B.-E. van Wyk, A.E. van Wyk,

An inventory of Vhavenḓa useful plants,

South African Journal of Botany,

Volume 122,

2019,

Pages 57-89,

ISSN 0254-6299,

https://doi.org/10.1016/j.sajb.2017.12.013.

(https://www.sciencedirect.com/science/article/pii/S0254629917314345)

Abstract: An inventory and analysis of the general uses of plants by the Vhavenḓa, a cultural group who historically occupied the region known as Venḓa, currently referred to as the Vhembe District, Limpopo Province, South Africa, are presented. Information on plant uses was gathered through a literature review and interviews conducted amongst Tshivenḓa-speaking rural communities in the Vhembe District. The aim of the study was to document all Vhavenḓa useful plants, i.e., all plants of cultural and practical importance in fulfilling the everyday needs of the people. A total of 574 plant species from 355 genera and 121 families was recorded. In addition 897 vernacular names have been recorded, of which 224 (25%) is published here for the first time. The list includes 189 trees, 143 shrubs, 170 herbs, 44 climbers, 21 grasses, four sedges, one parasite and two epiphytes. The number of species in the main use categories are as follow: medicine (384), vegetables (128), edible fruits (123), firewood (101), craftwork (93), construction (91), magic (45), beverages (34), cordage (31), ornamental (26), shade (22), fodder (16), birdlime or rubber (14), famine foods (11), snuff ingredient (11), edible caterpillars feeding on leaves (10), dyes and leather tanning (eight), live fence (seven), vegetable condiments (six) and fish poisons (six). Minor uses include edible seeds, edible cooked tubers, edible flower nectar, ritual purposes, edible gum, edible root tubers, edible rhizomes, edible gums, musical flutes, edible nuts, edible corn, edible leaf sap, soda for cooking vegetables, coffee substitutes, toothbrushes, polish, cosmetic oil, arrow poisons, insects repellents, soap substitutes, adhesives, toys and others. The top ten plant families i.e., those contributing the highest number of useful plant species are Fabaceae/Leguminosae (64 spp.), Asteraceae (33 spp.), Malvaceae s.l. (32 spp.), Apocynaceae s.l. (24 spp.), Euphorbiaceae (22 spp.), Poaceae (21 spp.), Rubiaceae (20 spp.), Solanaceae (20 spp.), Amaranthaceae (19 spp.) and Cucurbitaceae (18 spp.). The data is not only a contribution to the cultural heritage of the Vhavenḓa, but also of considerable scientific and practical interest. It provides the basic information that researchers can use to compare historical and contemporary biocultural plant-use patterns in southern Africa, as well as in controlled future studies to test the efficacy/merits of specific uses.

Keywords: Venda; Vhavenḓa ethnobotany; Checklist; Useful plants; Traditional plant use; Vhembe District; Soutpansberg; South Africa; Tshivenḓa

Saravanan V. Sathasivampillai, Pholtan R.S. Rajamanoharan, Michael Munday, Michael Heinrich,

Plants used to treat diabetes in Sri Lankan Siddha Medicine – An ethnopharmacological review of historical and modern sources,

Journal of Ethnopharmacology,

Volume 198,

2017,

Pages 531-599,

ISSN 0378-8741,

https://doi.org/10.1016/j.jep.2016.07.053.

(https://www.sciencedirect.com/science/article/pii/S0378874116304779)

Abstract: Introduction and background

In recent decades diabetes mellitus has become a considerable health problem in countries like Sri Lanka and results in an increasing economic burden hampering the social and economic development of these countries. About 60% to 70% of the rural population in Sri Lanka rely on indigenous medicinal systems as their main source for primary health care. Siddha (Tamil) Medicine is one of the four Sri Lankan traditional medicinal systems and it is practised mostly in the eastern and northern provinces of Sri Lanka where the majority of Tamils reside.

Aim

The foundation of this study is a documentation of plant species recorded in historical and modern Sri Lankan Siddha Medical documents used to treat diabetes. Based on the systematic documentation and analysis of Siddha concepts about diabetes and its signs and preparations used to treat diabetes in Sri Lankan Siddha Medicine, the plant species included in these preparations (excluding globally or very widely used, very well studied species) were evaluated in terms of the current state-of-the-art about these species' pharmacology and effectiveness in order to lay a foundation for their further development.

Method

Historic and modern Sri Lankan university texts books in Tamil were used as sources for information on diabetes Siddha concepts and antidiabetic Sri Lankan Siddha Medicine preparations. Information on the known antidiabetic effects of extracts and compounds obtained from these species were used in order to assess the current state of the art of these species.

Results and discussion

Information of ingredients, preparation methods, amount of ingredients used, and dosages of 60 antidiabetic Sri Lankan Siddha Medicine preparations were obtained. Animal parts including marine organisms, inorganic substances, and plants are the three types of ingredients used. Overall 171 plant species in 73 families were documented. Senna auriculata (L.) Roxb. (Fabaceae) was identified as the most frequently cited species. Globally distributed and very well studied plants were excluded in the pharmacological and clinical literature review which includes 123 plant species. The majority (48%) of the plant species reviewed were studied up to in vivo level as the current maximum level of scientific evidence available. Followed by 41% of species have not been studied for antidiabetic activities or did not show antidiabetic activity. Moreover, 6% and 5% were studied up to in vitro and in clinical levels, respectively. The majority of the species were studied only in the models that represent type 1 diabetes.

Conclusion

This is the first study systematically assessing the importance of preparations and plants used in antidiabetic Sri Lankan Siddha Medicine preparations. Antidiabetic plants are a crucial health care resource in Sri Lankan Siddha Medicine. This study also identified a wide range of methodological problems in the studies conducted so far. More and better type 2 diabetes models should be employed in future studies. This comprehensive review creates the basis for a more systematic study of these local resources.

Keywords: Alpha glucosidase inhibition assay; alpha amylase inhibition assay; Diabetes Mellitus; Fabaceae; Senna auriculata; Siddha Medicine; Sri Lanka; Streptozotocin; Tamil Medicine

Botao Wang, Junjie Han, Wei Xu, Yuhui Chen, Hongwei Liu,

Production of bioactive cyathane diterpenes by a bird’s nest fungus Cyathus gansuensis growing on cooked rice,

Food Chemistry,

Volume 152,

2014,

Pages 169-176,

ISSN 0308-8146,

https://doi.org/10.1016/j.foodchem.2013.11.137.

(https://www.sciencedirect.com/science/article/pii/S030881461301827X)

Abstract: Cyathane diterpenes are important bioactive substances produced by some edible and medicinal fungi. Seven new cyathane type diterpenes, named as cyathins J–P (1–7), together with two known diterpenes (8 and 9), were isolated from the solid culture of the bird’s nest fungus Cyathus gansuensis growing on cooked rice. The structures of the new secondary metabolites were elucidated by NMR experiments. Bioactivity screening indicated that compounds 1, 2, 4, and 8 showed moderate inhibitory activity against NO production in lipopolysaccharide-activated macrophages with an IC50 value of 42, 78, 80, and 16μM, respectively. The fungus C. gansuensis is a promising source of bioactive secondary metabolites and has application potential in preparing healthy food.

Keywords: Cyathus gansuensis; Cyathane diterpenes; Structure elucidation; NO inhibition

Behxhet Mustafa, Avni Hajdari, Bledar Pulaj, Cassandra L. Quave, Andrea Pieroni,

Medical and food ethnobotany among Albanians and Serbs living in the Shtërpcë/Štrpce area, South Kosovo,

Journal of Herbal Medicine,

Volume 22,

2020,

100344,

ISSN 2210-8033,

https://doi.org/10.1016/j.hermed.2020.100344.

(https://www.sciencedirect.com/science/article/pii/S2210803320300166)

Abstract: Ethnobotanical research in the Balkans is important for providing concrete insights aimed at developing small-scale markets of local medicinal plants and food products to support rural development. An ethnobotanical field study was carried out in the spring of 2017 among Muslim Albanians and Christian Orthodox Serbs living in 20 villages located in South Kosovo. The aim of the study was to assess if two different ethnic affiliations played a role in shaping traditions of local plant uses by ethnic groups living in the same natural environment in South Kosovo over many centuries. The field survey was conducted via semi-structured interviews with 181 local adults who were chosen for their retention of traditional ecological knowledge (TEK) regarding traditional uses of wild and cultivated food and medicinal plants and fungi relevant to either human or animal health. A total of 122 botanical and fungal folk taxa, belonging to 51 families, and 19 other domestic remedies were recorded. The most common plants species cited by the study participants belongs to family Rosaceae, followed by Lamiaceae, and Asteraceae. Approximately 10 % of the total reports have not been previously recorded in the Western Balkans. Comparison of the recorded reports between the Serbian and Albanian demonstrated that only 28.4 % of the recorded remedies are shared between the two ethnic groups, thus confirming the importance of religious and ethnic divides in shaping divergent traditional uses of natural resources. A more “herbophilic” attitude of the Slavic population (pointed out in previous studies) was not evident in this survey.

Keywords: Ethnobotany; Kosovo; Serbs; Medicinal plants; Albania

Yvan Gaillard, Gilbert Pepin,

Poisoning by plant material: review of human cases and analytical determination of main toxins by high-performance liquid chromatography–(tandem) mass spectrometry,

Journal of Chromatography B: Biomedical Sciences and Applications,

Volume 733, Issues 1–2,

1999,

Pages 181-229,

ISSN 0378-4347,

https://doi.org/10.1016/S0378-4347(99)00181-4.

(https://www.sciencedirect.com/science/article/pii/S0378434799001814)

Abstract: The authors have reviewed the main toxic plants responsible for human deaths throughout the world. Forty plants (genera or species) were listed in order to establish an inventory of the active molecules that could be identified, the already published analytical methods and the reported human fatal cases. In a second step, the authors have developed a general method for the detection of various toxins in whole blood by high-performance liquid chromatography coupled to mass spectrometry or tandem mass spectrometry. Sample preparation was realized by liquid–liquid extraction at pH 9.5 for oleandrine, taxol and the alkaloids. These latter compounds were divided into two groups following their chemical properties and could be subsequently purified by acid/base clean up. Cyanogenic compounds and atractyloside were isolated by precipitation of the protein content with acetone and purified for atractyloside by washing with chloroform. Separation of the drugs occurred under reversed-phase conditions on a C18 analytical column 150×2 mm I.D. (5 μm particle size) using two different mobile phases. The first one, formiate buffer 2 mM acidified at pH 3.0, was used for the separation of atractyloside, oleandrine, taxol, the cyanogenic molecules and some alkaloids. The second mobile phase, formiate buffer 10 mM made basic at pH 8.2 was used for the majority of other alkaloids. A gradient elution mode was chosen using acetonitrile or acetonitrile–methanol (50:50, v/v) as the eluting solvent. Detection under positive ionization mode was the mode of choice for all compounds except for atractyloside (negative ions) and for taxol (mixed mode available). Application to real forensic cases has been demonstrated.

Keywords: Oleandrine; Taxol; Alkaloids; Atractyloside; Cyanogenic compounds

Y. Marin-Felix, M. Hernández-Restrepo, I. Iturrieta-González, D. García, J. Gené, J.Z. Groenewald, L. Cai, Q. Chen, W. Quaedvlieg, R.K. Schumacher, P.W.J. Taylor, C. Ambers, G. Bonthond, J. Edwards, S.A. Krueger-Hadfield, J.J. Luangsa-ard, L. Morton, A. Moslemi, M. Sandoval-Denis, Y.P. Tan, R. Thangavel, N. Vaghefi, R. Cheewangkoon, P.W. Crous,

Genera of phytopathogenic fungi: GOPHY 3,

Studies in Mycology,

Volume 94,

2019,

Pages 1-124,

ISSN 0166-0616,

https://doi.org/10.1016/j.simyco.2019.05.001.

(https://www.sciencedirect.com/science/article/pii/S0166061619300089)

Abstract: This paper represents the third contribution in the Genera of Phytopathogenic Fungi (GOPHY) series. The series provides morphological descriptions, information about the pathology, distribution, hosts and disease symptoms for the treated genera, as well as primary and secondary DNA barcodes for the currently accepted species included in these. This third paper in the GOPHY series treats 21 genera of phytopathogenic fungi and their relatives including: Allophoma, Alternaria, Brunneosphaerella, Elsinoe, Exserohilum, Neosetophoma, Neostagonospora, Nothophoma, Parastagonospora, Phaeosphaeriopsis, Pleiocarpon, Pyrenophora, Ramichloridium, Seifertia, Seiridium, Septoriella, Setophoma, Stagonosporopsis, Stemphylium, Tubakia and Zasmidium. This study includes three new genera, 42 new species, 23 new combinations, four new names, and three typifications of older names.

Keywords: DNA barcodes; Fungal systematics; New taxa

Reginaldo Vicente Ribeiro, Isanete Geraldini Costa Bieski, Sikiru Olaitan Balogun, Domingos Tabajara de Oliveira Martins,

Ethnobotanical study of medicinal plants used by Ribeirinhos in the North Araguaia microregion, Mato Grosso, Brazil,

Journal of Ethnopharmacology,

Volume 205,

2017,

Pages 69-102,

ISSN 0378-8741,

https://doi.org/10.1016/j.jep.2017.04.023.

(https://www.sciencedirect.com/science/article/pii/S0378874116321973)

Abstract: Ethnopharmacological importance

Currently, in many traditional communities, such as the riverine community in the North Araguaia microregion (Mato Grosso, Brazil), plant knowledge and use represent the main, if not the only, therapeutic resource for the maintenance of health and/or treatment of diseases. This study aimed to identify and document species of medicinal plants used by local experts from riverine communities in the North Araguaia microregion in Mato Grosso State, and to further chemical and pharmacological studies on species selected based on searches in the relevant literature.

Materials and methods

This is a cross-sectional ethnobotanical study, with non-probabilistic sampling (n =60), that applied the snowball method to select local riverine experts who understand medicinal plant use. Socio-demographic, ethnobotanical and ethnopharmacological data (vernacular name, uses, geographical origin, habit, method of preparation and part used) on medicinal plants were collected during semi-structured interviews. The results were analyzed by descriptive and quantitative means: indices of use-report (UR) were used to select plant species with therapeutic potential.

Results

In total, 309 plant species belonging to 86 botanical families were cited; 73% were native to Brazil, and Fabaceae was the most representative family (11.3%). Arboreal was the predominant life form (37.2%). The leaf was the most used part (28.9%). Infusion was the most commonly reported method of preparation (31.3%). The plants reported in the survey were indicated for 18 of the 22 ICD-10 disease categories. The disease categories most commonly cited were the infectious and parasitic diseases (IPD, 718 UR), digestive system diseases (DSD, 565 UR) and respiratory system diseases (RSD, 504 UR), representing 16.6%, 13.1% and 11.7%, respectively of the total UR. Dysphania ambrosioides L. was the most sighted in the IPD category 50 UR. Copaifera langsdorffii Desf. (133), Lafoensia pacari A. St.-Hil. (131), and Cecropia pachystachya Trécul (126) were the species with the highest UR. Bidens pilosa L., Vernonia ferruginea Less, and L. pacari, respectively, were the most cited native plants used to treat such diseases. Of the 8 investigated native plants, C. langsdorffii, and Brosimum gaudichaudii are the most prominent: in addition to having been widely studied, in terms of phytochemical and pharmacological, these species have been marketed as pharmaceutical products, with associated patent deposits.

Conclusions

Local riverine experts from the North Araguaia microregion use a wide variety of medicinal plants in self-care health, especially those species used to treat IPD. The therapeutic potential of some of these plants has been scientifically validated; however, there are other species whose pharmacological effects and safety remain to be properly investigated. Thus, the present study, aside from being a basis for future chemical, pharmacological and agronomic bioprospecting studies, may contribute to the development of the management, conservation and sustainable use of medicinal flora in the microregion studied.

Keywords: Ethnobotany; Snowball-sampling; Riverine Population; Medicinal Plants; North Araguaia microregion

Isanete Geraldini Costa Bieski, Marco Leonti, John Thor Arnason, Jonathan Ferrier, Michel Rapinski, Ivana Maria Povoa Violante, Sikiru Olaitan Balogun, João Filipe Costa Alves Pereira, Rita de Cassia Feguri Figueiredo, Célia Regina Araújo Soares Lopes, Dennis Rodrigues da Silva, Aloir Pacini, Ulysses Paulino Albuquerque, Domingos Tabajara de Oliveira Martins,

Ethnobotanical study of medicinal plants by population of Valley of Juruena Region, Legal Amazon, Mato Grosso, Brazil,

Journal of Ethnopharmacology,

Volume 173,

2015,

Pages 383-423,

ISSN 0378-8741,

https://doi.org/10.1016/j.jep.2015.07.025.

(https://www.sciencedirect.com/science/article/pii/S0378874115300428)

Abstract: Ethnopharmacological importance

The use of medicinal plants for treatment, cure and prevention of diseases has been described by many people since time immemorial. Because of this use, commercial and scientific interests have emerged, making it necessary to realize ethnobotanical surveys of medicinal plants species, which is important for subsequent chemical and pharmacological bioprospections.

Aim of the study

This study aimed at surveying, identifying, cataloging and documenting the medicinal plants species used in the Valley of Juruena, Northwestern Mato Grosso, Legal Amazon Brazil for the treatment of various human diseases, as well as assessed the species of interest for bioprospecting potential.

Materials and methods

Informants were interviewed using semi-structured form to capture information on socio-demographic and ethnopharmacological data of medicinal plants such as vernacular name, uses, geographic origin, habit, form of preparation and part used. Results were analyzed using descriptive and quantitative means: indices of use-report (Ur) and informant consensus factor (ICF), for the selection of plant species with therapeutic potential.

Results

Three hundred and thirty two (332) plants species belonging to 90 families were reported for medicinal purposes and totaling 3973 use-reports were reported by 365 (92.9%) of the people interviewed. Asteraceae (32.2%), Fabaceae (26.7%) and Lamiaceae (24.4%) families were the most represented, with majority being species native (64.45%) to Brazil. Leaves (64.5%) were the part of the plant most used and infusion (45.7%) was the most utilized form. Gastrointestinal disorders followed by respiratory complaints topped the list of use-reports. The native or naturalized plants with the highest use reports in the order of decreasing absolute frequency per each emic-category are Cymbopogon citratus (DC.) Stapfc (104), Mentha pulegium L. (94), Arrabidaea chica (Humb. & Bonpl.) B. Verl. (97), Alternanthera brasiliana (L.) Kuntze (71), Baccharis crispa Spreng (57), Phyllanthus niruri L. (48), Gossypium barbadense L. (44), Solidago microglossa DC. (40) and Bauhinia forficata L. (20). And the most cited exotics are: Chenopodium ambrosioides L. (151), Aloe vera (L.) Burm. f., (89) and Rosmarinus officinalis L. (72). In some cases, high ICF values were found, which reflects high degree of homogeneity of consensus among informants in this region on medicinal plants.

Conclusion

The population of Valle of Juruena makes use of a wide array of medicinal plants distributed in all use categories with predominance of those use in the treatments of gastrointestinal and respiratory ailments. The therapeutic potential of some of the species of medicinal importance extensively utilized by the population of the region have been scientifically validated, and are therefore promising prototype of new drugs. However, there are some of these species whose ethnomedicinal uses are yet to be scientifically verified and thus constitute an unexplored terrain for future biological/pharmacological studies.

Keywords: Emic use; Ethnobotanical; Amazon rain forest; Use-reports; Informant consensus factor

Bharat B. Aggarwal, Shishir Shishodia,

Molecular targets of dietary agents for prevention and therapy of cancer,

Biochemical Pharmacology,

Volume 71, Issue 10,

2006,

Pages 1397-1421,

ISSN 0006-2952,

https://doi.org/10.1016/j.bcp.2006.02.009.

(https://www.sciencedirect.com/science/article/pii/S0006295206000955)

Abstract: While fruits and vegetables are recommended for prevention of cancer and other diseases, their active ingredients (at the molecular level) and their mechanisms of action less well understood. Extensive research during the last half century has identified various molecular targets that can potentially be used not only for the prevention of cancer but also for treatment. However, lack of success with targeted monotherapy resulting from bypass mechanisms has forced researchers to employ either combination therapy or agents that interfere with multiple cell-signaling pathways. In this review, we present evidence that numerous agents identified from fruits and vegetables can interfere with several cell-signaling pathways. The agents include curcumin (turmeric), resveratrol (red grapes, peanuts and berries), genistein (soybean), diallyl sulfide (allium), S-allyl cysteine (allium), allicin (garlic), lycopene (tomato), capsaicin (red chilli), diosgenin (fenugreek), 6-gingerol (ginger), ellagic acid (pomegranate), ursolic acid (apple, pears, prunes), silymarin (milk thistle), anethol (anise, camphor, and fennel), catechins (green tea), eugenol (cloves), indole-3-carbinol (cruciferous vegetables), limonene (citrus fruits), beta carotene (carrots), and dietary fiber. For instance, the cell-signaling pathways inhibited by curcumin alone include NF-κB, AP-1, STAT3, Akt, Bcl-2, Bcl-XL, caspases, PARP, IKK, EGFR, HER2, JNK, MAPK, COX2, and 5-LOX. The active principle identified in fruit and vegetables and the molecular targets modulated may be the basis for how these dietary agents not only prevent but also treat cancer and other diseases. This work reaffirms what Hippocrates said 25 centuries ago, let food be thy medicine and medicine be thy food.

Keywords: NF-κB; AP-1; MAP kinases; Apoptosis; Cell cycle; Cancer; Dietary agents

Alastair Fitter, Robert Hay,

2 - Energy and Carbon,

Editor(s): Alastair Fitter, Robert Hay,

Environmental Physiology of Plants (Third Edition),

Academic Press,

2002,

Pages 23-73,

ISBN 9780122577666,

https://doi.org/10.1016/B978-0-08-054981-1.50007-9.

(https://www.sciencedirect.com/science/article/pii/B9780080549811500079)

Dariusz Kulus, Małgorzata Zalewska,

Cryopreservation as a tool used in long-term storage of ornamental species – A review,

Scientia Horticulturae,

Volume 168,

2014,

Pages 88-107,

ISSN 0304-4238,

https://doi.org/10.1016/j.scienta.2014.01.014.

(https://www.sciencedirect.com/science/article/pii/S0304423814000181)

Abstract: The market for ornamental plants is growing every year, becoming an important part of the economy. Every year hundreds of new cultivars, replacing the current assortment, are produced. However, since consumer preferences are changing rapidly, the cultivars considered old-fashioned today may become popular once again. They are also a valuable breeding material source. Bearing that in mind, there is a great need to develop a strategy for their long-term conservation. Storage in gene banks under in vitro cultures, although offering many advantages, is expensive and threatened with somaclonal variation and contamination loss. Cryopreservation is believed to be a more promising method. It has been successfully used with many agricultural species. Unlike micropropagation, cryopreservation has not yet found wider employment with ornamental plants. The upcoming years and progress in cryobiology may, however, change this situation and broaden the potential of cryoconservation. Over years several freezing methods have been introduced. The first one developed, based on slow cooling, had limited usefulness in temperate species. Today the encapsulation-dehydration technique is most often used with ornamental plants. In the future, however, combined techniques will probably be the most popular. So far insufficient attention has been paid to the problem of the genetic stability of cryopreserved ornamental species, especially chimeras. The aim of this paper is to present different cryopreservation techniques and their use for the storage, protection and breeding of ornamental plants.

Keywords: Cryopreservation; Genetic stability; Ornamental plants; Storage

Adrian Bolliger, Jakob Magid, Jorge Carneiro Telmo Amado, Francisco Skóra Neto, Maria de Fatima dos Santos Ribeiro, Ademir Calegari, Ricardo Ralisch, Andreas de Neergaard,

Taking Stock of the Brazilian “Zero‐Till Revolution”: A Review of Landmark Research and Farmers' Practice,

Advances in Agronomy,

Academic Press,

Volume 91,

2006,

Pages 47-110,

ISSN 0065-2113,

ISBN 9780120008094,

https://doi.org/10.1016/S0065-2113(06)91002-5.

(https://www.sciencedirect.com/science/article/pii/S0065211306910025)

Abstract: Two decades of extensive research and experimentation with zero‐till methods has allowed “ideal” zero‐till systems to emerge in Brazil, involving no soil turning, maintenance of a permanent vegetative cover, and rotations of both cash and cover crops. By exploiting rapid successions of suitable crops, for example, as well as through careful temporal and spatial planning, Brazilian examples show that it is possible to continuously cover soil, gradually increase soil organic matter (SOM) stocks, integrate livestock, move surface‐applied lime through the soil profile, break compact soil layers, and reduce reliance on agrochemicals in zero‐till, all under a variety of edaphic and climatic conditions, and levels of mechanization/farm sizes. Various such technologies and systems are reviewed in this chapter. However, we also note that among smallholder zero‐till farmers, for example, the adaptations of “ideal” zero‐till systems are manifold and complex, partial adoption of certain components and technologies rather than full adoption of zero‐till systems being the norm. By examining farmers' experiences and practice, we ascertain that in many cases there is perhaps a divorce between the ideal, originating mainly from individual technology research on agricultural research stations, and farmers' reality, given the complexity of socioeconomical constraints facing the latter. We conclude that although there is a wealth of valuable zero‐till experience and technologies precipitating from the Brazilian zero‐till “revolution,” numerous challenges in zero‐till research, especially in respect to resource‐poor smallholder farmers, still remain, and perhaps more holistic, participatory and adaptive on farm‐research is necessary in future. © 2006, Elsevier Inc.

Editor(s): Michael E. Peterson, Patricia A. Talcott,

Small Animal Toxicology (Second Edition),

W.B. Saunders,

2006,

Pages 1119-1190,

ISBN 9780721606392,

https://doi.org/10.1016/B0-72-160639-3/50087-3.

(https://www.sciencedirect.com/science/article/pii/B0721606393500873)

Peter A.G.M. De Smet,

Traditional pharmacology and medicine in Africa: Ethnopharmacological themes in sub-Saharan art objects and utensils,

Journal of Ethnopharmacology,

Volume 63, Issues 1–2,

1998,

Pages 1-175,

ISSN 0378-8741,

https://doi.org/10.1016/S0378-8741(98)00031-2.

(https://www.sciencedirect.com/science/article/pii/S0378874198000312)

Abstract: Drawing from the general description that ethnopharmacology studies the human use of crude drugs and poisons in a traditional context, ethnopharmacological themes in native art can be defined as themes visualizing different features of traditional medicines and poisons, such as natural sources, methods of preparation, containers, usage and implements, target diseases and effects. This review documents that native African art objects and utensils are a goldmine of such ethnopharmacological themes by focusing on the following subjects: (a) objects related to the use of medicines (sources as well as tools for their collection, preparation and keeping); (b) objects related to the use of poisons (e.g. for ordeals, hunting and fishing); (c) objects related to the use of psychotropic agents (e.g. alcoholic beverages, kola nuts, smoking and snuffing materials); (d) pathological representations (e.g. treponematoses, leprosy, smallpox, swollen abdomen, scrotal enlargement, goiter and distorted faces); and (e) portrayals of certain types of treatment (e.g. topical instillations, perinatal care, and surgery). To avoid the impression that ethnopharmacology has little else to offer than armchair amusement, an epilogue outlines the medical relevance of this interdisciplinary science for Western and African societies.

Keywords: Ethnopharmacology; Culture; Botany; Traditional medicine; Herbal medicine; Medicinal plants; Poisons; Psychotropic drugs; Alcohol; Tobacco; Central nervous system stimulants; Hallucinogens; Art in medicine; History of medicine; Human; Africa

David A. Bender,

Dictionary,

Editor(s): David A. Bender,

In Woodhead Publishing Series in Food Science, Technology and Nutrition,

Benders' Dictionary of Nutrition and Food Technology (Eighth Edition),

Woodhead Publishing,

2006,

Pages 1-521,

ISBN 9781845690519,

https://doi.org/10.1533/9781845691653.1.

(https://www.sciencedirect.com/science/article/pii/B9781845690519500016)

Editor(s): Klaus Aktories, Ulrich Förstermann, Franz Hofmann, Klaus Starke,

Allgemeine und spezielle Pharmakologie und Toxikologie (Elfte Ausgabe),

Urban & Fischer,

2013,

Pages 1101-1187,

ISBN 9783437425233,

https://doi.org/10.1016/B978-3-437-42523-3.18001-7.

(https://www.sciencedirect.com/science/article/pii/B9783437425233180017)

Arlen W. Frank,

6 - Dinucleotides,

Editor(s): Arlen W. Frank,

Chemistry of Plant Phosphorus Compounds,

Elsevier,

2013,

Pages 405-438,

ISBN 9780124071940,

https://doi.org/10.1016/B978-0-12-407194-0.00006-8.

(https://www.sciencedirect.com/science/article/pii/B9780124071940000068)

Abstract: This group comprises the pyridine adenine dinucleotides NAD and NADP, the riboflavin adenine dinucleotide FAD, and their respective dihydro derivatives. The pyridine dinucleotides are considered first.

Keywords: Dinucleotides; pyridine nucleotides; riboflavin nucleotides; occurrence; extraction; separation; analysis; biosynthesis; physical properties; plant names (both scientific and common)

S.I.R. Videira, J.Z. Groenewald, C. Nakashima, U. Braun, R.W. Barreto, P.J.G.M. de Wit, P.W. Crous,

Mycosphaerellaceae – Chaos or clarity?,

Studies in Mycology,

Volume 87,

2017,

Pages 257-421,

ISSN 0166-0616,

https://doi.org/10.1016/j.simyco.2017.09.003.

(https://www.sciencedirect.com/science/article/pii/S0166061617300398)

Abstract: The Mycosphaerellaceae represent thousands of fungal species that are associated with diseases on a wide range of plant hosts. Understanding and stabilising the taxonomy of genera and species of Mycosphaerellaceae is therefore of the utmost importance given their impact on agriculture, horticulture and forestry. Based on previous molecular studies, several phylogenetic and morphologically distinct genera within the Mycosphaerellaceae have been delimited. In this study a multigene phylogenetic analysis (LSU, ITS and rpb2) was performed based on 415 isolates representing 297 taxa and incorporating ex-type strains where available. The main aim of this study was to resolve the phylogenetic relationships among the genera currently recognised within the family, and to clarify the position of the cercosporoid fungi among them. Based on these results many well-known genera are shown to be paraphyletic, with several synapomorphic characters that have evolved more than once within the family. As a consequence, several old generic names including Cercosporidium, Fulvia, Mycovellosiella, Phaeoramularia and Raghnildiana are resurrected, and 32 additional genera are described as new. Based on phylogenetic data 120 genera are now accepted within the family, but many currently accepted cercosporoid genera still remain unresolved pending fresh collections and DNA data. The present study provides a phylogenetic framework for future taxonomic work within the Mycosphaerellaceae.

Keywords: Multi-gene phylogeny; Mycosphaerella; Plant pathogen; Taxonomy

Amani S. Awaad, Reham M. El-Meligy, Gamal A. Soliman,

Natural products in treatment of ulcerative colitis and peptic ulcer,

Journal of Saudi Chemical Society,

Volume 17, Issue 1,

2013,

Pages 101-124,

ISSN 1319-6103,

https://doi.org/10.1016/j.jscs.2012.03.002.

(https://www.sciencedirect.com/science/article/pii/S1319610312000397)

Abstract: Ulcerative colitis is an inflammatory chronic disease that affects the mucosa and submucosa of the colon and rectum. Several types of drugs are available such as aminosalicylates. Peptic ulcer disease (PUD) is a common disorder that affects millions of individuals worldwide and it can be considered one of the most important common diseases in the world. Treatment of peptic ulcers depends on using a number of synthetic drugs that reduce the rate of stomach acid secretion (Antiacids), protect the mucous tissues that line the stomach and upper portion of the small intestine (Demulcents) or to eliminate Helicobacter pylori (H. pylori). In most cases, incidence of relapses and adverse reactions is seen in the following synthetic antiulcer therapy. Accordingly, the main concern of the current article is to introduce a safe drug (or more) of natural origin, to be used for the management of gastric ulcers without side effects. A widespread search has been launched to identify new anti-ulcer therapies from natural sources. Herbs, medicinal plants, spices, vegetables and crude drug substances are considered to be a potential source to control various diseases including gastric ulcer and ulcerative colitis. In the scientific literature, a large number of medicinal plants and their secondary metabolites with potential anti-ulcer (anti-peptic ulcer and antiulcerative colitis) activities have been reported. Treatment with natural products produces promising results and fewer side effects. Our goal is to collect the published data in the last 24 years and reviews the natural products reported in the treatment of these diseases and their mechanism of action.

Keywords: Plant extracts; Gastroprotective; Flavonoids; Peptic ulcer; Anti-Helicobacter pylori; Ulcerative colitis

Nicholas John Sadgrove,

The new paradigm for androgenetic alopecia and plant-based folk remedies: 5α-reductase inhibition, reversal of secondary microinflammation and improving insulin resistance,

Journal of Ethnopharmacology,

Volume 227,

2018,

Pages 206-236,

ISSN 0378-8741,

https://doi.org/10.1016/j.jep.2018.09.009.

(https://www.sciencedirect.com/science/article/pii/S0378874118303325)

Abstract: Ethnopharmacological relevance

Research in the past half a century has gradually sketched the biological mechanism leading to androgenetic alopecia (AGA). Until recently the aetiological paradigm has been too limited to enable intelligent commentary on the use of folk remedies to treat or reduce the expression of this condition. However, our understanding is now at a point where we can describe how some folk remedies work, predict how effective they will be or why they fail.

Results

The new paradigm of AGA is that inheritance and androgens (dihydrotestosterone) are the primary contributors and a secondary pathology, microinflammation, reinforces the process at more advanced stages of follicular miniaturisation. The main protagonist to microinflammation is believed to be microbial or Demodex over-colonisation of the infundibulum of the pilosebaceous unit, which can be ameliorated by antimicrobial/acaricidal or anti-inflammatory therapies that are used as adjuvants to androgen dependent treatments (either synthetic or natural). Furthermore, studies reveal that suboptimal androgen metabolism occurs in both AGA and insulin resistance (low SHBG or high DHT), suggesting comorbidity. Both can be ameliorated by dietary phytochemicals, such as specific classes of phenols (isoflavones, phenolic methoxy abietanes, hydroxylated anthraquinones) or polycyclic triterpenes (sterols, lupanes), by dual inhibition of key enzymes in AGA (5α-reductase) and insulin resistance (ie., DPP-4 or PTP1B) or agonism of nuclear receptors (PPARγ). Evidence strongly indicates that some plant-based folk remedies can ameliorate both primary and secondary aetiological factors in AGA and improve insulin resistance, or act merely as successful adjuvants to mainstream androgen dependent therapies.

Conclusion

Thus, if AGA is viewed as an outcome of primary and secondary factors, then it is better that a ‘multimodal’ or ‘umbrella’ approach, to achieve cessation and/or reversal, is put into practice, using complementation of chemical species (isoflavones, anthraquinones, procyanidins, triterpenes, saponins and hydrogen sulphide prodrugs), thereby targeting multiple ‘factors’.

Keywords: Male pattern baldness; Androgenetic alopecia; 5α-reductase; Folk medicine; Microinflammation; Insulin resistance; Hydrogen sulphide prodrug

R. Harold Brown,

14 - Agronomic Implications of C4 Photosynthesis,

Editor(s): Rowan F. Sage, Russell K. Monson,

In Physiological Ecology,

C4 Plant Biology,

Academic Press,

1999,

Pages 473-507,

ISBN 9780126144406,

https://doi.org/10.1016/B978-012614440-6/50015-X.

(https://www.sciencedirect.com/science/article/pii/B978012614440650015X)

Cumulative general subject index volumes 1-30,

Editor(s): Atta-ur- Rahman,

Studies in Natural Products Chemistry,

Elsevier,

Volume 31,

2005,

Pages 3-869,

ISSN 1572-5995,

ISBN 9780444518781,

https://doi.org/10.1016/S1572-5995(05)80099-7.

(https://www.sciencedirect.com/science/article/pii/S1572599505800997)

Abstract: Publisher Summary

This chapter lists the important subjects that are discussed in the publication Studies in Natural Products Chemistry, Volumes 1–30, such as abdominal pain, acaricidal activities, acanthenes, gibberellins, glucoamylases, macarpine, and quercetin. The terms are mentioned along with the page numbers in which they are discussed in the publication.

Y. Marin-Felix, J.Z. Groenewald, L. Cai, Q. Chen, S. Marincowitz, I. Barnes, K. Bensch, U. Braun, E. Camporesi, U. Damm, Z.W. de Beer, A. Dissanayake, J. Edwards, A. Giraldo, M. Hernández-Restrepo, K.D. Hyde, R.S. Jayawardena, L. Lombard, J. Luangsa-ard, A.R. McTaggart, A.Y. Rossman, M. Sandoval-Denis, M. Shen, R.G. Shivas, Y.P. Tan, E.J. van der Linde, M.J. Wingfield, A.R. Wood, J.Q. Zhang, Y. Zhang, P.W. Crous,

Genera of phytopathogenic fungi: GOPHY 1,

Studies in Mycology,

Volume 86,

2017,

Pages 99-216,

ISSN 0166-0616,

https://doi.org/10.1016/j.simyco.2017.04.002.

(https://www.sciencedirect.com/science/article/pii/S0166061617300209)

Abstract: Genera of Phytopathogenic Fungi (GOPHY) is introduced as a new series of publications in order to provide a stable platform for the taxonomy of phytopathogenic fungi. This first paper focuses on 21 genera of phytopathogenic fungi: Bipolaris, Boeremia, Calonectria, Ceratocystis, Cladosporium, Colletotrichum, Coniella, Curvularia, Monilinia, Neofabraea, Neofusicoccum, Pilidium, Pleiochaeta, Plenodomus, Protostegia, Pseudopyricularia, Puccinia, Saccharata, Thyrostroma, Venturia and Wilsonomyces. For each genus, a morphological description and information about its pathology, distribution, hosts and disease symptoms are provided. In addition, this information is linked to primary and secondary DNA barcodes of the presently accepted species, and relevant literature. Moreover, several novelties are introduced, i.e. new genera, species and combinations, and neo-, lecto- and epitypes designated to provide a stable taxonomy. This first paper includes one new genus, 26 new species, ten new combinations, and four typifications of older names.

Keywords: DNA barcodes; Fungal systematics; Phytopathogenic fungi; Plant pathology; Taxonomy; Typifications

Ibrahim Marroun, Jacques Quevaulliers, Thomas Sené, Abe Fingerhut,

M,

Editor(s): Ibrahim Marroun, Jacques Quevaulliers, Thomas Sené, Abe Fingerhut,

Nouveau Dictionnaire Médical (Septième Édition),

Elsevier Masson,

2018,

Pages 518-571,

ISBN 9782294743573,

https://doi.org/10.1016/B978-2-294-74357-3.50021-4.

(https://www.sciencedirect.com/science/article/pii/B9782294743573500214)

Kapitel 36 - Wichtige Gifte und Vergiftungen,

Editor(s): Klaus Aktories, Ulrich Förstermann, Franz Hofmann, Klaus Starke,

Allgemeine und spezielle Pharmakologie und Toxikologie (Elfte Ausgabe),

Urban & Fischer,

2013,

Pages 967-1099,

ISBN 9783437425233,

https://doi.org/10.1016/B978-3-437-42523-3.00036-1.

(https://www.sciencedirect.com/science/article/pii/B9783437425233000361)

S. Wikee, L. Lombard, C. Nakashima, K. Motohashi, E. Chukeatirote, R. Cheewangkoon, E.H.C. McKenzie, K.D. Hyde, P.W. Crous,

A phylogenetic re-evaluation of Phyllosticta (Botryosphaeriales),

Studies in Mycology,

Volume 76,

2013,

Pages 1-29,

ISSN 0166-0616,

https://doi.org/10.3114/sim0019.

(https://www.sciencedirect.com/science/article/pii/S0166061614600087)

Abstract: Phyllosticta is a geographically widespread genus of plant pathogenic fungi with a diverse host range. This study redefines Phyllosticta, and shows that it clusters sister to the Botryosphaeriaceae (Botryosphaeriales, Dothideomycetes), for which the older family name Phyllostictaceae is resurrected. In moving to a unit nomenclature for fungi, the generic name Phyllosticta was chosen over Guignardia in previous studies, an approach that we support here. We use a multigene DNA dataset of the ITS, LSU, ACT, TEF and GPDH gene regions to investigate 129 isolates of Phyllosticta, representing about 170 species names, many of which are shown to be synonyms of the ubiquitous endophyte P. capitalensis. Based on the data generated here, 12 new species are introduced, while epitype and neotype specimens are designated for a further seven species. One species of interest is P. citrimaxima associated with tan spot of Citrus maxima fruit in Thailand, which adds a fifth species to the citrus black spot complex. Previous morphological studies lumped many taxa under single names that represent complexes. In spite of this Phyllosticta is a species-rich genus, and many of these taxa need to be recollected in order to resolve their phylogeny and taxonomy. Taxonomic novelties: New species – Phyllosticta abieticola Wikee & Crous, P. aloeicola Wikee & Crous, P. citrimaxima Wikee, Crous, K.D. Hyde & McKenzie, P. leucothoicola Wikee, Motohashi & Crous, P. mangifera-indica Wikee, Crous, K.D. Hyde & McKenzie, P. neopyrolae Wikee, Motohashi, Crous, K.D. Hyde & McKenzie, P. pachysandricola Wikee, Motohashi & Crous, P. paxistimae Wikee & Crous, P. podocarpicola Wikee, Crous, K.D. Hyde & McKenzie, P. rhaphiolepidis Wikee, C. Nakash. & Crous, P. rubra Wikee & Crous, P. vacciniicola Wikee, Crous, K.D. Hyde & McKenzie; New combinations – P. foliorum (Sacc.) Wikee & Crous, P. philoprina (Berk. & M.A. Curtis) Wikee & Crous; Epitypifications (basionyms) – P. concentrica Sacc., P. cussoniae Cejp, P. owaniana G. Winter; Neotypifications (basionyms) – Phyllosticta cordylinophila P.A. Young, Physalospora gregaria var. foliorum Sacc., Sphaeropsis hypoglossi Mont., Sphaeropsis minima Berk. & M.A. Curtis.

Keywords: Botryosphaeriales; foliar pathogens; Guignardia; Phyllosticta; Phyllostictaceae; Multi-Locus Sequence Typing (MLST); systematics

Arlen W. Frank,

2 - Phytic Acid,

Editor(s): Arlen W. Frank,

Chemistry of Plant Phosphorus Compounds,

Elsevier,

2013,

Pages 75-134,

ISBN 9780124071940,

https://doi.org/10.1016/B978-0-12-407194-0.00002-0.

(https://www.sciencedirect.com/science/article/pii/B9780124071940000020)

Abstract: Phytic acid, the hexaphosphate ester of myo-inositol, is unique to the plant kingdom, serving as the storage form of phosphorus in the higher plants. The lower myo-inositol phosphates, i.e. those with one to five phosphate groups, are covered separately in Chapter 3.

Keywords: Inositol phosphates; occurrence; extraction; separation; analysis; synthesis; biosynthesis; hydrolysis; phosphorylation; conformation; physical properties; plant names (both scientific and common).

**Scopus database**

Mazoir N., Benharref A., Vaca L., Reina M., González-Coloma A.,"23035575000	7004179768	57218852278	7005693899	57215378415	,

“Optimization of Insecticidal Triterpene Derivatives by Biomimetic Oxidations with Hydrogen Peroxide and Iodosobenzene Catalyzed by MnIII and FeIII Porphyrin Complexes",2020,"Chemistry and Biodiversity","17","9","e2000287","","",,1,"10.1002/cbdv.202000287","https://www.scopus.com/inward/record.uri?eid=2-s2.0-85090439422&doi=10.1002%2fcbdv.202000287&partnerID=40&md5=430ddf98947fd3e81d1ed8c8290b33c5","Semisynthetic functionalized triterpenes (4α,14-dimethyl-5α,8α-8,9-epoxycholestan-3β-yl acetate	4α,14-dimethyl-5α-cholest-8-ene-3,7,11-trione	4α,14-dimethyl-5α-cholesta-7,9(11)-dien-3-one and 4α,14-dimethyl-5α-cholest-8-en-3β-yl acetate), previously prepared from 31-norlanostenol, a natural insecticide isolated from the latex of Euphorbia officinarum, have been subjected to oxidation with hydrogen peroxide (H2O2) and iodosobenzene (PhIO) catalyzed by porphyrin complexes (cytochrome P-450 models) in order to obtain optimized derivatives with high regioselectivity. The main transformations were epoxidation of the double bonds and hydroxylations of non-activated C–H groups and the reaction products were 25-hydroxy-4α,14-dimethyl-5α-cholesta-7,9(11)-dien-3β-yl acetate (59 %), 25-hydroxy-4α,14-dimethyl-5α-cholest-8-ene-3,7,11-trione (60 %), 4α,14-dimethyl-5α,7β-7,8-epoxycholest-9(11)-en-3-one (22 %), 8-hydroxy-4α,14-dimethyl-5α-cholest-9(11)-ene-3,7-dione (16 %), 12α-hydroxy-4α,14-dimethyl-5α,7β-7,8-epoxycholest-9(11)-en-3-one (16 %), and 4α,14-dimethyl-5α,8α-8,9-epoxycholestan-3β-yl acetate (26 %), respectively. We also investigated the insect (Myzus persicae, Rhopalosiphum padi and Spodoptera littoralis) antifeedant and postingestive effects of these terpenoid derivatives. None of the compounds tested had significant antifeedant effects, however, all were more effective postingestive toxicants on S. littoralis larvae than the natural compound 31-norlanostenol, with 4α,14-dimethyl-5α,8α-8,9-epoxycholestan-3β-yl acetate being the most active. The study of their structure–activity relationships points out at the importance of C3 and C7 substituents. © 2020 Wiley-VHCA AG, Zurich, Switzerland",Article,"Final","",Scopus,2-s2.0-85090439422

Phumthum M., Balslev H.,"57214073551	6701731079	,Anti-infectious plants of the thai karen: A meta-analysis",2020,"Antibiotics","9","6","298","1","15",,3,"10.3390/antibiotics9060298","https://www.scopus.com/inward/record.uri?eid=2-s2.0-85086479392&doi=10.3390%2fantibiotics9060298&partnerID=40&md5=bfbe88afb9c2a3d696dbe3e49d796b45","Pharmacology has developed many drugs to treat infections, but many people, especially in developing countries, cannot afford to purchase them, and still depend on traditional knowledge and local medicinal plants to fight off infections. In addition, numerous microbes have developed resistance to the pharmaceutical drugs developed to fight them, and for many, such as Covid-19, effective drugs remain to be found. Ethnomedicinal knowledge is useful, not only for local people as a source of medicine for primary health care, but also for new pharmacological discoveries. This study aimed to identify the plants that the Karen, the largest hill-tribe ethnic minority in northern and western Thailand, use for treatments of infectious diseases. We present a meta-analysis of data from 16 ethnobotanical studies of 25 Karen villages with the aim of understanding traditional knowledge and treatments and point to potential plants for further pharmacological development. The Karen used 127 plant species from 59 plant families to treat infections and infectious diseases. The Cultural Important Index (CI) showed that the Leguminosae, Euphorbiaceae, Asteraceae, Lauraceae, Apocynaceae, Menispermaceae, and Lamiaceae were the most commonly used families. As for species, Cleidion javanicum, Tinospora crispa, Litsea cubeba, Aesculus assamica, Tadehagi triquetrum, Senna alata, Tithonia diversifolia, Embelia sessiliflora, and Combretum indicum were the most commonly used in treatments of infectious diseases. We suggest that these plant species should be the first to be pharmacologically tested for possible development of medicines, and the remaining species registered should subsequently undergo testing. © 2020 by the authors. Licensee MDPI, Basel, Switzerland.",Article,"Final","All Open Access, Gold, Green",Scopus,2-s2.0-85086479392

Abubakar I.B., Ukwuani-Kwaja A.N., Olayiwola F.S., Malami I., Muhammad A., Ahmed S.J., Nurudeen Q.O., Falana M.B.,"56448401500	57214808263	57214807805	56027041800	57205913645	57214797239	56150394800	57214803102	,An inventory of medicinal plants used for treatment of cancer in Kwara and Lagos state, Nigeria",2020,"European Journal of Integrative Medicine","34",,"101062","","",,3,"10.1016/j.eujim.2020.101062","https://www.scopus.com/inward/record.uri?eid=2-s2.0-85079170950&doi=10.1016%2fj.eujim.2020.101062&partnerID=40&md5=5259801f5f86e9a81e63a8569be89ec1","Introduction: The southwestern and northcentral parts of Nigeria are highly endowed with medicinal plants that have been used for decades to treat cancer. However, these herbal recipes and traditional medicinal practices have been scarcely investigated and documented. New uninvestigated plants could serve as potential sources for novel cytotoxic agents. This study was aimed at documenting the traditional medicinal practices used for treating cancers in Ilorin and Lagos metropolis. Methods: Information on herbal practices, medicinal plants and personal information form herbal practitioners was collected using questionnaires and oral interviews. Plants cited were collected, identified and assigned voucher numbers. The names of plants were further authenticated using the plant list (www.theplantlist.org) and the world flora (www.worldfloraonline.org). Results: A total of 41 plants were identified via oral interviews and questionnaires from 65 willing respondents within Ilorin and Lagos metropolis, respectively. Pistia stratiotes was the most frequently cited plant with a citation frequency of 57.1 % whereas, Mangifera indica Linn was the least cited plant with a citation frequency of 2.9 %. Plants parts including leaf, bark, root, and seed were prepared as concoctions, decoctions or powders and administered topically or orally to treat breast, prostate, cervical, skin and ovarian cancer. Conclusion: The study revealed that medicinal plants previously shown to have cytotoxicity in vitro are currently used for cancers in traditional medicine practice. Twenty six medicinal plants are mentioned here for the first time as anticancer plants and could serve as sources for novel cytotoxic agents against cancers. © 2020 Elsevier GmbH",Article,"Final","",Scopus,2-s2.0-85079170950

Bettar I., González-Miret M.L., Hernanz D., Marconi A., Heredia F.J., Terrab A.,"15733503100	6508390746	6507599635	56041605400	57209224029	6602101847	,Characterisation of Moroccan Spurge (Euphorbia) honeys by their physicochemical characteristics, mineral contents and colour",2019,"Arabian Journal of Chemistry","12","8",,"2052","2060",,14,"10.1016/j.arabjc.2015.01.003","https://www.scopus.com/inward/record.uri?eid=2-s2.0-85077098854&doi=10.1016%2fj.arabjc.2015.01.003&partnerID=40&md5=bd16d006fec66c46a06a9d5b651bd468","The quality of 27 Moroccan Spurge (Euphorbia) honey samples was assessed. Eight physicochemical parameters and mineral composition were analysed and the CIELAB colour parameters (L∗, a∗, b∗, Cab ∗ and hab) were determined. Results show no significant differences between the two Euphorbia honey types (Euphorbia officinarum subsp. echinus and Euphorbia regis-jubae honeys) regarding the physicochemical parameters. Sodium and magnesium show average values that can help to differentiate between E. officinarum subsp. echinus and E. regis-jubae honeys. Potassium was quantitatively the most important mineral (66% of the total minerals quantified), while sodium and calcium were present in moderate amounts (20% and 11% of the minerals, respectively). The colour parameters also have shown significant differences between E. officinarum subsp. echinus and E. regis-jubae honeys. Considering the total information from physicochemical, mineral and colour data, Principal Component Analysis (PCA) and Stepwise Discriminant Analysis (SDA) were carried out to distinguish between the two Euphorbia honey types. PCA showed that the cumulative variance was approximately 56%. The results of SDA showed that parameters with a higher discriminant power were Na, Mg, L∗, Cab ∗ and hab, and almost 100% of the samples were properly classified in their corresponding group, except for one sample. © 2019",Article,"Final","All Open Access, Gold",Scopus,2-s2.0-85077098854

Yazbek P.B., Matta P., Passero L.F., Santos G.D., Braga S., Assunção L., Sauini T., Cassas F., Garcia R.J.F., Honda S., Barreto E.H.P., Rodrigues E.,"57189727522	57210372234	10840752600	57225841284	57210372041	57210371615	57205761542	56741396900	57210376076	56740032700	57210370191	56370594500	,Plants utilized as medicines by residents of Quilombo da Fazenda, Núcleo Picinguaba, Ubatuba, São Paulo, Brazil: A participatory survey",2019,"Journal of Ethnopharmacology","244",,"112123","","",,6,"10.1016/j.jep.2019.112123","https://www.scopus.com/inward/record.uri?eid=2-s2.0-85070530165&doi=10.1016%2fj.jep.2019.112123&partnerID=40&md5=3ebd262fdcec3903b345015e2f700cd9","Ethnopharmacological relevance: Participatory research can help to broaden the understanding of medical systems and beliefs of traditional communities. An ethnopharmacological survey in collaboration with local people focused on plants used in quilombos located in Southeast Region in Brazil identified cultural factors that influence plant and recipe choice. Aim of the study: To investigate the factors related to the therapeutic efficiency of medicinal plants from the perspective of Quilombo da Fazenda residents. Materials and methods: University researchers collaborated with community residents for both aims and methods of the study. The local partners were trained in the gathering of ethnopharmacological data and then selected and interviewed the residents considered experts on the use of medicinal plants. Data on the use of each species were supported by voucher specimens collected by the local partners and university researchers. Participant observations and field diaries by the university researchers supplemented the data. Results: Eight interviewees mentioned 92 medicinal species with 60 therapeutic uses, applied in 208 recipes or remedies. Asteraceae (13 species), Lamiaceae (5) and Urticaceae (5) contributed most medicinal plant species. Of the 12 etic categories of use, the circulatory system category had the highest number of plants mentioned. Decoction was the most commonly used preparation method (66.8%), and most remedies were administered orally (76.4%). Eighty-six recipes included more than one plant species and/or the addition of other components, such as sugar, salt or animal products. Several cultural factors influence medicinal plant use. Popular beliefs on the quality of blood or the humoral properties of plants and illnesses, characteristics of the plants and other factors determine which plant is used and why. Conclusions: The participatory method identified a large number of factors that influence medicinal plant use: the patient's blood type	the condition of the plant and the disease (hot-cold system)	the route of administration and dosage	the preventive uses of the plants	and the influence of other factors, such as the sun, the moon and dew. The participatory approach is useful for gaining insight on the decision processes of medicinal plant use in traditional societies, and also for those communities wanting to document their knowledge with or without the participation of the academy. © 2019 Elsevier B.V.",Article,"Final","",Scopus,2-s2.0-85070530165

Smaili A., Rifai L.A., Mazoir N., Koussa T., Faize L., Alburquerque N., Burgos L., Makroum K., Malika B., Benharref A., Faize M.,"56928096600	6507556099	23035575000	6602911374	6506513336	6602456263	56233826700	6507319608	57203305559	7004179768	6603499766	,Semisynthetic Triterpenes Derived from Euphorbia officinarum as Plant Growth Promoters and Inducers of Disease Resistance",2019,"Journal of Plant Growth Regulation","38","1",,"262","272",,2,"10.1007/s00344-018-9838-3","https://www.scopus.com/inward/record.uri?eid=2-s2.0-85051201425&doi=10.1007%2fs00344-018-9838-3&partnerID=40&md5=3584e4a788320698d68db90c8b864209","This study evaluated the effect of application of the semisynthetic triterpenes 3β-acetoxy-norlup-20-one (F4) and 3-chloro-4α,14α-dimethyl-5α-cholest-8-ene (F6) triterpene derivatives from Euphorbia officinarum on the growth of tomato seedlings under normal conditions and when challenged with the pathogens Verticillium dahliae and Agrobacterium tumefaciens. Foliar spray of F4 and F6 significantly improved growth rate, fresh weight, dry weight, and leaf area. In addition, they enhanced several physiological parameters including photosynthetic pigments, proline content, and nitrate reductase activity. Moreover, they induced H 2 O 2 accumulation and increased the activity of several antioxidant enzymes such as catalase, ascorbate peroxidase, and guaiacol peroxidase. They also enhanced disease resistance against V. dahliae and A. tumefaciens. These results suggest that the two semisynthetic triterpenes represent new plant growth regulators and inducers of plant disease resistance. © 2018, Springer Science+Business Media, LLC, part of Springer Nature.",Article,"Final","",Scopus,2-s2.0-85051201425

Cabrera D.C., Chaila S., Sobrero M.T., Varela A.E.,"57057226500	6507048351	6603539778	57209011016	,Phytosociological survey of sugarcane crop weeds in different agroecological areas in TucumÁn Province, Argentina [Levantamento fitossociológico de plantas daninhas na cultura da cana-de-açúcar em diferentes regiões agroecológicas na Província de Tucumán, Argentina]",2019,"Planta Daninha","37",,"e019179380","","",,3,"10.1590/S0100-83582019370100027","https://www.scopus.com/inward/record.uri?eid=2-s2.0-85066266189&doi=10.1590%2fS0100-83582019370100027&partnerID=40&md5=d14480857cf11ce23a29158ed3b23fa7","Weeds constitute the main constraint on the productivity of sugarcane agroecosystems in Argentina. This work aimed to analyze the floristic and phytosociological composition of sugarcane weed communities in different agroecological areas in Tucumán. Fifteen plots from each area were sampled 60 to 90 days after crop shooting, by throwing a 0.5 m x 0.5 m quadrat metallic frame six times in random directions in each of the plots. Species found within the frame were identified. Later, fresh and dry biomass weight were calculated per quadrat sample and species. Frequency, density, dominance, importance value index, Shannon-Wiener and Simpson species diversity index and Jaccard similarity index were calculated. The unweighted pair group method with arithmetic mean (UPGMA) was used to interpret similarity. Overall, 35 species were identified, 24 of which were dicotyledons and 11 monocotyledons. Eighteen of them were annual species, versus 17 which were perennial. Considering IVI, the most outstanding families were Poaceae, Cyperaceae, Euphorbiaceae, Amaranthaceae and Asteraceae. The results obtained in all the areas revealed the importance of the species Panicum maximum, Sorghum halepense, Cyperus rotundus, Cynodon dactylon and Euphorbia hyssopifolia. The areas with greater and less diversity were the Depressed Plain–Non-Saline Depressed Plain Subregion and the Subhumid-Humid Chaco-Pampean Plain, respectively. There was a 53% similarity between the Depressed Plain–Saline Depressed Plain and the Depressed Plain–Non-Saline Depressed Plain, and a 47% similarity between the Humid and Perhumid Foothills and the Subhumid-Humid Chaco-Pampean Plain. Hierarchical clustering and Jaccard index led to similar results. © 2019, Sociedade Brasileira da Ciencia das Plantas Daninha. All rights reserved.",Article,"Final","All Open Access, Gold, Green",Scopus,2-s2.0-85066266189

Smaili A., Mazoir N., Aicha Rifai L., Koussa T., Makroum K., Belfaiza M., Benharref A., Faize M.,"56928096600	23035575000	57194196050	6602911374	6507319608	6508201253	7004179768	6603499766	,Induced resistance to wild fire disease of Nicotiana benthamiana using seed treated with triterpene derivatives from Euphorbia",2018,"Journal of Plant Pathology","100","1",,"75","83",,2,"10.1007/s42161-018-0031-0","https://www.scopus.com/inward/record.uri?eid=2-s2.0-85046399825&doi=10.1007%2fs42161-018-0031-0&partnerID=40&md5=defc649b279ad767d5b1ee5c96237226","In this study, an approach based on seed treatment was undertaken to enhance resistance of Nicotiana benthamiana against wild fire disease caused by Pseudomonas syringae pv. tabaci. Seeds soaked in 10 μg ml−1 of three hemi-synthetic triterpenes (F3, F4 and F6) derived from the latex of Euphorbia officinarum and Euphorbia resinifera resulted in plants with reduced disease severity, which was correlated with a decrease of bacterial populations in planta. Analysis of plant defense markers revealed that H2O2 and guaiacol peroxidase were only slightly activated but were primed after pathogen infiltration. However, polyphenol oxidase, catalase and ascorbate peroxidase were directly induced by the triterpenic derivatives. These results underline the duality of the mode of action of the triterpenic compounds and suggest their use as plant defense inducers in crop protection. © 2018, Società Italiana di Patologia Vegetale (S.I.Pa.V.).",Article,"Final","",Scopus,2-s2.0-85046399825

Bulut G., Haznedaroğlu M.Z., Doğan A., Koyu H., Tuzlacı E.,"6602494121	9633574300	55760462100	55823852200	6603356089	,An ethnobotanical study of medicinal plants in Acipayam (Denizli-Turkey)",2017,"Journal of Herbal Medicine","10",,,"64","81",,29,"10.1016/j.hermed.2017.08.001","https://www.scopus.com/inward/record.uri?eid=2-s2.0-85028505310&doi=10.1016%2fj.hermed.2017.08.001&partnerID=40&md5=6af22b6a40c234a47c2418313d40c7c0","A comprehensive ethnobotanical study was conducted in Acipayam, situated in the western part of Turkey. This paper includes details of plants used in folk medicine and ethnopharmacological information obtained during this study. The aim of the authors was to collect and identify plants used by local people for therapeutic purposes and to present information about traditional herbal medicine. Plant specimens collected during field‐work form the subject of this investigation. Information was obtained by means of open and semi-structured interviews with local people. In addition, cultural importance index (CI) and use report (UR) values were calculated. Ninety-one taxa of plants used in folk medicine and belonging to 38 families were identified in this study. Of these, 82 species were wild, and 9 species were cultivated. The most common families were Lamiaceae (18.7%), Asteraceae (14.3%) and Rosaceae (6.6%). Consequently, 191 medicinal uses (remedies) of 91 taxa were recorded. According to the use reports (UR), the most important medicinal plants were Cydonia oblonga (99 UR), Juniperus oxycedrus subsp. oxycedrus (99 UR), Pinus brutia (98 UR), Hypericum perforatum (90 UR), Viscum album subsp. austriacum (82 UR) and Salvia tomentosa (80 UR). Infusion (38.2%) was the most common preparation method used within the research area. The ethnomedicinal capabilities of eight species (Amelanchier parviflora var. dentata, Echinops viscosus subsp. bithynicus, Onopordum sibthorpianum, Origanum hypericifolium, Quercus trojana, Salvia adenophylla, Sideritis montana. subsp. remota and Tamarix smyrnensis) have been recorded for the first time in Turkey. © 2017",Article,"Final","",Scopus,2-s2.0-85028505310

Abolhasanzadeh Z., Ashrafi H., Badr P., Azadi A.,"36600022200	54415320600	8212035300	14719026400	,Traditional neurotherapeutics approach intended for direct nose to brain delivery",2017,"Journal of Ethnopharmacology","209",,,"116","123",,8,"10.1016/j.jep.2017.07.026","https://www.scopus.com/inward/record.uri?eid=2-s2.0-85025670231&doi=10.1016%2fj.jep.2017.07.026&partnerID=40&md5=dc1b9563e1e6ad827bee1aaa26e8b2ea","Ethnopharmacological relevance Nasal delivery systems have a significant role in Persian traditional medicine. Most of them were utilized for central nervous system (CNS)-related disorders. In modern medicine, nasal drug delivery systems for brain delivery are highly regarded. Aim of the study Despite recent advances in drug delivery to the (CNS), delivery of therapeutics to the brain remains a major challenge because of the blood brain barrier (BBB). There are several mechanisms which regulate the drug transfer across the BBB. Local administration methods of therapeutic agents are often associated with adverse events, while the intranasal pathway has been suggested as a non-invasive alternative route to deliver drugs to the brain. This route can bypass the BBB and deliver drug molecules directly to the CNS. There are different nasal formulations have been addressed in Persian traditional pharmacopeias. The present review attempt to explore the famous and practical Qarabadin to find ancient nasal dosage forms. Materials and methods With an explore on traditional herbs in google scholar, scopus and science direct, we have found some original and review articles which have demonstrated our findings on the use of traditional herbs for CNS disorders. Four encyclopedia of multi-component formulations, including Qarabadin Salehi (1766), Qarabadin kabir (1781),Qarabadin Ghaderi (18th century), and Qarabadin Azam (1853), were searched for nasal formulations having CNS-related indications. Formulations were categorized based on dosage forms, and also, diseases which they were suggested for. While the names of illnesses were in ancient terminology of Traditional Medicine, they were translated to modern medical terminology by comparing their definitions, signs, and symptoms from two medical systems. Typical samples of each dosage form have been mentioned with details like amount of ingredients, scientific names of plants, and considerations pertaining to preparation or usage. Results Among all traditional nasal formulations, seven types were found that is used for sicknesses relating to CNS including Saoot, Nafookh, Atoos, Nashoogh, Shamoom, Lakhlakheh, and Bakhoor. Conclusions The findings of this study reveal the physicochemical characteristics of each formulation, route of administration, and type of disease which they are suitable and also present some famous formulations. © 2017 Elsevier Ireland Ltd",Review,"Final","",Scopus,2-s2.0-85025670231

Ribeiro R.V., Bieski I.G.C., Balogun S.O., Martins D.T.D.O.,"36609215900	57193764372	37118565500	7006093336	,Ethnobotanical study of medicinal plants used by Ribeirinhos in the North Araguaia microregion, Mato Grosso, Brazil",2017,"Journal of Ethnopharmacology","205",,,"69","102",,77,"10.1016/j.jep.2017.04.023","https://www.scopus.com/inward/record.uri?eid=2-s2.0-85018764259&doi=10.1016%2fj.jep.2017.04.023&partnerID=40&md5=7596529fb97ce02b81971d270100805a","Ethnopharmacological importance Currently, in many traditional communities, such as the riverine community in the North Araguaia microregion (Mato Grosso, Brazil), plant knowledge and use represent the main, if not the only, therapeutic resource for the maintenance of health and/or treatment of diseases. This study aimed to identify and document species of medicinal plants used by local experts from riverine communities in the North Araguaia microregion in Mato Grosso State, and to further chemical and pharmacological studies on species selected based on searches in the relevant literature. Materials and methods This is a cross-sectional ethnobotanical study, with non-probabilistic sampling (n =60), that applied the snowball method to select local riverine experts who understand medicinal plant use. Socio-demographic, ethnobotanical and ethnopharmacological data (vernacular name, uses, geographical origin, habit, method of preparation and part used) on medicinal plants were collected during semi-structured interviews. The results were analyzed by descriptive and quantitative means: indices of use-report (UR) were used to select plant species with therapeutic potential. Results In total, 309 plant species belonging to 86 botanical families were cited	73% were native to Brazil, and Fabaceae was the most representative family (11.3%). Arboreal was the predominant life form (37.2%). The leaf was the most used part (28.9%). Infusion was the most commonly reported method of preparation (31.3%). The plants reported in the survey were indicated for 18 of the 22 ICD-10 disease categories. The disease categories most commonly cited were the infectious and parasitic diseases (IPD, 718 UR), digestive system diseases (DSD, 565 UR) and respiratory system diseases (RSD, 504 UR), representing 16.6%, 13.1% and 11.7%, respectively of the total UR. Dysphania ambrosioides L. was the most sighted in the IPD category 50 UR. Copaifera langsdorffii Desf. (133), Lafoensia pacari A. St.-Hil. (131), and Cecropia pachystachya Trécul (126) were the species with the highest UR. Bidens pilosa L., Vernonia ferruginea Less, and L. pacari, respectively, were the most cited native plants used to treat such diseases. Of the 8 investigated native plants, C. langsdorffii, and Brosimum gaudichaudii are the most prominent: in addition to having been widely studied, in terms of phytochemical and pharmacological, these species have been marketed as pharmaceutical products, with associated patent deposits. Conclusions Local riverine experts from the North Araguaia microregion use a wide variety of medicinal plants in self-care health, especially those species used to treat IPD. The therapeutic potential of some of these plants has been scientifically validated	however, there are other species whose pharmacological effects and safety remain to be properly investigated. Thus, the present study, aside from being a basis for future chemical, pharmacological and agronomic bioprospecting studies, may contribute to the development of the management, conservation and sustainable use of medicinal flora in the microregion studied. © 2017 Elsevier Ireland Ltd",Article,"Final","All Open Access, Bronze",Scopus,2-s2.0-85018764259

Smaili A., Mazoir N., Aicha Rifai L., Koussa T., Makroum K., Benharref A., Faize L., Alburquerque N., Burgos L., Belfaiza M., Faize M.,"56928096600	23035575000	57194196050	6602911374	6507319608	7004179768	6506513336	6602456263	56233826700	6508201253	6603499766	,Antimicrobial activity of two semisynthetic triterpene derivatives from euphorbia officinarum latex against fungal and bacterial phytopathogens",2017,"Natural Product Communications","12","3",,"331","336",,12,"10.1177/1934578x1701200305","https://www.scopus.com/inward/record.uri?eid=2-s2.0-85019153127&doi=10.1177%2f1934578x1701200305&partnerID=40&md5=57905ed4862801957ac358a2ffc4092d","This study evaluated the in vitro antimicrobial effect of 3β-acetoxy-norlup-20-one (1) and 3-chloro-4α,14α-dimethyl-5α-cholest-8-ene (2), triterpene derivatives from Euphorbia officinarum latex against fungal and bacterial phytopathogens. Results showed that although mycelial growth of several strains of Verticillium dahlia, and Fusarium oxysporum fsp. melonis and Penicillium expansum was affected only moderately, the two compounds were able to reduce highly conidia formation and germination, suggesting that they act as fungistatic compounds. Their antibacterial activity was tested against Pseudomonas syringae pv. syringae (Pss), P. syringae pv. tabacci (Pst), Erwinia amylovora (Ea) and Agrobacterium tumefaciens (At) using disc diffusion method. Results showed that compound 2 was more effective in inhibiting the growth of Pss, Pst and Ea than compound 1.",Article,"Final","All Open Access, Bronze",Scopus,2-s2.0-85019153127

Smaili A., Mazoir N., Rifai L.A., Koussa T., Makroum K., Kabil E.M., Benharref A., Faize M.,"56928096600	23035575000	6507556099	6602911374	6507319608	56928171000	7004179768	6603499766	,Triterpene derivatives from Euphorbia enhance resistance against Verticillium wilt of tomato",2017,"Phytochemistry","135",,,"169","180",,8,"10.1016/j.phytochem.2016.12.017","https://www.scopus.com/inward/record.uri?eid=2-s2.0-85008201504&doi=10.1016%2fj.phytochem.2016.12.017&partnerID=40&md5=0aeac8c695487e63a3bc15ac7541a446","Oxidation of α-euphorbol and 31-norlanostenol, two triterpenic compounds isolated from the latex of Euphorbia resinifera and Euphorbia officinarum respectively, yielded four products named 3β-tosyloxy-4α,14α-dimethyl-5α-cholesta-7,9-diene	4α,14α-dimethyl-5α-cholesta-7,9-dien-3β-ol	24-methylen-elemo-lanosta-8,24-dien-3-one and elemo-lanost-8-en-3,11,24-trione. They were evaluated for protection of tomato plants against Verticillium dahliae in a greenhouse. The four semisynthesized products were phytotoxic at higher concentrations as they completely inhibited tomato germination at 100 and 500 μg/ml. However at lower concentrations (10 and 50 μg/ml) germination and root length were not affected. Disease resistance against Verticillium wilt was assessed in tomato plants derived from seeds that germinated in the presence of 10 and 50 μg/ml of the four products. All of them were able to reduce significantly disease severity, with 10 μg/ml being more effective than 50 μg/ml. Reduction of leaf alteration index and of stunting index ranged from 52 to 68% and from 43 to 67%, respectively, while vessel discoloration was reduced by at least 95%. The compounds were also able to elicit H2O2 accumulation before and after fungal inoculation and to significantly enhance peroxidase and polyphenol oxidase activities. These results suggest that the hemisynthetized triterpenes can be used as elicitors of disease resistance. © 2016 Elsevier Ltd",Article,"Final","",Scopus,2-s2.0-85008201504

Giraldeli A.L., San Gregorio J.P.R., Monquero P.A., Aguillera M.M., Ribeiro N.M.,"57194276758	57194282866	9274221400	9739982700	57194286799	,Weeds hosts of nematodes in sugarcane culture [Plantas daninhas hospedeiras de nematoides na cultura da cana-de-açúcar]",2017,"Planta Daninha","35",,"e017156815","","",,,"10.1590/S0100-83582017350100029","https://www.scopus.com/inward/record.uri?eid=2-s2.0-85019600385&doi=10.1590%2fS0100-83582017350100029&partnerID=40&md5=63ccff416c917f80541b7671b049350e","The presence of weeds and nematodes can affect of sugarcane yield. This research evaluated whether weeds that are very frequent in mechanized harvested sugarcane can be hosts for root-knot nematodes: Meloidogyne incognita or Meloidogyne javanica. Regarding nematode hospitability, ten weed species as well as a control sample (tomato) were evaluated	they were inoculated with M. incognita and M. javanica. The plants were inoculated with 2,000 eggs and second-stage juveniles (J2), and there were three plants per experimental unit (6,000 eggs and J2 per pot - repeatition)	60 days after inoculation (DAI), the plants were removed and evaluated according to reproduction factor (RF), nematode final population (FP) and reproducibility index (RI). Regarding weed hospitability, it was found that Luffa aegyptiaca acted as a host for both nematodes, with RF &gt	1, which was higher than the control sample. Digitaria horizontalis was classified as a host for M. incognita and as a non-host for M. javanica. Mucuna aterrima and Crotalaria spectabilis presented the lowest RF and FP. Ricinus communis and Ipomoea triloba presented galls when inoculated with M. incognita, but were not considered host, since they presented RF &lt	1. None of the weed species was considered immune, i.e., with RF = 0. The lowest RF values of M. incognita (race 3) were related to Crotalaria spectabilis and Euphorbia heterophylla plants. This demonstrates the ability of crotalarias in decreasing nematode population in the field and justifies its use in several areas before planting main crops. © 2017, Sociedade Brasileira da Ciencia das Plantas Daninha. All rights reserved.",Article,"Final","All Open Access, Gold",Scopus,2-s2.0-85019600385

Bailen M., Khamlichi M.D., Benharref A., Martinez-Diaz R.A., Gonzalez-Coloma A.,"14008361100	57191361777	7004179768	6602193107	57215378415	,New bioactive semisynthetic derivatives of 31-norlanostenol and obtusifoliol from euphorbia officinarum",2016,"Natural Product Communications","11","6",,"733","738",,2,"10.1177/1934578x1601100609","https://www.scopus.com/inward/record.uri?eid=2-s2.0-84989154040&doi=10.1177%2f1934578x1601100609&partnerID=40&md5=e49e4c0f6cf185cd74cd2f8985721400","Fifteen semisynthetic terpenoid derivatives from the major latex components of Euphorbia officinarum have been evaluated against the insect pest Spodoptera littoralis, two species of protozoan parasites, Trypanosoma cruzi and Leishmania infantum, and also against insect Sf9 and mammalian CHO cells to test their selective cytotoxicity. Our results showed that 40% of the test substances were postingestive toxicants to S. littoralis. All the tested derivatives had cytotoxic effects on insect-derived Sf9 cells, whereas mammalian CHO cells were affected by a lower number of compounds (47%). Furthermore, 87% of the test compounds had antiparasitic effects on both L. infantum and T. cruzi, with some of them being selective parasite toxicants.",Article,"Final","All Open Access, Bronze, Green",Scopus,2-s2.0-84989154040

Kantati Y.T., Kodjo K.M., Dogbeavou K.S., Vaudry D., Leprince J., Gbeassor M.,"57118080200	57212644762	57118206200	6603869843	6603815221	6602595238	,Ethnopharmacological survey of plant species used in folk medicine against central nervous system disorders in Togo",2016,"Journal of Ethnopharmacology","181",,,"214","220",,16,"10.1016/j.jep.2016.02.006","https://www.scopus.com/inward/record.uri?eid=2-s2.0-84958214778&doi=10.1016%2fj.jep.2016.02.006&partnerID=40&md5=b2ac9c58515ea09aa762c496e3303524","Ethnopharmacological relevance Neurological diseases are rising all around the world. In a developing country such as Togo, although plant-based medicines are the only means, still very little is known regarding the nature and efficiency of medicinal plants used by indigenous people to manage central nervous system (CNS) disorders. Aim of the study This study, an ethnobotanical survey, aimed to report plant species used in traditional medicine (TM) for the management of various CNS disorders in Togo. Materials and methods 52 traditional actors (TA) including 33 traditional healers (TH) and 19 medicinal plant sellers (MPS) were interviewed, using a questionnaire mentioning informants' general data and uses of medicinal plants. Results The present study reports 44 medicinal plant species distributed into 26 families, mentioning scientific and common local names, plant organs used, preparation method, root of administration and putative applications. Conclusion It appears that there is a real knowledge on medicinal plants used for traditional treatment of CNS disorders in Togo and that the local flora abounds of potentially neuroactive plants which could be useful for the discovery of antipsychotic or neuroprotective molecules. © 2016 Elsevier Ireland Ltd. All rights reserved.",Article,"Final","",Scopus,2-s2.0-84958214778

Crous P.W., Wingfield M.J., Guarro J., Hernández-Restrepo M., Sutton D.A., Acharya K., Barber P.A., Boekhout T., Dimitrov R.A., Dueñas M., Dutta A.K., Gené J., Gouliamova D.E., Groenewald M., Lombard L., Morozova O.V., Sarkar J., Th Smith M., Stchigel A.M., Wiederhold N.P., Alexandrova A.V., Antelmi I., Armengol J., Barnes I., Cano-Lira J.F., Castañeda Ruiz R.F., Contu M., Courtecuisse P.R., Dasilveira A.L., Decock C.A., Degoes A., Edathodu J., Ercole E., Firmino A.C., Fourie A., Fournier J., Furtado E.L., Geering A.D.W., Gershenzon J., Giraldo A., Gramaje D., Hammerbacher A., He X.-L., Haryadi D., Khemmuk W., Kovalenko A.E., Krawczynski R., Laich F., Lechat C., Lopes U.P., Madrid H., Malysheva E.F., Marín-Felix Y., Martín M.P., Mostert L., Nigro F., Pereira O.L., Picillo B., Pinho D.B., Popov E.S., Rodas Peláez C.A., Rooney-Latham S., Sandoval-Denis M., Shivas R.G., Silva V., Stoilova-Disheva M.M., Telleria M.T., Ullah C., Unsicker S.B., Vandermerwe N.A., Vizzini A., Wagner H.-G., Wong P.T.W., Wood A.R., Groenewald J.Z.,"7101909107	7102265584	7103053290	36571730700	7201396174	7005757775	57224115927	7004979199	56719331100	7004115626	44861270900	7006527920	6507356966	8678888900	8537871100	35969878000	57221176308	56731111200	6603802331	6603273223	7102641324	55944787700	23092953500	7102125912	7201865981	7003936992	6508118568	56731141700	56730948000	7003372075	56731082300	24176898800	36782224100	24721268000	56462369900	7202900140	6603783423	7003752166	7004144359	55387191800	24331188200	53877364400	36008635000	56730783900	56731085300	8075028600	24280703500	6506447059	24171456400	35119232100	26656838200	15119177800	56177454800	7406971964	6603329600	36809682600	7004644592	56731107000	35722816000	8075028900	56730981600	8511964400	55827930300	7003656158	57213116822	6506207969	6701363104	56586073400	8233982300	56731074100	7003887651	56729697100	7403979927	56257244300	7004957342

,Fungal Planet description sheets: 320–370",2015,"Persoonia: Molecular Phylogeny and Evolution of Fungi","34",,,"167","266",,162,"10.3767/003158515X688433","https://www.scopus.com/inward/record.uri?eid=2-s2.0-84937545834&doi=10.3767%2f003158515X688433&partnerID=40&md5=7ccd970a7e6595ea952e82a75419f951","Novel species of fungi described in the present study include the following from Malaysia: Castanediella eucalypti from Eucalyptus pellita, Codinaea acacia from Acacia mangium, Emarcea eucalyptigena from Eucalyptus brassiana, Myrtapenidiella eucalyptorum from Eucalyptus pellita, Pilidiella eucalyptigena from Eucalyptus brassiana and Strelitziana malaysiana from Acacia mangium. Furthermore, Stachybotrys sansevieriicola is described from Sansevieria ehrenbergii (Tanzania), Phacidium grevilleae from Grevillea robusta (Uganda), Graphium jumulu from Adansonia gregorii and Ophiostoma eucalyptigena from Eucalyptus marginata (Australia), Pleurophoma ossicola from bone and Plectosphaerella populi from Populus nigra (Germany), Colletotrichum neosansevieriae from Sansevieria trifasciata, Elsinoë othonnae from Othonna quinquedentata and Zeloasperisporium cliviae (Zeloasperisporiaceae fam. nov.) from Clivia sp. (South Africa), Neodevriesia pakbiae, Phaeophleospora hymenocallidis and Phaeophleospora hymenocallidicola on leaves of a fern (Thailand), Melanconium elaeidicola from Elaeis guineensis (Indonesia), Hormonema viticola from Vitis vinifera (Canary Islands), Chlorophyllum pseudoglobossum from a grassland (India), Triadelphia disseminata from an immunocompromised patient (Saudi Arabia), Colletotrichum abscissum from Citrus (Brazil), Polyschema sclerotigenum and Phialemonium limoniforme from human patients (USA), Cadophora vitícola from Vitis vinifera (Spain), Entoloma flavovelutinum and Bolbitius aurantiorugosus from soil (Vietnam), Rhizopogon granuloflavus from soil (Cape Verde Islands), Tulasnella eremophila from Euphorbia officinarum subsp. echinus (Morocco), Verrucostoma martinicensis from Danaea elliptica (French West Indies), Metschnikowia colchici from Colchicum autumnale (Bulgaria), Thelebolus microcarpus from soil (Argentina) and Ceratocystis adelpha from Theobroma cacao (Ecuador). Myrmecridium iridis (Myrmecridiales ord. nov., Myrmecridiaceae fam. nov.) is also described from Iris sp. (The Netherlands). Novel genera include (Ascomycetes): Budhanggurabania from Cynodon dactylon (Australia), Soloacrosporiella, Xenocamarosporium, Neostrelitziana and Castanediella from Acacia mangium and Sabahriopsis from Eucalyptus brassiana (Malaysia), Readerielliopsis from basidiomata of Fuscoporia wahlbergii (French Guyana), Neoplatysporoides from Aloe ferox (Tanzania), Wojnowiciella, Chrysofolia and Neoeriomycopsis from Eucalyptus (Colombia), Neophaeomoniella from Eucalyptus globulus (USA), Pseudophaeomoniella from Olea europaea (Italy), Paraphaeomoniella from Encephalartos altensteinii, Aequabiliella, Celerioriella and Minutiella from Prunus (South Africa). Tephrocybella (Basidiomycetes) represents a novel genus from wood (Italy). Morphological and culture characteristics along with ITS DNA barcodes are provided for all taxa. © 2014-2015, Naturalis Biodiversity Center & Centraalbureau voor Schimmelcultures.",Article,"Final","All Open Access, Green",Scopus,2-s2.0-84937545834

Mazoir N., Belahyan A., Benharref A.,"23035575000	14119453100	7004179768	,New 1,3,4-thiadiazolines hemisynthesized from Moroccan endemic plants: Euphorbia officinarum latex",2015,"Journal of Materials and Environmental Science","6","12",,"3604","3608",,,,"https://www.scopus.com/inward/record.uri?eid=2-s2.0-84952773052&partnerID=40&md5=ac9ab32b7ea5d61162bf43d8cc004f31","Heterocycles are inextricably woven into the life processes. The vital interest of the pharmaceutical and agrochemical industries in heterocycles is often connected with their natural occurrence. The synthesis of novel heterocyclic derivatives and investigation of their chemical and biological behaviour have gained more importance in recent decades. Heterocyclic chemistry is an integral part of the chemical sciences and constitutes a considerable part of the modern researches that are occurring presently throughout the world. Hemisynthesis of 1,3,4-thiadiazolic triterpenes resulting from Euphorbia officinarum latex require initially preparation of their precursors, the thiosemicarbazones and gave thiadiazolic compounds characterized by new spiro carbons.",Article,"Final","",Scopus,2-s2.0-84952773052

Mazoir N., Benharref A.,"23035575000	7004179768	,Hemisynthesis of new thiosemicarbazone derivatives resulting from latex of Moroccan endemic plant: Euphorbia officinarum",2015,"Journal of Materials and Environmental Science","6","2",,"592","597",,2,,"https://www.scopus.com/inward/record.uri?eid=2-s2.0-84922749396&partnerID=40&md5=19af79c42a3777deed52a722a81215df","The thiosemicarbazone derivatives were prepared by introducing new functions on triterpenic skeletons. The product hemisynthezised from latex of Moroccan endemic plant: Euphorbia officinarum, were obtained with good yield and high regioselectivity.",Article,"Final","",Scopus,2-s2.0-84922749396

Mootoosamy A., Fawzi Mahomoodally M.,"55936695300	9537009000	,Ethnomedicinal application of native remedies used against diabetes and related complications in Mauritius",2014,"Journal of Ethnopharmacology","151","1",,"413","444",,79,"10.1016/j.jep.2013.10.069","https://www.scopus.com/inward/record.uri?eid=2-s2.0-84891496535&doi=10.1016%2fj.jep.2013.10.069&partnerID=40&md5=19bcffe0cfcdb34f1f8664c2f4660fcc","Ethnopharmacological relevance Notoriously, the tropical island of Mauritius has one of the highest prevalence of diabetes worldwide and the economic burden associated with it is alarming. The use of native remedies (NRs) is well anchored in the local culture and it continues to be the cornerstone of therapy for diabetic patients. However, there is currently a dearth of updated primary data on NRs used by Mauritians against diabetes and diabetes related complications (DRCs). This study was therefore designed to record, analyze and document orally transmitted ethnopharmacological knowledge from diabetic patients and traditional medicine practitioners (TMPs) in Mauritius concerning NRs commonly used against diabetes and DRCs which might open new avenues to initiate novel antidiabetic drugs discovery. Materials and methods Data was collected following interviews from diabetic patients (n=328) and TMPs (n=20). Eleven quantitative indexes, namely informant consensus factor (FIC), fidelity level (FL), use value (UV), relative frequency of citation (RFC), relative importance (RI), cultural importance index (CII), index of agreement on remedies (IAR), cultural agreement index (CAI), quality use value (QUV), quality use agreement value (QUAV) and ethnobotanicity index (EI) were calculated. Statistical analysis such as Pearson correlation and Chi-squared test were performed to determine any association. Results A total of 111 plant species distributed over 56 families, 30 polyherbal formulations and 16 animal species were documented to be traditionally used against diabetes and DRCs. For the first time 8 endemic plants have been recorded to be used against diabetes and DRCs from Mauritius. The most encountered medicinal plant family was Asteraceae. According to the EI, 16.2% of the native plants in Mauritius were used against diabetes and DRCs. As far as we know, Vangueria madagascariensis, Apium graveolens, Petroselinum crispum and Rubus alceifolius with high RFC values are recorded against diabetes and DRCs for the first time. Sociodemographic characteristics (age, gender, income, religious belief, education and residence) were found to significantly (p<0.05) influence the use of NRs. The average FIC for all ailments for plant and animal products were 0.94 and 0.87 respectively. Bryophyllum pinnatum, a native plant to Mauritius scored a high FL value (100%) used against diabetic neuropathy, Allium sativum had the highest RI value (2.00) due to its versatility, Aloe vera had the highest RFC (0.61), the CII (0.640) and the highest CAI value (0.635), Psidium guajava had the highest QUAV (0.961) which indicates its high bioactivity and Allium cepa was reported as the most effective plant species (QUV=0.965). According to UV, the most important species was Morinda citrifolia (1.21). Panoply of animal products were reported whereby fish (39.7%) was recorded as the most utilised zootherapy and Salmo salar scored the highest FL (100%) for diabetes. Some animal species (n=14) not previously documented against diabetes and DRCs are reported in the present study. Conclusion Our present investigation revealed that the use of NRs constitutes the common legacy of Mauritians and despite the penetration of allopathic medicine	NRs continue to play a crucial role in the primary health care system of Mauritius. To this effect, it is of uttermost importance to record this knowledge before it disappears. In addition, further experimental investigations are required to elucidate the pharmacological properties of the reported medicinal flora and fauna of Mauritius. © 2013 Elsevier Ireland Ltd.",Article,"Final","",Scopus,2-s2.0-84891496535

Terrab A., Marconi A., Bettar I., Msanda F., Díez M.J.,"6602101847	56041605400	15733503100	6507260242	35492588400	,Palynological characterisation of Euphorbia honeys from Morocco",2014,"Palynology","38","1",,"138","146",,8,"10.1080/01916122.2013.871797","https://www.scopus.com/inward/record.uri?eid=2-s2.0-84899586877&doi=10.1080%2f01916122.2013.871797&partnerID=40&md5=b9fa932602bd76c25afe4cffdd70950b","Pollen was analysed in 30 unifloral Euphorbia honey samples from the Ifni Massif Region (Anti Atlas, Morocco). The honey samples were directly provided by beekeepers. The quantitative analyis showed that nectar is the main honey source in the samples studied. The qualitative analysis of the samples showed the presence of 35 taxa belonging to 17 families. The Moroccan Euphorbia honeys of the studied region are characterized by their low-medium number of pollen grains (NGP	mean = 5700), 76% of the honeys belong to Class I and II of Maurizio, and by their low honeydew indicator (HDE/NGPn < 0.28), wich indicates their floral origin. For the Euphorbia officinarum subsp. echinus honeys, the most characteristic accompanying species are Eryngium ilicifolium, present in > 90% of the samples, followed by Bellis sp., Capsella f. and Reseda sp. (85%). However, for the E. regis-jubae honeys, the most characteristic accompanying species are Crepis f., present in 100% of the samples. © 2014 © 2014 AASP - The Palynological Society.",Article,"Final","",Scopus,2-s2.0-84899586877

Banne S.T., Nayak S., Girigaon Y., Parida N., Naik M.,"56372425100	57209561558	56372492800	56372609400	56372336000	,Anti-hemorrhoidal drugs of Bhavaprakash Nighantu: A literary survey",2014,"International Journal of Research in Ayurveda and Pharmacy","5","4",,"551","557",,,"10.7897/2277-4343.054111","https://www.scopus.com/inward/record.uri?eid=2-s2.0-84907465116&doi=10.7897%2f2277-4343.054111&partnerID=40&md5=fd633df5ae814e95e400877db6db3cc5","Hemorrhoid is a lifestyle disease. Lifestyle disease is caused by a change in the way in which people live, as a result of the advancements in society or its scientific progress. Lack of adequate physical activity, no exercise, smoking, consumption of alcohol and irregular lifestyle patterns can be one of the main causes of these diseases. Hemorrhoid is one such lifestyle related disease. It can be effectively treated with over-the-counter medications, good fluid intake and by following a diet rich in fiber. Diet failure leads to many diseases including hemorrhoids, constipation, irritable bowel syndrome, ulcerative colitis and Crohn's disease. In severe cases, piles may require surgical intervention. Ayurveda plays vital role in curing Arsha (Hemorrhoids) and is explained under mamsaroga. Many treatment modalities have been explained in Brihatrayi (Charak, Sushruta, Vagbhata) and Laghutrayi (Sharangdhara, Bhavaprakash and Madhav Nidan). Bhavaprakash mentions numerous drugs acting on hemorrhoids. The present study was designed to screen the drugs with Arshoghna, Visheshat Gudajapaham and Raktarshara, Gudaraktahruta, Gudankurnash, Gudartinash, Vatarshahara properties. Out of 426 drugs 82 were described for the treatment of hemorrhoids throughout the text which includes herbal, mineral and animal origin drugs.",Review,"Final","All Open Access, Bronze",Scopus,2-s2.0-84907465116

Beck N.R., Samal P.,"54898920200	37087680700	,Traditional medicinal plants used by the tribes and rural people of Bilaspur District, Chhattisgarh (India)",2012,"Research Journal of Pharmacy and Technology","5","10",,"1281","1282",,4,,"https://www.scopus.com/inward/record.uri?eid=2-s2.0-84875136806&partnerID=40&md5=9d8754ac259023a7cbdaad7649bd6856","Herbs are still an important part of traditional healing systems. Drugs of herbal origin are being used for many degenerative diseases like liver damage, arthritis, cardiovascular disorders, diabetes, cancer etc. Herbal preparations are natural, intrinsically harmless and produce desired pharmacological effects. There are many tribal communities live in Chhattisgarh state having great deal of knowledge about the utility of the uses of medicinal plants. The Literature reveals that about 6,000 Traditional Healers are still practicing the knowledge of herbal medicines in the state. They adopt traditional diagnostic methods and prescribe drugs in treatment of internal as well as external common as well as complicated diseases. This paper reports about the use of thirty five plant species for the treatment of various ailments by the rural people of Bilaspur District. The study emphasizes the need for ethno botanical exploration in order to revive the traditional knowledge for the healthcare systems in India. © RJPT All right reserved.",Article,"Final","",Scopus,2-s2.0-84875136806

Liu Q., Luyten W., Pellens K., Wang Y., Wang W., Thevissen K., Liang Q., Cammue B.P.A., Schoofs L., Luo G.,"12766228400	15763156800	8314889300	57201318138	56701360100	6602675943	7102360401	55977802400	57189882662	7401536462	,Antifungal activity in plants from Chinese traditional and folk medicine",2012,"Journal of Ethnopharmacology","143","3",,"772","778",,37,"10.1016/j.jep.2012.06.019","https://www.scopus.com/inward/record.uri?eid=2-s2.0-84866738785&doi=10.1016%2fj.jep.2012.06.019&partnerID=40&md5=7ef5d5381ac1a7cac037add6824e918e","Ethnopharmacological relevance: From over 100 Chinese clinical trial publications, we retrieved 22 commercial preparations and 17 clinical prescriptions used as Traditional Chinese Medicine (TCM) for treating mycotic vaginitis, typically caused by Candida albicans. The 8 most frequently used plants as well as another 7 TCM and 18 folk medicinal plants used in the South of China for antifungal therapy were investigated for in vitro antifungal activity. Materials and methods: For each plant we tested 4 extracts prepared with different solvents (water, ethanol, acetone, and n-hexane) for inhibition of Candida albicans and Saccharomyces cerevisiae growth in liquid culture. Results: Some plants have quite strong antifungal activity, such as Tujinpi (Pseudolarix kaempferi Gord.), of which each extract could significantly inhibit the growth of both tested fungi. In addition, the acetone extract of Kushen (Sophora flavescens Ait.), the ethanol, acetone, and hexane extracts of Guanghuoxiang (Pogostemon cablin (Blanco) Benth.) and Gaoliangjiang (Alpinia officinarum Hance), the hexane extract of Dingxiang (Eugenia caryophyllata Thunb.), and the ethanol and acetone extracts of Kulianpi (Melia toosendan Sieb. et Zucc.) and Laliao (Polygonum hydropiper L.), all inhibited Candida albicans growth by more than 50%. In some cases growth inhibition was even comparable to that by the clinically used antifungal miconazole, which we used as our positive control. Conclusions: The majority of plants, whose clinical use for antifungal treatment is well supported within TCM or Chinese folk medicine, show in vitro antifungal activity against Candida albicans. Since Candida species represent the most common fungal pathogen of humans, these results provide more scientific evidence supporting the clinical application of these plants, and can serve as a starting point for new drug discovery from TCM and Chinese folk medicine. © 2012 Elsevier Ireland Ltd. All rights reserved.",Article,"Final","",Scopus,2-s2.0-84866738785

Sharma J., Gairola S., Gaur R.D., Painuli R.M.,"57197185230	33867554400	7004385614	36638154100	,The treatment of jaundice with medicinal plants in indigenous communities of the Sub-Himalayan region of Uttarakhand, India",2012,"Journal of Ethnopharmacology","143","1",,"262","291",,72,"10.1016/j.jep.2012.06.034","https://www.scopus.com/inward/record.uri?eid=2-s2.0-84864718978&doi=10.1016%2fj.jep.2012.06.034&partnerID=40&md5=b342c734c0f82798219fdd5e7511406b","Ethnopharmacological relevance: Inspite of tremendous advances made in allopathic medical practices, herbs still play an important role in the management of various liver diseases. A large number of plants and formulations have been claimed to have hepatoprotective activity. Jaundice is a symptom, indicative of the malfunctioning of the liver. This paper provides ethnomedicinal information on the plants used to treat jaundice by three important indigenous communities, i.e.	nomadic Gujjars, Tharu and Bhoxa of Sub-Himalayan region, Uttarakhand, India. Aims of the study: To record herbal preparations used by the studied indigenous communities in treatment of jaundice and discuss hepatoprotective properties of the recorded plants. Research strategy and methods: The traditional knowledge of the studied indigenous communities on herbal preparations used for treating jaundice was collected through structured questionnaire and personal interviews. The interviews were conducted with 91 traditional healers (29 Bhoxa, 35 Tharu and 27 nomadic Gujjars) in Sub-Himalayan region of Uttarakhand, India. More than 250 research papers reporting ethnomedicinal information on the hepatoprotective plants used by various communities from different parts of India were extensively reviewed. Results: A total of 40 medicinal plants belonging to 31 families and 38 genera were recorded to be used by the studied communities in 45 formulations as a remedy of jaundice. Bhoxa, nomadic Gujjars and Tharu communities used 15, 23 and 9 plants, respectively. To our knowledge eight plants reported in the present survey viz.	Amaranthus spinosus L.	Cissampelos pareira L.	Ehretia laevis Roxb.	Holarrhena pubescens Wall.	Ocimum americanum L.	Physalis divaricata D. Don, Solanum incanum L. and Trichosanthes cucumerina L. have not been reported earlier as remedy of jaundice in India. Literature review revealed that a total of 214 (belonging to 181 genus and 78 families), 19 (belonging to 18 genus and 12 families) and 14 (belonging to 14 genus and 11 families) plant species are used as internal, external and magico-religious remedies for jaundice, respectively by various communities in different parts of India. Most widely used hepatoprotective plant species for treatment of jaundice in India is Boerhavia diffusa L. followed by Tinospora cordifolia (Willd.) Miers, Saccharum officinarum L.	Phyllanthus amarus Schumach. & Thonn.	Ricinus communis L.	Andrographis paniculata (Burm. f.) Nees.	Oroxylum indicum (L.) Kurz, Lawsonia inermis L. and Eclipta prostrata (L.) L. Conclusions: The plants recorded in the present survey have also been discussed in relation to pharmacological studies and hepatoprotective phytoconstituents present in them. Most of the recorded plants have shown hepatoprotective effects on experimental animals in earlier studies but more studies are needed to assess hepatoprotective properties of some recorded medicinal plants viz.	Averrhoa carambola L.	Ehretia laevis Roxb.	Holarrhena pubescens Wall.	Mangifera indica L.	Ocimum americanum L.	Oroxylum indicum (L.) Kurz, Physalis divaricata D. Don, Solanum incanum L.	Sphaeranthus senegalensis DC. and Tribulus terrestris L. The plants enumerated in this study with high number of citations and wider distributions have given some useful leads for further biomedical research. Nevertheless more phytochemical, pharmaceutical and clinical studies are needed to evaluate hepatoprotective properties, efficacy and safety of all the claimed medicinal plants. © 2012 Elsevier Ireland Ltd.",Article,"Final","",Scopus,2-s2.0-84864718978

Polat R., Satil F.,"42862270600	55929315200	,An ethnobotanical survey of medicinal plants in Edremit Gulf (Balikesir - Turkey)",2012,"Journal of Ethnopharmacology","139","2",,"626","641",,150,"10.1016/j.jep.2011.12.004","https://www.scopus.com/inward/record.uri?eid=2-s2.0-84856008876&doi=10.1016%2fj.jep.2011.12.004&partnerID=40&md5=8c0bec98fecdb88b468ea753f9425d01","Ethnopharmacological relevance: This paper provides significant ethnobotanical information on medical plants in the Western Region of Turkey. There is urgency in recording such data. This is the first ethnobotanical study in which statistical calculations about plants are done by FIC method in western part of Turkey. Aim of study: This study aimed to identify plants collected for medical purposes by the local people of Edremit Gulf, located in the Western Region of Turkey, and to document the uses and local names of these plants. Materials and methods: This study, conducted between 2007 and 2010, gathered information on the medicinal plant species traditionally used in Edremit Bay, Turkey and the local names of these plants. In the scope of the study, medicinals plant species and related information were collected	herbarium materials were prepared	and the specimens were entitled. Field research was conducted by collecting ethnobotanical information during structured and semi-structured interviews with native knowledgeable people in territory. In addition, the relative importance value of species was determined and informant consensus factor (FIC) was calculated for the medicinal plants included in the study. Results: A total of 118 medical plants belonging to 50 families were identified in the region. Among them, 99 species are wild and 19 species are cultivated plant. The most common medicinal plant families were Lamiaceae (>18%), Asteraceae (>11%), Rosaceae (>7%)	the most common preparations were infusion and decoction. It was found that Hypericum perforatum, Lavandula stoechas, Salviatomentosa, Origanum onites, Origanum vulgare, and Teucrium polium were the most commonly used species. A total of 218 medicinal uses (remedies) were recorded. The traditional medicinal plants have been mostly used for the treatment of abdominal and stomach pain (17%), cough and cold (12%), diabetes (6%), kidney ailments (5%), wounds (4%). Conclusion: The use of traditional medicine was still widespread among the people interviewed during this study. Due to the increasing health service facilities in the area, herbal medicine, seemed to be more related to health care and disease prevention than cure. © 2011 Elsevier Ireland Ltd.",Article,"Final","",Scopus,2-s2.0-84856008876

López-Muñoz F., Álamo C., García-García P.,"20534769400	56227867900	6602451902	,Tosigos and antidotes in Cervantine literature: On the poisons in the late renaissance spain [Tósigos y antídotos en la literatura cervantina: Sobre los venenos en la españa tardorrenacentista]",2011,"Revista de Toxicologia","28","2",,"119","134",,1,,"https://www.scopus.com/inward/record.uri?eid=2-s2.0-84866440632&partnerID=40&md5=9432fb2c1b66944ad92c200879345d03","Cervantine texts are an interesting source for the study of Spanish late Renaissance society. We have studied the works of Cervantes from the toxicology viewpoint, analyzing the use of toxic and poisonous substances, essentially hallucinogenic and narcotic agents in the field of witchcraft and sorcery practice (witches ointments, love filters, poisonous potions, etc.), as well as references to hypothetical substances endowed with alexipharmic action, as the vervain (Verbena officinalis), unicorn horn, bezoar stones or castoreum. Cervantine works which refers to these preparations are the novels Don Quixote, The Galatea, Voyage to Parnassus and four Exemplary Novels (The Spanish-English Lady, The Licentiate of Glass, The Jealous Extremaduran and The Colloquy of the Dogs), as well as the comedies Pedro de Urdemalas, The Amusing Woman and The Labyrinth of Love. Toxic agents of herbal origin mentioned explicitly by Cervantes in the analyzed context include the henbane (Hyoscyamus niger/albus), tobacco (Nicotiana tabacum), oleander (Nerium oleander), spurge (Euphorbia lathyris), rhubarb (Rheum officinale, Rumex alpinus), and masked way, opium (Papaver somniferum). In relation to the rest of preparations endowed with toxic activity, Cervantes does not identify its ingredients, though, within the meaning of the symptoms described by the author, they could be plants of the Solanaceae family, as the henbane, nightshade, jimsonweed, belladonna or mandrake. Concerning toxic minerals, only there are references to the effects of mercury (""azogue""). The Dioscorides commented by Andrés Laguna could be the technical font used by Cervantes to document in this matter.",Article,"Final","",Scopus,2-s2.0-84866440632

Correia N.M., Kronka B.,"35336545400	36809892900	,Efficacy of herbicides applied during the dry and wet seasons for Euphorbia heterophylla control in sugarcane [Eficácia de herbicidas aplicados nas épocas seca e úmida para o controle de Euphorbia heterophylla na cultura da cana-de-açúcar]",2010,"Planta Daninha","28","4",,"853","863",,2,"10.1590/s0100-83582010000400019","https://www.scopus.com/inward/record.uri?eid=2-s2.0-78751473553&doi=10.1590%2fs0100-83582010000400019&partnerID=40&md5=33841b6ff07d7d06d7cf2db9131bc0e6","An experiment was carried out in a commercial sugarcane plantation area in Jaboticabal, Sao Paulo, Brazil, from August 2008 to June 2009 to evaluate the effect of herbicides applied in preemergence and postemergence, alone and combination, during the dry and wet seasons, for poinsettia control (Euphorbia heterophylla). The experiment was arranged in a randomized block design in split-plot, and four replications. Three herbicide treatments [amicarbazone (1.4 kg ha-1),imazapic (0.147 kg ha-1) and sulfentrazone (0.9 kg ha-1 plus 0.2 kg ha-1),applied on August 01, 2008 after sugarcane harvest, and one treatment without herbicide were tested. The herbicides used during the wet season were: Mesotrione alone (0.192 kg ha-1), and in mixture (0.12 kg ha-1) with ametryn (1.5 kg ha-1), atrazine (1.5 kg ha-1) or diuron plus hexazinone (0.702 plus 0.198 kg ha-1), applied in postemergence on November 14, 2008, and two treatments without herbicide. The application of imazapic during the dry season was efficient for E. heterophylla control, without the need of management complementation during the wet season. However, the amicarbazone and sulfentrazone treatments required mesotrione application, alone or mixed with ametryn, atrazine or diuron, plus hexazinone to control E. heterophylla during the wet season (105 to 230 days after application during the dry season). Without herbicide application during the dry season, mesotrione mixed with ametryn, atrazine or diuron plus hexazinone resulted in a better weed control than when applied alone. Despite the visual symptoms of phytointoxication caused by the treatments mesotrione plus ametryn and mesotrione plus (diuron plus hexazinone), none of the types of management adopted or combinations between them interfered in the number of viable stalk by linear meter, and in stalk diameter and height in sugarcane.",Article,"Final","All Open Access, Gold, Green",Scopus,2-s2.0-78751473553

Al-Qura'n S.,"8605460600	,Ethnopharmacological survey of wild medicinal plants in Showbak, Jordan",2009,"Journal of Ethnopharmacology","123","1",,"45","50",,152,"10.1016/j.jep.2009.02.031","https://www.scopus.com/inward/record.uri?eid=2-s2.0-64249109576&doi=10.1016%2fj.jep.2009.02.031&partnerID=40&md5=e20b3f0d58bd66617c552e7dbc767193","Two main research questions are framing this investigation: (1) the main taxa of the medicinal importance value altered the Showbak forest stand and species composition? (2) The most safe species and what are the toxic ones (unsafe). These two research questions are the vital ones to draw a clear image about the wild medicinal plants of this investigated area of Showbak region in Jordan. 79 wild medicinal plant species were investigated in this study which are used in traditional medication for the treatment of various diseases. Most of the locals interviewed dealt with well-known safe medicinal plants such as Aaronsohnia factorovskyi Warb. et Eig., Achillea santolina L., Adiantum capillus-veneris L., Artemisia herba-alba L., Ceratonia siliqua L., Clematis recta L., Herniaria hirsuta L., Malva neglecta Wallr., Rosmarinus officinalis L., Ruta chalepensis L., Salvia triloba L., Sarcopoterium spinosa (L.) Spach., Thymbra capitata (L.) Hof, and Urginea maritima Barker. Many of the wild medicinal plants investigated were toxic and needed to be practiced by practitioners and herbalists rather than the local healers. These plants include Calotropis procera Willd R.Br., Citrullus colocynthis (L.) Sch., Datura stramonium L., Digitalis purpurea L., Ecballium elaterium (L.) A.Rich., Euphorbia helioscopia L., Euphorbia tinctoria Boiss., Glaucium corniculatum (L.) Curt., Hyoscyamus aureus L., Mandragora officinarum L., Nerium oleander L., Ricinus communis L., Solanum nigrum L., Withania somnifera (L.) Dunel. The conservation of medicinal plants and natural resources is becoming increasingly important, so this research is trying to collect information from local population concerning the use of medicinal plants in Showbak	identify the most important specie	determine the relative importance value of the species and calculate the informant consensus factor (ICF) for the medicinal plants. Obtaining results is relied on the interviewee's personal information and the medicinal use of specific plants. © 2009 Elsevier Ireland Ltd. All rights reserved.",Article,"Final","",Scopus,2-s2.0-64249109576

Marc E.B., Nelly A., Annick D.-D., Frederic D.,"35268044800	25650289200	25649061800	7005396778	,Plants used as remedies antirheumatic and antineuralgic in the traditional medicine of Lebanon",2008,"Journal of Ethnopharmacology","120","3",,"315","334",,146,"10.1016/j.jep.2008.08.024","https://www.scopus.com/inward/record.uri?eid=2-s2.0-56349165179&doi=10.1016%2fj.jep.2008.08.024&partnerID=40&md5=1b578c5d0c5841084454cc4565cf5bb1","Aim of the study: In the present investigation, ethnobotanical and ethnopharmacological research of the traditional use of plants to cure antirheumatic and antineuralgic diseases was carried out in Lebanon. Materials and methods: The information was collected from different cities and villages of the twenty-six districts ""aqdya or qadaa"" of the six governorates ""mohaafazah"" of Lebanon. The knowledge on the medicinal use of plants has been recorded directly on the basis of a detailed survey on herbalists ""Attarin or dabbous"", folk healers, on older experienced people and midwives ""daye"". This research was carried out for 5 years (2002-2007). Results and conclusions: In this survey, 231 species of which 5 are endemic, distributed in 79 botanical families, currently used as antirheumatic herbal remedies, were collected and identified. Among them 53.3% are indigenous, 19.2% are cultivated and 17.5% are imported. Their latin names, local names, local medicinal uses, the used parts of the plant and the preparations and the ways of administration are described. The anti-inflammatory properties due to the plant compounds have been demonstrated and discussed. © 2008 Elsevier Ireland Ltd. All rights reserved.",Article,"Final","",Scopus,2-s2.0-56349165179

Kuva M.A., Pitelli R.A., Alves P.L.C.A., Salgado T.P., Pavani M.C.D.M.,"23094764600	16064607800	7006346326	23095296700	35612283500	,Weed seedbank and its correlation with the established flora in no-burn sugarcane area [Banco de sementes de plantas daninhas e sua correlação com a flora estabelecida no agroecossistema cana-crua]",2008,"Planta Daninha","26","4",,"735","744",,8,"10.1590/s0100-83582008000400004","https://www.scopus.com/inward/record.uri?eid=2-s2.0-58049176697&doi=10.1590%2fs0100-83582008000400004&partnerID=40&md5=52c4051bf5793cb67f4c06eddafaa6a0","To set suitable weed management programs, one the most important parameters is knowing the specific composition of the seedbank and its correlation with the established flora. Such information is fundamental to guide the control strategy to be adopted and the herbicide choices. Mechanical harvest of no-burn sugar-cane maintaining a layer of straw on the soil surface promoted important changes in weed seed germination and seedling survival. Lack of soil mobilization and the lack of sugarcane burning are two additional factors influencing weed flora composition and population densities. Field sampling was conducted in twenty-eight no-burn sugarcane areas to evaluate (i) seedbank composition and seed population densities and later (ii) weed community composition and population density. A predominance of broad-leaf weeds was foud in the seed bank mainly Amaranthus spp., as well as species of the families Euphorbiaceae and Convulvulaceae. The traditional gramineous seeds were not profuse in the seedbanks of the studied areas. The correlation between the seedbank and the weed community compositions was small, regardless of the harvesting time, seedbank quantification methodology and weed species.",Article,"Final","All Open Access, Gold, Green",Scopus,2-s2.0-58049176697

Mazoir N., Benharref A., Bailén M., Reina M., González-Coloma A.,"23035575000	7004179768	14008361100	7005693899	57215378415	,Bioactive triterpene derivatives from latex of two Euphorbia species",2008,"Phytochemistry","69","6",,"1328","1338",,48,"10.1016/j.phytochem.2008.01.004","https://www.scopus.com/inward/record.uri?eid=2-s2.0-41049114971&doi=10.1016%2fj.phytochem.2008.01.004&partnerID=40&md5=c7e5c7f860643937efce5a5b07b193ec","We have investigated the antifeedant and toxic effects of 23 semisynthetic terpenoid derivatives obtained through chemical modifications of the major components of Euphorbia resinifera (α-euphol and α-euphorbol) and E. officinarum (obtusifoliol and 31-norlanostenol) latex on several insect species (Spodoptera littoralis, Myzus persicae and Rhopalosiphum padi), their selective cytotoxicity on insect Sf9 and mammalian CHO cells and their phytotoxic effects on Lactuca sativa. The conversions focused mainly on positions 3, 7, 11, and 24 with several oxidizing agents. A total of 18 compounds affected S. littoralis growth (IGR). Our results support the importance of the C-3 substituent, suggest the involvement of the C-7 substituent and indicate that the C-3 hydroxyl is not essential for the IGR effect. Overall, Sf9 cells were more sensitive to the active compounds than CHO cells. All of these compounds had non selective moderate phytotoxic effects on radicle elongation of L. sativa. © 2008 Elsevier Ltd. All rights reserved.",Article,"Final","",Scopus,2-s2.0-41049114971

López-Rodríguez M., Mazoir N., Daoubi M., Reina M., Benharref A.,"7404254884	23035575000	6602864457	7005693899	7004179768	,1-(1,5-Dimethyl-hexyl)-3a,5b,12a,14a-tetra-methyl-2,3,3a,4,5,5a,5b,11,12, 13,14,14a-dodeca-hydro-1H,12aH-cyclo-penta-[1,2]phenanthro[7,8-b]indole",2007,"Acta Crystallographica Section E: Structure Reports Online","63","12",,"","",,,"10.1107/S1600536807053913","https://www.scopus.com/inward/record.uri?eid=2-s2.0-36849045258&doi=10.1107%2fS1600536807053913&partnerID=40&md5=c8e824239db7e74f96dff7cdd1e1422d","The title compound, C35H51N, is a semi-synthesized derivative obtained from 4,14-dimethyl-5-cholest-8-en-3Β-ol, a major triterpene isolated from Euphorbia officinarum latex. The mol-ecular structure was established by 1H and 13C NMR spectroscopy and confirmed as having the Β stereochemistry at the new C11b stereocenter by single-crystal X-ray diffraction. The planar indole group is folded over the extended tetra-cyclic perhydro-cyclo-penta-nephenanthrene triterpene skeleton. There are no π-π interactions between the indole ring systems. © International Union of Crystallography 2007.",Article,"Final","All Open Access, Green",Scopus,2-s2.0-36849045258

Daoubi M., Marquez N., Mazoir N., Benharref A., Hernández-Galán R., Muñoz E., Collado I.G.,"6602864457	7004180868	23035575000	7004179768	6701466216	7202348306	7005250854	,Isolation of new phenylacetylingol derivatives that reactivate HIV-1 latency and a novel spirotriterpenoid from Euphorbia officinarum latex",2007,"Bioorganic and Medicinal Chemistry","15","13",,"4577","4584",,37,"10.1016/j.bmc.2007.04.009","https://www.scopus.com/inward/record.uri?eid=2-s2.0-34249016432&doi=10.1016%2fj.bmc.2007.04.009&partnerID=40&md5=91194168a0eded8a9031b04bb0b09599","Three new, highly functionalized ingol diterpenes, ingol 7,8,12-triacetate 3-phenylacetate (1), ingol 7,8,12-triacetate 3-(4-methoxyphenyl)acetate (2) and 8-methoxyingol 7,12-diacetate 3-phenylacetate (3), together with the novel spirotriterpene, 3S,4S,5R,7S,9R,14R-3,7-dihydroxy-4,14-dimethyl-7[8 → 9]-Abeo-cholestan-8-one (4), have been isolated from Euphorbia officinarum latex. Structures were established on the basis of their spectroscopic data, including two-dimensional NMR analysis and NOE experiments. The biological effects of 1-3 on cell cycle and HIV-1 gene transcription were analysed in the Jurkat T cell line. Compound 3 induced cell-cycle arrest and HIV-1-LTR promoter activation and could represent a novel lead compound for the development of therapies against HIV-1 latency. © 2007 Elsevier Ltd. All rights reserved.",Article,"Final","",Scopus,2-s2.0-34249016432

De Natale A., Pollio A.,"23570415100	6701466225	,Plants species in the folk medicine of Montecorvino Rovella (inland Campania, Italy)",2007,"Journal of Ethnopharmacology","109","2",,"295","303",,79,"10.1016/j.jep.2006.07.038","https://www.scopus.com/inward/record.uri?eid=2-s2.0-33845607183&doi=10.1016%2fj.jep.2006.07.038&partnerID=40&md5=9a9175cb5fbd89893128f71cd3407a84","An ethnobotanical survey was conducted in the territory of Montecorvino Rovella (MR), inland Campania, Italy. Information on traditional uses of 95 botanical taxa was collected	more than half of which are used to treat a wide range of diseases, ranging from pains of different origins, to skin problems, and diseases of the respiratory system. For each medicinal species, data were collected including the vernacular names, preparation, plant part, and therapeutic use. Twenty-four out of the 57 medicinal plants listed have a therapeutic use not previously reported in Campania and neighbouring regions. Plant uses by Montecorvino inhabitants are compared with those described in medical and botanical literature by Campanian authors from the last three centuries. © 2006 Elsevier Ireland Ltd. All rights reserved.",Article,"Final","",Scopus,2-s2.0-33845607183

Mazoir N., Auhmani A., Daoubi M., Collado I.G., Benharref A.,"23035575000	55920325300	6602864457	7005250854	7004179768	,Hemisynthesis of new triterpene derivatives using oxidation by CrO3 and NaIO4-(RuCl3, 3H2O)",2007,"Synthetic Communications","37","8",,"1289","1299",,6,"10.1080/00397910701226756","https://www.scopus.com/inward/record.uri?eid=2-s2.0-34247173999&doi=10.1080%2f00397910701226756&partnerID=40&md5=d2f03bdcb1217cc2228f2a922f1bd6f3","Oxidation of triterpenes resulting from E. resinifera, E. officinarum, and their derivatives, using chromic anhydride (CrO3) and the system NaIO4-(RuCl3,3H2O), gives carbonyl triterpenic compounds with high chemioselectivity. Copyright © Taylor & Francis Group, LLC.",Article,"Final","",Scopus,2-s2.0-34247173999

Mazoir N., Itto M.Y.A., Artiles M.R., Benharref A.,"23035575000	6603454312	47560918900	7004179768	,4α,14α,dimethyl-5α-cholest-8-en-3-one thiosemicarbazone",2006,"MolBank","2006","6","M504","","",,,"10.3390/M504","https://www.scopus.com/inward/record.uri?eid=2-s2.0-85117963500&doi=10.3390%2fM504&partnerID=40&md5=c4c88cecd62ca1c48137792218236206","The compound 2 was prepared from equimolecular quantity of 1 (1g, 2.43 mmol), derivative triterpene resulting from Euphorbia officinarum1, and thiosemicarbazide2,3 dissolved in ethanol with several drops of conc. H2SO4. The mixture was heated at reflux for 5h, and evaporated under reduced pressure. The residue was purified on silica gel column using hexane: ethyl acetate (90:10) as eluent yielded compound 2 (0.99g, 2.06mmol) in 85% yield. © 2006 MDPI. All rights reserved.",Article,"Final","All Open Access, Gold, Green",Scopus,2-s2.0-85117963500

Mazoir N., Itto M.Y.A., Lassaba E., Benharref A.,"23035575000	6603454312	6602794532	7004179768	,4α,14α,dimethyl-5α-cholest-8-en-3-one thiadiazoline",2006,"MolBank","2006","6","M505","","",,,"10.3390/M505","https://www.scopus.com/inward/record.uri?eid=2-s2.0-85117939325&doi=10.3390%2fM505&partnerID=40&md5=4f6e9f0fa056d42e6ab2dda3e928a0af",[No abstract available],Article,"Final","All Open Access, Gold",Scopus,2-s2.0-85117939325

Boydron-Le Garrec R., Benoit E., Sauviat M.-P., Lewis R.J., Molgó J., Laurent D.,"36657428800	56194177100	7004037929	55457251800	7006666682	7103291399	,Ability of some plant extracts, traditionally used to treat ciguatera fish poisoning, to prevent the in vitro neurotoxicity produced by sodium channel activators",2005,"Toxicon","46","6",,"625","634",,34,"10.1016/j.toxicon.2005.07.002","https://www.scopus.com/inward/record.uri?eid=2-s2.0-26444619403&doi=10.1016%2fj.toxicon.2005.07.002&partnerID=40&md5=f3cabbdbd4b763defd5b740e5a69c36c","The effects of 31 plant extracts, which most are traditionally used to treat ciguatera fish poisoning in the Pacific area, were studied on the cytotoxicity of mouse neuroblastoma cells produced by ouabain, veratridine and/or brevetoxin-3 or Pacific ciguatoxin-1. The cell viability was determined using a quantitative colorimetric method. A marked cytotoxicity of seven of the 31 plant extracts studied, was observed. Despite this, these plant extracts were suspected to contain active compound(s) against the cytotoxicity produced by brevetoxin (2 extracts), brevetoxin, ouabain and/or veratridine (3 extracts), or only against that of ouabain and/or veratridine (2 extracts). Among the 24 plant extracts that exhibited by themselves no cytotoxicity, 22 were active against the effect of brevetoxin or against that of both veratridine and brevetoxin. Similar results were obtained when the seven most active plant extracts were reassayed using ciguatoxin instead of brevetoxin. In conclusion, the present work reports the first activity assessment of some plant extracts, achieved in vitro on a quite large scale. The fact that 27 plant extracts were found to exert, in vitro, a protective effect against the action of ciguatoxin and/or brevetoxin, paves the way for finding new active compounds to treat ciguatera fish poisoning, provided these compounds also reverse the effects of sodium channel activators. © 2005 Elsevier Ltd. All rights reserved.",Article,"Final","",Scopus,2-s2.0-26444619403

Guarrera P.M., Salerno G., Caneva G.,"6507575197	8528461200	6602810117	,Folk phytotherapeutical plants from Maratea area (Basilicata, Italy)",2005,"Journal of Ethnopharmacology","99","3",,"367","378",,71,"10.1016/j.jep.2005.01.039","https://www.scopus.com/inward/record.uri?eid=2-s2.0-20844447633&doi=10.1016%2fj.jep.2005.01.039&partnerID=40&md5=17ee1acd3d535a8cc5a892a343b17512","Field ethnobotanical survey was undertaken for the period of 2002-2003 in the Tyrrhenian part of the Basilicata region of southern Italy. Data of 56 species of plants belonging to 29 families where gathered through interviews	among the species, 47 are used in human therapy, 6 as insect repellents, 15 in veterinary medicine, 1 for its ichthyotoxic properties and 3 for magic therapeutic purposes. The most important findings in ethnomedicine relate to Nasturtium officinale (renal colic, liver diseases), Foeniculum vulgare subsp. piperitum (mouth ulcers), Leopoldia comosa (toothache, headache), Micromeria graeca subsp. graeca (coughs) and Ceterach officinarum (malaria), while in the ethnoveterinary field, we have Pteridium aquilinum (wolf bites) and Spartium junceum (fractures of animal limbs). © 2005 Elsevier Ireland Ltd. All rights reserved.",Article,"Final","",Scopus,2-s2.0-20844447633

Al-Qura'n S.,"8605460600	,Ethnobotanical survey of folk toxic plants in southern part of Jordan",2005,"Toxicon","46","2",,"119","129",,53,"10.1016/j.toxicon.2005.04.010","https://www.scopus.com/inward/record.uri?eid=2-s2.0-22044441360&doi=10.1016%2fj.toxicon.2005.04.010&partnerID=40&md5=95d26243e8722a406369d4036e36c554","Thirteen representative phytogeographical sites in the southern part of Jordan was surveyed to recognize the folk toxic plant wealth by calculating of FL, RPL, and ROP values. One hundred and twenty-five toxic species were recorded in the area of study belonging to 56 plant families that were mentioned by three informants or more, 53 toxic species (42.4%) were popular, since they were cited by more than half of the maximum number of informants which is 30	therefore have Rank-Order Priority (ROP) value 50 or more, while 72 species (57.6%) were unpopular, since they were cited by less than half of the maximum number of informants	therefore have ROP value less than 50. Eighty species (63.5%) have known toxicity in neighboring countries while 46 species (36.5%) have not such known toxicity. Major toxic effects cited by popular species have exhibited major symptoms ranging from skin irritation, gastric and abdominal disturbances, abortion, sterility, neuralgic pains including hysteria, and fatigue. The studied area has been shown a high level of toxic species diversity, since it is dominated by at least four phytogeographical elements, which requires certain ecological awareness to protect and reserve the wild and endemic species from further threats to enhance the sustainable development. © 2005 Elsevier Ltd. All rights reserved.",Review,"Final","",Scopus,2-s2.0-22044441360

Fraunfelder F.W.,"7004695504	,Ocular side effects from herbal medicines and nutritional supplements",2004,"American Journal of Ophthalmology","138","4",,"639","647",,57,"10.1016/j.ajo.2004.04.072","https://www.scopus.com/inward/record.uri?eid=2-s2.0-7044253250&doi=10.1016%2fj.ajo.2004.04.072&partnerID=40&md5=869bfc3bf2be498bf44ae094e4414f57","To review the more significant herbal and nutritional agents of clinical importance to ophthalmologists and describe the ocular side effects for each. World Health Organization (WHO) classification and guidelines for clinicians are provided. Retrospective observational case series. A retrospective observational case series of reports of ocular side effects or systemic side effects from medications used for the eye from herbal medicines and nutritional supplements. Cases were collected from spontaneous reports submitted to the WHO, the Food and Drug Administration, and the National Registry of Drug-Induced Ocular Side Effects. A review of the world's literature was performed to obtain additional case reports and insight into adverse ocular reactions. Data were collected on age, gender, duration of therapy, concomitant medications, dosage, and dechallenge and rechallenge results. The National Registry of Drug-Induced Ocular Side Effects received 263 spontaneous reports, in addition to 60 case reports from the literature. Canthaxanthine, chamomile, Datura, Echinacea purpurea, Ginkgo biloba, licorice, niacin, and vitamin A are all associated with clinically significant ocular side effects. Herbal medicines and nutritional supplements can cause ocular side effects. Clinicians need to recognize these adverse events, because a large segment of the population uses them, many times without the treating physician's knowledge. © 2004 by Elsevier Inc. All rights reserved.",Article,"Final","",Scopus,2-s2.0-7044253250

Daoubi M., Benharref A., Hernandez-Galán R., Macías-Sánchez A.J., Collado I.G.,"6602864457	7004179768	6701466216	6602774755	7005250854	,Two novel steroid from Euphorbia officinarum latex",2004,"Natural Product Research","18","2",,"177","181",,12,"10.1080/14786410310001608118","https://www.scopus.com/inward/record.uri?eid=2-s2.0-1142303736&doi=10.1080%2f14786410310001608118&partnerID=40&md5=7cb48528b58e5038c39768fca75c7c88","Two novel steroids, 3β,7α-dihydroxy-4α,14α-dimethyl-5α-cholest-8-en-11 -one (2) and 3β,7β-dihydroxy-4α,14α-dimethyl-5α-cholest-8-en-11-o ne (3) were isolated from the latex of Euphorbia officinarwn. Their structures were established on the basis of NMR and MS studies.",Article,"Final","",Scopus,2-s2.0-1142303736

Pfündel E., Nagel E., Meister A.,"6603945249	36776254100	7101954277	,Analyzing the light energy distribution in the photosynthetic apparatus of C4 plants using highly purified mesophyll and bundle-sheath thylakoids",1996,"Plant Physiology","112","3",,"1055","1070",,22,"10.1104/pp.112.3.1055","https://www.scopus.com/inward/record.uri?eid=2-s2.0-0029774918&doi=10.1104%2fpp.112.3.1055&partnerID=40&md5=03357efae65efc9f0c16d85671f76fbc","The chlorophyll fluorescence characteristics of mesophyll and bundle- sheath thylakoids from plant species with the C4 dicarboxylic acid pathway of photosynthesis were investigated using flow cytometry. Ten species with the NADP-malic enzyme (NADP-ME) biochemical type of C4 photosynthesis were tested: Digitaria sanguinalis (L.) Scop., Euphorbia maculata L., Portulaca grandiflora Hooker, Saccharum officinarum L., Setaria viridis (L.) Beauv., Zea mays L., and four species of the genus Flaveria. This study also included three species with NAD-ME biochemistry (Atriplex rosea L., Atriplex spongiosa F. Muell., and Portulaca oleracea L.). Two C4 species of unknown biochemical type were investigated: Cyperus papyrus L. and Atriplex tatarica L. Pure mesophyll and bundle-sheath thylakoids were prepared by flow cytometry and characterized by low-temperature fluorescence spectroscopy. In pure bundle-sheath thylakoids from many species with C4 photosynthesis of the NADP-ME type, significant amounts of photosystem II (PSII) emission can be detected by fluorescence spectroscopy. Simulation of fluorescence excitation spectra of these thylakoids showed that PSII light absorption contributes significantly to the apparent excitation spectrum of photosystem I. Model calculations indicated that the excitation energy of PSII is efficiently transferred to photosystem I in bundle-sheath thylakoids of many NADP-ME species.",Article,"Final","All Open Access, Bronze, Green",Scopus,2-s2.0-0029774918

Peltier J.P., anda F.,"7006667955	57190931071	,Diversité et originalité de la steppe à Euphorbia officinarum L. subsp. echinus (HOOKER fil. et COSSON) VINDT du sud‐ouest Marocain",1995,"Feddes Repertorium","106","3-4",,"215","229",,6,"10.1002/fedr.4921060316","https://www.scopus.com/inward/record.uri?eid=2-s2.0-85047691710&doi=10.1002%2ffedr.4921060316&partnerID=40&md5=99d461a95d3518fc1e7c8b208af83aab","La steppe à «euphorbe oursin» couvre au Maroc une bande cǒtière qui englobe le sud du Souss et une partie du Sahara atlantique, région sous l'influence de l'alizé maritime. On peut y reconnaître six associations dominées, dans le nord, par l'arganier et dans le sud, par les Chénopodiacées sahariennes. La steppe est caractérisée par la présence simultanée d'espèces de souche tropicale, saharienne et méditerranéenne. Elle peut être considérée comme une des formes océaniques du thermoméditerranéen. Profondément modifiée par l'action de l'homme, sa conservation est aujourd'hui compromise. Copyright © 1995 Wiley‐VCH",Article,"Final","",Scopus,2-s2.0-85047691710

**Database Web of sciences**

Publication Type	Authors	Book Authors	Book Editors	Book Group Authors	Author Full Names	Book Author Full Names	Group Authors	Article Title	Source Title	Book Series Title	Book Series Subtitle	Language	Document Type	Conference Title	Conference Date	Conference Location	Conference Sponsor	Conference Host	Author Keywords	Keywords Plus	Abstract	Addresses	Reprint Addresses	Email Addresses	Researcher Ids	ORCIDs	Funding Orgs	Funding Text	Cited References	Cited Reference Count	Times Cited, WoS Core	Times Cited, All Databases	180 Day Usage Count	Since 2013 Usage Count	Publisher	Publisher City	Publisher Address	ISSN	eISSN	ISBN	Journal Abbreviation	Journal ISO Abbreviation	Publication Date	Publication Year	Volume	Issue	Part Number	Supplement	Special Issue	Meeting Abstract	Start Page	End Page	Article Number	DOI	Book DOI	Early Access Date	Number of Pages	WoS Categories	Research Areas	IDS Number	UT (Unique WOS ID)	Pubmed Id	Open Access Designations	Highly Cited Status	Hot Paper Status	Date of Export

J	Bailen, M; Khamlichi, MD; Benharref, A; Martinez-Diaz, RA; Gonzalez-Coloma, A	Bailen, Maria; Khamlichi, Mourad Daoubi; Benharref, Ahmed; Martinez-Diaz, Rafael A.; Gonzalez-Coloma, Azucena	New Bioactive Semisynthetic Derivatives of 31-Norlanostenol and Obtusifoliol from Euphorbia officinarum	NATURAL PRODUCT COMMUNICATIONS	Fifteen semisynthetic terpenoid derivatives from the major latex components of Euphorbia officinarum have been evaluated against the insect pest Spodoptera littoralis, two species of protozoan parasites, Trypanosoma cruzi and Leishmania infantum, and also against insect Sf9 and mammalian CHO cells to test their selective cytotoxicity. Our results showed that 40% of the test substances were postingestive toxicants to S. littoralis. All the tested derivatives had cytotoxic effects on insect-derived Sf9 cells, whereas mammalian CHO cells were affected by a lower number of compounds (47%). Furthermore, 87% of the test compounds had antiparasitic effects on both L. infantum and T. cruzi, with some of them being selective parasite toxicants.	Martinez-Diaz, Rafael A/K-5926-2014; Bailen, Maria/ABF-9684-2020; Colonna, Azucena Gonzalez/G-8781-2015	Martinez-Diaz, Rafael A/0000-0002-4203-4845; Colonna, Azucena Gonzalez/0000-0001-5124-664X; Bailen, Maria/0000-0001-5201-906X	1934-578X	1555-9475	JUN	2016	11	6	733	738	WOS:000379631600009	27534104

J	Smaili, A; Mazoir, N; Rifai, LA; Koussa, T; Makroum, K; Benharref, A; Faize, L; Alburquerque, N; Burgos, L; Belfaiza, M; Faize, M	Smaili, Amal; Mazoir, Noureddine; Aicha Rifai, Lalla; Koussa, Tayeb; Makroum, Kacem; Benharref, Ahmed; Faize, Lydia; Alburquerque, Nuria; Burgos, Lorenzo; Belfaiza, Malika; Faize, Mohamed	Antimicrobial Activity of two Semisynthetic Triterpene Derivatives from Euphorbia officinarum Latex against Fungal and Bacterial Phytopathogens	NATURAL PRODUCT COMMUNICATIONS	This study evaluated the in vitro antimicrobial effect of 3 beta-acetoxy-norlup-20-one (1) and 3-chloro-4 alpha,14 alpha-dimethy1-5 alpha-cholest-8-ene (2), triterpene derivatives from Euphorbia officinarum latex against fungal and bacterial phytopathogens. Results showed that although mycelial growth of several strains of Verticillium dahlia, and Fusarium oxysporum fsp. melonis and Penicillium expansum was affected only moderately, the two compounds were able to reduce highly conidia formation and germination, suggesting that they act as fungistatic compounds. Their antibacterial activity was tested against Pseudomonas syringae pv. syringae (Pss), P. syringae pv. tabacci (Pst), Erwinia amylovora (Ea) and Agrobacterizun tumefaciens (At) using disc diffusion method. Results showed that compound 2 was more effective in inhibiting the growth of Pss, Pst and Ea than compound 1.	Burgos, Lorenzo/B-4353-2008	Burgos, Lorenzo/0000-0003-2399-8232; FAIZE nee BREMAUD, Lydia/0000-0002-1999-6034	1934-578X	1555-9475	MAR	2017	12	3	331	336	WOS:000398017400005	30549878

J	Smaili, A; Rifai, LA; Mazoir, N; Koussa, T; Faize, L; Alburquerque, N; Burgos, L; Makroum, K; Malika, B; Benharref, A; Faize, M	Smaili, Amal; Aicha Rifai, Lalla; Mazoir, Noureddine; Koussa, Tayeb; Faize, Lydia; Alburquerque, Nuria; Burgos, Lorenzo; Makroum, Kacem; Malika, Belfaiza; Benharref, Ahmed; Faize, Mohamed	Semisynthetic Triterpenes Derived from Euphorbia officinarum as Plant Growth Promoters and Inducers of Disease Resistance	JOURNAL OF PLANT GROWTH REGULATION	This study evaluated the effect of application of the semisynthetic triterpenes 3 beta-acetoxy-norlup-20-one (F4) and 3-chloro-4 alpha,14 alpha-dimethyl-5 alpha-cholest-8-ene (F6) triterpene derivatives from Euphorbia officinarum on the growth of tomato seedlings under normal conditions and when challenged with the pathogens Verticillium dahliae and Agrobacterium tumefaciens. Foliar spray of F4 and F6 significantly improved growth rate, fresh weight, dry weight, and leaf area. In addition, they enhanced several physiological parameters including photosynthetic pigments, proline content, and nitrate reductase activity. Moreover, they induced H2O2 accumulation and increased the activity of several antioxidant enzymes such as catalase, ascorbate peroxidase, and guaiacol peroxidase. They also enhanced disease resistance against V. dahliae and A. tumefaciens. These results suggest that the two semisynthetic triterpenes represent new plant growth regulators and inducers of plant disease resistance.	Faize, Mohamed/0000-0001-5718-1787	0721-7595	1435-8107	MAR	2019	38	1	262	272	10.1007/s00344-018-9838-3	WOS:000464024400024

J	Daoubi, M; Benharref, A; Hernandez-Galan, R; Macias-Sanchez, AJ; Collado, IG	Daoubi, M; Benharref, A; Hernandez-Galan, R; Macias-Sanchez, AJ; Collado, IG	Two novel steroids from Euphorbia officinarum latex	NATURAL PRODUCT RESEARCH	Two novel steroids, 3beta, 7alpha-dihydroxy-4alpha, 14alpha-dimethyl-5alpha-cholest-8-en-11-one (2) and 3beta,7beta-dihydroxy-4 alpha, 14alpha-dimethyl-5alpha-cholest-8-en-11-one (3) were isolated from the latex of Euphorbia officinarum. Their structures were established on the basis of NMR and MS studies.	Hernandez-Galan, Rosario/B-7697-2009; Collado, Isidro G. Gonzalez/G-5501-2015; Sanchez, Antonio Jose Macias/AAM-8503-2020	Collado, Isidro G. Gonzalez/0000-0002-8612-0593; Sanchez, Antonio Jose Macias/0000-0001-6002-4977; Hernandez Galan, Rosario/0000-0003-1887-4796	1478-6419	APR	2004	18	2	177	181	10.1080/14786410310001608118	WOS:000186616700014	14984093

J	Daoubi, M; Marquez, N; Mazoir, N; Benharref, A; Hernandez-Galan, R; Munoz, E; Collado, IG	Daoubi, Mourad; Marquez, Nieves; Mazoir, Noureddine; Benharref, Ahmed; Hernandez-Galan, Rosario; Munoz, Eduardo; Collado, Isidro G.	Isolation of new phenylacetylingol derivatives that reactivate HIV-1 latency and a novel spirotriterpenoid from Euphorbia officinarum latex	BIOORGANIC & MEDICINAL CHEMISTRY	Three new, highly functionalized ingol diterpenes, ingol 7,8,12-triacetate 3-phenylacetate (1), ingol 7,8,12-triacetate 3-(4methoxyphenyl)acetate (2) and 8-methoxyingol 7,12-diacetate 3-phenylacetate (3), together with the novel spirotriterpene, 3S,4S,5R,7S,9R,14R-3,7-dihydroxy-4,14-dimethyl-7[8 9]-Abeo-cholestan-8-one (4), have been isolated from Euphorbia officinarum latex. Structures were established on the basis of their spectroscopic data, including two-dimensional NMR analysis and NOE experiments. The biological effects of 1-3 on cell cycle and HIV-1 gene transcription were analysed in the Jurkat T cell line. Compound 3 induced cell-cycle arrest and HIV-1-LTR promoter activation and could represent a novel lead compound for the development of therapies against HIV-1 latency. (c) 2007 Elsevier Ltd. All rights reserved.	Collado, Isidro G. Gonzalez/G-5501-2015; Munoz, Eduardo/I-5225-2012; Hernandez-Galan, Rosario/B-7697-2009	Collado, Isidro G. Gonzalez/0000-0002-8612-0593; Munoz, Eduardo/0000-0001-8478-5842; noureddine, mazoir/0000-0002-6359-1739; Hernandez Galan, Rosario/0000-0003-1887-4796	0968-0896	1464-3391	JUL 1	2007	15	13	4577	4584	10.1016/j.bmc.2007.04.009	WOS:000247360400022	17481906

J	Terrab, A; Marconi, A; Bettar, I; Msanda, F; Diez, MJ	Terrab, Anass; Marconi, Alfredo; Bettar, Ismail; Msanda, Fouad; Josefa Diez, Maria	Palynological characterisation of Euphorbia honeys from Morocco	PALYNOLOGY	Pollen was analysed in 30 unifloral Euphorbia honey samples from the Ifni Massif Region (Anti Atlas, Morocco). The honey samples were directly provided by beekeepers. The quantitative analyis showed that nectar is the main honey source in the samples studied. The qualitative analysis of the samples showed the presence of 35 taxa belonging to 17 families. The Moroccan Euphorbia honeys of the studied region are characterized by their low-medium number of pollen grains (NGP; mean = 5700), 76% of the honeys belong to Class I and II of Maurizio, and by their low honeydew indicator (HDE/NGPn < 0.28), wich indicates their floral origin. For the Euphorbia officinarum subsp. echinus honeys, the most characteristic accompanying species are Eryngium ilicifolium, present in > 90% of the samples, followed by Bellis sp., Capsella f. and Reseda sp. (85%). However, for the E. regis-jubae honeys, the most characteristic accompanying species are Crepis f., present in 100% of the samples.	Terrab, Anass/F-1490-2016	Terrab, Anass/0000-0002-7979-3669; Diez Dapena, Maria Josefa/0000-0001-7857-3085	0191-6122	1558-9188	JAN 2	2014	38	1	138	146	10.1080/01916122.2013.871797	WOS:000334827700010

J	Bettar, I; Gonzalez-Miret, ML; Hernanz, D; Marconi, A; Heredia, FJ; Terrab, A	Bettar, Ismail; Lourdes Gonzalez-Miret, M.; Hernanz, Dolores; Marconi, Alfredo; Heredia, Francisco J.; Terrab, Anass	Characterisation of Moroccan Spurge (Euphorbia) honeys by their physicochemical characteristics, mineral contents and colour	ARABIAN JOURNAL OF CHEMISTRY	The quality of 27 Moroccan Spurge (Euphorbia) honey samples was assessed. Eight physicochemical parameters and mineral composition were analysed and the CIELAB colour parameters (L*, a*, b*, C-ab* and h(ab)) were determined. Results show no significant differences between the two Euphorbia honey types (Euphorbia officinarum subsp. echinus and Euphorbia regis-jubae honeys) regarding the physicochemical parameters. Sodium and magnesium show average values that can help to differentiate between E. officinarum subsp. echinus and E. regis-jubae honeys. Potassium was quantitatively the most important mineral (66% of the total minerals quantified), while sodium and calcium were present in moderate amounts (20% and 11% of the minerals, respectively). The colour parameters also have shown significant differences between E. officinarum subsp. echinus and E. regis-jubae honeys. Considering the total information from physicochemical, mineral and colour data, Principal Component Analysis (PCA) and Stepwise Discriminant Analysis (SDA) were carried out to distinguish between the two Euphorbia honey types. PCA showed that the cumulative variance was approximately 56%. The results of SDA showed that parameters with a higher discriminant power were Na, Mg, L*, C-ab* and h(ab), and almost 100% of the samples were properly classified in their corresponding group, except for one sample. (C) 2019 Published by Elsevier B.V. on behalf of King Saud University. This is an open access article under the CC BY-NC-ND license (http://creativecommons.org/licenses/by-nc-nd/4.0/).	Hernanz, Dolores/L-1348-2014	Hernanz, Dolores/0000-0001-9737-8816	1878-5352	1878-5379	DEC	2019	12	8	2052	2060	10.1016/j.arabjc.2015.01.003	WOS:000504900300026

J	Smaili, A; Mazoir, N; Rifai, LA; Koussa, T; Makroum, K; Kabil, E; Benharref, A; Faize, M	Smaili, Amal; Mazoir, Noureddine; Rifai, Lalla Aicha; Koussa, Tayeb; Makroum, Kacem; Kabil, El Mostafa; Benharref, Ahmed; Faize, Mohamed	Triterpene derivatives from Euphorbia enhance resistance against Verticillium wilt of tomato	PHYTOCHEMISTRY	Oxidation of alpha-euphorbol and 31-norlanostenol, two triterpenic compounds isolated from the latex of Euphorbia resinifera and Euphorbia officinarum respectively, yielded four products named 3 beta-tosyloxy-4 alpha,14 alpha-dimethyl-5 alpha-cholesta-7,9-diene; 4 alpha,14 alpha-dimethyl-5 alpha-cholesta-7,9-dien-3 beta-ol; 24-methylenelemo-lanosta-8,24-dien-3-one and elemo-lanost-8-en-3,11,24-trione. They were evaluated for protection of tomato plants against Verticillium dahliae in a greenhouse. The four semisynthesized products were phytotoxic at higher concentrations as they completely inhibited tomato germination at 100 and 500 mu g/ml. However at lower concentrations (10 and 50 mu g/ml) germination and root length were not affected. Disease resistance against Verticillium wilt was assessed in tomato plants derived from seeds that germinated in the presence of 10 and 50 mu g/ml of the four products. All of them were able to reduce significantly disease severity, with 10 mu g/ml being more effective than 50 mu g/ml. Reduction of leaf alteration index and of stunting index ranged from 52 to 68% and from 43 to 67%, respectively, while vessel discoloration was reduced by at least 95%. The compounds were also able to elicit H2O2 accumulation before and after fungal inoculation and to significantly enhance peroxidase and polyphenol oxidase activities. These results suggest that the hemisynthetized triterpenes can be used as elicitors of disease resistance. (C) 2016 Elsevier Ltd. All rights reserved.	Faize, Mohamed/0000-0001-5718-1787	0031-9422	MAR	2017	135	169	180	10.1016/j.phytochem.2016.12.017	WOS:000394197400017	28027775

J	Mazoir, N; Benharref, A; Bailen, M; Reina, M; Gonzalez-Coloma, A	Mazoir, Noureddine; Benharref, Ahmed; Bailen, Maria; Reina, Matias; Gonzalez-Coloma, Azucena	Bioactive triterpene derivatives from latex of two Euphorbia species	PHYTOCHEMISTRY	We have investigated the antifeedant and toxic effects of 23 semisynthetic terpenoid derivatives obtained through chemical modifications of the major components of Euphorbia resinifera (alpha-euphol and alpha-euphorbol) and E. officinarum (obtusifoliol and 31-norlanostenol) latex on several insect species (Spodoptera littoralis, Myzus persicae and Rhopalosiphum padi), their selective cytotoxicity on insect Sf9 and mammalian CHO cells and their phytotoxic effects on Lactuca sativa. The conversions focused mainly on positions 3,7, 11, and 24 with several oxidizing agents. A total of 18 compounds affected S. littoralis growth (IGR). Our results support the importance of the C-3 substituent, suggest the involvement of the C-7 substituent and indicate that the C-3 hydroxyl is not essential for the IGR effect. Overall, Sf9 cells were more sensitive to the active compounds than CHO cells. All of these compounds had non selective moderate phytotoxic effects on radicle elongation of L. sativa. (C) 2008 Elsevier Ltd. All rights reserved.	Bailen, Maria/ABF-9684-2020; Colonna, Azucena Gonzalez/G-8781-2015	Colonna, Azucena Gonzalez/0000-0001-5124-664X; noureddine, mazoir/0000-0002-6359-1739; Bailen, Maria/0000-0001-5201-906X	0031-9422	APR	2008	69	6	1328	1338	10.1016/j.phytochem.2008.01.004	WOS:000255669000005	18304594

J	Smaili, A; Mazoir, N; Rifai, LA; Koussa, T; Makroum, K; Belfaiza, M; Benharref, A; Faize, M	Smaili, Amal; Mazoir, Noureddine; Rifai, Lalla Aicha; Koussa, Tayeb; Makroum, Kacem; Belfaiza, Malika; Benharref, Ahmed; Faize, Mohamed	Induced resistance to wild fire disease of Nicotiana benthamiana using seed treated with triterpene derivatives from Euphorbia	JOURNAL OF PLANT PATHOLOGY	In this study, an approach based on seed treatment was undertaken to enhance resistance of Nicotiana benthamiana against wild fire disease caused by Pseudomonas syringae pv. tabaci. Seeds soaked in 10 mu g ml(-1) of three hemi-synthetic triterpenes (F3, F4 and F6) derived from the latex of Euphorbia officinarum and Euphorbia resinifera resulted in plants with reduced disease severity, which was correlated with a decrease of bacterial populations in planta. Analysis of plant defense markers revealed that H2O2 and guaiacol peroxidase were only slightly activated but were primed after pathogen infiltration. However, polyphenol oxidase, catalase and ascorbate peroxidase were directly induced by the triterpenic derivatives. These results underline the duality of the mode of action of the triterpenic compounds and suggest their use as plant defense inducers in crop protection.	Faize, Mohamed/0000-0001-5718-1787	1125-4653	APR	2018	100	1	75	83	10.1007/s42161-018-0031-0	WOS:000431345400009

J	Mazoir, N; Benharref, A; Bailen, M; Reina, M; Gonzalez-Coloma, A; Martinez-Diaz, RA	Mazoir, Noureddine; Benharref, Ahmed; Bailen, Maria; Reina, Matias; Gonzalez-Coloma, Azucena; Martinez-Diaz, Rafael A.	Antileishmanial and Antitrypanosomal Activity of Triterpene Derivatives from Latex of Two Euphorbia Species	ZEITSCHRIFT FUR NATURFORSCHUNG SECTION C-A JOURNAL OF BIOSCIENCES	The in vitro activity on Leishmania infantum promastigotes and Trypanosoma cruzi epimastigotes of 25 semisynthetic terpenoid derivatives has been evaluated. These compounds were obtained through chemical modifications of the major components of Euphorbia res-inifera (alpha-euphol and alpha-euphorbol) and Euphorhia officinarum (obtusifoliol and 31-norianosterol). Leishmaniasis and Chagas disease are major worldwide health problems. The drugs of choice for their treatment are still problematic in both cases, and therefore there is an urgent need to discover new drugs with high activity and low side effects. Natural products have become a key source of new drugs in the last years. The genus Euphorbia has been the subject of abundant phytochemical and pharmacological research because of its potential medical applications, but the antiparasitic effects of derivatives from plants of this genus are still unknown. Our results showed that 76% and 64% of the test compounds had antiparasitic effects on L. infantum and T cruzi, respectively. The different activities on both parasites, especially their moderate effects on mammalian cells, indicate an interesting selective toxicity.	Martinez-Diaz, Rafael A/K-5926-2014; Bailen, Maria/ABF-9684-2020; Colonna, Azucena Gonzalez/G-8781-2015	Martinez-Diaz, Rafael A/0000-0002-4203-4845; Colonna, Azucena Gonzalez/0000-0001-5124-664X; Bailen, Maria/0000-0001-5201-906X; noureddine, mazoir/0000-0002-6359-1739	0939-5075	1865-7125	JUL-AUG	2011	66	7-8	360	366	10.5560/ZNC.2011.66c0360	WOS:000295498600007	21950160

J	Correia, NM; Kronka, B	Correia, N. M.; Kronka Jr, B.	Efficacy of Herbicides Applied During the Dry and Wet Seasons for Euphorbia heterophylla Control in Sugarcane	PLANTA DANINHA	An experiment was carried out in a commercial sugarcane plantation area in Jaboticabal, Sao Paulo, Brazil, from August 2008 to June 2009 to evaluate the effect of herbicides applied in preemergence and postemergence, alone and combination, during the dry and wet seasons, for poinsettia control (Euphorbia heterophylla). The experiment was arranged in a randomized block design in split-plot, and four replications. Three herbicide treatments [amicarbazone (1.4 kg ha(-1)), imazapic (0.147 kg ha(-1)) and sulfentrazone (0.9 kg ha(-1) plus 0.2 kg ha(-1)), applied on August 01, 2008 after sugarcane harvest, and one treatment without herbicide were tested. The herbicides used during the wet season were: mesotrione alone (0.192 kg ha(-1)), and in mixture (0.12 kg ha(-1)) with ametryn (1.5 kg ha(-1)), atrazine (1.5 kg ha(-1)) or diuron plus hexazinone (0.702 plus 0.198 kg ha(-1)), applied in postemergence on November 14, 2008, and two treatments without herbicide. The application of imazapic during the dry season was efficient for E. heterophylla control, without the need of management complementation during the wet season. However, the amicarbazone and sulfentrazone treatments required mesotrione application, alone or mixed with ametryn, atrazine or diuron, plus hexazinone to control E. heterophylla during the wet season (105 to 230 days after application during the dry season). Without herbicide application during the dry season, mesotrione mixed with ametryn, atrazine or diuron plus hexazinone resulted in a better weed control than when applied alone. Despite the visual symptoms of phytointoxication caused by the treatments mesotrione plus ametryn and mesotrione plus (diuron plus hexazinone), none of the types of management adopted or combinations between them interfered in the number of viable stalk by linear meter, and in stalk diameter and height in sugarcane.	0100-8358	1806-9681	OCT-DEC	2010	28	4	853	863	10.1590/S0100-83582010000400019	WOS:000286108700019

J	Terrab, A; Moujanni, A; Essamadi, AK; Hernanz, D; Diez, MJ; Berjano, R	Terrab, Anass; Moujanni, Abdelkarim; Khalid Essamadi, Abdel; Hernanz, Dolores; Josefa Diez, Maria; Berjano, Regina	A palynological and geographical characterization of labeled resin spurge honey: Euphorbia resinifera	PALYNOLOGY	Unifloral honeys are highly valued, and their botanical and geographical characterization plays an important role in quality control. This work is an essential contribution to the limited knowledge of the unique resin spurge (Euphorbia resinifera) honey. Palynological and geographical characterization was performed on 29 unifloral resin spurge honey samples distributed along the protected geographic indication (PGI) area in the Middle Atlas Mountains (Morocco). The quantitative pollen analysis shows that resin spurge honeys were characterized by its low to very low pollen content (mean NPG = 1490/g honey) and honeydew indicator elements (mean HDE = 28/g honey). The most characteristic accompanying species are Ceratonia siliqua, Echium plantagineum, Olea sp., Plantago coronopus and Quercus. The proposed markers could further contribute as a useful tool to the quality control of the unique resin spurge honey and thus, guarantee its commercial value, both in the local and international markets. It also allows us to distinguish them from other unifloral honeys from highly related botanical origin, such E. officinarum subsp. echinus and E. regis-jubae honeys. The present characterization will allow further strengthen and protect the reputation of this honey especially since it is being registered in the EU list of protected products. Lastly, considering the lower percentage of E. resinifera pollen found in most of our resin spurge honey samples, we encourage for a revision of the established threshold and propose to lower it to 20% of pollen of E. resinifera to be considered as unifloral honey.	Terrab, Anass/F-1490-2016	Terrab, Anass/0000-0002-7979-3669	0191-6122	1558-9188	10.1080/01916122.2021.1933639	MAY 2021	WOS:000666902900001

J	Mazoir, N; Auhmani, A; Daoubi, M; Benharref, A	Mazoir, N.; Auhmani, A.; Daoubi, M.; Benharref, A.	Hemisynthesis of new triterpene derivatives using oxidation by CrO3 and NaIO4-(RuCl3, 3H(2)O)	SYNTHETIC COMMUNICATIONS	Oxidation of triterpenes resulting from E. resinifera, E. officinarum, and their derivatives, using chromic anhydride (CrO3) and the system NaIO4- (RuCl3,3H(2)O), gives carbonyl triterpenic compounds with high chemioselectivity.	Collado, Isidro G. Gonzalez/G-5501-2015	Collado, Isidro G. Gonzalez/0000-0002-8612-0593; noureddine, mazoir/0000-0002-6359-1739	0039-7911	2007	37	7-9	1289	1299	10.1080/00397910701226756	WOS:000247620500027

J	Mazoir, N; Itto, MYA; Lassaba, E; Benharref, A	Mazoir, Noureddine; Itto, My Youssef Ait; Lassaba, Essediya; Benharref, Ahmed	4 alpha,14 alpha,Dimethyl-5 alpha-cholest-8-en-3-one thiadiazoline	MOLBANK	ITTO, Moulay Youssef AIT/R-4780-2019	1422-8599	DEC	2006	6	M505	10.3390/M505	WOS:000415460400002

J	Mazoir, N; Itto, MYA; Artiles, MR; Benharref, A	Mazoir, Noureddine; Itto, My Youssef Ait; Artiles, Matias Reina; Benharref, Ahmed	4 alpha,14 alpha,Dimethyl-5 alpha-cholest-8-en-3-one thiosemicarbazone	MOLBANK	ITTO, Moulay Youssef AIT/R-4780-2019	1422-8599	DEC	2006	6	M504	10.3390/M504	WOS:000415460400001

J	Lopez-Rodriguez, M; Mazoir, N; Daoubi, M; Reina, M; Benharref, A	Lopez-Rodriguez, Matias; Mazoir, Noureddine; Daoubi, Mourad; Reina, Matias; Benharref, Ahmed	1-(1,5-dimethylhexyl)-3a,5b,12a,14a-tetramethyl-2,3,3a,4,5,5a,5b,11,12,13,14,14a-dodecahydro-1H,12aH-cyclopenta[1,2] phenanthro[7,8-b]indole	ACTA CRYSTALLOGRAPHICA SECTION E-CRYSTALLOGRAPHIC COMMUNICATIONS	The title compound, C35H51N, is a semi-synthesized derivative obtained from 4 alpha,14 alpha-dimethyl-5 alpha-cholest-8-en-3 beta-ol, a major triterpene isolated from Euphorbia officinarum latex. The molecular structure was established by H-1 and C-13 NMR spectroscopy and confirmed as having the beta stereochemistry at the new C11b stereocenter by single-crystal X-ray diffraction. The planar indole group is folded over the extended tetracyclic perhydrocyclopentanephenanthrene triterpene skeleton. There are no pi-pi interactions between the indole ring systems.	noureddine, mazoir/0000-0002-6359-1739	2056-9890	DEC	2007	63	12	O4911	U6983	10.1107/S1600536807053913	WOS:000251282300651

J	Cabrera, DC; Chaila, S; Sobrero, MT; Varela, AE	Cabrera, D. C.; Chaila, S.; Sobrero, M. T.; Varela, A. E.	PHYTOSOCIOLOGICAL SURVEY OF SUGARCANE CROP WEEDS IN DIFFERENT AGROECOLOGICAL AREAS IN TUCUMAN PROVINCE, ARGENTINA	PLANTA DANINHA	Weeds constitute the main constraint on the productivity of sugarcane agroecosystems in Argentina. This work aimed to analyze the floristic and phytosociological composition of sugarcane weed communities in different agroecological areas in Tucuman. Fifteen plots from each area were sampled 60 to 90 days after crop shooting, by throwing a 0.5 m x 0.5 m quadrat metallic frame six times in random directions in each of the plots. Species found within the frame were identified. Later, fresh and dry biomass weight were calculated per quadrat sample and species. Frequency, density, dominance, importance value index, Shannon-Wiener and Simpson species diversity index and Jaccard similarity index were calculated. The unweighted pair group method with arithmetic mean (UPGMA) was used to interpret similarity. Overall, 35 species were identified, 24 of which were dicotyledons and 11 monocotyledons. Eighteen of them were annual species, versus 17 which were perennial. Considering M, the most outstanding families were Poaceae, Cyperaceae, Euphorbiaceae, Amaranthaceae and Asteraceae. The results obtained in all the areas revealed the importance of the species Panicum maximum, Sorghum halepense, Cyperus rotundus, Cynodon dactylon and Euphorbia hyssopifolia. The areas with greater and less diversity were the Depressed Plain-Non-Saline Depressed Plain Subregion and the Subhumid-Humid Chaco-Pampean Plain, respectively. There was a 53% similarity between the Depressed Plain-Saline Depressed Plain and the Depressed Plain-Non-Saline Depressed Plain, and a 47% similarity between the Humid and Perhumid Foothills and the Subhumid-Humid Chaco-Pampean Plain. Hierarchical clustering and Jaccard index led to similar results.	0100-8358	1806-9681	2019	37	e019179380	10.1590/S0100-83582019370100027	WOS:000467799400001

J	Giraldeli, AL; San Gregorio, JPR; Monquero, PA; Aguillera, MM; Ribeiro, NM	Giraldeli, A. L.; San Gregorio, J. P. R.; Monquero, P. A.; Aguillera, M. M.; Ribeiro, N. M.	WEEDS HOSTS OF NEMATODES IN SUGARCANE CULTURE	PLANTA DANINHA	The presence of weeds and nematodes can affect of sugarcane yield. This research evaluated whether weeds that are very frequent in mechanized harvested sugarcane can be hosts for root-knot nematodes: Meloidogyne incognita or Meloidogyne javanica. Regarding nematode hospitability, ten weed species as well as a control sample (tomato) were evaluated; they were inoculated with M. incognita and M. javanica. The plants were inoculated with 2,000 eggs and second-stage juveniles (J(2)), and there were three plants per experimental unit (6,000 eggs and J(2) per pot-repeatition); 60 days after inoculation (DAI), the plants were removed and evaluated according to reproduction factor (RF), nematode final population (FP) and reproducibility index (RI). Regarding weed hospitability, it was found that Luffa aegyptiaca acted as a host for both nematodes, with RF > 1, which was higher than the control sample. Digitaria horizontalis was classified as a host for M. incognita and as a non-host for M. javanica. Mucuna aterrima and Crotalaria spectabilis presented the lowest RF and FP. Ricinus communis and Ipomoea triloba presented galls when inoculated with M. incognita, but were not considered host, since they presented RF < 1. None of the weed species was considered immune, i.e., with RF = 0. The lowest RF values of M. incognita (race 3) were related to Crotalaria spectabilis and Euphorbia heterophylla plants. This demonstrates the ability of crotalarias in decreasing nematode population in the field and justifies its use in several areas before planting main crops.	Ligia, Ana/S-6761-2017; Monquero, Patricia Andrea P.A.M/C-3323-2012; Monquero, Patricia/N-6580-2019	Ligia, Ana/0000-0002-7452-0370; Monquero, Patricia/0000-0002-9123-1861	0100-8358	1806-9681	2017	35	e017156815	10.1590/S0100-83582017350100029	WOS:000401993700001

J	Al-Qura'n, S	Al-Qura'n, S.	Ethnopharmacological survey of wild medicinal plants in Showbak, Jordan	JOURNAL OF ETHNOPHARMACOLOGY	Two main research questions are framing this investigation: (1) the main taxa of the medicinal importance value altered the Showbak forest stand and species composition? (2) The most safe species and what are the toxic ones (unsafe). These two research questions are the vital ones to draw a clear image about the wild medicinal plants of this investigated area of Showbak region in Jordan. 79 wild medicinal plant species were investigated in this study which are used in traditional medication for the treatment of various diseases. Most of the locals interviewed dealt with well-known safe medicinal plants such as Aaronsohnia factorovskyi Warb. et Eig., Achillea santolina L., Adiantum capillus-veneris L., Artemisia herba-alba L., Ceratonia siliqua L., Clematis recta L., Herniaria hirsuta L, Malva neglecta Wallr., Rosmarinus officinalis L., Ruta chalepensis L., Salvia triloba L., Sarcopoterium spinosa (L.) Spach., Thymbra capitata (L.) Hof, and Urginea maritima Barker. Many of the wild medicinal plants investigated were toxic and needed to be practiced by practitioners and herbalists rather than the local healers. These plants include Calotropis procera Willd R.Br., Citrullus colocynthis (L.) Sch., Datura stramonium L, Digitalis purpurea L, Ecballium elaterium (L.) A.Rich., Euphorbia helioscopia L., Euphorbia tinctoria Boiss., Glaucium corniculatum (L) Curt., Hyoscyamus aureus L., Mandragora officinarum L., Nerium oleander L., Ricinus communis L., Solanum nigrum L., Withania somnifera (L.) Dunel. The conservation of medicinal plants and natural resources is becoming increasingly important, so this research is trying to collect information from local population concerning the use of medicinal plants in Showbak; identify the most important specie; determine the relative importance value of the species and calculate the informant consensus factor (ICF) for the medicinal plants. Obtaining results is relied on the interviewee's personal information and the medicinal use of specific plants. (C) 2009 Elsevier Ireland Ltd. All rights reserved.	0378-8741	1872-7573	MAY 4	2009	123	1	45	50	10.1016/j.jep.2009.02.031	WOS:000266124600008	19429338

J	Pfundel, E; Nagel, E; Meister, A	Pfundel, E; Nagel, E; Meister, A	Analyzing the light energy distribution in the photosynthetic apparatus of C-4 plants using highly purified mesophyll and bundle-sheath thylakoids	PLANT PHYSIOLOGY	The chlorophyll fluorescence characteristics of mesophyll and bundle-sheath thylakoids from plant species with the C-4 dicarboxylic acid pathway of photosynthesis were investigated using flow cytometry. Ten species with the NADP-malic enzyme (NADP-ME) biochemical type of C-4 photosynthesis were tested: Digitaria sanguinalis (L.) Scop., Euphorbia maculata L., Portulaca grandiflora Hooker, Saccharum officinarum L., Setaria viridis (L.) Beauv., Zea mays L., and four species of the genus Flaveria. This study also included three species with NAD-ME biochemistry (Atriplex rosea L., Atriplex spongiosa F. Muell., and Portulaca oleracea L.). Two C-4 species of unknown biochemical type were investigated: Cyperus papyrus L. and Atriplex tatarica L. Pure mesophyll and bundle-sheath thylakoids were prepared by flow cytometry and characterized by low-temperature fluorescence spectroscopy. In pure bundle-sheath thylakoids from many species with C-4 photosynthesis of the NADP-ME type, significant amounts of photosystem II (PSII) emission can be detected by fluorescence spectroscopy. Simulation of fluorescence excitation spectra of these thylakoids showed that PSII light absorption contributes significantly to the apparent excitation spectrum of photosystem I. Model calculations indicated that the excitation energy of PSII is efficiently transferred to photosystem I in bundle-sheath thylakoids of many NADP-ME species.	Meister, Armin/0000-0002-2281-8781	0032-0889	NOV	1996	112	3	1055	1070	10.1104/pp.112.3.1055	WOS:A1996VT97500021	12226432

J	Browne, EP; Morejon, M; Bonilla, M	Philbert Browne, Eric; Morejon, Mariol; Bonilla, Marta	Agroforestry establishment on the Jomane Nisbett farm in Cayon-Phillips	AVANCES	From the time of the English colonization, most of the arable and more accessible lands especially in the low and flat places were dedicated to the production of Saccharum officinarum L. (sugarcane). As a result, the farmers had to establish their small farm plots in the mountainous areas of the island and marginal places along the dry rivers. The objective of this work is the transformation of the productive systems of the farms in the Cayon-Phillips area by the development of agroforestry systems keeping in mind their geomorphologic characteristics. The development of the proposed agroforestry systems is carried out with the establishment of small parcels keeping in mind the changing morphological characteristics along the terrain using agroforestry techniques such as live fences, trees in rows, taungya method, etc. Iin each proposed variant it is recommended to use fruit trees such as Artocarpus altilis (Parkinson) Fosberg, Chrysobalanus icaco L., etc. and timber trees such as Inga laurina (Sw.) Willd and Guazuma ulmifolia Lam., etc. In the deforested properties a total of 11728 trees were proposed to be established on a surface of 10.5 hectares. To control the invasion of monkeys a live fence is designed using species such as Gliricidia sepium (Jacq.) Kunth., Euphorbia Haw, etc. The farm with the agroforestry development contributes 13600 kg/yr of Artocarpus altilis as non-timber forestry product for the local and export market.	1562-3297	JAN-MAR	2017	19	1	23	31	WOS:000418006000004

J	Mazoir, N; Benharref, A; Vaca, L; Reina, M; Gonzalez-Coloma, A	Mazoir, Noureddine; Benharref, Ahmed; Vaca, Laura; Reina, Matias; Gonzalez-Coloma, Azucena	Optimization of Insecticidal Triterpene Derivatives by Biomimetic Oxidations with Hydrogen Peroxide and Iodosobenzene Catalyzed by Mn-III and Fe-III Porphyrin Complexes	CHEMISTRY & BIODIVERSITY	Semisynthetic functionalized triterpenes (4 alpha,14-dimethyl-5 alpha,8 alpha-8,9-epoxycholestan-3 beta-yl acetate; 4 alpha,14-dimethyl-5 alpha-cholest-8-ene-3,7,11-trione; 4 alpha,14-dimethyl-5 alpha-cholesta-7,9(11)-dien-3-one and 4 alpha,14-dimethyl-5 alpha-cholest-8-en-3 beta-yl acetate), previously prepared from 31-norlanostenol, a natural insecticide isolated from the latex ofEuphorbia officinarum, have been subjected to oxidation with hydrogen peroxide (H2O2) and iodosobenzene (PhIO) catalyzed by porphyrin complexes (cytochrome P-450 models) in order to obtain optimized derivatives with high regioselectivity. The main transformations were epoxidation of the double bonds and hydroxylations of non-activated C-H groups and the reaction products were 25-hydroxy-4 alpha,14-dimethyl-5 alpha-cholesta-7,9(11)-dien-3 beta-yl acetate (59 %), 25-hydroxy-4 alpha,14-dimethyl-5 alpha-cholest-8-ene-3,7,11-trione (60 %), 4 alpha,14-dimethyl-5 alpha,7 beta-7,8-epoxycholest-9(11)-en-3-one (22 %), 8-hydroxy-4 alpha,14-dimethyl-5 alpha-cholest-9(11)-ene-3,7-dione (16 %), 12 alpha-hydroxy-4 alpha,14-dimethyl-5 alpha,7 beta-7,8-epoxycholest-9(11)-en-3-one (16 %), and 4 alpha,14-dimethyl-5 alpha,8 alpha-8,9-epoxycholestan-3 beta-yl acetate (26 %), respectively. We also investigated the insect (Myzus persicae,Rhopalosiphum padiandSpodoptera littoralis) antifeedant and postingestive effects of these terpenoid derivatives. None of the compounds tested had significant antifeedant effects, however, all were more effective postingestive toxicants onS. littoralislarvae than the natural compound 31-norlanostenol, with 4 alpha,14-dimethyl-5 alpha,8 alpha-8,9-epoxycholestan-3 beta-yl acetate being the most active. The study of their structure-activity relationships points out at the importance of C3 and C7 substituents.	1612-1872	1612-1880	SEP	2020	17	9	10.1002/cbdv.202000287	SEP 2020	WOS:000566806500001	32644248

J	Crous, PW; Wingfield, MJ; Guarro, J; Hernandez-Restrepo, M; Sutton, DA; Acharya, K; Barber, PA; Boekhout, T; Dimitrov, RA; Duenas, M; Dutta, AK; Gene, J; Gouliamova, DE; Groenewald, M; Lombard, L; Morozova, OV; Sarkar, J; Smith, MT; Stchigel, AM; Wiederhold, NP; Alexandrova, AV; Antelmi, I; Armengol, J; Barnes, I; Cano-Lira, JF; Ruiz, RFC; Contu, M; Courtecuisse, PR; da Silveira, AL; Decock, CA; de Goes, A; Edathodu, J; Ercole, E; Firmino, AC; Fouriem, A; Fournier, J; Furtado, EL; Geering, ADW; Gershenzon, J; Giraldo, A; Gramaje, D; Hammerbacher, A; He, XL; Haryadi, D; Khemmuk, W; Kovalenko, AE; Krawczynski, R; Laich, F; Lechat, C; Lopes, UP; Madrid, H; Malysheva, EF; Marin-Felix, Y; Martin, MP; Mostert, L; Nigro, F; Pereira, OL; Picillo, B; Pinho, DB; Popov, ES; Pelaez, CAR; Rooney-Latham, S; Sandoval-Denis, M; Shivas, RG; Silva, V; Stoilova-Disheval, MM; Telleria, MT; Ullah, C; Unsickern, SB; Merwe, NAD; Vizzini, A; Wagner, HG; Wong, PTW; Wood, AR; Groenewald, JZ	Crous, P. W.; Wingfield, M. J.; Guarro, J.; Hernandez-Restrepo, M.; Sutton, D. A.; Acharya, K.; Barber, P. A.; Boekhout, T.; Dimitrov, R. A.; Duenas, M.; Dutta, A. K.; Gene, J.; Gouliamova, D. E.; Groenewald, M.; Lombard, L.; Morozova, O. V.; Sarkar, J.; Smith, M. Th.; Stchigel, A. M.; Wiederhold, N. P.; Alexandrova, A. V.; Antelmi, I.; Armengol, J.; Barnes, I.; Cano-Lira, J. F.; Castaneda Ruiz, R. F.; Contu, M.; Courtecuisse, Pr. R.; da Silveira, A. L.; Decock, C. A.; de Goes, A.; Edathodu, J.; Ercole, E.; Firmino, A. C.; Fouriem, A.; Fournier, J.; Furtado, E. L.; Geering, A. D. W.; Gershenzon, J.; Giraldo, A.; Gramaje, D.; Hammerbacher, A.; He, X. -L.; Haryadi, D.; Khemmuk, W.; Kovalenko, A. E.; Krawczynski, R.; Laich, F.; Lechat, C.; Lopes, U. P.; Madrid, H.; Malysheva, E. F.; Marin-Felix, Y.; Martin, M. P.; Mostert, L.; Nigro, F.; Pereira, O. L.; Picillo, B.; Pinho, D. B.; Popov, E. S.; Pelaez, C. A. Rodas; Rooney-Latham, S.; Sandoval-Denis, M.; Shivas, R. G.; Silva, V.; Stoilova-Disheval, M. M.; Telleria, M. T.; Ullah, C.; Unsickern, S. B.; van der Merwe, N. A.; Vizzini, A.; Wagner, H. -G.; Wong, P. T. W.; Wood, A. R.; Groenewald, J. Z.	Fungal Planet description sheets: 320-370	PERSOONIA	Novel species of fungi described in the present study include the following from Malaysia: Castanediella eucalypti from Eucalyptus pellita, Codinaea acacia from Acacia man glum, Emarcea eucalyptigena from Eucalyptus brassiana, Myrtapenidiella eucalyptorum from Eucalyptus peffita, Pilidiella eucalyptigena from Eucalyptus brassiana and Strelitziana malaysiana from Acacia man glum. Furthermore, Stachybotrys sansevieriicola is described from Sansevieria ehrenbergii (Tanzania), Phacidium grevilleae from Grevillea robusta (Uganda), Graphium jumulu from Adansonia gregorii and Ophiostoma eucalyptigena from Eucalyptus marginata (Australia), Pleurophoma ossicola from bone and Plectosphaerella populi from Populus nigra (Germany), Colletotrichum neosansevieriae from Sansevieria L;/QSZLAKCVEQL,Atrifasciata, Elsinoe othonnae from Othonna quinquedentata and Zeloasperisporium cliviae (Zeloasperisporiaceae tam. nov.) from Clivia sp. (South Africa), Neodevriesia pakbiae, Phaeophleospora hymenocallidis and Phaeophleospora hymenocallidicola on leaves of a fern (Thailand), Melanconium elaeidicola from Elaeis guineensis (Indonesia), Hormonema viticola from Vitis vinifera (Canary Islands), Chlorrophyllum pseudoglobossum from a grassland (India), Triadelphia disseminata from an immunocompromised patient (Saudi Arabia), Colletotrichum abscissum from Citrus (Brazil), Polyschema sclerotigenum and Phialemonium limoniforme from human patients (USA), Cadophora viticola from Vitis vinifera (Spain), Entoloma flavovelutinum and Bolbitius aura ntiorugosus from soil (Vietnam), Rhizopogon granuloflavus from soil (Cape Verde Islands), Tulasnella eremophila from Euphorbia officinarum subsp. echinus (Morocco), Verrucostoma martinicensis from Danaea elliptica (French West Indies), Metschnikowia colchici from Colchicum autumnale (Bulgaria), Thelebolus microcarpus from soil (Argentina) and Ceratocystis adelpha from Theobroma cacao (Ecuador). Myrmecridium iridis (Mynnecridiales ord. nov., Myrmecridiaceae fam. nov.) is also described from Iris sp. (The Netherlands). Novel genera include (Ascomycetes): Budhanggurabania from Cynodon dactylon (Australia), Soloacrosporiella, Xenocamatrosporium, Neostrelitziana and Castanediella from Acacia man glum and Sabahriopsis from Eucalyptus brassiana (Malaysia), Readerielliopsis from basidiomata of Fuscopotia wahlbergii (French Guyana), Neoplatysporoides from Aloe ferox (Tanzania), Wojnowiciella, Chrysofolia and Neoeriomycopsis from Eucalyptus (Colombia), Neophaeomoniella from Eucalyptus globulus (USA), Pseudophaeomoniella from Olea europaea (Italy), Paraphaeomoniella from Encephalartos altensteinii, Aequabiliella, Celerioriella and Minutiella from Prunus (South Africa). Tephrocybella (Basidiomycetes) represents a novel genus from wood (Italy). Morphological and culture characteristics along with ITS DNA barcodes are provided for all taxa.	Wood, Alan/AAC-8733-2019; Pinho, Danilo Batista/D-3481-2014; Madrid, Hugo/AAA-4995-2019; Groenewald, Johannes Z/F-4667-2011; Groenewald, Marizeth/G-4976-2012; Ullah, Chhana/T-4309-2019; Gramaje, David/M-7006-2017; Unsicker, Sybille/AAW-6968-2021; Crous, Pedro W/H-1489-2012; Geering, Andrew/A-7912-2011; Dutta, Arun Kumar/AAH-2797-2020; Nigro, Franco/AAG-5755-2020; Acharya, Krishnendu/G-2317-2012; Martin, Maria/H-8069-2012; firmino, ana carolina/D-8225-2015; Popov, Eugene S/J-2481-2012; Restrepo, Margarita Hernandez/L-2720-2017; Gene, Josepa/AAG-6855-2019; Stchigel, Alberto/G-1261-2016; Lombard, Lorenzo/B-2042-2010; Furtado, Edson/AAX-8409-2021; Wiederhold, Nathan/ABB-1226-2021; SARKAR, JIT/AAW-7179-2021; Duenas, Margarita/H-4944-2015; Wingfield, Michael J/A-9473-2008; Gershenzon, Jonathan/K-1331-2013; Morozova, Olga/H-5135-2013; Malysheva, Ekaterina F./H-5582-2013; Sandoval-Denis, Marcelo/H-1112-2019; Alexandrova, Alina V/R-9859-2016; Ruiz, Rafael F. Castaneda/B-2511-2008; Telleria, M. Teresa/H-4928-2015; Cano-Lira, Jose F./ABB-5724-2020; Furtado, Edson/Q-1852-2018; Nigro, Franco/J-1499-2012	Wood, Alan/0000-0001-7315-3196; Pinho, Danilo Batista/0000-0003-2624-302X; Groenewald, Johannes Z/0000-0002-9474-6246; Groenewald, Marizeth/0000-0003-0835-5925; Ullah, Chhana/0000-0002-8898-669X; Gramaje, David/0000-0003-1755-3413; Unsicker, Sybille/0000-0002-9738-0075; Crous, Pedro W/0000-0001-9085-8825; Dutta, Arun Kumar/0000-0001-5234-3441; Acharya, Krishnendu/0000-0003-1193-1823; Martin, Maria/0000-0002-1235-4418; Popov, Eugene S/0000-0001-8599-3117; Restrepo, Margarita Hernandez/0000-0001-5157-3392; Gene, Josepa/0000-0001-6195-9299; Stchigel, Alberto/0000-0003-3987-7996; Lombard, Lorenzo/0000-0002-5942-5375; SARKAR, JIT/0000-0003-0756-5269; Duenas, Margarita/0000-0003-0621-8003; Gershenzon, Jonathan/0000-0002-1812-1551; Morozova, Olga/0000-0002-7329-528X; Malysheva, Ekaterina F./0000-0002-8507-2083; Sandoval-Denis, Marcelo/0000-0001-6832-0252; Alexandrova, Alina V/0000-0003-2359-4780; Ruiz, Rafael F. Castaneda/0000-0003-0063-3265; Telleria, M. Teresa/0000-0002-9876-6914; Cano-Lira, Jose F./0000-0003-4495-4394; Furtado, Edson/0000-0002-6924-835X; Mostert, Lizel/0000-0001-9063-2447; Vizzini, Alfredo/0000-0001-8390-6446; Shivas, Roger/0000-0001-7494-8716; Madrid, Hugo/0000-0002-7767-0796; Laich, Federico Salvador/0000-0002-4530-7474; Hammerbacher, Almuth/0000-0002-0262-2634; Barnes, Irene/0000-0002-4349-3402; Barber, Paul/0000-0002-7477-8697; Armengol, Josep/0000-0003-3815-8578; Nigro, Franco/0000-0002-8273-0052; Lopes, Ueder/0000-0001-5693-7976; Fourie, Arista/0000-0003-4931-7626; Marin Felix, Yasmina/0000-0001-8045-4798; Firmino, Ana Carolina/0000-0001-6684-0457; Wiederhold, Nathan/0000-0002-2225-5122; Guarro, Josep/0000-0002-7839-7568	0031-5850	JUN	2015	34	167	266	10

**References of REVIEW**

Reference

1.	A. Pritchard: “Introduction to the’Euphorbiaceae’.” Cactus & Company , 2003.

2.	A.R. Jassbi: “Chemistry and biological activity of secondary metabolites in Euphorbia from Iran.” Phytochemistry. vol. 67, no. 18, pp. 1977–1984, 2006.

3.	F.J. Evans and S.E. Taylor: “Pro-Inflammatory, Tumour-Promoting and Anti-Tumour Diterpenes of the Plant Families Euphorbiaceae and Thymelaeaceae.” In: Buchanan, J.G., Crews, P., Epe, B., Evans, F.J., Hanke, F.J., Manes, L.V., Mondon, A., Naylor, S., and Taylor, S.E. (eds.) Fortschritte der Chemie organischer Naturstoffe / Progress in the Chemistry of Organic Natural Products. pp. 1–99. Springer, Vienna (1983).

4.	Q.-W. Shi, X.-H. Su, and H. Kiyota: “Chemical and Pharmacological Research of the Plants in Genus Euphorbia.” Chem. Rev. vol. 108, no. 10, pp. 4295–4327, 2008.

5.	S. Shamsabadipour, M. Ghanadian, H. Saeedi, M.R. Rahimnejad, M. Mohammadi-Kamalabadi, S.M. Ayatollahi, and L. Salimzadeh: “Triterpenes and Steroids from Euphorbia denticulata Lam. With Anti-Herpes Symplex Virus Activity.” Iran J Pharm Res. vol. 12, no. 4, pp. 759–767, 2013.

6.	T.S. Wu, Y.M. Lin, M. Haruna, D.J. Pan, T. Shingu, Y.P. Chen, H.Y. Hsu, T. Nakano, and K.H. Lee: “Antitumor agents, 119. Kansuiphorins A and B, two novel antileukemic diterpene esters from euphorbia kansui.” Journal Of Natural Products. vol. 54, no. 3, pp. 823–829, 1991.

7.	M. Bailen, M.D. Khamlichi, A. Benharref, R.A. Martinez-Diaz, and A. Gonzalez-Coloma: “New Bioactive Semisynthetic Derivatives of 31-Norlanostenol and Obtusifoliol from Euphorbia Officinarum.” Natural Product Communications. vol. 11, no. 6, pp. 1934578X1601100609, 2016.

8.	N. Mazoir, A. Benharref, M. Bailén, M. Reina, A. González-Coloma, and R.A. Martínez-Díaz: “Antileishmanial and antitrypanosomal activity of triterpene derivatives from latex of two Euphorbia species.” Z. Naturforsch., C, J. Biosci. vol. 66, no. 7–8, pp. 360–366, 2011.

9.	S.F. Ahmad, S.M. Attia, S.A. Bakheet, A.E. Ashour, K.M.A. Zoheir, and A.R.A. Abd-Allah: “Anti-inflammatory effect of Euphorbia hirta in an adjuvant-induced arthritic murine model.” Immunological Investigations. vol. 43, no. 3, pp. 197–211, 2014.

10.	A. Smaili, N. Mazoir, L.A. Rifai, T. Koussa, K. Makroum, A. Benharref, L. Faize, N. Alburquerque, L. Burgos, M. Belfaiza, and M. Faize: “Antimicrobial Activity of two Semisynthetic Triterpene Derivatives from Euphorbia Officinarum Latex Against Fungal and Bacterial Phytopathogens.” Natural Product Communications. vol. 12, no. 3, pp. 1934578X1701200305, 2017.

11.	J.P. Peltier and F. Msanda: “Diversité et originalité de la steppe à Euphorbia officinarum L. subsp. echinus (HOOKER fil. et COSSON) VINDT du sud-ouest Marocain.” Feddes Repertorium. vol. 106, no. 3–4, pp. 215–229, 1995.

12.	J. BELLAKHDAR: “La pharmacopee marocaine traditionnelle.” Medicine arabe ancienne et savoirs populaires. vol. 189, 1997.

13.	G. Volpato, S.M. Lamin Saleh, and A. Di Nardo: “Ethnoveterinary of Sahrawi pastoralists of Western Sahara: camel diseases and remedies.” Journal of Ethnobiology and Ethnomedicine. vol. 11, no. 1, pp. 54, 2015.

14.	L.R. Huang, H. Luo, X.S. Yang, L. Chen, J.X. Zhang, D.P. Wang, and X.J. Hao: “Enhancement of anti-bacterial and anti-tumor activities of pentacyclic triterpenes by introducing exocyclic α,β-unsaturated ketone moiety in ring A.” Med Chem Res. vol. 23, no. 11, pp. 4631–4641, 2014.

15.	É. Jahandiez, R. Maire, and L. Emberger: “Catalogue des plantes du Maroc (spermatophytes et ptéridophytes).” Imprimerie Minerva, Alger , 1931.

16.	J.-P. Peltier: “La végétation du bassin versant de l’oued Sous (Maroc),” (1982).

17.	F. Msanda, A.E. Aboudi, and J.P. Peltier: “Originalité de la flore et de la végétation de l’Anti-Atlas sud-occidental (Maroc).” Feddes Repertorium. vol. 113, no. 7–8, pp. 603–615, 2002.

18.	A. Crousilles: “Usages, propriétés antibactériennes et physicochimie de miels marocains,” (2014).

19.	E. Hecker: “New toxic, irritant and cocarcinogenic diterpene esters from Euphorbiaceae and from Thymelaeaceae.” Pure and Applied Chemistry. vol. 49, no. 9, pp. 1423–1431, 1977.

20.	M. Ghourri: “La phytothérapie et les infections urinaires (La pyélonéphrite et la cystite) au Sahara Marocain (Tan-Tan).” . Vol. no. 3, pp. 23,.

21.	A. Ben Harref and J. p Lavergne: “Triterpenes from the latex of Moroccan euphorbia: Euphorbia resinifera, Euphorbia echinus and Euphorbia officinarum. Isolation and comparative 13C NMR study of the 4 tetracyclic triterpene classes: eupho-lanostane, elemo-lanostane, lanostane and 31-nor lanostane.” Bulletin de la Societe Chimique de France. 1985.

22.	M. Daoubi, A. Benharref, R. Hernandez-Galán, A.J. Macías-Sánchez, and I.G. Collado * †: “Two novel steroids from Euphorbia officinarum latex.” Natural Product Research. vol. 18, no. 2, pp. 177–181, 2004.

23.	N. Mazoir, A. Liazid, A. Auhmani, M. Daoubi, M. Dakir, A. Benharref, A. Kenz, and M. Pierrot: “1H and 13C NMR structural study of products obtained by hemisynthesis on the triterpenes isolated from latex of Euphorbia officinarum.” Physical & chemical news. no. 21, pp. 123–128, 2005.

24.	M. Daoubi, N. Marquez, N. Mazoir, A. Benharref, R. Hernández-Galán, E. Munoz, and I.G. Collado: “Isolation of new phenylacetylingol derivatives that reactivate HIV-1 latency and a novel spirotriterpenoid from Euporbia officinarum latex.” Bioorganic & medicinal chemistry. vol. 15, no. 13, pp. 4577–4584, 2007.

25.	N. Mazoir, A. Benharref, M. Bailén, M. Reina, and A. González-Coloma: “Bioactive triterpene derivatives from latex of two Euphorbia species.” Phytochemistry. vol. 69, no. 6, pp. 1328–1338, 2008.

26.	A. Smaili, N. Mazoir, L.A. Rifai, T. Koussa, K. Makroum, E.M. Kabil, A. Benharref, and M. Faize: “Triterpene derivatives from Euphorbia enhance resistance against Verticillium wilt of tomato.” Phytochemistry. vol. 135, pp. 169–180, 2017.

27.	R. Abboud, C. Charcosset, and H. Greige-Gerges: “Tetra- and Penta-Cyclic Triterpenes Interaction with Lipid Bilayer Membrane: A Structural Comparative Study.” J Membrane Biol. vol. 249, no. 3, pp. 327–338, 2016.

28.	S.G. Goijman, J.F. Turrens, G.B. Marini-Bettolo, and A.O. Stoppani: “[Inhibition of growth and macromolecular biosynthesis in Trypanosoma cruzi by natural products. Effects of miconidine and tingenone].” Medicina (B Aires). vol. 44, no. 4, pp. 361–370, 1984.

29.	S.G. Goijman and A.O.M. Stoppani: “Effects of β-lapachone, a peroxide-generating quinone, on macromolecule synthesis and degradation in Trypanosoma cruzi.” Archives of Biochemistry and Biophysics. vol. 240, no. 1, pp. 273–280, 1985.

30.	J.A. Urbina, J.L. Concepcion, S. Rangel, G. Visbal, and R. Lira: “Squalene synthase as a chemotherapeutic target in Trypanosoma cruzi and Leishmania mexicana.” Molecular and Biochemical Parasitology. vol. 125, no. 1, pp. 35–45, 2002.

31.	Y.V. Mishina, S. Krishna, R.K. Haynes, and J.C. Meade: “Artemisinins Inhibit Trypanosoma cruzi and Trypanosoma brucei rhodesiense In Vitro Growth.” Antimicrob Agents Chemother. vol. 51, no. 5, pp. 1852–1854, 2007.

32.	A. Smaili, N. Mazoir, L. Aicha Rifai, T. Koussa, K. Makroum, M. Belfaiza, A. Benharref, and M. Faize: “Induced resistance to wild fire disease of Nicotiana benthamiana using seed treated with triterpene derivatives from Euphorbia.” J Plant Pathol. vol. 100, no. 1, pp. 75–83, 2018.

33.	A. Smaili, L.A. Rifai, N. Mazoir, T. Koussa, L. Faize, N. Alburquerque, L. Burgos, K. Makroum, B. Malika, A. Benharref, and M. Faize: “Semisynthetic Triterpenes Derived from Euphorbia officinarum as Plant Growth Promoters and Inducers of Disease Resistance.” J Plant Growth Regul. vol. 38, no. 1, pp. 262–272, 2019.
